# A pipeline for the *de novo* assembly of the *Themira biloba* (Sepsidae: Diptera) transcriptome using a multiple k-mer length approach

**DOI:** 10.1186/1471-2164-15-188

**Published:** 2014-03-12

**Authors:** Dacotah Melicher, Alex S Torson, Ian Dworkin, Julia H Bowsher

**Affiliations:** Department of Biological Sciences, North Dakota State University, 1340 Bolley Drive, 218 Stevens Hall, Fargo, ND 58102 USA; Department of Zoology, Michigan State University, 328 Giltner Hall, East Lansing, MI 48823 USA

**Keywords:** Multiple k-mer, *de novo* assembly, Sepsidae, Transcriptome, Pipeline, Cloud computing

## Abstract

**Background:**

The Sepsidae family of flies is a model for investigating how sexual selection shapes courtship and sexual dimorphism in a comparative framework. However, like many non-model systems, there are few molecular resources available. Large-scale sequencing and assembly have not been performed in any sepsid, and the lack of a closely related genome makes investigation of gene expression challenging. Our goal was to develop an automated pipeline for *de novo* transcriptome assembly, and to use that pipeline to assemble and analyze the transcriptome of the sepsid *Themira biloba*.

**Results:**

Our bioinformatics pipeline uses cloud computing services to assemble and analyze the transcriptome with off-site data management, processing, and backup. It uses a multiple k-mer length approach combined with a second meta-assembly to extend transcripts and recover more bases of transcript sequences than standard single k-mer assembly. We used 454 sequencing to generate 1.48 million reads from cDNA generated from embryo, larva, and pupae of *T. biloba* and assembled a transcriptome consisting of 24,495 contigs. Annotation identified 16,705 transcripts, including those involved in embryogenesis and limb patterning. We assembled transcriptomes from an additional three non-model organisms to demonstrate that our pipeline assembled a higher-quality transcriptome than single k-mer approaches across multiple species.

**Conclusions:**

The pipeline we have developed for assembly and analysis increases contig length, recovers unique transcripts, and assembles more base pairs than other methods through the use of a meta-assembly. The *T. biloba* transcriptome is a critical resource for performing large-scale RNA-Seq investigations of gene expression patterns, and is the first transcriptome sequenced in this Dipteran family.

**Electronic supplementary material:**

The online version of this article (doi:10.1186/1471-2164-15-188) contains supplementary material, which is available to authorized users.

## Background

The Sepsidae family of flies consists of over 200 species with a global distribution [1]. Sepsids are a model system for the investigation of sexual selection and how it affects courtship and sexual dimorphism [2]. Sepsids have complex courtship behaviors that include elements of male display, female choice, and sexual conflict [3–6]. Specialized male traits have evolved alongside these complex courtship behaviors. Sexual selection has resulted in the evolution of modified forelimbs, body size, and abdominal appendage-like structures, which are articulated and have long bristles attached to their distal ends [7–15]. Next-generation sequencing in combination with gene expression analysis has the potential to answer multiple questions including: how new morphologies evolve, whether shared developmental mechanisms underlie traits that have evolved multiple times, what the genetic basis of sexual dimorphism is and how to resolve the phylogenetic relationships within Sepsidae. Despite the potential of sepsids as a model to test a wide variety of evolutionary hypotheses, almost no molecular resources exist in this family, nor are any genomes or EST databases available.

Most Dipteran families have few genomic resources compared to drosophilids and mosquitoes. Sepsids shared a common ancestor with *Drosophila melanogaster* and houseflies between 74 and 98 MYA, and are not closely related to any taxon with significant genomic resources [16, 17]. A detailed investigation of the *even*-*skipped* locus revealed that approximately twice as many nucleotide substitutions exist between coding regions of *D. melanogaster* and sepsid species as exists between *D. melanogaster* and the most distantly related *Drosophila* species [18]. The Sepsidae are a sister taxon to the Tephritoidea or true “fruit flies,” which contains four species with genomic and transcriptomic resources [19–22], but these are not as well annotated as *Drosophila* and the level of sequence similarity with sepsids is unknown. A sepsid transcriptome would not only facilitate gene expression studies across the Sepsidae, but would also enhance comparative bioinformatics within Diptera.

For non-model organisms, the challenge of gene discovery no longer resides in a dearth of sequence data, but from the computational challenges of large and complex datasets [23]. This challenge is particularly true for *de novo* assembly, which is more computationally intensive than syntenic assembly via mapping to a reference genome. Another hurdle to *de novo* assembly is recovering rare transcripts from a datasets with heterogeneous sequence coverage. Assemblies that combine multiple k-mer lengths generally recover a greater number of unique transcripts during *de novo* assembly than single k-mer approaches [24, 25], but with additional potential for mis-assembly. Although both cloud computing and multiple k-mer approaches are widely available, they have not been employed as broadly as reference-based pipelines because some programing knowledge is required.

Our objectives were two-fold: 1) to construct a general purpose *de novo* transcriptome assembly pipeline that compares the output of multiple programs and automatically analyzes this data for downstream applications, and 2) to use that pipeline to assemble the transcriptome of the sepsid *T. biloba*. Our pipeline uses Velvet-Oases and Trinity for the initial assembly and constructs a meta-assembly with CAP3 followed by analysis with various downstream programs, including BLAST and Blast2GO [26–29]. The pipeline functions on a low-cost cloud computing network, and can be operated from a standard desktop computer. In addition to assembling the *de novo* transcriptome of the sepsid fly *T. biloba*, we used this pipeline to re-assemble previously published transcriptomes that used both 454 and Illumina sequencing platforms. Compared to the standard single k-mer assembly, our pipeline assembles longer contigs and more base pairs in all four species. By comparing annotated transcripts from different assemblies of the *T. biloba* transcriptome, we demonstrate that our pipeline recovers a greater number of transcripts than standard approaches by pooling unique transcripts from multiple assemblies.

## Results

### General overview of computational pipeline

**Figure 1:**
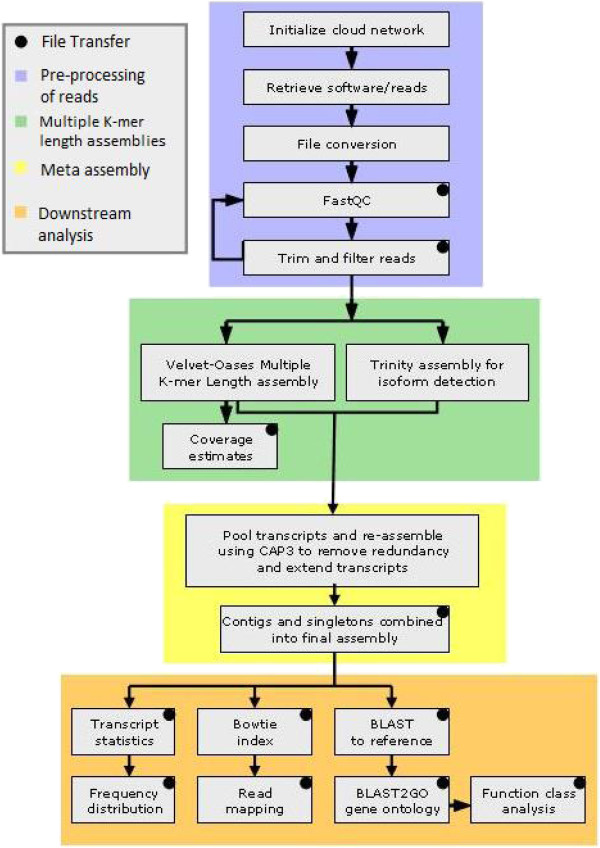
**Flowchart of the bioinformatic pipeline.** The pipeline performs multiple operations from sequence editing to annotation. First, a cloud network is initialized and algorithms are retrieved and installed. The sequence reads are parsed and filtered for quality and removal of adaptor sequences (blue). Next, assemblies are generated using various k-mer lengths and algorithms to create a diversity of transcript fragments (green). Then, the transcripts from all assemblies are pooled and re-assembled to remove redundant contigs and extend sequences based on overlap (yellow). The resulting multiple k-mer length meta-assembly is then analyzed and formatted for various downstream applications. Reads are mapped back to contigs, genes are annotated, and gene ontology is applied using BLAST and Blast2GO (orange). The pipeline generates an analysis of the assembly and the quantity and distribution of sequences. The resulting data is packaged in an archive for transfer and the cloud network is disbanded.

This pipeline was designed to automate a large number of intermediate bioinformatic activities such as trimming and filtering reads, converting sequence files through various formats, performing a large number of sequential assemblies using different assemblers and parameters, and formatting the output for downstream use (Figure 1). This pipeline was also designed to circumvent what have traditionally been significant limitations for small research groups, a lack of computing facilities and programing knowledge. This pipeline, while functional on a local network, is designed to make use of virtual cloud computing units, which provide scalable resources with direct interaction. Our pipeline produces intermediate products that are compatible with graphical user interface (GUI) based platforms such as The iPlant Collaborative and Galaxy, so that researchers can use these interfaces for downstream applications if desired [30–33].

We used this pipeline to perform the *de novo* assembly of the *T. biloba* transcriptome, the first transcriptome assembly for any species for the family Sepsidae. We also used the pipeline to re-assemble archived RNA-seq reads from other studies to assess the performance of the multiple k-mer length assembly process compared to a single k-mer assembly. Archived sequence from an arthropod (the milkweed bug, *Oncopeltus fasciatus*: [SRR:057573]), a plant (*Silene vulgaris*: [SRR:245489]), and a mammal (the ground squirrel *Ictidomys tridecemlineatus*: [SRR:352220]) were selected to test the performance of the pipeline across taxa and genome sizes. Each of these data sets consists of 454 sequence reads of approximately 3.2-4× coverage, the same coverage as our *T. biloba* data set. The *O. fasciatus* and *S. vulgaris* sequence reads were generated for *de novo* assembly of the entire transcriptome of the organism while the *I. tridecemlineatus* sequences were generated for differential expression analysis [34–36].

### Cloud computing network and data management

All of the data presented here were generated using Amazon Web Services Elastic Cloud Compute (AWS EC2) using a Debian Linux operating system (version 6.0.3). Software, sequence reads, reference assemblies, and other files are stored persistently on AWS Elastic Block Storage (EBS) volumes for the purpose of off-site backup, reduced network traffic, and storage. Data produced by the pipeline may be parsed and manipulated further through AWS or downloaded locally as needed. As presented here, the pipeline runs software in series. However, it is simple to create many duplicate systems through AWS, which may then run the processes in parallel.

Cloud computing instances were initialized using memory-optimized architecture to memory requirements the high memory requirements of Velvet-Oases assembly of 454 sequence reads. An instance with 64 gigabytes (GB) of available memory was used to during initial analysis of assembly performance at different k-mer lengths. This was sufficient to produce assemblies with a k-mer length up to 31 bp after which available memory became a limiting factor, which coincided with a reduction in assembly quality. At the time of this writing high-memory instance types with up to 244GB of available memory are available for larger data sets. Instances were initialized using a publically available Linux operating system disc image hosted by Amazon. Software, data, and scripts are stored on EBS volumes and software installation is simplified by a script that unpacks and installs all of the packages required for this pipeline to a newly created ‘bare’ cloud instance. All functional aspects of the pipeline shown in Figure 1 are performed by a wrapper script which sequentially performs the assembly and analysis of sequence data before storing it remotely and terminating the instance to minimize computing cost which is calculated in hourly blocks based on instance type. The pipeline ran to completion in approximately 20 hours. Larger sequence data sets requiring more memory and computing time may benefit from separating memory-intensive assembly from processor-intensive downstream analysis as the cost of processing with cloud computing is much lower than reserving large blocks of memory and storage space.

### Trimming and quality filtering reads

Prior to assembly, the reads are processed to remove adaptor sequences, low-quality reads and regions, and highly redundant sequences. The initial quality of the untrimmed sequence reads is assessed using FastQC, which also generates a list of over-represented sequences which may then be removed [37]. The raw sequence reads are then converted to a standard format which is passed on to the FastX Toolkit which removes adaptor sequences using trimming and clipping functions [38]. The reads are subsequently run through the FastX quality filter which removes reads that fail to pass a quality check (80% of the bases having a Phred score of 20 or higher, corresponding to a 1:100 base-calling error rate were used for the data presented here). The remaining reads are analyzed for redundancy by FastX and then collapsed into a single representative read. This removes large numbers of identical reads that may result from the amplification process prior to sequencing. Reducing the number of reads can dramatically reduce the amount of memory needed during the assembly process. It can also significantly reduce the amount of time required for assembly, which is an important consideration when generating multiple assemblies [39].

### Assembly

It has been shown that performance varies significantly between assemblers and data sets [40]. This has prompted the development of a number of techniques, such as multiple-k approaches, to retrieve more contigs from the initial sequence reads [25, 41–44].

To assemble the *T. biloba* sequence reads we have used a multiple k-mer length approach that creates a large number of assemblies, each of which contains potentially unique transcripts. Because many assembly programs can support multiple k-mer assembly after the addition of custom scripts, we compared the performance of four different assembly programs: Abyss, Newbler, Trinity and Velvet-Oasis, using a previously described protocol (Additional file 1: Table S1) [26, 27, 40, 45–48]. *T. biloba* sequence reads from multiple life stages were pooled and assembled with a k-mer length of 25 using each of the four assembly programs (Table 1). The resulting transcripts were then aligned to the *D. melanogaster* transcriptome. A conservative cut-off value with a minimum aligned length of 400 bp was used to create the distribution in Table 1. While Velvet-Oases produced the longest contigs, Trinity generated a larger number of contigs. A nucleotide BLAST of contigs in each assembly showed an increase in the number of contigs unique to one assembly in those produced by Trinity and Velvet-Oases. Based on these results, Velvet-Oases was selected for the length of the resulting transcripts and the ease of generating assemblies of different k-mer lengths, and a single Trinity assembly is included to provide isoform detection. The Velvet-Oases and Trinity *de novo* assembler algorithms have complementary strengths and weaknesses when comparing memory requirements and run-time.

**Table 1:** **Comparison of assemblers and identification of unique transcripts**

Assembler	Contigs	Contig n50	BLAST hits	Unique hits
Velvet-Oases	18960	296	5114	1817
Abyss	19664	127	5341	1566
Newbler	13398	208	4302	1509
Trinity	25144	244	6826	2194

**Figure 2:**
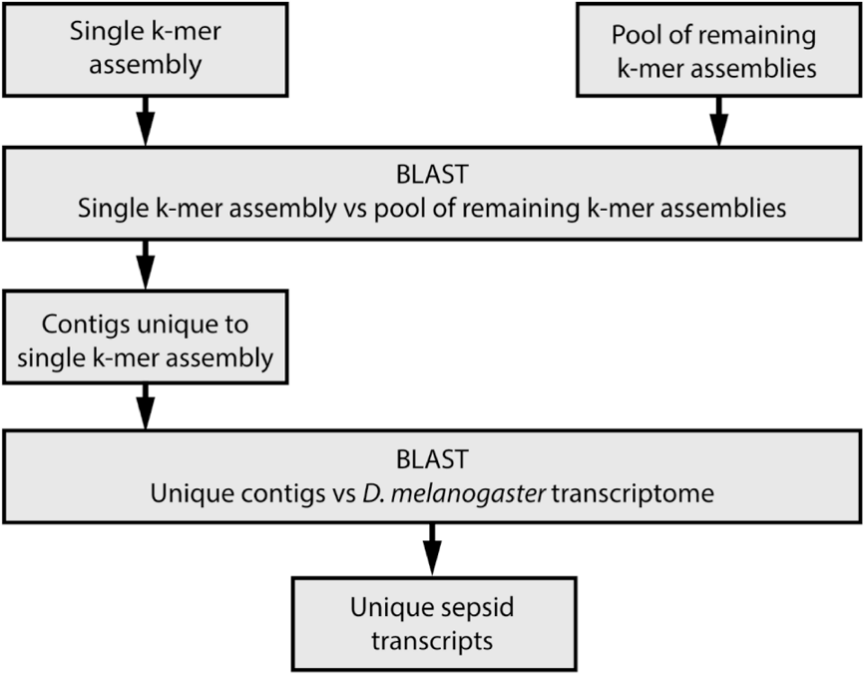
**BLAST strategy to identify unique transcripts.** Identification of unique transcripts in each individual assembly was performed by reserving contigs from one assembly and pooling all contigs from the remaining assemblies. The contigs from the single assembly were aligned to the pooled contigs. Contigs that fail to align were considered unique to that single assembly. The unique contigs were annotated by aligning to the *D. melanogaster* transcriptome.

The *T. biloba* sequence data was used to generate assemblies with k-mer lengths of 17, 19, 21, 23, 25, 27, 29, and 31 base pairs. To demonstrate that assemblies with different k-mer lengths recover unique transcripts, the stand-alone BLAST algorithm was used to align contigs from each assembly to a pool of contigs from all assemblies, with the resulting unaligned contigs representing those unique to one assembly (Figure 2). For example, to determine the number of contigs unique to the K17 assembly, the K17 contigs were blasted against the pooled contigs from all other assemblies. If a contig did not align, then it was unique to the k17 assembly. Contigs were discarded that were less than 200 base pairs. Next, BLAST was performed against *D. melanogaster* to annotate the unique contigs, and only those contigs with orthology to *D. melanogaster* were reported (Table 2). After the initial analysis, the pooled assemblies were also annotated using the *D. melanoga*ster transcriptome to generate a total number of transcripts for the pool, to which the number of unique transcripts could be compared (Table 2). A significant number of transcripts were represented in only one of the single k-mer length assemblies (Table 2). In total, 2,296 transcripts were identified as unique to a specific assembly using BLAST analysis. For k-mer lengths 17–27, unique transcripts were approximately 2% of each assembly, and this percentage did not decrease with increasing k-mer length. However, at K29, unique transcripts decreased to only 0.8% of the total. The number of unique transcripts generated from this analysis is a low estimate because it contains only conserved *Drosophila* orthologs, and excludes transcripts unique to *T. biloba* and those too divergent to be identified by BLAST. Therefore, the number of unique transcripts recovered from different k-mer assemblies is likely higher. Our analysis confirms that restricting assemblies to only a single k-mer length limits the number of transcripts recovered, regardless of which k-mer length is chosen.

**Table 2:** **Unique transcripts per k**-**mer length in paired**-**end assemblies using Velvet**-**Oases**

K-mer length	Total transcripts	Transcripts absent from one or more assemblies	Transcripts unique to one assembly	% unique transcripts
17	21296	2331	464	2.2
19	20080	2105	397	2.0
21	17950	1875	410	2.3
23	16668	1686	316	1.9
25	15894	1434	280	1.8
27	15496	2398	313	2.0
29	15138	3855	116	0.8
Total	122522	15684	2296	1.9

### Meta-assembly

The assemblies generated with k-mer lengths of 23, 25, 27, and 29 base pairs were combined through meta-assembly which extends contigs found in multiple assemblies and retains contigs found in only one. K-mer lengths shorter than 23 resulted in a large number of singletons and short contigs. Assemblies with a k-mer length larger than 29 required much larger memory allocations and computational time and were more conservative than other assemblies resulting in diminishing returns in which larger k-mer word sizes produce few novel transcripts not present in other assemblies.

The CAP3 software was used to construct the meta-assembly [28]. The CAP3 software removes the redundancy generated within and between assemblies of different k-mer lengths to consolidate the transcripts. Consolidating the results of all k-mer assemblies created a pool of 138,954 contigs. CAP3 clustered and assembled these sequences into a meta-assembly of 15,984 extended contigs and 8,511 singletons. The singletons represent sequences for which no overlap exists between assemblies and thus could not be extended by CAP3. The final meta-assembly consisted of 24,495 contigs with a mean sequence length 1,403 base pairs, an increase of 372 bp (34.1%) compared to the K25 assembly.

**Figure 3:**
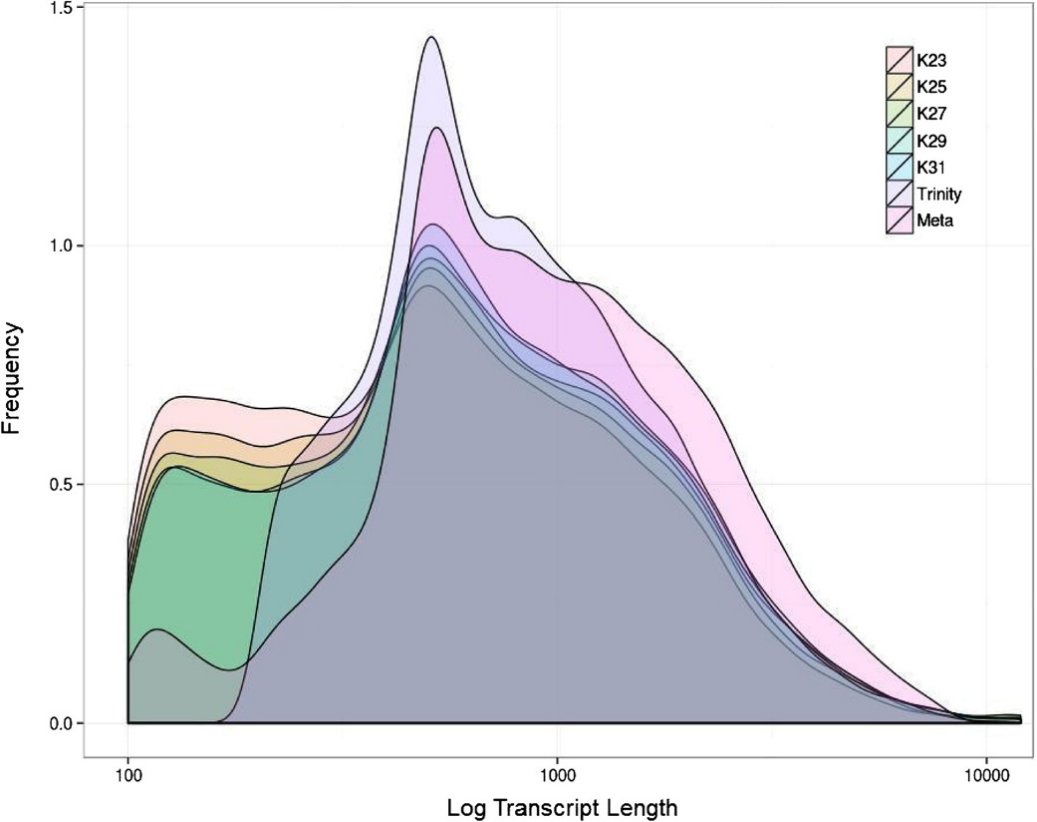
**Frequency distribution of transcript lengths by assembly.** A plot of the quantity of transcripts with a given length per assembly shows differences in assembly output and a pronounced peak representing the median transcript length. The meta-assembly was generated by the re-assembly of all k-mer lengths using CAP3. Meta-assembly improved transcript length, as indicated by the leading edge of the graph. Meta-assembly also reduced the number of short contigs, compared to the single k-mer assemblies. Trinity automatically removes contigs smaller than 200 base pairs.

**Figure 4:**
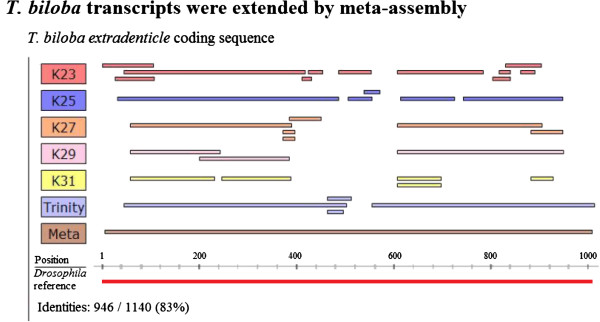
**Extension of** ***extradenticle*** **sequence by meta**-**assembly.** Contigs generated by multiple k-mer lengths were consolidated by meta-assembly to recover the entire coding sequence of the gene *extradenticle* from sequence fragments. Contigs from individual assemblies of multiple k-mer lengths are shown in alignment to the meta-assembly and the *Drosophila* transcript. The k-mer length 31 contigs were not included in the meta-assembly and show a reduction in coverage compared to other assemblies. Assemblies with shorter k-mer lengths also show a reduction in coverage but are not shown due to excessive fragmentation which results in a large number of short contigs that cannot be confidently aligned. The extended transcript aligns to the full length of the *Drosophila* reference sequence with 83% nucleotide sequence conservation.

Analysis of transcript length revealed that the total number of base pairs assembled improved significantly from 17.4 Mb to 32.7 Mb and the mean contig length increased by 310 bp from 1,093 bp to 1,403 bp. A frequency distribution of the number of contigs of a given length (Figure 3) shows an increase in the number of longer contigs in the meta-assembly, compared to the single k-mer assemblies and the Trinity assembly. The single k-mer assemblies have a relatively high number of singletons (sequences of less than 500 bp). The number of singletons was greatly reduced in the meta-assembly, indicating that meta-assembly was able to extend contigs by incorporating singletons. To demonstrate that contigs from different k-mer assemblies were used to create extended consensus contigs, genes from a candidate list of transcription factors were tracked from the 454 reads through the assembly and meta-assembly process (Table 3). Transcription factors are generally low abundance transcripts, and therefore full-length sequences are less likely to be recovered in single k-mer assemblies. Five out of the seven transcripts were extended through CAP3 re-assembly (Table 3). Primers were designed for four sequences and PCR amplification using *T. biloba* cDNA produced bands of the expected size, indicating that these extended contigs are correctly assembled transcripts (Additional file 2: Figure S1). To better visualize how meta-assembly extends transcript length, we examined in further detail how *extradenticle* contigs from different assemblies were meta-assembled (Figure 4). The meta-assembly recovered the entire length of the coding sequence of the *Tbil*-*exd* transcript, as compared to *Drosophila*. Assembling the full transcript required contigs from multiple assemblies, and only a subset of the individual assemblies contained sequences fragments for the middle of the transcript. Contigs from assemblies outside the 23–29 k-mer range show a reduction in coverage caused by fragmentation in assemblies with shorter k-mer lengths and conservative assembly with larger k-mer lengths. The *Tbil*-*exd* sequence contains several single nucleotide insertions within the region aligned to the *Drosophila* reference and 83% of the nucleotide identities are conserved.

**Table 3:** **Transcripts of interest extended by meta**-**assembly**

Identity	Meta-assembly	Individual assembly
Engrailed*	1140	1140
Escargot*	1244	782
Evenskipped*	876	717
Extradenticle	1143	574, 417, 138
Hunchback	800	699, 472
Sex-combs reduced	232	281
Ultrabithorax	1084	526, 368, 370, 874
*Validated by PCR		

**Figure 5:**
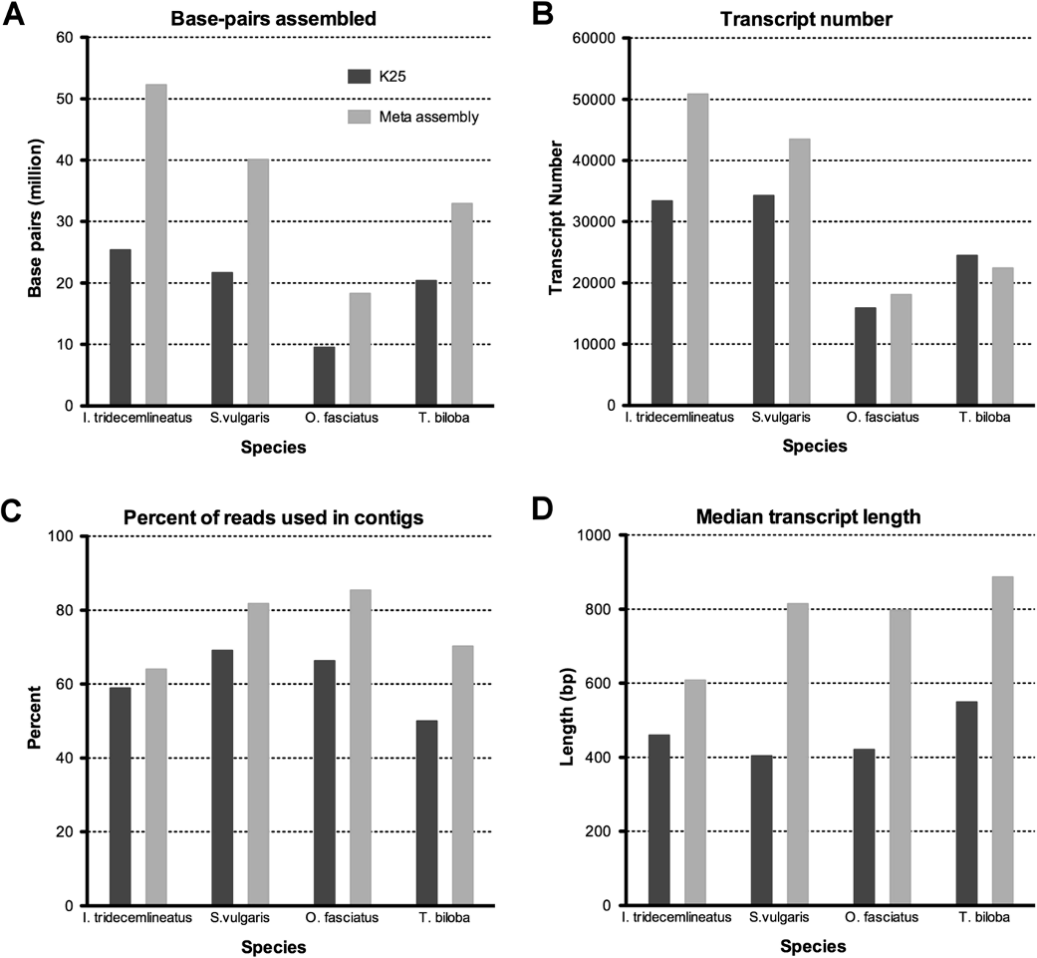
**Performance of meta**-**assembly across species.** A single assembly using Velvet-Oases with a K-mer length of 25 (light gray) was compared to the multiple k-mer length meta-assembly (black) for four species. Meta-assembly improved overall transcript length. The total assembled base-pairs **(A)**, transcript number **(B)**, percent of reads used in contigs **(C)**, and median transcript length **(D)** show improvement in transcript assembly.

To determine whether meta-assembly would improve transcriptome quality across taxa, the meta-assembly process was performed on three archived datasets (*Oncopeltus fasciatus*: SRR057573; *Silene vulgaris*: SRR245489; *Ictidomys tridecemlineatus*: SRR352220) using the same pipeline used to generate the *T. biloba* transcriptome. (Table 4; Figure 5). The meta-assemblies for each of the four datasets were compared to a single 25 k-mer length assembly.

**Table 4:** **Single and multiple k**-**mer length meta assembly across 4 species**

Assembly	Base- pairs	n	Median	Mean	n50	% reads used
*I. tridecemlineatus*						
25-mer assembly	25446725	33363	460	762	1328	49.97%
Meta assembly	52328097	50869	608	1028	1708	70.26%
*S. vulgaris*						
25-mer assembly	21706584	34262	404	633	1124	66.31%
Meta assembly	40068740	43475	815	921	1351	85.45%
*O. fasciatus*						
25-mer assembly	9487925	15886	421	597	894	69.12%
Meta assembly	18283749	18106	797	1009	1312	81.80%
*T. biloba*						
25-mer assembly	20431185	22423	549	911	1571	58.87%
Meta assembly	32887248	24495	887	1342	2010	64.01%

We used multiple metrics to compare transcription quality between the 25 k-mer length assembly and the meta-assembly including: number of base pairs assembled, number of contigs, percent of reads used in the contigs, and median contig length (Figure 5; Table 4). In all four datasets, the number of base pairs assembled was greater in the meta-assembly. The greatest increased was observed in *I. tridecemlineatus* in which the number of base pairs assembled doubled with meta-assembly. Overall, the total number of assembled base pairs is 60.1% to 105.6% greater. The increase in base-pairs assembled was mirrored by an increase in contig length in all four species, as measured by mean contig length, median contig length, and n50 (Figure 5D; Table 4). The increase in length is presumably a result of incorporating more reads, because the percent of total reads that were assembled into contigs also increased with meta-assembly (Figure 5B). In addition to increasing contig length, the meta-assembly also increased contig number in the *I. tridecemlineatus*, *S. vulgaris*, and *O. faciatus*, data sets (Figure 5B). The increase in contig number is further evidence that meta-assembly recovers unique contigs from different k-mer length assemblies. The gain in contig number was likely even greater than the observed increase because the 25 k-mer assembly includes redundant contigs, whereas the meta-assembly does not. The same pre-processing steps were used to generate the filtered reads for both the 25 k-mer and meta-assemblies but the 25 k-mer assemblies did not undergo a secondary assembly to remove internal redundancy. When applied to a single Velvet-Oases assembly, CAP3 reduces the number of contigs by 5.5%. The only species to see a reduction in the number of contigs after meta-assembly was *T. biloba*. We hypothesize this reduction was due to either elimination of duplicates, consolidation of contigs, or both.

### Alignment and annotation of the *Themira biloba*transcriptome

The *T. biloba* transcriptome was annotated using the *D. melanogaster* transcriptome as a reference. The pipeline aligned the *T. biloba* transcripts to *D. melanogaster* using the standalone BLAST package and a reference database available from FlyBase [49]. 11,008 transcripts from the meta-assembly were identified via BLAST as homologous to *Drosophila* sequences (44.9%). We found that the aligned *T. biloba* sequences were 82.3% conserved (mean sequence conservation taken from a subset of 500 BLAST hits) indicating that BLAST may not be sufficient to identify some sequences. Therefore, sequence divergence between the two species could explain why over half the *T. biloba* contigs in the meta-assembly could be annotated based on *Drosophila*. However, contig mis-assembly could also cause low annotation rates. To determine whether sequence divergence or mis-assembly was the cause, we annotated the T. biloba transcriptome with a more closely related Dipteran.

Sepsidae is more closely related to Tephritidae than the drosophilids [17], so it would be expected that higher sequence conservation exists between these two families, and that comparison to a tephritid would identify more transcripts. To determine whether such a comparison would identify more transcripts than *Drosophila*, a transcriptome was constructed using archived Illumina sequence reads from adult male and female *Bactrocera dorsalis* (SRR818498, SRR818496) [50]. Bi-directional alignments were created using *T. biloba*, *B. dorsalis*, and *D. melanogaster*. Contrary to our prediction, the alignments between *T. biloba* and *B. dorsalis* did not show increased aligned contigs or even conserved sequence versus *Drosophila* (Table 5). On average, *B. dorsalis* had around the same sequence similarity to *T. biloba* that *Drosophila* did, and the number of matching transcripts actually decreased, as did the average length of the matching region. The decrease in number of matches may be due to the nature of the datasets. The *Drosophila* transcriptome includes multiple life stages and has a high level of coverage, whereas the *B. dorsalis* transcriptome only includes the adult stage [50]. Decreased representation could result in alignment of fewer genes even though the amount of sequence divergence is similar. In the end, annotation to *B. dorsalis* had the same limitations as Drosophila because of sequence divergence in the Sepsidae lineage.

**Table 5:** **BLAST matches and percent identities**

Query	Database	Matched	Unmatched	Mean length	Mean % conserved
*T. biloba*	*D. melanogaster*	11008	13487	1200	82.21%
*T. biloba*	*B. dorsalis*	6273	18222	729	82.46%
*B. dorsalis*	*D. melanogaster*	9334	41053	802	84.17%
*B. dorsalis*	*T. biloba*	6277	40273	726	85.99%
*D. melanogaster*	*T. biloba*	13544	23852	1336	82.37%
*D. melanogaster*	*B. dorsalis*	10333	26337	794	83.62%

To determine whether comparison with other more complete databases could increase the number of annotated contigs, the contigs from the *T. biloba* meta-assembly were compared to the SwissProt databases. SwissProt has the ability to compare translated contigs, thus reducing the problem posed by nucleotide divergence. Additional transcripts were annotated through BLASTx against the SwissProt database, which had not been annotated through the comparison with *D. melanogaster*. An expect-value cutoff of 0.00001 resulted in alignment of 16,705 (68.2%) of the translated sequences to sequences in the SwissProt database, which was a difference of 5,697 contigs (23.2%) compared to nucleotide BLAST against a single species. Analysis was performed to determine known protein domains in the Pfam database using the Trinity utility TransDecoder [51]. An additional 221 contigs that had not been annotated were found to contain Pfam domains increasing the number of contigs identified by at least one searched database to 16,926 (69.1%). The number of annotated contigs compares favorably to other *de novo* assemblies [52–54]. The high percentage of annotated transcripts indicates that the contigs generated through meta-assembly are true transcripts, and not mis-assembled contigs. Further improvements in annotation likely require greater coverage through increased sequencing depth and a larger sequence data set.

**Figure 6:**
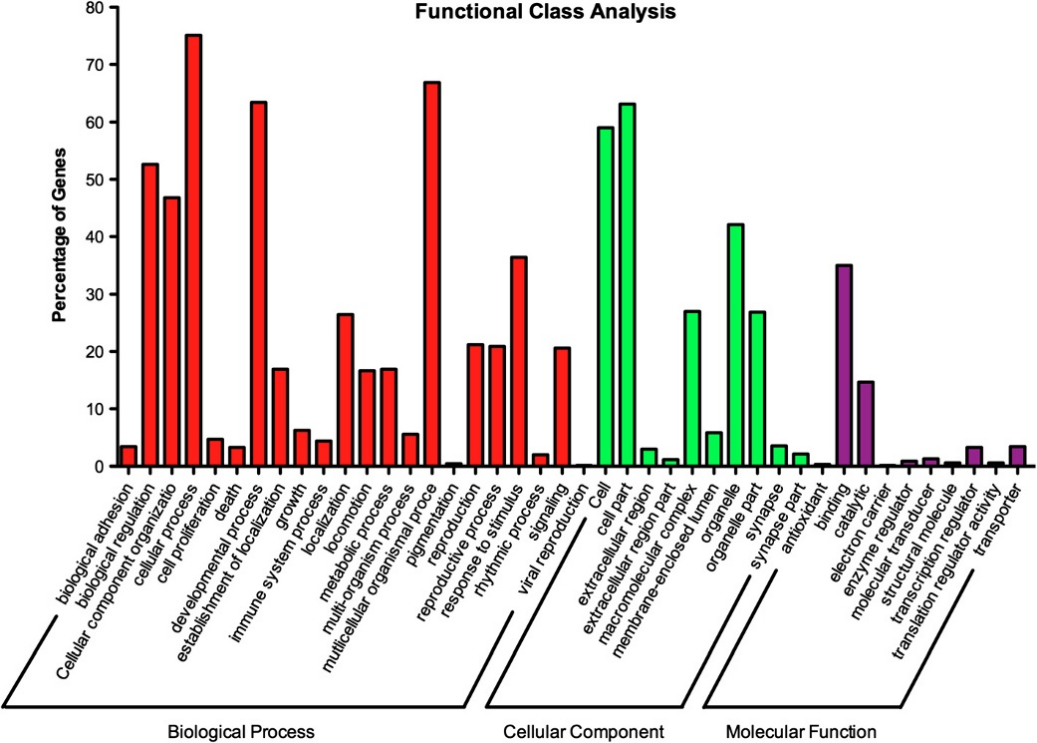
**Gene Ontology classification of the** ***T. biloba*** **transcriptome.** Gene Ontology (GO) was assigned to all contigs from the *T. biloba* meta-assembly. Gene ontologies were group into three main categories and 42 sub-categories. Contigs are grouped by the percentage of sequences that match a specific GO term within three major groups. The most abundant transcripts represent the sub-categories containing structural proteins and regulators of various cellular processes.

To determine ontology, *T. biloba* transcripts were submitted for KEGG pathway analysis resulting in 5,080 contigs with identified functions. Many developmentally import pathways involved in cell signaling such as the notch pathway were near complete (Additional file 3: Table S2). Transcripts were assigned gene ontologies, which were then grouped by function (Figure 6) to determine whether the transcripts recovered from the meta-assembly were representative of the main cellular processes. A broad range of functional groups were present in the assembly, indicating that transcripts representing many different kinds of proteins were recovered. The distribution of contig gene ontologies is similar to those found in the distribution of GO terms found in the *Drosophila* transcriptome and other *de novo* transcriptome assembly efforts [34, 55, 52, 54].

## Discussion

### Bioinformatics and data management

The *de novo* assembly of a transcriptome presents multiple challenges including computational requirements and accurate assembly of low abundance transcripts. Here we present a pipeline for *de novo* assembly that uses cloud computing and a multiple k-mer meta-assembly processes. The strength of a distributed, cloud-based approach to transcriptome assembly and sequence analysis is its versatility and the low initial investment in data processing [23, 56]. We have found the primary advantage of hosting data analysis off-site is the ability to construct a low-cost, scalable network on demand with unrestricted access. The increased computing power is particularly important when generating multiple *de novo* assemblies, as is done in our meta-assembly processes. Meta-assembly processes that use a multiple k-mer length approach have been previously demonstrated to significantly improve the quality of transcriptomes [24, 57].

The pipeline presented here incorporates an extensive and automated toolkit for parsing and trimming sequence reads prior to multiple k-mer assembly and the generation of a meta-assembly that best represents the transcripts available to be recovered. Automated sequence analysis tools are included to provide graphical views of read quality, transcript length and coverage per assembly, transcript extension, annotation information of sequence homologs from various databases, and the presence of unique sequences, and the assembly parameters used to recover the sequences.

### Increasing transcriptome quality with meta-assembly

We validated our pipeline by assembling three previous published transcriptomes and the transcriptome of the sepsid fly *T. biloba*, which was sequenced as part of this project. Transcriptome quality was compared between our pipleline, which employs a meta-assembly process, and the standard practice of using a single 25 bp k-mer length for assembly. In all four species, the meta-assembly increased the number of base pairs assembled, increased the length of contigs, increased the percentage of reads used in the contigs and recovered a greater number of transcripts than the 25 k-mer assembly. The increased quality of meta-assembly was further investigated in the *T. biloba* transcriptome by tracking the improvement in a candidate list of low abundance transcripts. For a subset of these transcripts, RT-PCR confirmed that meta-assembly increased the length of the transcripts by connecting fragments recovered from multiple k-mer length assemblies.

## Conclusions

We have assembled transcript sequences from the complete life cycle of *T. biloba*, a sepsid fly which exhibits primary gain of a novel trait, and identified many developmentally important genes. These transcripts represent the first large-scale sequencing that has been performed within the family Sepsidae, a large and diverse family with over 250 species distributed globally. Sepsid flies have been used for taxonomic and behavioral studies and have diverse genital and appendage morphologies, but lack of sequence data has made genetic investigation of these traits difficult [58, 9, 4, 8, 11, 2]. While many orthologous genes retain their functions between dipterans, large regions of gene sequence are often not conserved [18, 59].

The *T. biloba* transcriptome and many of the genes we have identified will be used for future RNA-Seq studies of comparative gene expression, knockdown, and *in situ* hybridization experiments. Sequence for many developmentally important genes and transcription factors of interest were obtained including members of the HOX family and those associated with embryonic and morphological development. In addition, many sequences for genes involved in cell signaling pathways such as notch and torso signaling were recovered. Sequence for the *T. biloba doublesex* ortholog as well as several transcripts associated with mating and courtship in *Drosophila* were also recovered which aids investigation of the sepsid sex allocation pathway and the genetic mechanisms behind behavioral traits associated with the sepsid novel appendage.

As more genomes become available, researchers using non-model organisms will have the opportunity to assemble RNA-seq reads to reference genomes of closely related species. Assembling to a reference, when available, yields a higher quality transcriptome than *de novo* assembly, and this result is robust to low-levels of genomic divergence between species [42, 44]. Although these findings are encouraging, those working with non-model organisms should proceed with caution [60]. Based on *in silico* studies, assembling to a reference that has a sequence divergence greater than 15% decreases the number of transcripts recovered compared to *de novo* assembly [44]. In our case, assembling the *T. biloba* reads to the *Drosophila* genome would have been inappropriate because the 17% sequence divergence between the two species would have resulted in decreased transcript recovery compared to *de novo* assembly. Choosing a closer relative based on phylogeny does not necessarily solve the problem, as our additional comparison to *B. dorsalis* revealed. Because the amount of sequence divergence between a non-model organism and its closely related reference species is rarely known prior to high-throughput sequencing, *de novo* assembly remains a powerful tool for recovering transcripts in non-model organisms.

## Methods

### *T. biloba*colony

Cultures of *T. biloba* were maintained in an incubator at 25C with a 16:8 hour light–dark cycle in overlapping generations. Larvae were raised in Petri dishes and fed agar mixed with soy infant formula (ProSobee) covered with a 1.0 cm layer of cow dung. Adults were fed honey mixed with water and provided with cow dung to facilitate mating and egg-laying.

### Tissue collection

Tissue was collected from embryos, 3^rd^ instar larva, and 48–72 hour pupa. During collection all material was stored at -80°C in RNALater, prior to shipment to the sequencing facility. Embryos were collected regularly and washed several times with an egg wash solution of 0.12 M NaCl and 0.01% Triton X-100 to remove dung. The eggs were dechorionated using a 3% bleach solution. Third instar, wandering-phase larvae were everted in PEM buffer (100 mM PIPES-disodium salt, 2.0 mM EGTA, 1.0 mM MgSO_4_ anhydrous, pH 7.0) to facilitate RNA extraction. Prior to pupation, gut-purged larvae were allowed to wander on moistened filter paper to remove dung and particulates. Pupae were staged to 48–72 hours before collection. All samples were stored in RNALater overnight at 4°C and transferred to -80°C for storage prior to sequencing.

### Sequencing

RNA isolation, library cDNA preparation, and 454 sequencing were performed by the University of Arizona Genetics Core (UAGC). Prior to sequencing, the cDNA was screened using a 2100 Bioanalyzer (Agilent Technologies). Sequencing was done on a GS FLX Titanium (454 Life Sciences). Embryos, larvae, and pupae were sequenced separately, creating 3 separate pools of sequence. Approximately 1.48 million reads total with an average length of 400 bp were generated.

### Assembly and annotation

Pre-processing of the sequence reads generated from *T. biloba* was performed using the FastX Toolkit [38]. Adaptor sequences were removed using the trimmer function. The quality filter removed sequences in which 80% of the base pairs had a Phred score of less than 20. The remaining 1.01 million reads were then converted to FASTA. The FastX collapsing tool was used to consolidate redundant sequences to reduce the amount of memory needed during the assembly process. An assembly was performed using the collapsed reads to determine the reduction in memory required for assembly (Additional file 4: Figure S2). We determined that although collapsing the reads significantly reduced the memory requirements for assembly, it was not necessary for the data sets described in this publication and may lead to a reduction in coverage. FastQC (v0.10.1) was used to assess the quality of reads before and after pre-processing [37].

Paired-end assemblies with K-mer lengths of 19 to 29 were generated using Velvet-Oases with an insert size of 200 bp [26, 27]. Trinity was used to generate an additional paired-end assembly [47, 48]. The resulting contigs were aligned to *Drosophila* using standalone BLAST to identify developmentally important transcripts. A BLAST alignment was then performed using each individual assembly as the query and the pooled contigs from all other assemblies as the database to identify contigs unique to each assembly. The assemblies were then concatenated and the pool of 138,954 transcripts was re-assembled using CAP3 [28].

## Additional files

The 454 reads from this study have been archived at the NCBI Sequence Read Archive under BioProject [PRJNA:218740]. Custom scripts for assembly and analysis of the *T. biloba* transcriptome and a disc image of the complete pipeline with all programs and scripts used in this pipeline is available at https://sourceforge.net/projects/themiratranscriptome.

## Electronic supplementary material

Additional file 1: **FastQC reports for untrimmed and trimmed sequence reads.** Quality reports generated before and after quality filtering and trimming show an improvement in multiple quality metrics. (ZIP 2 MB)

Additional file 2: **PCR validation of assembled contigs.** Primers designed from bioinformatically generated contigs annotated using the *Drosophila* transcriptome produced the expected band sizes (from left to right) for *engrailed, escargot, and evenskipped.* (TIFF 216 KB)

Additional file 3: **KEGG classification and functional maps of assembled contigs.** Contigs annotated using KEGG Automatic Annotation Server identified sequences in a broad range of functional groups including developmental pathways and cell signaling. (ZIP 11 MB)

Additional file 4: **Average distribution of coverage of** ***T. biloba*** **contigs.** Coverage estimates were generated using the Velvet software. (JPEG 33 KB)
