# Supplementary material for: A pipeline for the de novo assembly of the Themira biloba (Sepsidae: Diptera) transcriptome using a multiple k-mer length approach
Source: BMC Genomics. 2014 Mar 12;15(1):188. doi: 10.1186/1471-2164-15-188 (PMC4008362; doi:10.1186/1471-2164-15-188)
Supplement: Supplementary file 1 — Additional file 1: FastQC reports for untrimmed and trimmed sequence reads. Quality reports generated before and after quality filtering and trimming show an improvement in multiple quality metrics. (ZIP 2 MB) [file 12864_2013_7026_MOESM1_ESM.zip › FastQC/sep1-1-upper_fastqc/sep1-1-upper_fastqc/fastqc_report.html]

sep1-1-upper.fastq FastQC Report


FastQC Report

Fri 10 May 2013  
sep1-1-upper.fastq

## Summary

- Basic Statistics
- Per base sequence quality
- Per sequence quality scores
- Per base sequence content
- Per base GC content
- Per sequence GC content
- Per base N content
- Sequence Length Distribution
- Sequence Duplication Levels
- Overrepresented sequences
- Kmer Content

## Basic Statistics

| Measure | Value |
| --- | --- |
| Filename | sep1-1-upper.fastq |
| File type | Conventional base calls |
| Encoding | Sanger / Illumina 1.9 |
| Total Sequences | 753407 |
| Filtered Sequences | 0 |
| Sequence length | 50-1201 |
| %GC | 45 |

## Per base sequence quality

## Per sequence quality scores

## Per base sequence content

## Per base GC content

## Per sequence GC content

## Per base N content

## Sequence Length Distribution

## Sequence Duplication Levels

## Overrepresented sequences

| Sequence | Count | Percentage | Possible Source |
| --- | --- | --- | --- |
| GACTACACGACGACTCTTTCGTACTAAAATATTATATATTATTAAAGATA | 2079 | 0.2759464671817491 | No Hit |
| GACTACACGACGACTGTCCTTTCGTACTAAAATATTATATATTATTAAAG | 1839 | 0.2440911751549959 | No Hit |
| GACTACACGACGACTTTTCGTACTAAAATATTATATATTATTAAAGATAG | 1026 | 0.13618137341436964 | No Hit |

## Kmer Content

| Sequence | Count | Obs/Exp Overall | Obs/Exp Max | Max Obs/Exp Position |
| --- | --- | --- | --- | --- |
| TTTTT | 2568130 | 4.461569 | 30.35985 | 1100-1196 |
| AAAAA | 2266860 | 3.997711 | 32.33853 | 1100-1196 |
| CGGCG | 857165 | 3.4879947 | 7.0572214 | 500-599 |
| ACTAC | 1300375 | 3.2879417 | 976.22565 | 2 |
| CGACG | 825770 | 2.870249 | 269.0451 | 8 |
| ACACG | 930530 | 2.7627397 | 883.72345 | 5 |
| TCGGC | 790370 | 2.738973 | 5.7304454 | 500-599 |
| TACAC | 1063775 | 2.6897087 | 973.3377 | 4 |
| CTACA | 1033020 | 2.6119463 | 974.9806 | 3 |
| ACGAC | 858975 | 2.5502934 | 229.98888 | 7 |
| GTCGG | 742315 | 2.5113683 | 7.8084927 | 500-599 |
| GACTA | 999210 | 2.4664772 | 955.9699 | 1 |
| GTGGT | 867695 | 2.4406195 | 7.414186 | 500-599 |
| CGTCG | 693740 | 2.404108 | 5.538212 | 10-14 |
| ACGTA | 971525 | 2.3981392 | 542.0791 | 7 |
| TGTTG | 985305 | 2.360204 | 7.5852666 | 15-19 |
| GACGA | 805035 | 2.3334002 | 224.3941 | 9 |
| GGTCG | 687620 | 2.3263266 | 7.932662 | 500-599 |
| CACAC | 740505 | 2.2520225 | 5.0992827 | 500-599 |
| TTGTT | 1085555 | 2.2145047 | 5.252718 | 20-24 |
| TCGTC | 714680 | 2.1091857 | 14.75052 | 10-14 |
| GTAGT | 846460 | 2.0337074 | 527.62366 | 9 |
| TTTTA | 1163560 | 2.0275083 | 12.417739 | 1100-1196 |
| CACGT | 683255 | 2.0225031 | 649.8816 | 6 |
| GGCAC | 570605 | 1.9833349 | 5.2215543 | 500-599 |
| GGCGT | 584380 | 1.9770494 | 5.510282 | 500-599 |
| TAAAA | 1117625 | 1.9650774 | 16.996181 | 1100-1196 |
| CGTAG | 674960 | 1.9505152 | 634.4036 | 8 |
| GCGTC | 544815 | 1.8880188 | 5.464074 | 500-599 |
| TTAAA | 1075090 | 1.8846257 | 17.2396 | 1100-1196 |
| TTTAA | 1075345 | 1.8794245 | 15.784627 | 1100-1196 |
| GTACG | 604340 | 1.7464356 | 11.438657 | 700-799 |
| TAGTA | 846690 | 1.7376235 | 90.294785 | 10-14 |
| CGTAC | 581955 | 1.722645 | 10.507435 | 700-799 |
| CTCGT | 567755 | 1.6755761 | 52.063614 | 10-14 |
| CACGA | 558200 | 1.6572934 | 231.81224 | 6 |
| AAAAC | 723340 | 1.5297314 | 18.579098 | 1100-1196 |
| GTTTT | 727100 | 1.4832655 | 11.671817 | 1100-1196 |
| TCGTG | 505455 | 1.4562991 | 24.879633 | 10-14 |
| AGTAT | 705895 | 1.448676 | 90.85357 | 10-14 |
| AAACG | 555585 | 1.3755428 | 16.644669 | 1100-1196 |
| CGTGG | 404740 | 1.369299 | 6.9839077 | 10-14 |
| CGACT | 461030 | 1.3646948 | 46.604076 | 10-14 |
| CCGGT | 391085 | 1.3552781 | 12.39906 | 900-999 |
| AACGG | 465380 | 1.3489075 | 14.724965 | 1100-1196 |
| ACCGG | 387220 | 1.345917 | 13.525559 | 1100-1196 |
| CGTTT | 539415 | 1.3235396 | 10.415911 | 1100-1196 |
| TGTTT | 646795 | 1.3194454 | 8.519735 | 15-19 |
| ATGGC | 451075 | 1.3035271 | 5.227832 | 10-14 |
| TACGT | 526985 | 1.2969267 | 9.373791 | 700-799 |
| CCGTT | 434390 | 1.2819852 | 9.229321 | 1100-1196 |
| ACTCG | 424650 | 1.2570065 | 54.341915 | 10-14 |
| GTATG | 519515 | 1.2481885 | 50.615807 | 10-14 |
| AACGT | 490225 | 1.2100848 | 10.415884 | 1100-1196 |
| ACGGT | 413280 | 1.1943061 | 9.574727 | 800-899 |
| ATGCC | 395575 | 1.1709416 | 5.0313478 | 10-14 |
| ACGTT | 472080 | 1.1618038 | 7.5742426 | 900-999 |
| GGGGA | 348345 | 1.1539843 | 5.1168556 | 500-599 |
| AAACC | 453045 | 1.1489471 | 17.593578 | 1100-1196 |
| ACCGT | 387620 | 1.1473941 | 8.302333 | 800-899 |
| AACCG | 384875 | 1.1426922 | 13.294256 | 1100-1196 |
| CGGTT | 389345 | 1.1217669 | 11.171383 | 800-899 |
| CTACT | 439085 | 1.1068809 | 218.27786 | 8 |
| ACACT | 431885 | 1.0920024 | 219.23518 | 5 |
| AGGGG | 327220 | 1.084002 | 5.8863235 | 1100-1196 |
| GGTTT | 450470 | 1.0790579 | 14.53532 | 1100-1196 |
| CGTGC | 306000 | 1.060422 | 6.3477674 | 10-14 |
| CCTCC | 291250 | 1.0589937 | 6.168442 | 1100-1196 |
| CGTGT | 352240 | 1.0148615 | 11.029055 | 10-14 |
| TCCGG | 285310 | 0.98872215 | 5.7284408 | 900-999 |
| GTATT | 482680 | 0.98761404 | 9.252707 | 10-14 |
| CGTAA | 396325 | 0.9782996 | 8.557532 | 1100-1196 |
| TCGTA | 397185 | 0.9774848 | 6.4395924 | 10-14 |
| CGGTA | 338195 | 0.9773237 | 9.01963 | 900-999 |
| GGGGG | 251975 | 0.9772342 | 33.077778 | 1100-1196 |
| ATGTT | 470890 | 0.9634906 | 7.947473 | 10-14 |
| TACGG | 332720 | 0.961502 | 8.202521 | 900-999 |
| TTACG | 383745 | 0.94440836 | 7.146996 | 800-899 |
| TTTAC | 447660 | 0.9382344 | 7.3505726 | 1100-1196 |
| TACCG | 315915 | 0.93514 | 8.608085 | 800-899 |
| GTAAA | 453330 | 0.9331444 | 10.311932 | 1100-1196 |
| ATGTC | 376130 | 0.9256679 | 5.354482 | 10-14 |
| CCGTA | 309865 | 0.9172313 | 6.808853 | 800-899 |
| TACTC | 362090 | 0.9127857 | 218.29736 | 9 |
| CCGGG | 222545 | 0.905585 | 23.830698 | 1100-1196 |
| CACTA | 351855 | 0.88965017 | 219.11786 | 6 |
| CCCGG | 212915 | 0.8874681 | 19.674974 | 1100-1196 |
| GGTAC | 301900 | 0.87243766 | 6.63129 | 700-799 |
| TAAAC | 413125 | 0.87106586 | 9.717209 | 1100-1196 |
| TAACG | 338900 | 0.8365502 | 9.522012 | 800-899 |
| GTACC | 281065 | 0.83198047 | 5.843243 | 700-799 |
| GTTTA | 401690 | 0.82189995 | 9.528382 | 1100-1196 |
| TATGT | 399550 | 0.81752133 | 17.975721 | 10-14 |
| ACGGG | 239335 | 0.8121413 | 13.131879 | 1000-1099 |
| GTAAC | 328230 | 0.8102119 | 7.7470527 | 800-899 |
| CGGGT | 236430 | 0.7998798 | 12.017771 | 1000-1099 |
| CCCGT | 223365 | 0.79288006 | 8.790073 | 1100-1196 |
| ACCCG | 221825 | 0.78977996 | 11.793576 | 1100-1196 |
| GTTAC | 315710 | 0.7769723 | 7.567409 | 800-899 |
| TTAAC | 368750 | 0.77517235 | 7.795719 | 900-999 |
| GTATC | 313775 | 0.7722102 | 31.986017 | 10-14 |
| GACTG | 265995 | 0.7686785 | 21.303265 | 10-14 |
| CGTTA | 310790 | 0.764864 | 7.5087466 | 800-899 |
| GTTAA | 369885 | 0.7590981 | 8.31419 | 1100-1196 |
| GGTCC | 217010 | 0.75203323 | 5.10764 | 800-899 |
| TATCG | 305095 | 0.7508484 | 9.593394 | 10-14 |
| CGGGG | 188285 | 0.7479834 | 22.285332 | 1100-1196 |
| GGTTC | 255840 | 0.7371172 | 5.733961 | 900-999 |
| ACTGT | 298120 | 0.73368275 | 8.991823 | 10-14 |
| CCCCG | 171055 | 0.730327 | 14.144596 | 1100-1196 |
| AAAGG | 300080 | 0.72531307 | 6.184503 | 1100-1196 |
| GACTC | 239950 | 0.71027595 | 12.077677 | 10-14 |
| CCCCC | 157045 | 0.6868166 | 24.336708 | 1100-1196 |
| TATGC | 277555 | 0.6830716 | 12.2953825 | 10-14 |
| GGGTT | 238380 | 0.6705062 | 17.663235 | 1100-1196 |
| TATGG | 277230 | 0.66607374 | 11.5884075 | 10-14 |
| GGTAA | 275615 | 0.6641836 | 9.591516 | 1100-1196 |
| TTACC | 262865 | 0.66265124 | 8.893435 | 1100-1196 |
| AAGGG | 231830 | 0.6560079 | 7.2856054 | 1100-1196 |
| GGGGT | 195060 | 0.64425135 | 17.525904 | 1100-1196 |
| AACCC | 211825 | 0.6442018 | 16.323729 | 1100-1196 |
| TATCA | 303150 | 0.63727057 | 5.0138206 | 10-14 |
| GTATA | 309565 | 0.6353061 | 13.913891 | 10-14 |
| ACCCC | 168790 | 0.61556995 | 13.033954 | 1100-1196 |
| GGGTA | 206605 | 0.58287716 | 10.088577 | 1100-1196 |
| TACCC | 186245 | 0.5647108 | 7.089381 | 1100-1196 |
| TAACC | 208610 | 0.52746135 | 7.9566813 | 1100-1196 |
| GGTTA | 218995 | 0.5261581 | 8.950935 | 1000-1099 |
| TATCC | 197520 | 0.49792436 | 6.623495 | 10-14 |
| TATCT | 211385 | 0.44303417 | 8.022935 | 10-14 |

Produced by FastQC (version 0.10.1)
