# Supplementary material for: A pipeline for the de novo assembly of the Themira biloba (Sepsidae: Diptera) transcriptome using a multiple k-mer length approach
Source: BMC Genomics. 2014 Mar 12;15(1):188. doi: 10.1186/1471-2164-15-188 (PMC4008362; doi:10.1186/1471-2164-15-188)
Supplement: Supplementary file 1 — Additional file 1: FastQC reports for untrimmed and trimmed sequence reads. Quality reports generated before and after quality filtering and trimming show an improvement in multiple quality metrics. (ZIP 2 MB) [file 12864_2013_7026_MOESM1_ESM.zip › FastQC/sep1-filtered_fastqc/sep1-filtered_fastqc/fastqc_report.html]

sep1-filtered.fastq FastQC Report


FastQC Report

Fri 10 May 2013  
sep1-filtered.fastq

## Summary

- Basic Statistics
- Per base sequence quality
- Per sequence quality scores
- Per base sequence content
- Per base GC content
- Per sequence GC content
- Per base N content
- Sequence Length Distribution
- Sequence Duplication Levels
- Overrepresented sequences
- Kmer Content

## Basic Statistics

| Measure | Value |
| --- | --- |
| Filename | sep1-filtered.fastq |
| File type | Conventional base calls |
| Encoding | Sanger / Illumina 1.9 |
| Total Sequences | 597520 |
| Filtered Sequences | 0 |
| Sequence length | 75-798 |
| %GC | 47 |

## Per base sequence quality

## Per sequence quality scores

## Per base sequence content

## Per base GC content

## Per sequence GC content

## Per base N content

## Sequence Length Distribution

## Sequence Duplication Levels

## Overrepresented sequences

| Sequence | Count | Percentage | Possible Source |
| --- | --- | --- | --- |
| CTTTCGTACTAAAATATTATATATTATTAAAGATAGAAACCAACCTGGCT | 2276 | 0.38090775204177263 | No Hit |
| GTCCTTTCGTACTAAAATATTATATATTATTAAAGATAGAAACCAACCTG | 1804 | 0.30191458026509577 | No Hit |
| TTTCGTACTAAAATATTATATATTATTAAAGATAGAAACCAACCTGGCTT | 1195 | 0.19999330566340873 | No Hit |
| TTCGTACTAAAATATTATATATTATTAAAGATAGAAACCAACCTGGCTTA | 637 | 0.10660731021555764 | No Hit |
| ATTATATTCATTTATAAAAATTAATCATTCTAATATTTGGTCCTTTCGTA | 611 | 0.10225599143124915 | No Hit |

## Kmer Content

| Sequence | Count | Obs/Exp Overall | Obs/Exp Max | Max Obs/Exp Position |
| --- | --- | --- | --- | --- |
| CGGCG | 711535 | 3.2506597 | 16.633493 | 600-649 |
| AAATT | 941775 | 2.7571588 | 7.254647 | 650-685 |
| AATTT | 946330 | 2.7404845 | 8.937224 | 650-685 |
| TCGGC | 652225 | 2.730813 | 14.881179 | 600-649 |
| TGTTG | 779105 | 2.6283445 | 5.9303856 | 6 |
| AAAAA | 866840 | 2.5936623 | 15.247357 | 650-685 |
| GTGGT | 668735 | 2.4616203 | 22.443323 | 600-649 |
| TTTTT | 863200 | 2.4458866 | 10.508869 | 2 |
| TTGTT | 761935 | 2.355719 | 8.051364 | 2 |
| ATTTT | 799000 | 2.288767 | 5.2380476 | 1 |
| TGGTG | 621065 | 2.2861464 | 9.147515 | 600-649 |
| CACAC | 566055 | 2.2665 | 16.024502 | 600-649 |
| GTGTG | 596705 | 2.196477 | 13.880823 | 600-649 |
| GCACA | 549200 | 2.153794 | 16.347872 | 550-599 |
| TGTGG | 582810 | 2.1453295 | 13.614908 | 600-649 |
| ACACA | 575235 | 2.0901053 | 13.491609 | 600-649 |
| GTCGG | 505995 | 2.0749962 | 15.108418 | 600-649 |
| GGTGT | 562320 | 2.0699053 | 12.817159 | 600-649 |
| GGCAC | 483625 | 2.0470722 | 20.977612 | 650-685 |
| GGCGT | 480785 | 1.9716145 | 15.404661 | 600-649 |
| GTTGT | 574510 | 1.9381347 | 10.04611 | 1 |
| CAAGG | 491445 | 1.887666 | 15.26127 | 600-649 |
| GCGTC | 446885 | 1.8710712 | 15.9095545 | 600-649 |
| GGTCG | 452815 | 1.8569144 | 15.04917 | 600-649 |
| GCCAA | 472855 | 1.8543923 | 5.631834 | 1 |
| ACAAA | 553405 | 1.8247013 | 5.9741526 | 650-685 |
| AATTG | 572620 | 1.8093889 | 5.6140337 | 5 |
| GACGA | 467615 | 1.7961338 | 6.7001367 | 650-685 |
| AATAA | 605560 | 1.7922632 | 7.48826 | 650-685 |
| TCGCC | 415460 | 1.7760171 | 5.16362 | 650-685 |
| TAAAA | 599435 | 1.7741351 | 23.943943 | 650-685 |
| TGGTC | 471725 | 1.7728798 | 14.280844 | 600-649 |
| TTTTG | 571980 | 1.768424 | 6.7215805 | 2 |
| ATAAA | 596305 | 1.7648714 | 5.162713 | 650-685 |
| TTATT | 612275 | 1.7538857 | 5.5044527 | 600-649 |
| TTTGT | 567260 | 1.7538309 | 6.1906023 | 5 |
| TTTTA | 612250 | 1.7538141 | 8.6503 | 650-685 |
| TAAAT | 586405 | 1.7167709 | 11.293866 | 650-685 |
| GGTGG | 424180 | 1.7037185 | 7.9436226 | 650-685 |
| ATTTA | 587720 | 1.701983 | 10.772018 | 650-685 |
| TTAAA | 579960 | 1.6979022 | 24.12303 | 650-685 |
| TTTAA | 584505 | 1.6926727 | 20.961016 | 650-685 |
| TCAAG | 471120 | 1.6584451 | 13.129235 | 550-599 |
| AAGGC | 429655 | 1.6503274 | 15.982712 | 600-649 |
| TTGAA | 503530 | 1.5910753 | 5.8741384 | 7 |
| CTCAA | 441355 | 1.5862843 | 14.1517105 | 550-599 |
| GTCGT | 416065 | 1.5636934 | 11.926319 | 650-685 |
| CGTCT | 400580 | 1.5371034 | 14.358906 | 600-649 |
| AATTC | 472605 | 1.5247098 | 16.179316 | 650-685 |
| GTTGG | 413600 | 1.5224658 | 8.426767 | 1 |
| TGCCA | 384215 | 1.4904522 | 13.533408 | 650-685 |
| GCCCA | 342730 | 1.4811532 | 7.4611983 | 650-685 |
| CACAG | 375575 | 1.47289 | 15.2336235 | 550-599 |
| TTAAT | 501180 | 1.451371 | 14.522988 | 650-685 |
| ATTAA | 493165 | 1.4437994 | 5.2449827 | 600-649 |
| TTTCG | 410140 | 1.4126737 | 7.5367393 | 2 |
| ATAAT | 474395 | 1.3888482 | 5.106791 | 650-685 |
| ATTAT | 475100 | 1.3758459 | 7.3269134 | 650-685 |
| ACGAG | 356970 | 1.3711405 | 5.8520184 | 650-685 |
| ACGAC | 348060 | 1.3649846 | 5.3464527 | 650-685 |
| GTCGA | 359000 | 1.3640013 | 5.4919987 | 1 |
| GTTTG | 402710 | 1.3585596 | 8.4602995 | 1 |
| GTTCG | 359825 | 1.3523271 | 5.8211775 | 1 |
| TGCCC | 313955 | 1.3421013 | 10.067435 | 650-685 |
| CTCTC | 342290 | 1.3410083 | 15.735834 | 600-649 |
| GTCTC | 348985 | 1.3391234 | 14.525225 | 600-649 |
| AGGTG | 357140 | 1.329032 | 9.221436 | 650-685 |
| AAACA | 401670 | 1.3243966 | 5.4257436 | 650-685 |
| ATTGA | 418905 | 1.3236737 | 5.0943003 | 6 |
| TTCTC | 371920 | 1.3079244 | 17.636175 | 650-685 |
| TCTCA | 366115 | 1.3016092 | 14.125488 | 600-649 |
| GAAAA | 399435 | 1.2899456 | 16.195581 | 650-685 |
| AAAAC | 388320 | 1.2803786 | 7.3491554 | 650-685 |
| TCGTG | 339230 | 1.2749251 | 6.9899297 | 650-685 |
| TCGTT | 368300 | 1.2685612 | 5.5129104 | 2 |
| GTTTT | 409335 | 1.2655652 | 14.475379 | 1 |
| CGAGG | 304610 | 1.2628307 | 6.293447 | 650-685 |
| CGTGG | 303775 | 1.2457277 | 7.820828 | 600-649 |
| ATTCG | 356280 | 1.240598 | 8.81 | 650-685 |
| AGGCA | 322935 | 1.2404103 | 17.28721 | 650-685 |
| CTTTT | 388430 | 1.2261449 | 5.030298 | 1 |
| AAAAG | 379615 | 1.2259382 | 19.504354 | 650-685 |
| TAATA | 418220 | 1.224389 | 7.9824595 | 650-685 |
| AACAG | 342420 | 1.2185926 | 17.847225 | 650-685 |
| TTCGT | 352620 | 1.2145536 | 6.192502 | 3 |
| TCTCT | 345050 | 1.2134311 | 14.632998 | 600-649 |
| TATTA | 413670 | 1.1979502 | 7.8959947 | 650-685 |
| TCTTT | 376785 | 1.1893854 | 6.38933 | 650-685 |
| GTTGA | 342225 | 1.1671535 | 5.923736 | 1 |
| GCCAG | 272735 | 1.1544237 | 10.709291 | 650-685 |
| AAAGT | 359180 | 1.1473805 | 16.84748 | 650-685 |
| TATAA | 381280 | 1.1162428 | 7.407147 | 650-685 |
| GTTTC | 323950 | 1.1158036 | 6.4116526 | 1 |
| AGTCG | 288660 | 1.0967482 | 15.028696 | 650-685 |
| TCCTT | 305265 | 1.07352 | 5.190592 | 2 |
| GGGAT | 284820 | 1.0599061 | 16.774872 | 600-649 |
| ATATA | 361175 | 1.057383 | 7.720125 | 650-685 |
| ACAGG | 274510 | 1.0544074 | 15.483253 | 600-649 |
| ACACG | 266615 | 1.0455823 | 8.078504 | 650-685 |
| CAGGG | 248855 | 1.0316856 | 15.812941 | 600-649 |
| GCCTT | 268530 | 1.0304018 | 6.693407 | 650-685 |
| TATAT | 353840 | 1.0246881 | 7.953912 | 650-685 |
| GGGGA | 250045 | 1.0153031 | 18.890402 | 600-649 |
| CTGCC | 236905 | 1.0127264 | 12.90905 | 650-685 |
| CGCCT | 229855 | 0.98258895 | 10.815691 | 650-685 |
| AGGGG | 221720 | 0.90029013 | 17.306425 | 600-649 |
| GGATA | 255990 | 0.8826106 | 12.503384 | 600-649 |
| AAGAC | 247785 | 0.88180894 | 17.847227 | 650-685 |
| ATCTT | 273810 | 0.8737923 | 8.0741205 | 650-685 |
| AAGTC | 247310 | 0.8705851 | 26.169321 | 650-685 |
| CCAGT | 223810 | 0.86820686 | 5.615667 | 650-685 |
| CGTAC | 222830 | 0.8644053 | 5.114396 | 5 |
| TGACA | 233960 | 0.8235902 | 9.50595 | 650-685 |
| AGACG | 211380 | 0.81192166 | 9.718216 | 650-685 |
| GATCT | 228990 | 0.79736316 | 17.462683 | 650-685 |
| TGACC | 203630 | 0.7899243 | 19.807987 | 650-685 |
| ACAGT | 222215 | 0.7822452 | 8.147957 | 650-685 |
| CTTTC | 222250 | 0.7815826 | 6.802491 | 1 |
| CCTGC | 181385 | 0.77538836 | 13.251012 | 650-685 |
| CTTTA | 236810 | 0.75571656 | 8.0741205 | 650-685 |
| CAGTC | 194510 | 0.7545459 | 15.981995 | 650-685 |
| GATAG | 215520 | 0.74307686 | 11.407472 | 600-649 |
| ATAGG | 212600 | 0.73300916 | 11.457284 | 600-649 |
| GTCCT | 190935 | 0.73265475 | 5.87124 | 1 |
| TTTAC | 223625 | 0.7136402 | 5.271927 | 650-685 |
| CTAAA | 216120 | 0.7048776 | 16.356485 | 650-685 |
| GTCTG | 183960 | 0.69137526 | 24.482767 | 650-685 |
| GTACT | 197535 | 0.68783414 | 6.073976 | 650-685 |
| AGTAC | 194015 | 0.6829752 | 8.906477 | 650-685 |
| GAGGT | 182905 | 0.6806479 | 6.4912806 | 650-685 |
| TAGGT | 194400 | 0.6629984 | 6.530465 | 650-685 |
| TCAGT | 189550 | 0.6600296 | 8.81 | 650-685 |
| GGGGG | 148415 | 0.65043837 | 8.623437 | 550-599 |
| GACAG | 167770 | 0.6444134 | 9.518136 | 650-685 |
| TAACA | 196895 | 0.6421751 | 8.251921 | 650-685 |
| ACTGA | 182360 | 0.641947 | 8.13678 | 650-685 |
| ACCTG | 142315 | 0.5520703 | 9.903994 | 650-685 |
| CTGAC | 139455 | 0.5409758 | 28.131062 | 650-685 |
| GGGAA | 142640 | 0.5366216 | 6.562363 | 650-685 |
| TCTGA | 154060 | 0.5364504 | 19.127827 | 650-685 |
| GTCAG | 140865 | 0.53520906 | 19.05424 | 650-685 |
| CCGGG | 116630 | 0.53282607 | 22.911127 | 650-685 |
| GACCT | 134795 | 0.5228987 | 12.786847 | 650-685 |
| CCTGA | 133615 | 0.51832116 | 6.766704 | 650-685 |
| TTAAC | 157245 | 0.5073009 | 16.179314 | 650-685 |
| CGGGA | 112145 | 0.4649229 | 10.4890785 | 650-685 |
| AGTCT | 130155 | 0.45321107 | 21.49253 | 650-685 |
| GTAGG | 103435 | 0.38491464 | 9.813109 | 650-685 |

Produced by FastQC (version 0.10.1)
