# Supplementary material for: A pipeline for the de novo assembly of the Themira biloba (Sepsidae: Diptera) transcriptome using a multiple k-mer length approach
Source: BMC Genomics. 2014 Mar 12;15(1):188. doi: 10.1186/1471-2164-15-188 (PMC4008362; doi:10.1186/1471-2164-15-188)
Supplement: Supplementary file 1 — Additional file 1: FastQC reports for untrimmed and trimmed sequence reads. Quality reports generated before and after quality filtering and trimming show an improvement in multiple quality metrics. (ZIP 2 MB) [file 12864_2013_7026_MOESM1_ESM.zip › FastQC/sep2-1-upper_fastqc/sep2-1-upper_fastqc/fastqc_report.html]

sep2-1-upper.fastq FastQC Report


FastQC Report

Fri 10 May 2013  
sep2-1-upper.fastq

## Summary

- Basic Statistics
- Per base sequence quality
- Per sequence quality scores
- Per base sequence content
- Per base GC content
- Per sequence GC content
- Per base N content
- Sequence Length Distribution
- Sequence Duplication Levels
- Overrepresented sequences
- Kmer Content

## Basic Statistics

| Measure | Value |
| --- | --- |
| Filename | sep2-1-upper.fastq |
| File type | Conventional base calls |
| Encoding | Sanger / Illumina 1.9 |
| Total Sequences | 732276 |
| Filtered Sequences | 0 |
| Sequence length | 50-1201 |
| %GC | 45 |

## Per base sequence quality

## Per sequence quality scores

## Per base sequence content

## Per base GC content

## Per sequence GC content

## Per base N content

## Sequence Length Distribution

## Sequence Duplication Levels

## Overrepresented sequences

| Sequence | Count | Percentage | Possible Source |
| --- | --- | --- | --- |
| GACTACACGACGACTCTTTCGTACTAAAATATTATATATTATTAAAGATA | 1946 | 0.2657467949243182 | No Hit |
| GACTACACGACGACTGTCCTTTCGTACTAAAATATTATATATTATTAAAG | 1754 | 0.23952717281462182 | No Hit |
| GACTACACGACGACTTTTCGTACTAAAATATTATATATTATTAAAGATAG | 1029 | 0.14052078724415384 | No Hit |

## Kmer Content

| Sequence | Count | Obs/Exp Overall | Obs/Exp Max | Max Obs/Exp Position |
| --- | --- | --- | --- | --- |
| TTTTT | 2509635 | 4.517348 | 28.643497 | 1100-1196 |
| AAAAA | 2192965 | 3.965736 | 29.172697 | 1000-1099 |
| CGGCG | 830360 | 3.465433 | 6.803343 | 500-599 |
| ACTAC | 1264810 | 3.2898638 | 976.2656 | 2 |
| CGACG | 802445 | 2.861507 | 267.76093 | 8 |
| ACACG | 907795 | 2.766023 | 881.1332 | 5 |
| TCGGC | 769425 | 2.741208 | 5.5815377 | 500-599 |
| TACAC | 1033815 | 2.6890287 | 973.0053 | 4 |
| CTACA | 1006355 | 2.6176033 | 974.944 | 3 |
| ACGAC | 840535 | 2.5610838 | 228.97316 | 7 |
| GTCGG | 725665 | 2.5216875 | 7.679011 | 500-599 |
| GACTA | 976760 | 2.4781058 | 955.14056 | 1 |
| GTGGT | 852920 | 2.4678988 | 7.19264 | 500-599 |
| CGTCG | 668585 | 2.3819485 | 5.6213207 | 10-14 |
| ACGTA | 936370 | 2.3756337 | 541.35754 | 7 |
| TGTTG | 956950 | 2.363703 | 7.9283147 | 15-19 |
| GACGA | 787840 | 2.3414526 | 223.1363 | 9 |
| GGTCG | 673365 | 2.3399448 | 7.90121 | 500-599 |
| CACAC | 724250 | 2.2624404 | 5.027383 | 500-599 |
| TTGTT | 1052430 | 2.2191236 | 5.3457427 | 20-24 |
| TCGTC | 688785 | 2.0948071 | 14.669036 | 10-14 |
| CACGT | 666135 | 2.027806 | 649.3211 | 6 |
| GTAGT | 818870 | 2.0245218 | 527.51886 | 9 |
| TTTTA | 1122100 | 2.021661 | 13.0474 | 1100-1196 |
| GGCAC | 559025 | 1.9934747 | 5.077385 | 500-599 |
| GGCGT | 566245 | 1.9677023 | 5.3809714 | 500-599 |
| TAAAA | 1088050 | 1.9657902 | 13.104757 | 1100-1196 |
| CGTAG | 659000 | 1.9567212 | 633.4098 | 8 |
| GCGTC | 529195 | 1.8853477 | 5.206783 | 500-599 |
| TTAAA | 1042950 | 1.8825561 | 13.226174 | 1100-1196 |
| TTTAA | 1036245 | 1.8687148 | 12.724872 | 1100-1196 |
| TAGTA | 822870 | 1.7383099 | 90.27042 | 10-14 |
| GTACG | 584575 | 1.7357364 | 11.082947 | 700-799 |
| CGTAC | 563285 | 1.7147166 | 10.051316 | 700-799 |
| CTCGT | 550255 | 1.6734949 | 52.23658 | 10-14 |
| CACGA | 544410 | 1.6588002 | 231.08221 | 6 |
| CGTCT | 523445 | 1.5919572 | 14.181283 | 1100-1196 |
| AAAAC | 711845 | 1.5445776 | 14.453779 | 1100-1196 |
| GTTTT | 705030 | 1.486606 | 12.953317 | 1100-1196 |
| AGTAT | 690470 | 1.4586154 | 90.90459 | 10-14 |
| TCGTG | 490900 | 1.4562392 | 24.699049 | 10-14 |
| AAACG | 545800 | 1.3860195 | 12.355899 | 1000-1099 |
| CGTGG | 397135 | 1.3800449 | 7.026262 | 10-14 |
| CCGGT | 384000 | 1.3680657 | 14.199529 | 900-999 |
| CGACT | 448330 | 1.3647778 | 46.519497 | 10-14 |
| AACGG | 458135 | 1.3615726 | 17.817509 | 1000-1099 |
| ACCGG | 380635 | 1.3573388 | 14.9090395 | 900-999 |
| CGTTT | 526950 | 1.3344237 | 10.398305 | 1100-1196 |
| CGAGG | 382290 | 1.3296944 | 8.109313 | 1100-1196 |
| TGTTT | 624845 | 1.3175303 | 8.397554 | 15-19 |
| CCGTT | 429250 | 1.3054814 | 13.930362 | 1100-1196 |
| ATGGC | 436295 | 1.2954592 | 5.0326214 | 10-14 |
| TACGT | 506900 | 1.2848442 | 9.177864 | 700-799 |
| ACTCG | 414985 | 1.2632712 | 54.338963 | 10-14 |
| GTATG | 501065 | 1.2388012 | 50.57843 | 10-14 |
| AACGT | 477555 | 1.2115891 | 9.043825 | 800-899 |
| ACGGT | 406795 | 1.207867 | 12.985233 | 1000-1099 |
| ACGTT | 464610 | 1.1776514 | 7.8797264 | 800-899 |
| ACCGT | 385495 | 1.1734995 | 9.893807 | 1100-1196 |
| GGGGA | 342630 | 1.1624215 | 5.038647 | 500-599 |
| AACCG | 379860 | 1.1574215 | 14.373684 | 1100-1196 |
| AAACC | 443675 | 1.1551049 | 15.030231 | 1100-1196 |
| CGGTT | 380770 | 1.1295421 | 14.175975 | 1000-1099 |
| CTACT | 423585 | 1.1007518 | 218.64955 | 8 |
| AGGGG | 323670 | 1.0980968 | 5.3001885 | 500-599 |
| ACACT | 420305 | 1.0932442 | 219.42244 | 5 |
| GGTTT | 437850 | 1.0815064 | 16.087961 | 1100-1196 |
| CGTGC | 293980 | 1.047354 | 6.216714 | 10-14 |
| CGTGT | 340990 | 1.011536 | 10.868378 | 10-14 |
| GTATT | 478780 | 1.010481 | 9.364288 | 10-14 |
| CGGTA | 334760 | 0.99397874 | 10.147401 | 1000-1099 |
| GGGGG | 249615 | 0.9911067 | 27.62436 | 1100-1196 |
| TCGTA | 389370 | 0.9869398 | 6.540965 | 10-14 |
| TCCGG | 276770 | 0.98604053 | 5.729664 | 800-899 |
| CGTAA | 387960 | 0.9842806 | 7.5490365 | 700-799 |
| TACGG | 331050 | 0.982963 | 9.003601 | 900-999 |
| ATGTT | 459555 | 0.96990603 | 8.177695 | 10-14 |
| TACCG | 316735 | 0.96418476 | 9.0559225 | 900-999 |
| TTACG | 379005 | 0.96066755 | 8.238108 | 1100-1196 |
| GTAAA | 448955 | 0.9492981 | 9.859502 | 1100-1196 |
| TTTAC | 437775 | 0.9472481 | 8.744199 | 1100-1196 |
| CCGTA | 310485 | 0.9451589 | 7.0912404 | 800-899 |
| ATGTC | 361530 | 0.9163735 | 5.1629567 | 10-14 |
| TACTC | 351715 | 0.9139863 | 218.58925 | 9 |
| CCGGG | 216495 | 0.90352243 | 21.309446 | 1100-1196 |
| GGTAC | 300905 | 0.89345545 | 7.4347906 | 900-999 |
| CCCGG | 208290 | 0.89121014 | 19.598091 | 1100-1196 |
| CACTA | 342440 | 0.8907116 | 219.26144 | 6 |
| TAAAC | 405775 | 0.87964153 | 8.664998 | 900-999 |
| GTACC | 281105 | 0.85572207 | 6.8768263 | 1000-1099 |
| TAACG | 326250 | 0.8277183 | 9.306727 | 800-899 |
| GTTTA | 391430 | 0.8261259 | 9.719256 | 1100-1196 |
| ACGGG | 236775 | 0.82355905 | 13.700206 | 1000-1099 |
| TATGT | 389230 | 0.8214828 | 18.163586 | 10-14 |
| CGGGT | 234650 | 0.8154093 | 14.391975 | 1000-1099 |
| CCCGT | 222320 | 0.81203526 | 9.8169 | 1100-1196 |
| GTAAC | 318660 | 0.80846184 | 7.388657 | 700-799 |
| ACCCG | 220950 | 0.80778205 | 10.58838 | 1000-1099 |
| GTTAC | 316410 | 0.8020073 | 8.48573 | 1100-1196 |
| CGTTA | 311080 | 0.7884974 | 7.974283 | 800-899 |
| TTAAC | 360505 | 0.7807786 | 9.534315 | 1000-1099 |
| GTATC | 304505 | 0.7718317 | 31.899462 | 10-14 |
| GACTG | 259520 | 0.77057403 | 21.29445 | 10-14 |
| GTTAA | 361415 | 0.7634878 | 9.544426 | 1000-1099 |
| TATCG | 296310 | 0.7510598 | 9.5702305 | 10-14 |
| GGTCC | 210295 | 0.7492119 | 5.035607 | 800-899 |
| GAGGG | 220525 | 0.7481627 | 5.2731743 | 1100-1196 |
| GGTTC | 250960 | 0.7444648 | 5.656093 | 900-999 |
| CGGGG | 182195 | 0.74166346 | 19.35083 | 1100-1196 |
| ACTGT | 288310 | 0.7307821 | 8.851156 | 10-14 |
| CCCCG | 166110 | 0.7286655 | 18.116207 | 1100-1196 |
| GACTC | 236830 | 0.7209429 | 12.1060295 | 10-14 |
| CCCCC | 157665 | 0.7090687 | 24.295162 | 1100-1196 |
| GGAAT | 282280 | 0.69854015 | 5.7694774 | 1100-1196 |
| GGGTT | 233985 | 0.67702866 | 19.916664 | 1100-1196 |
| TTACC | 260330 | 0.6765081 | 12.629558 | 1100-1196 |
| GGTAA | 273260 | 0.6762191 | 11.323944 | 1000-1099 |
| TATGC | 265405 | 0.67272455 | 12.134059 | 10-14 |
| ACTAG | 264675 | 0.67149824 | 5.0700283 | 1000-1099 |
| TATGG | 266675 | 0.6593103 | 11.528138 | 10-14 |
| AACCC | 207320 | 0.6476343 | 14.498627 | 1100-1196 |
| GGGGT | 189035 | 0.6407323 | 17.052238 | 1100-1196 |
| GTATA | 298780 | 0.63117164 | 13.995212 | 10-14 |
| TATCA | 291060 | 0.63037527 | 5.0315084 | 10-14 |
| TTCCC | 201120 | 0.62709916 | 5.16519 | 1100-1196 |
| ACCCC | 163290 | 0.6120409 | 17.564806 | 1100-1196 |
| GGGTA | 204975 | 0.59364086 | 9.310546 | 1000-1099 |
| TACCC | 185955 | 0.5803536 | 8.974092 | 1100-1196 |
| TAACC | 207550 | 0.53985286 | 10.780908 | 1100-1196 |
| GGTTA | 216355 | 0.53490233 | 11.743044 | 1000-1099 |
| TATCC | 191575 | 0.49783754 | 6.5223503 | 10-14 |
| TATCT | 203315 | 0.43992865 | 8.063691 | 10-14 |

Produced by FastQC (version 0.10.1)
