# Supplementary material for: A pipeline for the de novo assembly of the Themira biloba (Sepsidae: Diptera) transcriptome using a multiple k-mer length approach
Source: BMC Genomics. 2014 Mar 12;15(1):188. doi: 10.1186/1471-2164-15-188 (PMC4008362; doi:10.1186/1471-2164-15-188)
Supplement: Supplementary file 1 — Additional file 1: FastQC reports for untrimmed and trimmed sequence reads. Quality reports generated before and after quality filtering and trimming show an improvement in multiple quality metrics. (ZIP 2 MB) [file 12864_2013_7026_MOESM1_ESM.zip › FastQC/sep2-filtered_fastqc/sep2-filtered_fastqc/fastqc_report.html]

sep2-filtered.fastq FastQC Report


FastQC Report

Fri 10 May 2013  
sep2-filtered.fastq

## Summary

- Basic Statistics
- Per base sequence quality
- Per sequence quality scores
- Per base sequence content
- Per base GC content
- Per sequence GC content
- Per base N content
- Sequence Length Distribution
- Sequence Duplication Levels
- Overrepresented sequences
- Kmer Content

## Basic Statistics

| Measure | Value |
| --- | --- |
| Filename | sep2-filtered.fastq |
| File type | Conventional base calls |
| Encoding | Sanger / Illumina 1.9 |
| Total Sequences | 573804 |
| Filtered Sequences | 0 |
| Sequence length | 75-737 |
| %GC | 47 |

## Per base sequence quality

## Per sequence quality scores

## Per base sequence content

## Per base GC content

## Per sequence GC content

## Per base N content

## Sequence Length Distribution

## Sequence Duplication Levels

## Overrepresented sequences

| Sequence | Count | Percentage | Possible Source |
| --- | --- | --- | --- |
| CTTTCGTACTAAAATATTATATATTATTAAAGATAGAAACCAACCTGGCT | 2046 | 0.3565677478720957 | No Hit |
| GTCCTTTCGTACTAAAATATTATATATTATTAAAGATAGAAACCAACCTG | 1669 | 0.2908658705760155 | No Hit |
| TTTCGTACTAAAATATTATATATTATTAAAGATAGAAACCAACCTGGCTT | 1151 | 0.20059114262012812 | No Hit |
| TTCGTACTAAAATATTATATATTATTAAAGATAGAAACCAACCTGGCTTA | 611 | 0.10648235285916445 | No Hit |
| ATTATATTCATTTATAAAAATTAATCATTCTAATATTTGGTCCTTTCGTA | 592 | 0.10317111766387128 | No Hit |

## Kmer Content

| Sequence | Count | Obs/Exp Overall | Obs/Exp Max | Max Obs/Exp Position |
| --- | --- | --- | --- | --- |
| CGGCG | 684100 | 3.2574334 | 18.365875 | 600-649 |
| TCGGC | 631130 | 2.757296 | 16.76978 | 600-649 |
| AAATT | 887390 | 2.7436903 | 9.856686 | 650-678 |
| AATTT | 891645 | 2.7209885 | 8.254471 | 650-678 |
| TGTTG | 746025 | 2.6345057 | 5.9992776 | 7 |
| AAAAA | 809120 | 2.5680592 | 9.694988 | 650-678 |
| GTGGT | 643350 | 2.4761922 | 26.564093 | 650-678 |
| TTTTT | 823445 | 2.4479232 | 16.413874 | 650-678 |
| TTGTT | 730280 | 2.366159 | 7.719729 | 2 |
| AAAAT | 745370 | 2.3349535 | 6.961598 | 650-678 |
| ATTTT | 758185 | 2.2836215 | 5.236467 | 1 |
| TGGTG | 592580 | 2.2807837 | 14.724099 | 650-678 |
| CACAC | 542610 | 2.2785094 | 16.938612 | 550-599 |
| GTGTG | 571250 | 2.1986864 | 13.917592 | 600-649 |
| GCACA | 523385 | 2.1536057 | 17.370386 | 550-599 |
| TGTGG | 556915 | 2.1435125 | 14.48857 | 600-649 |
| GTCGG | 491200 | 2.1028335 | 17.226562 | 600-649 |
| ACACA | 547710 | 2.0950294 | 14.63292 | 650-678 |
| CACCA | 497865 | 2.090618 | 11.358409 | 650-678 |
| GGTGT | 537110 | 2.0672846 | 13.061123 | 600-649 |
| GGCAC | 466420 | 2.0645602 | 31.399527 | 650-678 |
| GGCGT | 458425 | 1.9625231 | 16.909021 | 600-649 |
| GTTGT | 551035 | 1.9459199 | 9.740841 | 1 |
| CAAGG | 470340 | 1.8964384 | 46.27428 | 650-678 |
| GGTCG | 436720 | 1.8696038 | 17.22656 | 600-649 |
| GCGTC | 426190 | 1.8619494 | 15.392551 | 600-649 |
| CAAAT | 541240 | 1.8613149 | 5.292616 | 650-678 |
| GCCAA | 448750 | 1.8465003 | 5.809429 | 1 |
| AATTG | 546025 | 1.8160951 | 5.665924 | 5 |
| TGGTC | 454675 | 1.7858964 | 19.939873 | 650-678 |
| TAAAA | 565650 | 1.7719609 | 39.946316 | 650-678 |
| TTTTG | 542805 | 1.7587266 | 12.394987 | 650-678 |
| TTTGT | 540080 | 1.7498974 | 8.7641325 | 650-678 |
| TTATT | 578695 | 1.7430052 | 5.9088902 | 650-678 |
| ATAAA | 556190 | 1.7423264 | 8.473461 | 650-678 |
| TTTTA | 575905 | 1.734602 | 19.11288 | 650-678 |
| TTTAT | 571565 | 1.7215298 | 6.401298 | 650-678 |
| TAAAT | 547960 | 1.6942185 | 22.377342 | 650-678 |
| TTAAA | 546320 | 1.6891478 | 27.484201 | 650-678 |
| ATTTA | 551610 | 1.6833208 | 7.7459006 | 650-678 |
| TCAAG | 451655 | 1.6708686 | 53.54925 | 650-678 |
| TTTAA | 545980 | 1.66614 | 21.695377 | 650-678 |
| AAGGC | 412100 | 1.6616112 | 43.625584 | 650-678 |
| TTCAA | 488305 | 1.6574305 | 5.32274 | 7 |
| TTCTT | 494445 | 1.6348977 | 12.649234 | 650-678 |
| GGCAA | 404850 | 1.6323787 | 5.1845555 | 1 |
| CTCAA | 423425 | 1.5985641 | 15.68173 | 600-649 |
| CCAAA | 417680 | 1.597655 | 6.783472 | 650-678 |
| TTGAA | 478120 | 1.5902411 | 5.821155 | 7 |
| GTCGT | 399750 | 1.5701591 | 7.6262617 | 1 |
| CGTCT | 386380 | 1.5487738 | 14.791725 | 600-649 |
| GTTGG | 394770 | 1.5194318 | 8.290333 | 1 |
| TGCCA | 365000 | 1.4823543 | 9.624327 | 650-678 |
| CACAG | 355805 | 1.4640535 | 16.304897 | 550-599 |
| GCCCA | 323185 | 1.4598887 | 32.693127 | 650-678 |
| AATTA | 466870 | 1.4434992 | 6.296286 | 650-678 |
| ATTAA | 463390 | 1.4327395 | 9.098479 | 650-678 |
| TTTCG | 392255 | 1.413619 | 7.2240186 | 2 |
| ACCAA | 366550 | 1.4020795 | 8.1294 | 650-678 |
| GTCGA | 342090 | 1.3613863 | 5.209992 | 1 |
| GTTCG | 345820 | 1.35833 | 5.857189 | 1 |
| GTCTC | 336510 | 1.3488739 | 15.813028 | 600-649 |
| GTTTG | 381320 | 1.3465898 | 8.208016 | 1 |
| CTCTC | 328455 | 1.3435917 | 16.707096 | 600-649 |
| AAACA | 383885 | 1.3375713 | 17.688173 | 650-678 |
| TCACC | 321920 | 1.334213 | 15.855094 | 650-678 |
| ATTGA | 399410 | 1.3284494 | 5.0216722 | 6 |
| TCTCA | 353725 | 1.3180552 | 15.685057 | 600-649 |
| AAAAC | 369415 | 1.2871532 | 19.238165 | 650-678 |
| ACATT | 378015 | 1.2830786 | 5.223777 | 650-678 |
| TCGTG | 326110 | 1.280912 | 5.5519977 | 550-599 |
| GTTTT | 388685 | 1.2593669 | 14.177149 | 1 |
| CGTGG | 292615 | 1.2526885 | 6.5425487 | 600-649 |
| AGGCA | 310130 | 1.2504622 | 42.846554 | 650-678 |
| AAAAG | 361480 | 1.2341896 | 16.745367 | 650-678 |
| TCTCT | 332900 | 1.2243224 | 15.482955 | 600-649 |
| TTCGT | 338405 | 1.2195529 | 6.097052 | 6 |
| CTTTT | 368215 | 1.2175143 | 25.443861 | 650-678 |
| TATTA | 392430 | 1.1975591 | 8.662818 | 650-678 |
| TCTTT | 360940 | 1.1934593 | 23.455532 | 650-678 |
| AACAT | 343720 | 1.182047 | 12.224538 | 650-678 |
| GTTGA | 326930 | 1.169732 | 5.878106 | 1 |
| CGTTT | 321095 | 1.1571707 | 5.029017 | 3 |
| AAAGT | 342855 | 1.1553733 | 15.943552 | 650-678 |
| CCGTC | 257745 | 1.14914 | 8.233877 | 650-678 |
| CGGGC | 241045 | 1.1477678 | 7.328183 | 650-678 |
| AGTCG | 281025 | 1.118371 | 5.4928055 | 550-599 |
| TTGTA | 340025 | 1.1162233 | 6.9768496 | 650-678 |
| GTTTC | 309645 | 1.1159068 | 6.500946 | 1 |
| TTATA | 365645 | 1.1158206 | 22.086285 | 650-678 |
| ACTTT | 332405 | 1.1135917 | 11.882317 | 650-678 |
| TATAA | 357480 | 1.10528 | 11.828024 | 650-678 |
| TCCTT | 297170 | 1.0929165 | 5.1922936 | 2 |
| CGTCC | 241535 | 1.076869 | 8.039814 | 650-678 |
| GGGAT | 274370 | 1.0699402 | 18.636011 | 600-649 |
| ACACG | 254980 | 1.0491825 | 5.222183 | 650-678 |
| ACAGG | 259365 | 1.0457748 | 17.138622 | 650-678 |
| AATAC | 300655 | 1.0339473 | 49.779205 | 650-678 |
| GGGGA | 240370 | 1.0216318 | 20.371017 | 600-649 |
| CAGGG | 234445 | 1.0168884 | 23.076305 | 650-678 |
| CTGCC | 223960 | 0.99851185 | 5.6583514 | 650-678 |
| ACCGG | 225145 | 0.9965813 | 7.849882 | 650-678 |
| CCGGT | 227805 | 0.99524003 | 7.4045243 | 650-678 |
| ATTCT | 291815 | 0.9776108 | 24.246347 | 650-678 |
| ATACT | 284780 | 0.9666154 | 25.969633 | 650-678 |
| CACTG | 232995 | 0.94624966 | 8.63134 | 650-678 |
| AGTGG | 238705 | 0.93086004 | 28.223494 | 650-678 |
| CGGTC | 210695 | 0.92048955 | 7.2891893 | 650-678 |
| GTCCG | 207480 | 0.90644383 | 14.398953 | 650-678 |
| AGGGG | 211810 | 0.9002448 | 18.994312 | 600-649 |
| TACTA | 264365 | 0.8973216 | 18.74097 | 650-678 |
| CCCAC | 193840 | 0.893573 | 17.635113 | 650-678 |
| AAGTC | 236700 | 0.87565655 | 17.080366 | 650-678 |
| GGATA | 240260 | 0.87096125 | 12.973795 | 600-649 |
| ATCTT | 258550 | 0.8661696 | 14.239918 | 650-678 |
| CTGAA | 225045 | 0.8325395 | 5.6934547 | 650-678 |
| AACCG | 200315 | 0.82424897 | 8.745085 | 650-678 |
| TCGTA | 221280 | 0.80796367 | 5.03569 | 7 |
| TCGGG | 187615 | 0.8031821 | 7.592052 | 650-678 |
| AGATC | 216810 | 0.8020746 | 14.085229 | 650-678 |
| GATCT | 217975 | 0.7958961 | 19.753048 | 650-678 |
| TGACC | 195600 | 0.7943794 | 23.716019 | 650-678 |
| CTTTC | 214910 | 0.79038495 | 6.436896 | 1 |
| CCACT | 184960 | 0.7665757 | 11.210672 | 650-678 |
| CCTGC | 171880 | 0.7663163 | 7.854685 | 650-678 |
| TACTT | 227885 | 0.76343864 | 14.239918 | 650-678 |
| CTTTA | 224535 | 0.7522158 | 14.239918 | 650-678 |
| ATTAC | 219880 | 0.7463283 | 6.268532 | 650-678 |
| GATAG | 203190 | 0.7365796 | 26.236464 | 650-678 |
| GTCCT | 182545 | 0.7317172 | 5.734123 | 1 |
| TCCGT | 181855 | 0.7289514 | 7.5858893 | 650-678 |
| ATAGG | 200775 | 0.72782505 | 13.867845 | 650-678 |
| TCCGG | 166340 | 0.72671026 | 7.1554427 | 650-678 |
| TTTAC | 214850 | 0.7197699 | 5.9411573 | 650-678 |
| CTCAC | 173420 | 0.71874756 | 29.996122 | 650-678 |
| CTAAA | 206035 | 0.70855075 | 24.395103 | 650-678 |
| TGTAG | 197745 | 0.70751727 | 6.3451843 | 650-678 |
| GGAAC | 175455 | 0.70744467 | 15.424758 | 650-678 |
| GTCTG | 175870 | 0.6907914 | 17.299807 | 650-678 |
| CGGAA | 169655 | 0.68405885 | 6.524068 | 650-678 |
| GGGGG | 146070 | 0.6678527 | 10.607931 | 600-649 |
| AGTGT | 184555 | 0.6603244 | 7.60415 | 650-678 |
| ACTGA | 173850 | 0.643147 | 6.391363 | 650-678 |
| ACTAA | 179210 | 0.6163001 | 21.926554 | 650-678 |
| ACTAG | 166320 | 0.6152902 | 9.390154 | 650-678 |
| ACCTG | 137135 | 0.55693877 | 16.73152 | 650-678 |
| TTACT | 163800 | 0.5487472 | 9.061766 | 650-678 |
| CTGAC | 134015 | 0.54426765 | 24.09127 | 650-678 |
| GAACC | 132055 | 0.5433751 | 11.130106 | 650-678 |
| TCTGA | 145945 | 0.53289163 | 18.924696 | 650-678 |
| GACCT | 128850 | 0.5232913 | 16.835121 | 650-678 |
| AGTAG | 139080 | 0.50417584 | 13.867845 | 650-678 |
| TTAAC | 148270 | 0.50326586 | 6.423584 | 650-678 |
| GAGTA | 135350 | 0.49065432 | 26.236464 | 650-678 |
| AGTTA | 144720 | 0.48134297 | 6.2944717 | 650-678 |
| GGTCC | 108945 | 0.47596157 | 7.244055 | 650-678 |
| GTAGA | 128505 | 0.46584067 | 5.5790186 | 650-678 |
| AGTCT | 124025 | 0.45285475 | 13.168699 | 650-678 |
| GTAGG | 101860 | 0.39721584 | 10.548176 | 650-678 |
| TAGTG | 110090 | 0.39389405 | 9.678008 | 650-678 |
| TAGAT | 109320 | 0.3636015 | 9.182142 | 650-678 |
| CTAGT | 61750 | 0.2254689 | 8.6528845 | 650-678 |

Produced by FastQC (version 0.10.1)
