# Supplementary figures and images for: A pipeline for the de novo assembly of the Themira biloba (Sepsidae: Diptera) transcriptome using a multiple k-mer length approach (part 1 of 2)
Source: BMC Genomics. 2014 Mar 12;15(1):188. doi: 10.1186/1471-2164-15-188 (PMC4008362; doi:10.1186/1471-2164-15-188)

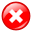

Supplement: Supplementary file 1 — Additional file 1: FastQC reports for untrimmed and trimmed sequence reads. Quality reports generated before and after quality filtering and trimming show an improvement in multiple quality metrics. (ZIP 2 MB) [file 12864_2013_7026_MOESM1_ESM.zip › FastQC/sep1-1-upper_fastqc/sep1-1-upper_fastqc/Icons/error.png]

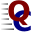

Supplement: Supplementary file 1 — Additional file 1: FastQC reports for untrimmed and trimmed sequence reads. Quality reports generated before and after quality filtering and trimming show an improvement in multiple quality metrics. (ZIP 2 MB) [file 12864_2013_7026_MOESM1_ESM.zip › FastQC/sep1-1-upper_fastqc/sep1-1-upper_fastqc/Icons/fastqc_icon.png]

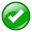

Supplement: Supplementary file 1 — Additional file 1: FastQC reports for untrimmed and trimmed sequence reads. Quality reports generated before and after quality filtering and trimming show an improvement in multiple quality metrics. (ZIP 2 MB) [file 12864_2013_7026_MOESM1_ESM.zip › FastQC/sep1-1-upper_fastqc/sep1-1-upper_fastqc/Icons/tick.png]

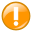

Supplement: Supplementary file 1 — Additional file 1: FastQC reports for untrimmed and trimmed sequence reads. Quality reports generated before and after quality filtering and trimming show an improvement in multiple quality metrics. (ZIP 2 MB) [file 12864_2013_7026_MOESM1_ESM.zip › FastQC/sep1-1-upper_fastqc/sep1-1-upper_fastqc/Icons/warning.png]

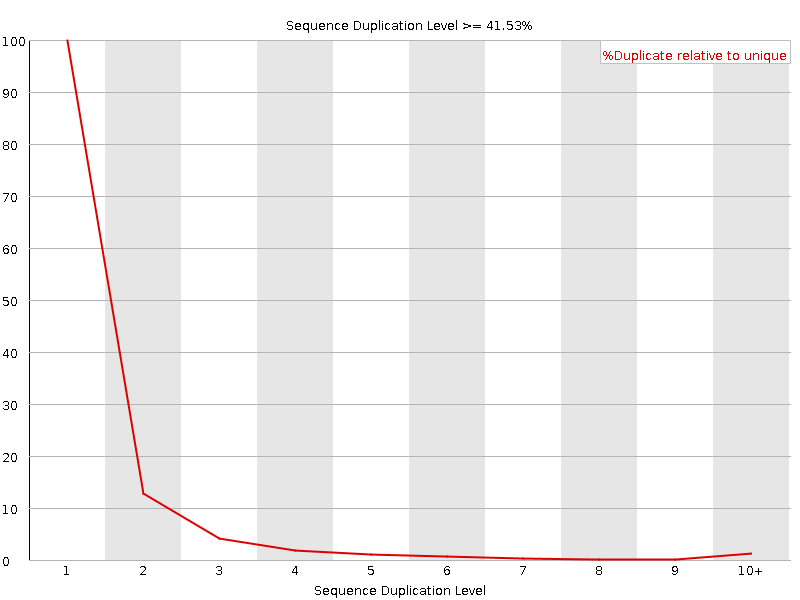

Supplement: Supplementary file 1 — Additional file 1: FastQC reports for untrimmed and trimmed sequence reads. Quality reports generated before and after quality filtering and trimming show an improvement in multiple quality metrics. (ZIP 2 MB) [file 12864_2013_7026_MOESM1_ESM.zip › FastQC/sep1-1-upper_fastqc/sep1-1-upper_fastqc/Images/duplication_levels.png]

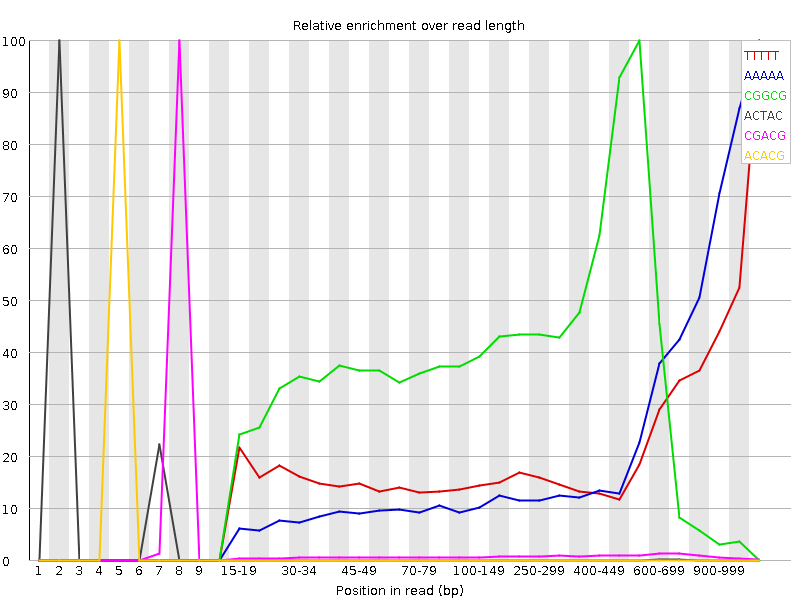

Supplement: Supplementary file 1 — Additional file 1: FastQC reports for untrimmed and trimmed sequence reads. Quality reports generated before and after quality filtering and trimming show an improvement in multiple quality metrics. (ZIP 2 MB) [file 12864_2013_7026_MOESM1_ESM.zip › FastQC/sep1-1-upper_fastqc/sep1-1-upper_fastqc/Images/kmer_profiles.png]

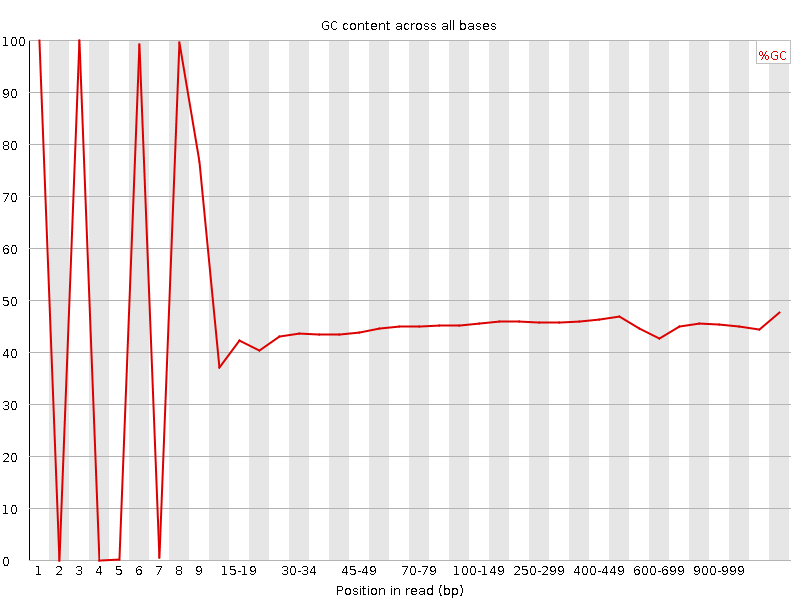

Supplement: Supplementary file 1 — Additional file 1: FastQC reports for untrimmed and trimmed sequence reads. Quality reports generated before and after quality filtering and trimming show an improvement in multiple quality metrics. (ZIP 2 MB) [file 12864_2013_7026_MOESM1_ESM.zip › FastQC/sep1-1-upper_fastqc/sep1-1-upper_fastqc/Images/per_base_gc_content.png]

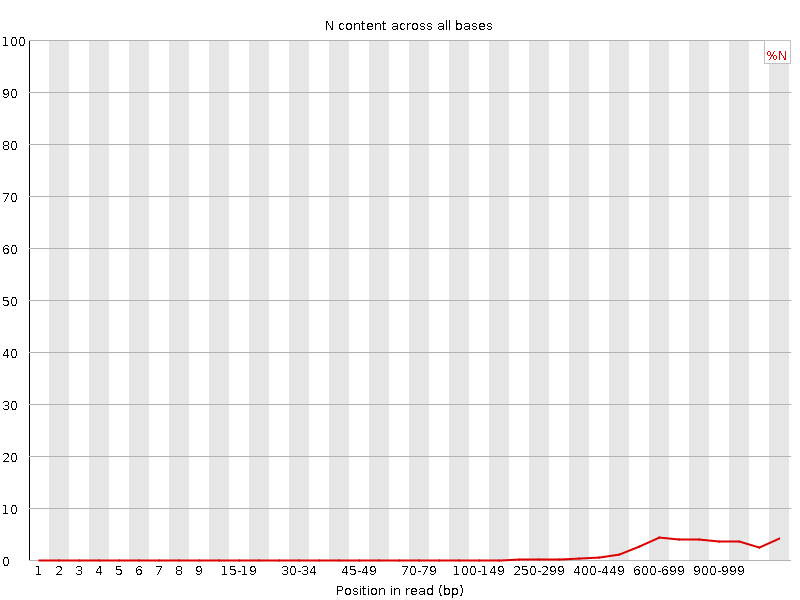

Supplement: Supplementary file 1 — Additional file 1: FastQC reports for untrimmed and trimmed sequence reads. Quality reports generated before and after quality filtering and trimming show an improvement in multiple quality metrics. (ZIP 2 MB) [file 12864_2013_7026_MOESM1_ESM.zip › FastQC/sep1-1-upper_fastqc/sep1-1-upper_fastqc/Images/per_base_n_content.png]

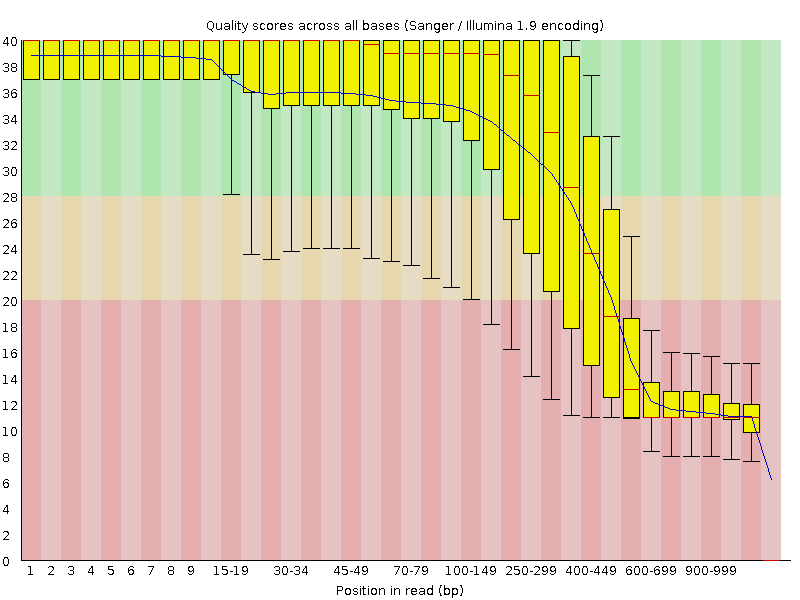

Supplement: Supplementary file 1 — Additional file 1: FastQC reports for untrimmed and trimmed sequence reads. Quality reports generated before and after quality filtering and trimming show an improvement in multiple quality metrics. (ZIP 2 MB) [file 12864_2013_7026_MOESM1_ESM.zip › FastQC/sep1-1-upper_fastqc/sep1-1-upper_fastqc/Images/per_base_quality.png]

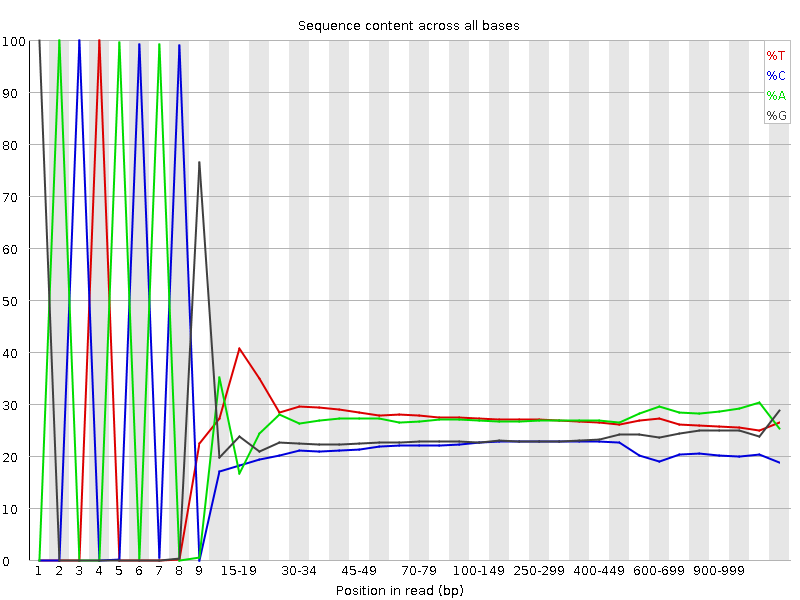

Supplement: Supplementary file 1 — Additional file 1: FastQC reports for untrimmed and trimmed sequence reads. Quality reports generated before and after quality filtering and trimming show an improvement in multiple quality metrics. (ZIP 2 MB) [file 12864_2013_7026_MOESM1_ESM.zip › FastQC/sep1-1-upper_fastqc/sep1-1-upper_fastqc/Images/per_base_sequence_content.png]

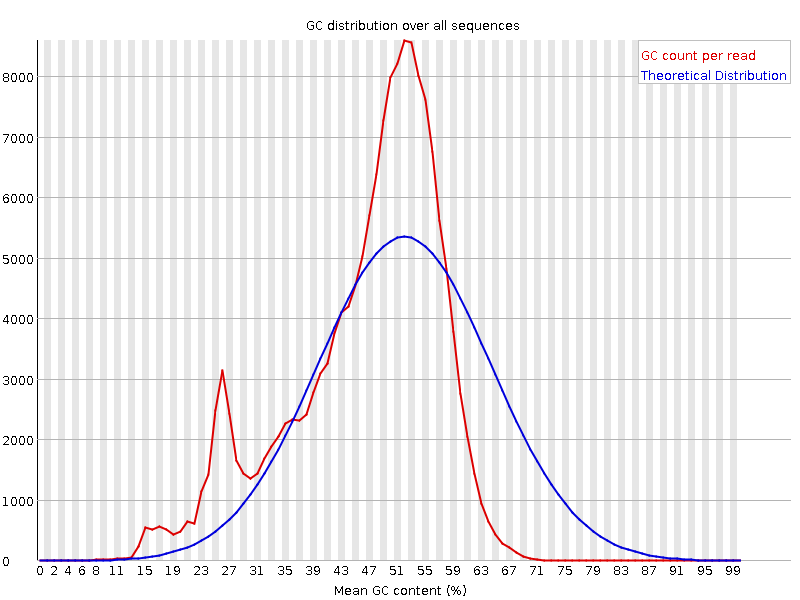

Supplement: Supplementary file 1 — Additional file 1: FastQC reports for untrimmed and trimmed sequence reads. Quality reports generated before and after quality filtering and trimming show an improvement in multiple quality metrics. (ZIP 2 MB) [file 12864_2013_7026_MOESM1_ESM.zip › FastQC/sep1-1-upper_fastqc/sep1-1-upper_fastqc/Images/per_sequence_gc_content.png]

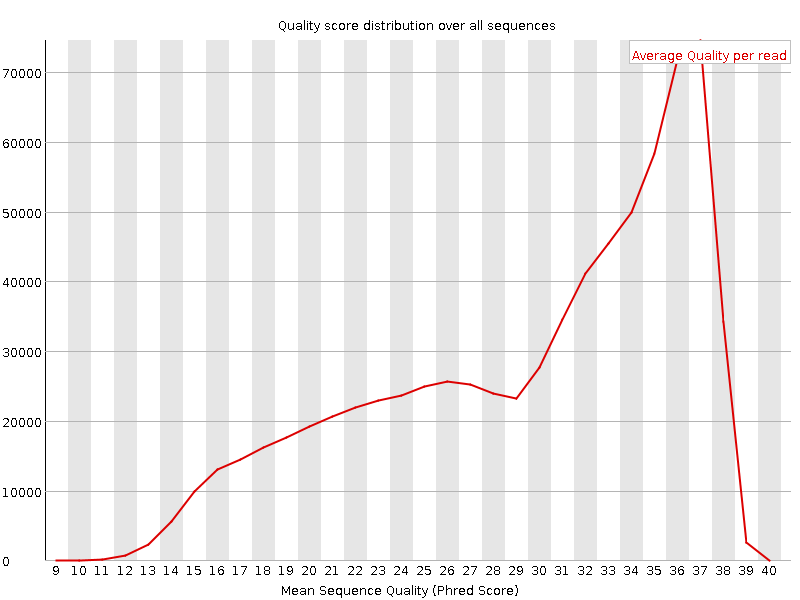

Supplement: Supplementary file 1 — Additional file 1: FastQC reports for untrimmed and trimmed sequence reads. Quality reports generated before and after quality filtering and trimming show an improvement in multiple quality metrics. (ZIP 2 MB) [file 12864_2013_7026_MOESM1_ESM.zip › FastQC/sep1-1-upper_fastqc/sep1-1-upper_fastqc/Images/per_sequence_quality.png]

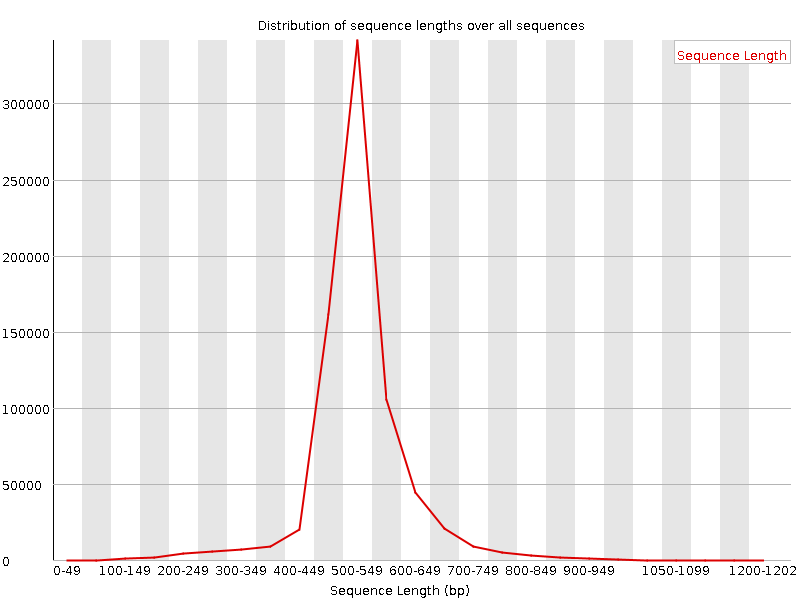

Supplement: Supplementary file 1 — Additional file 1: FastQC reports for untrimmed and trimmed sequence reads. Quality reports generated before and after quality filtering and trimming show an improvement in multiple quality metrics. (ZIP 2 MB) [file 12864_2013_7026_MOESM1_ESM.zip › FastQC/sep1-1-upper_fastqc/sep1-1-upper_fastqc/Images/sequence_length_distribution.png]

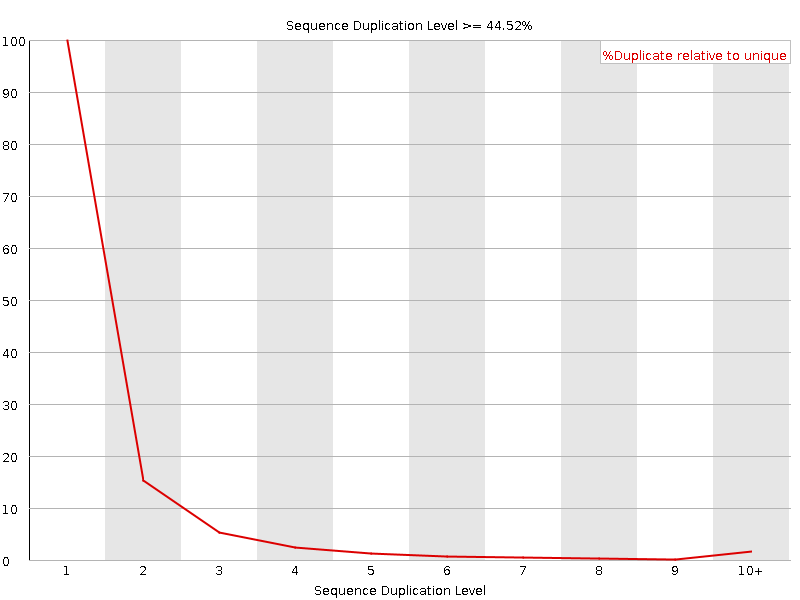

Supplement: Supplementary file 1 — Additional file 1: FastQC reports for untrimmed and trimmed sequence reads. Quality reports generated before and after quality filtering and trimming show an improvement in multiple quality metrics. (ZIP 2 MB) [file 12864_2013_7026_MOESM1_ESM.zip › FastQC/sep1-filtered_fastqc/sep1-filtered_fastqc/Images/duplication_levels.png]

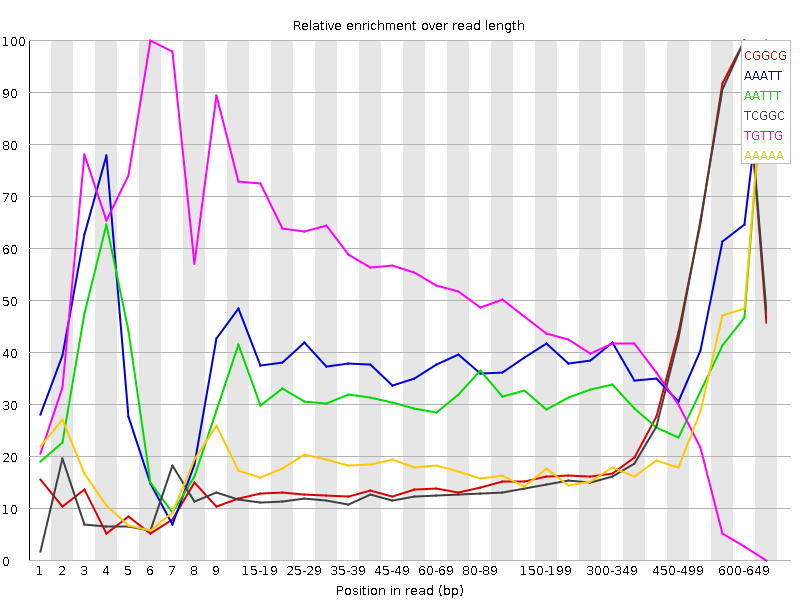

Supplement: Supplementary file 1 — Additional file 1: FastQC reports for untrimmed and trimmed sequence reads. Quality reports generated before and after quality filtering and trimming show an improvement in multiple quality metrics. (ZIP 2 MB) [file 12864_2013_7026_MOESM1_ESM.zip › FastQC/sep1-filtered_fastqc/sep1-filtered_fastqc/Images/kmer_profiles.png]

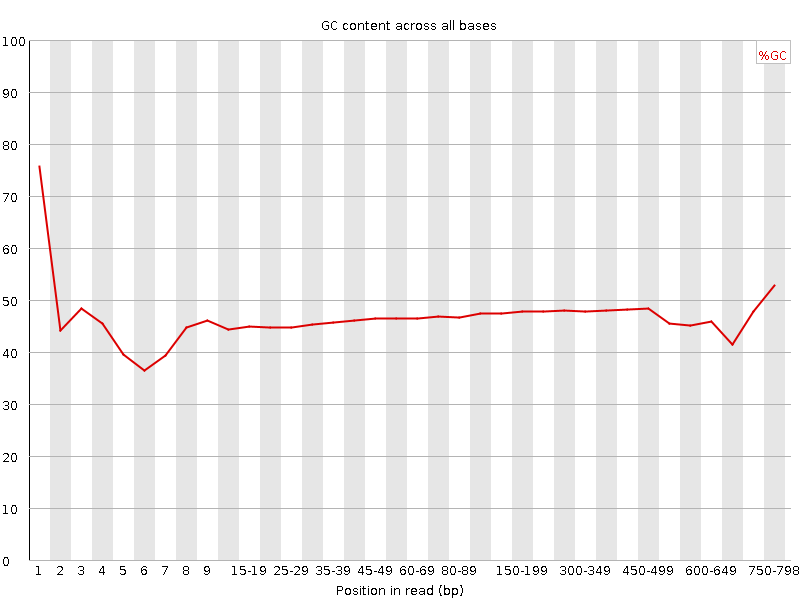

Supplement: Supplementary file 1 — Additional file 1: FastQC reports for untrimmed and trimmed sequence reads. Quality reports generated before and after quality filtering and trimming show an improvement in multiple quality metrics. (ZIP 2 MB) [file 12864_2013_7026_MOESM1_ESM.zip › FastQC/sep1-filtered_fastqc/sep1-filtered_fastqc/Images/per_base_gc_content.png]

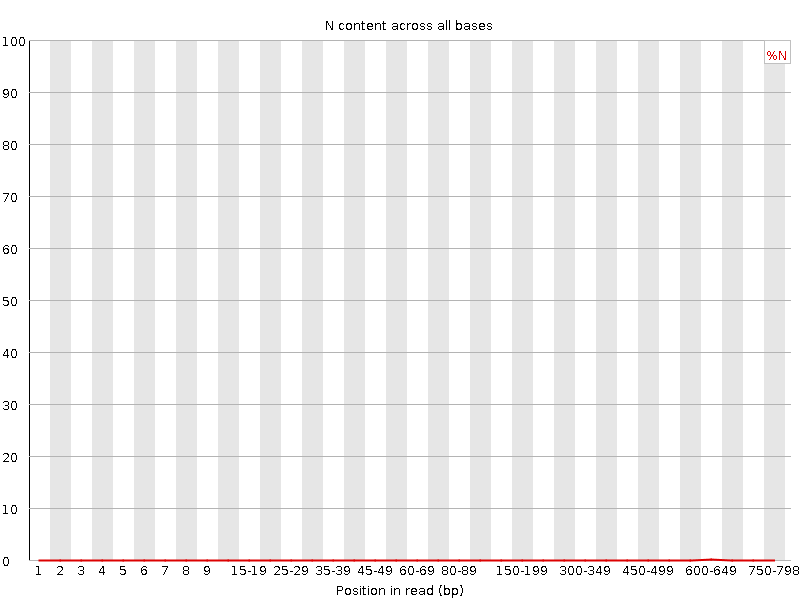

Supplement: Supplementary file 1 — Additional file 1: FastQC reports for untrimmed and trimmed sequence reads. Quality reports generated before and after quality filtering and trimming show an improvement in multiple quality metrics. (ZIP 2 MB) [file 12864_2013_7026_MOESM1_ESM.zip › FastQC/sep1-filtered_fastqc/sep1-filtered_fastqc/Images/per_base_n_content.png]

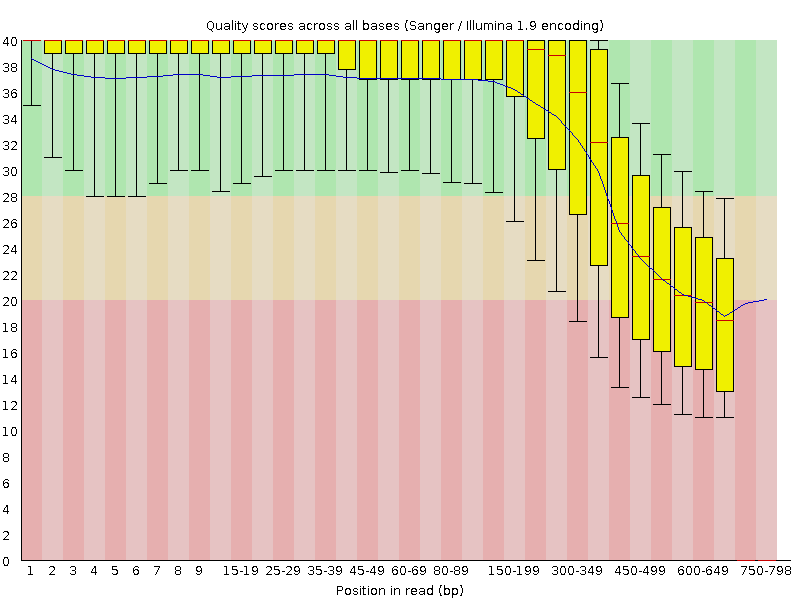

Supplement: Supplementary file 1 — Additional file 1: FastQC reports for untrimmed and trimmed sequence reads. Quality reports generated before and after quality filtering and trimming show an improvement in multiple quality metrics. (ZIP 2 MB) [file 12864_2013_7026_MOESM1_ESM.zip › FastQC/sep1-filtered_fastqc/sep1-filtered_fastqc/Images/per_base_quality.png]

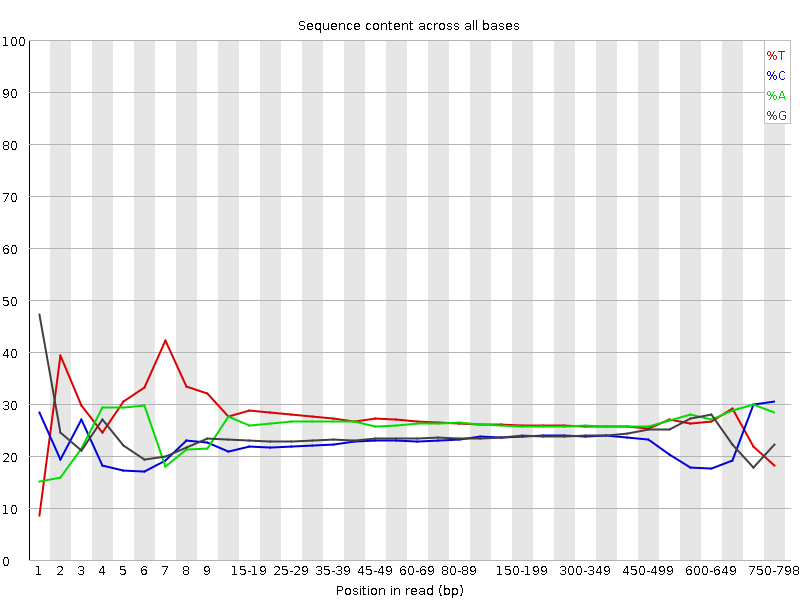

Supplement: Supplementary file 1 — Additional file 1: FastQC reports for untrimmed and trimmed sequence reads. Quality reports generated before and after quality filtering and trimming show an improvement in multiple quality metrics. (ZIP 2 MB) [file 12864_2013_7026_MOESM1_ESM.zip › FastQC/sep1-filtered_fastqc/sep1-filtered_fastqc/Images/per_base_sequence_content.png]

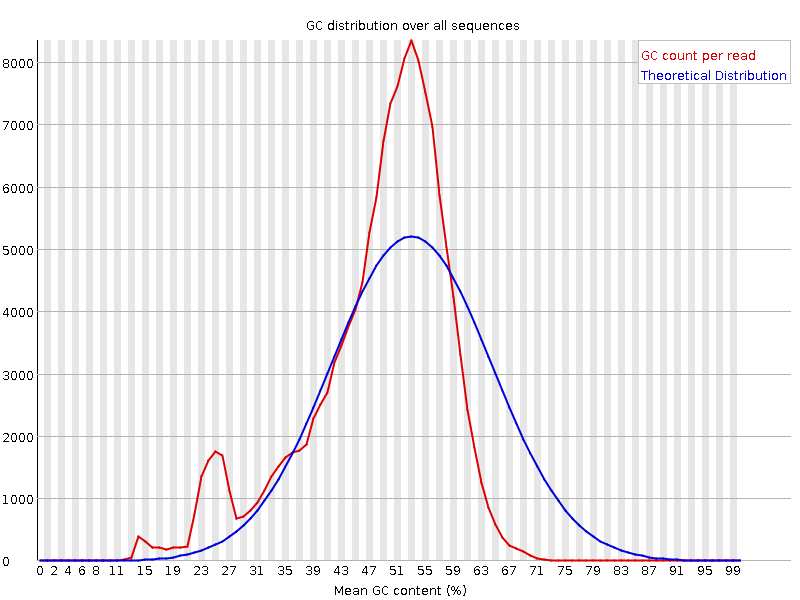

Supplement: Supplementary file 1 — Additional file 1: FastQC reports for untrimmed and trimmed sequence reads. Quality reports generated before and after quality filtering and trimming show an improvement in multiple quality metrics. (ZIP 2 MB) [file 12864_2013_7026_MOESM1_ESM.zip › FastQC/sep1-filtered_fastqc/sep1-filtered_fastqc/Images/per_sequence_gc_content.png]

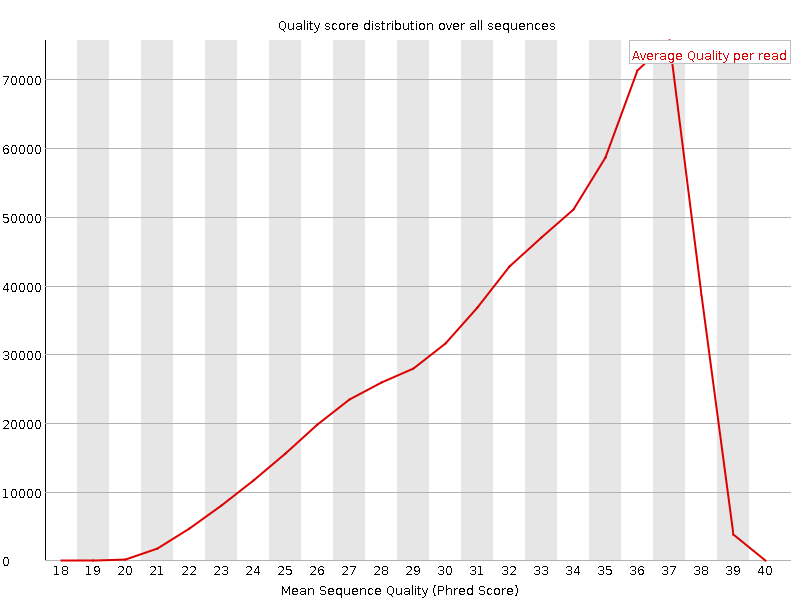

Supplement: Supplementary file 1 — Additional file 1: FastQC reports for untrimmed and trimmed sequence reads. Quality reports generated before and after quality filtering and trimming show an improvement in multiple quality metrics. (ZIP 2 MB) [file 12864_2013_7026_MOESM1_ESM.zip › FastQC/sep1-filtered_fastqc/sep1-filtered_fastqc/Images/per_sequence_quality.png]

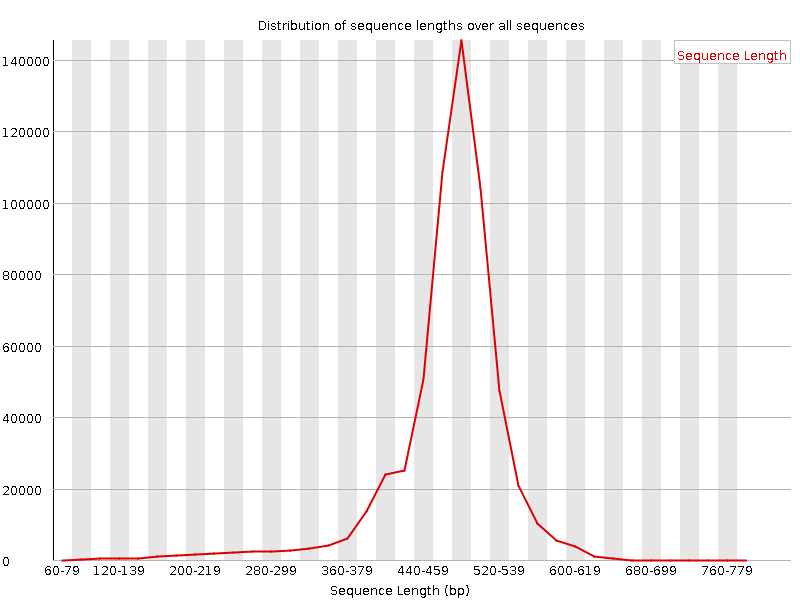

Supplement: Supplementary file 1 — Additional file 1: FastQC reports for untrimmed and trimmed sequence reads. Quality reports generated before and after quality filtering and trimming show an improvement in multiple quality metrics. (ZIP 2 MB) [file 12864_2013_7026_MOESM1_ESM.zip › FastQC/sep1-filtered_fastqc/sep1-filtered_fastqc/Images/sequence_length_distribution.png]

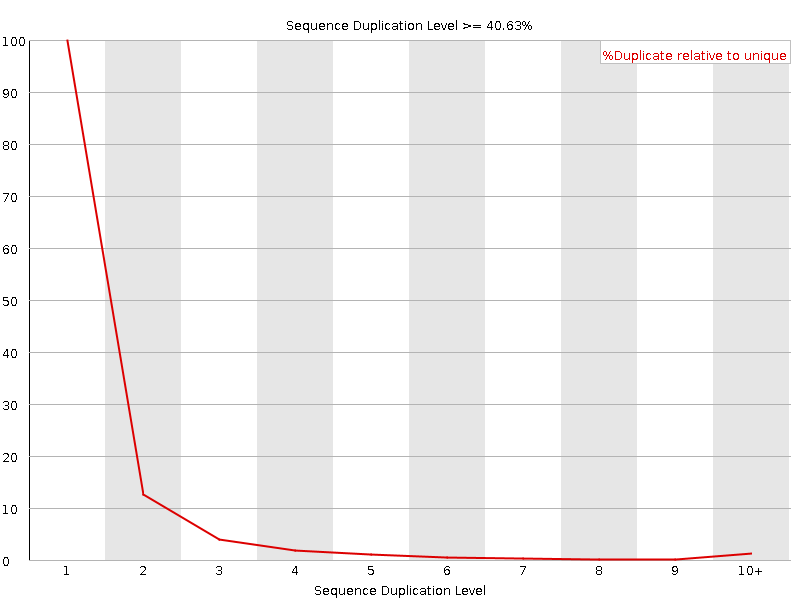

Supplement: Supplementary file 1 — Additional file 1: FastQC reports for untrimmed and trimmed sequence reads. Quality reports generated before and after quality filtering and trimming show an improvement in multiple quality metrics. (ZIP 2 MB) [file 12864_2013_7026_MOESM1_ESM.zip › FastQC/sep2-1-upper_fastqc/sep2-1-upper_fastqc/Images/duplication_levels.png]

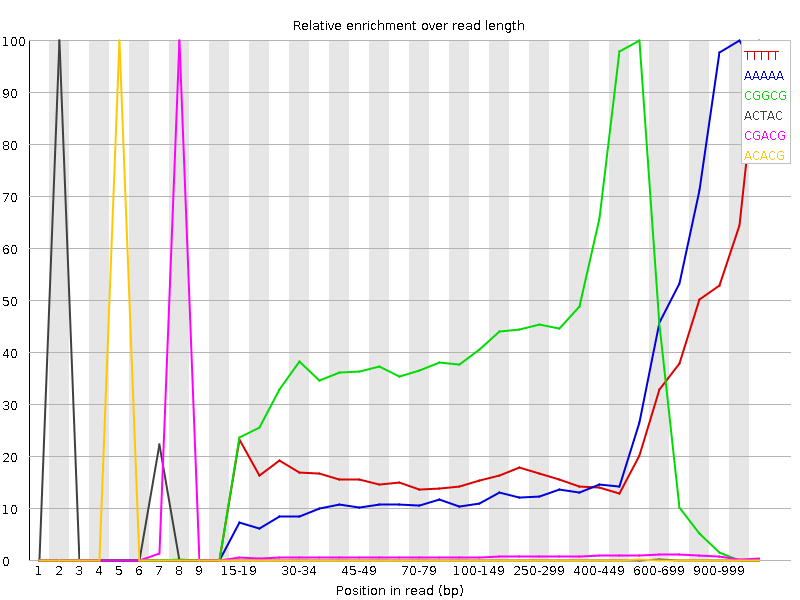

Supplement: Supplementary file 1 — Additional file 1: FastQC reports for untrimmed and trimmed sequence reads. Quality reports generated before and after quality filtering and trimming show an improvement in multiple quality metrics. (ZIP 2 MB) [file 12864_2013_7026_MOESM1_ESM.zip › FastQC/sep2-1-upper_fastqc/sep2-1-upper_fastqc/Images/kmer_profiles.png]

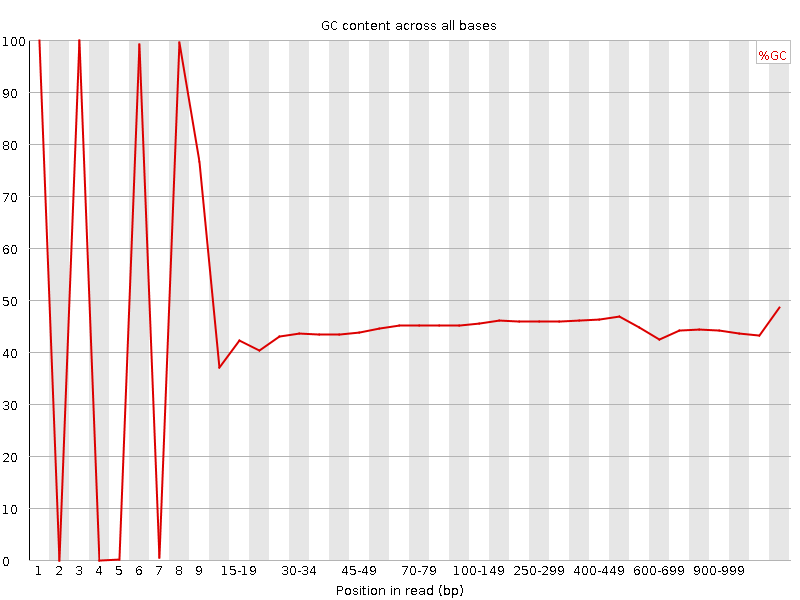

Supplement: Supplementary file 1 — Additional file 1: FastQC reports for untrimmed and trimmed sequence reads. Quality reports generated before and after quality filtering and trimming show an improvement in multiple quality metrics. (ZIP 2 MB) [file 12864_2013_7026_MOESM1_ESM.zip › FastQC/sep2-1-upper_fastqc/sep2-1-upper_fastqc/Images/per_base_gc_content.png]

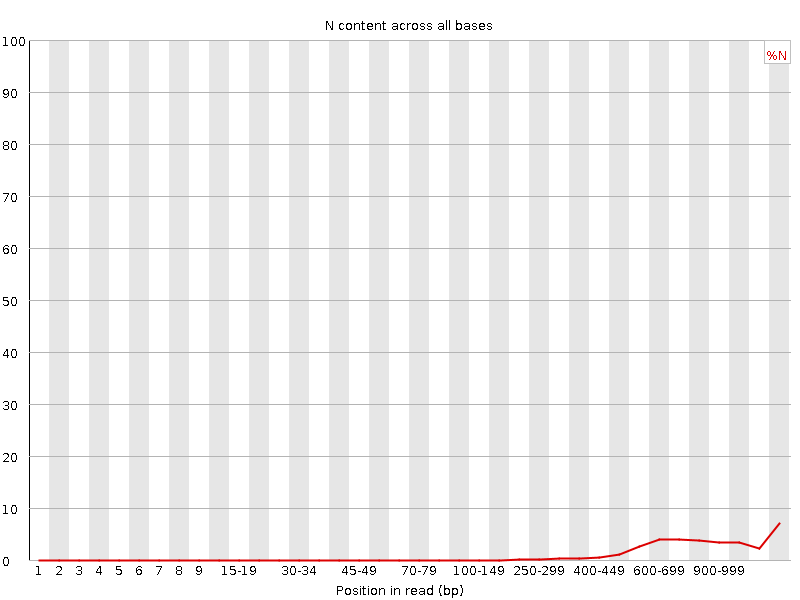

Supplement: Supplementary file 1 — Additional file 1: FastQC reports for untrimmed and trimmed sequence reads. Quality reports generated before and after quality filtering and trimming show an improvement in multiple quality metrics. (ZIP 2 MB) [file 12864_2013_7026_MOESM1_ESM.zip › FastQC/sep2-1-upper_fastqc/sep2-1-upper_fastqc/Images/per_base_n_content.png]

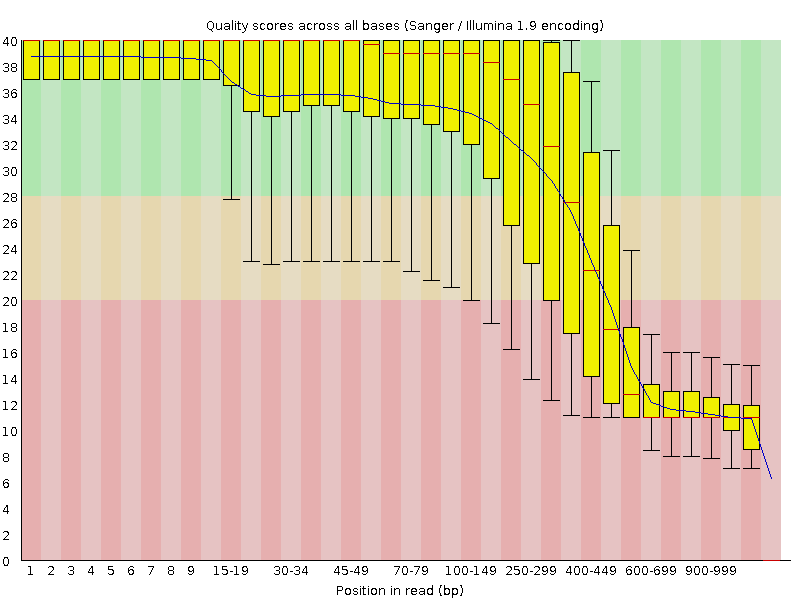

Supplement: Supplementary file 1 — Additional file 1: FastQC reports for untrimmed and trimmed sequence reads. Quality reports generated before and after quality filtering and trimming show an improvement in multiple quality metrics. (ZIP 2 MB) [file 12864_2013_7026_MOESM1_ESM.zip › FastQC/sep2-1-upper_fastqc/sep2-1-upper_fastqc/Images/per_base_quality.png]

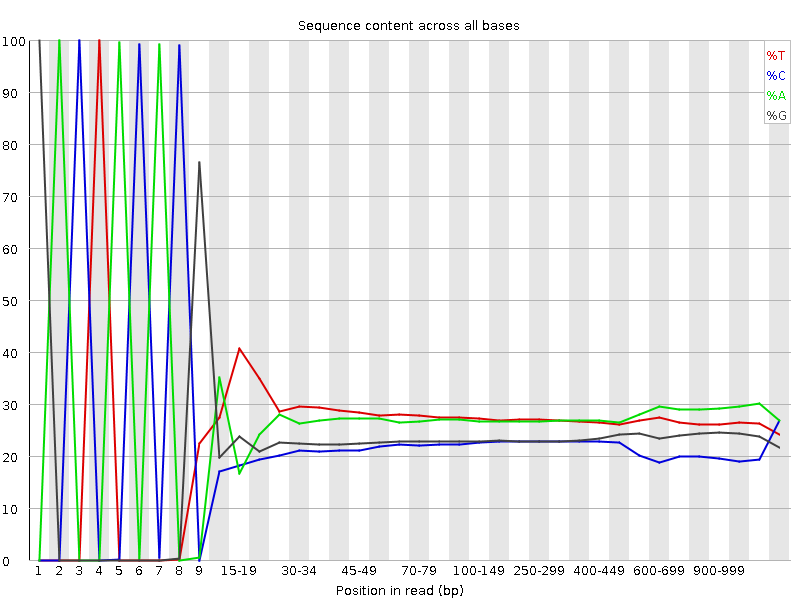

Supplement: Supplementary file 1 — Additional file 1: FastQC reports for untrimmed and trimmed sequence reads. Quality reports generated before and after quality filtering and trimming show an improvement in multiple quality metrics. (ZIP 2 MB) [file 12864_2013_7026_MOESM1_ESM.zip › FastQC/sep2-1-upper_fastqc/sep2-1-upper_fastqc/Images/per_base_sequence_content.png]

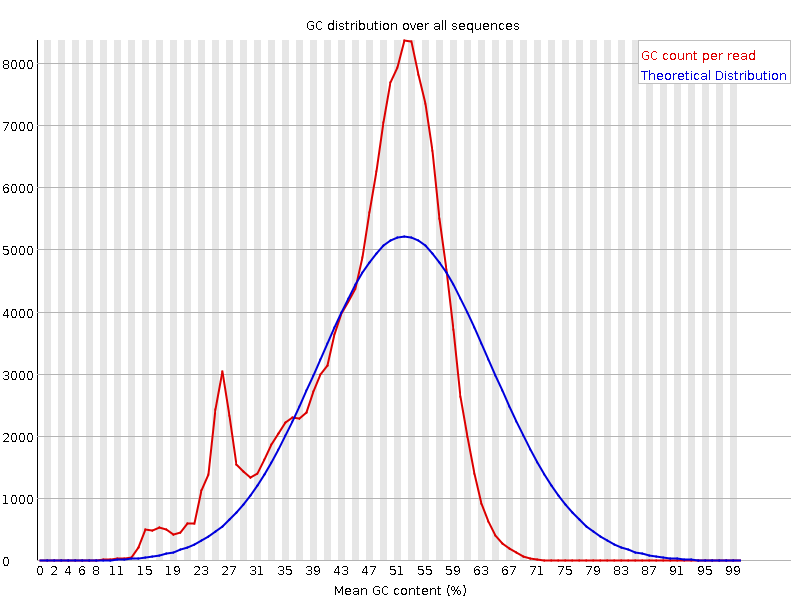

Supplement: Supplementary file 1 — Additional file 1: FastQC reports for untrimmed and trimmed sequence reads. Quality reports generated before and after quality filtering and trimming show an improvement in multiple quality metrics. (ZIP 2 MB) [file 12864_2013_7026_MOESM1_ESM.zip › FastQC/sep2-1-upper_fastqc/sep2-1-upper_fastqc/Images/per_sequence_gc_content.png]

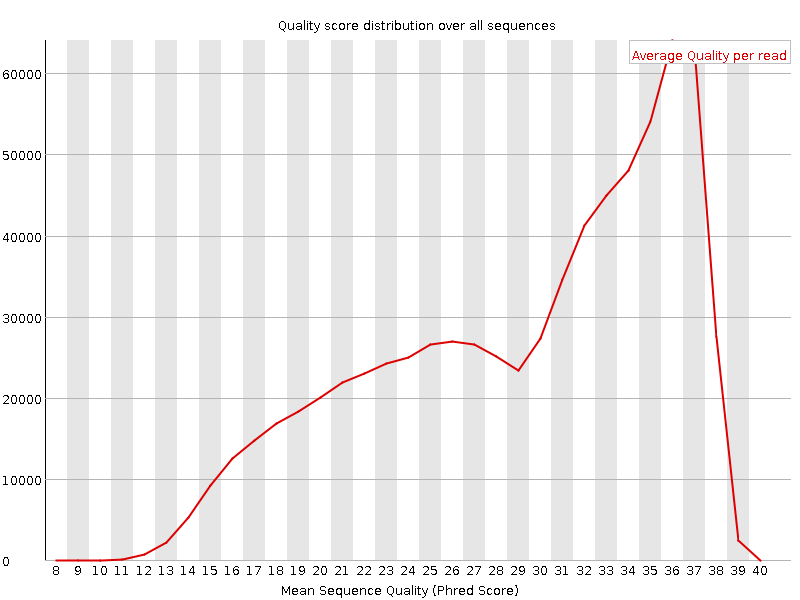

Supplement: Supplementary file 1 — Additional file 1: FastQC reports for untrimmed and trimmed sequence reads. Quality reports generated before and after quality filtering and trimming show an improvement in multiple quality metrics. (ZIP 2 MB) [file 12864_2013_7026_MOESM1_ESM.zip › FastQC/sep2-1-upper_fastqc/sep2-1-upper_fastqc/Images/per_sequence_quality.png]

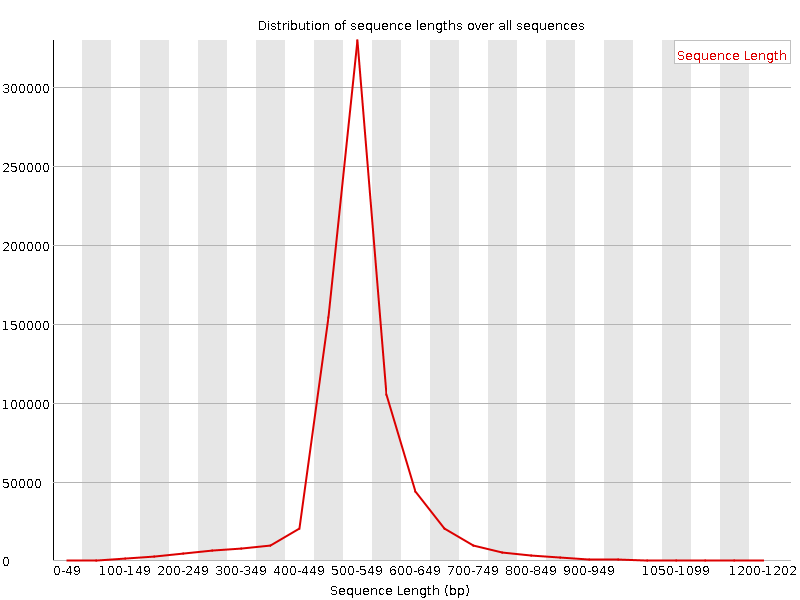

Supplement: Supplementary file 1 — Additional file 1: FastQC reports for untrimmed and trimmed sequence reads. Quality reports generated before and after quality filtering and trimming show an improvement in multiple quality metrics. (ZIP 2 MB) [file 12864_2013_7026_MOESM1_ESM.zip › FastQC/sep2-1-upper_fastqc/sep2-1-upper_fastqc/Images/sequence_length_distribution.png]

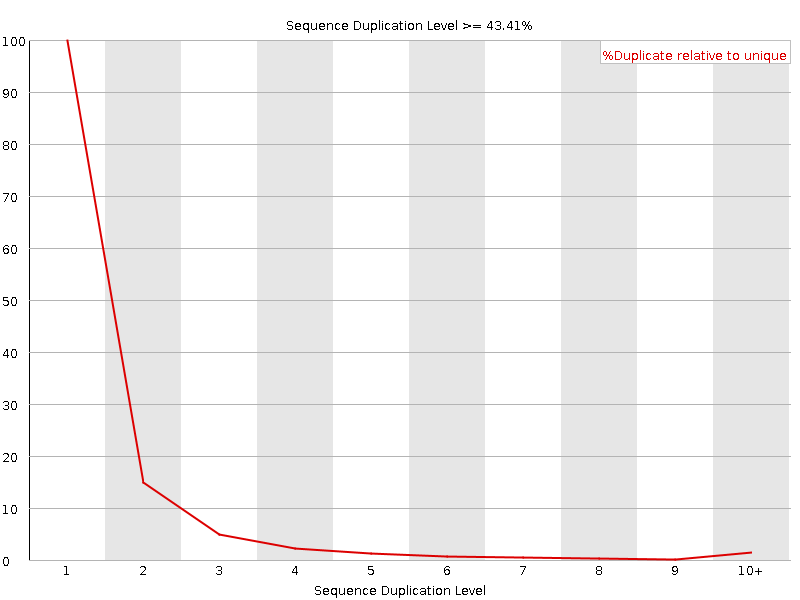

Supplement: Supplementary file 1 — Additional file 1: FastQC reports for untrimmed and trimmed sequence reads. Quality reports generated before and after quality filtering and trimming show an improvement in multiple quality metrics. (ZIP 2 MB) [file 12864_2013_7026_MOESM1_ESM.zip › FastQC/sep2-filtered_fastqc/sep2-filtered_fastqc/Images/duplication_levels.png]

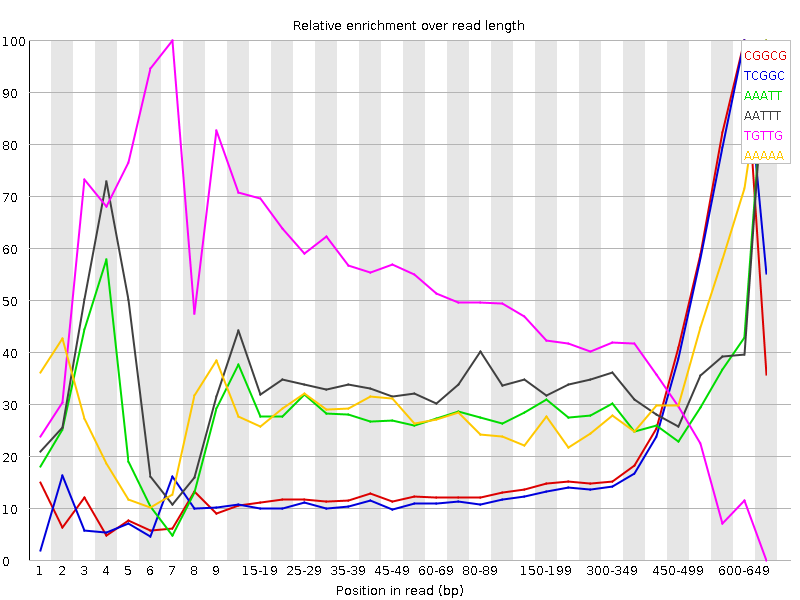

Supplement: Supplementary file 1 — Additional file 1: FastQC reports for untrimmed and trimmed sequence reads. Quality reports generated before and after quality filtering and trimming show an improvement in multiple quality metrics. (ZIP 2 MB) [file 12864_2013_7026_MOESM1_ESM.zip › FastQC/sep2-filtered_fastqc/sep2-filtered_fastqc/Images/kmer_profiles.png]

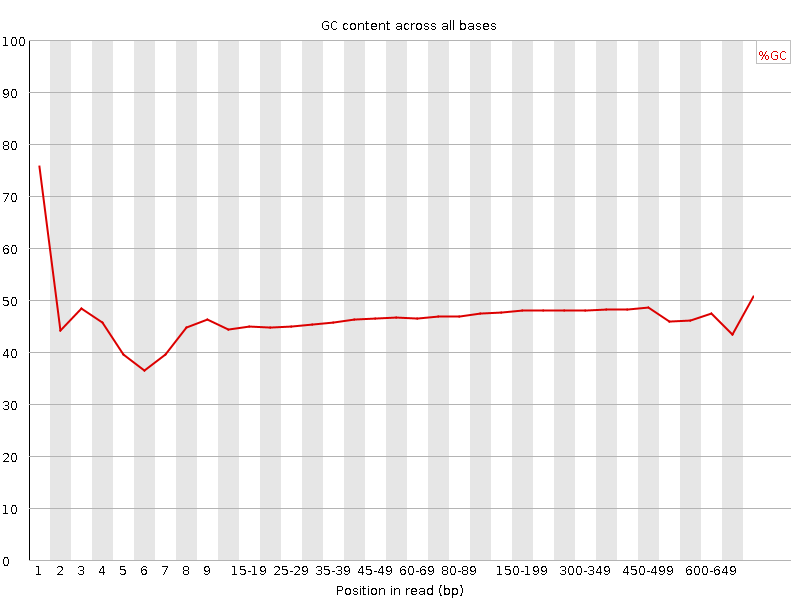

Supplement: Supplementary file 1 — Additional file 1: FastQC reports for untrimmed and trimmed sequence reads. Quality reports generated before and after quality filtering and trimming show an improvement in multiple quality metrics. (ZIP 2 MB) [file 12864_2013_7026_MOESM1_ESM.zip › FastQC/sep2-filtered_fastqc/sep2-filtered_fastqc/Images/per_base_gc_content.png]

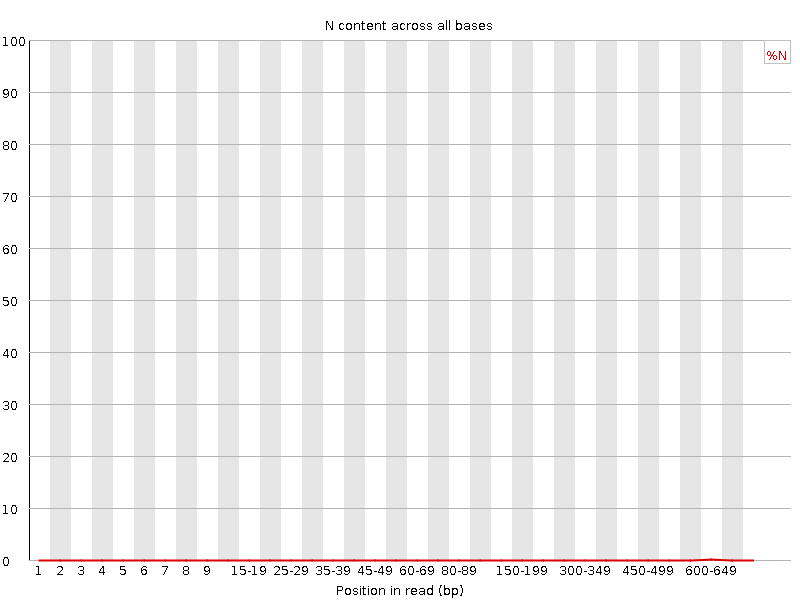

Supplement: Supplementary file 1 — Additional file 1: FastQC reports for untrimmed and trimmed sequence reads. Quality reports generated before and after quality filtering and trimming show an improvement in multiple quality metrics. (ZIP 2 MB) [file 12864_2013_7026_MOESM1_ESM.zip › FastQC/sep2-filtered_fastqc/sep2-filtered_fastqc/Images/per_base_n_content.png]

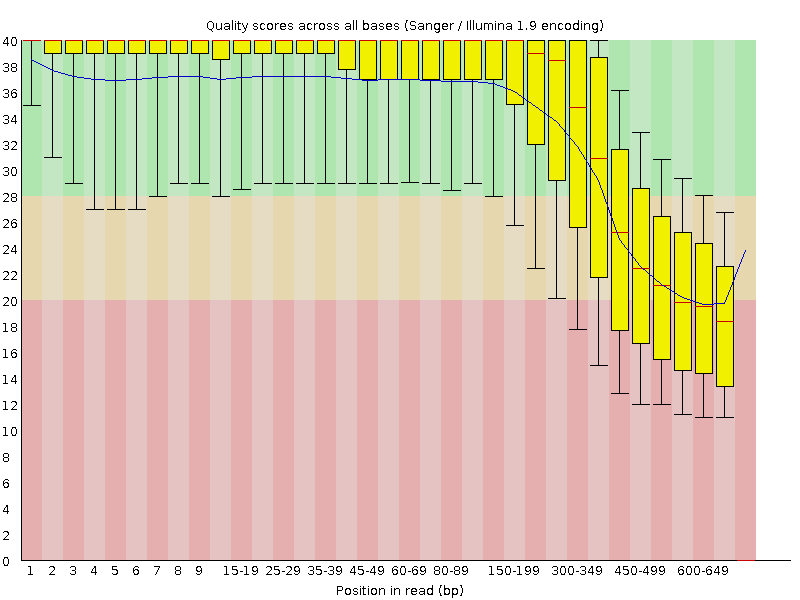

Supplement: Supplementary file 1 — Additional file 1: FastQC reports for untrimmed and trimmed sequence reads. Quality reports generated before and after quality filtering and trimming show an improvement in multiple quality metrics. (ZIP 2 MB) [file 12864_2013_7026_MOESM1_ESM.zip › FastQC/sep2-filtered_fastqc/sep2-filtered_fastqc/Images/per_base_quality.png]

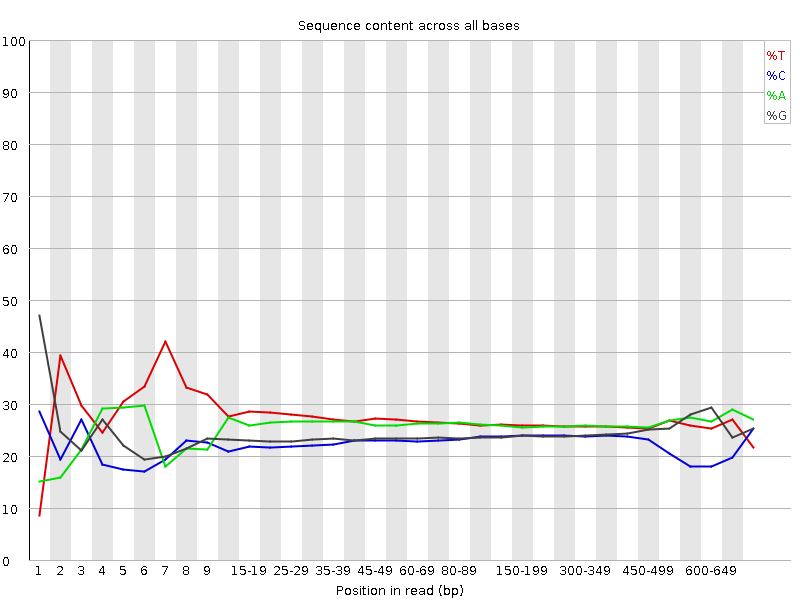

Supplement: Supplementary file 1 — Additional file 1: FastQC reports for untrimmed and trimmed sequence reads. Quality reports generated before and after quality filtering and trimming show an improvement in multiple quality metrics. (ZIP 2 MB) [file 12864_2013_7026_MOESM1_ESM.zip › FastQC/sep2-filtered_fastqc/sep2-filtered_fastqc/Images/per_base_sequence_content.png]

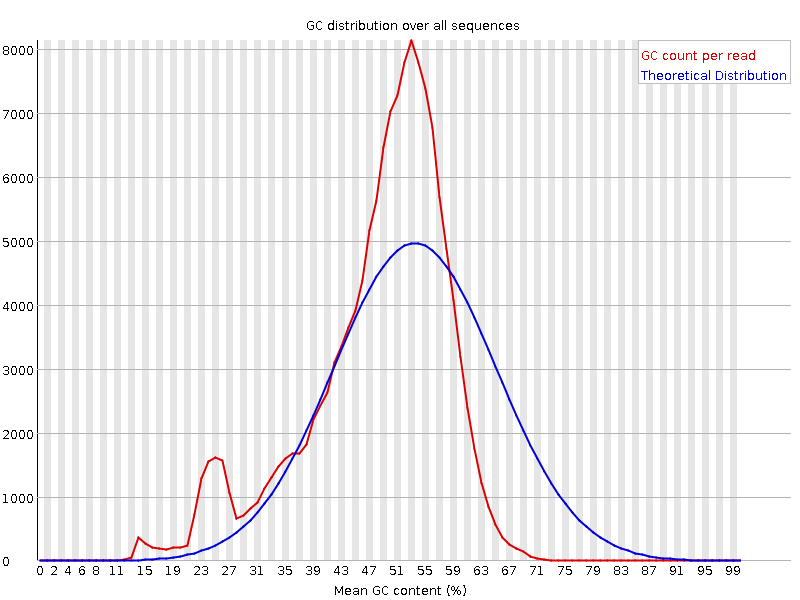

Supplement: Supplementary file 1 — Additional file 1: FastQC reports for untrimmed and trimmed sequence reads. Quality reports generated before and after quality filtering and trimming show an improvement in multiple quality metrics. (ZIP 2 MB) [file 12864_2013_7026_MOESM1_ESM.zip › FastQC/sep2-filtered_fastqc/sep2-filtered_fastqc/Images/per_sequence_gc_content.png]

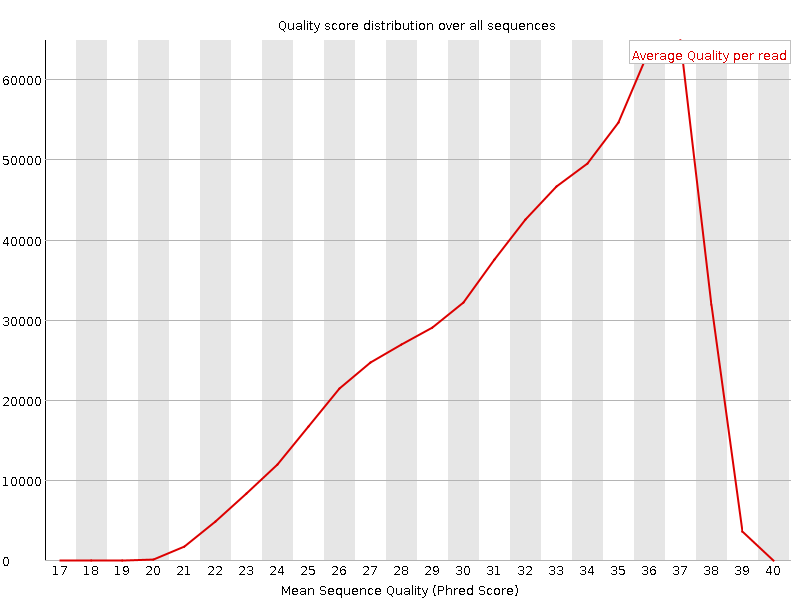

Supplement: Supplementary file 1 — Additional file 1: FastQC reports for untrimmed and trimmed sequence reads. Quality reports generated before and after quality filtering and trimming show an improvement in multiple quality metrics. (ZIP 2 MB) [file 12864_2013_7026_MOESM1_ESM.zip › FastQC/sep2-filtered_fastqc/sep2-filtered_fastqc/Images/per_sequence_quality.png]

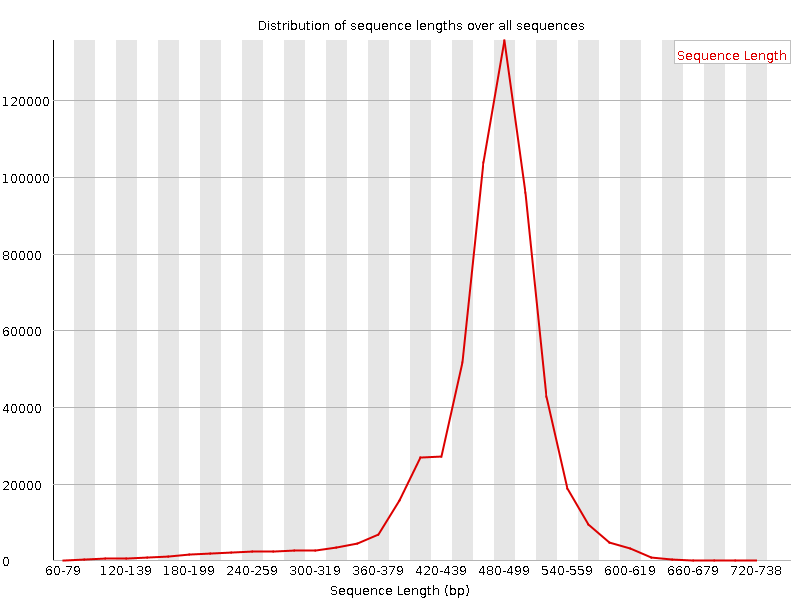

Supplement: Supplementary file 1 — Additional file 1: FastQC reports for untrimmed and trimmed sequence reads. Quality reports generated before and after quality filtering and trimming show an improvement in multiple quality metrics. (ZIP 2 MB) [file 12864_2013_7026_MOESM1_ESM.zip › FastQC/sep2-filtered_fastqc/sep2-filtered_fastqc/Images/sequence_length_distribution.png]

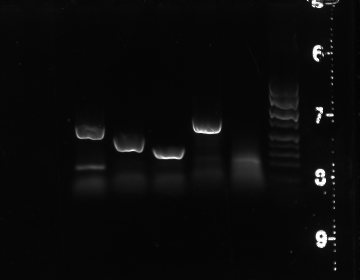

Supplement: Supplementary file 2 — Additional file 2: PCR validation of assembled contigs. Primers designed from bioinformatically generated contigs annotated using the Drosophila transcriptome produced the expected band sizes (from left to right) for engrailed, escargot, and evenskipped. (TIFF 216 KB) [file 12864_2013_7026_MOESM2_ESM.tiff]

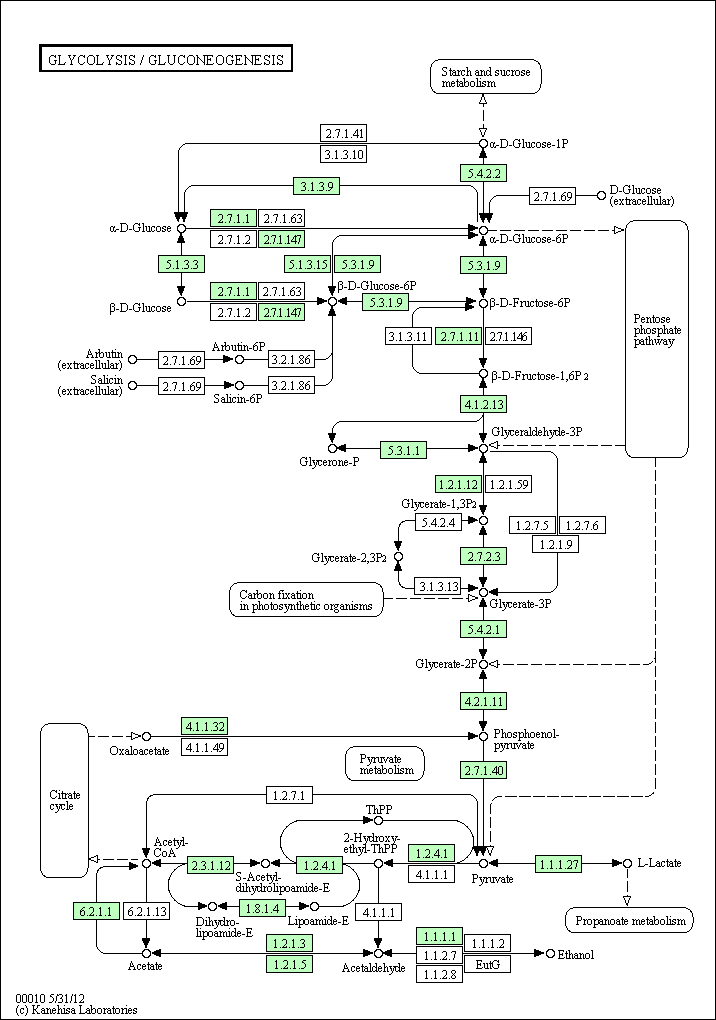

Supplement: Supplementary file 3 — Additional file 3: KEGG classification and functional maps of assembled contigs. Contigs annotated using KEGG Automatic Annotation Server identified sequences in a broad range of functional groups including developmental pathways and cell signaling. (ZIP 11 MB) [file 12864_2013_7026_MOESM3_ESM.zip › KEGG classification/map/map00010.png]

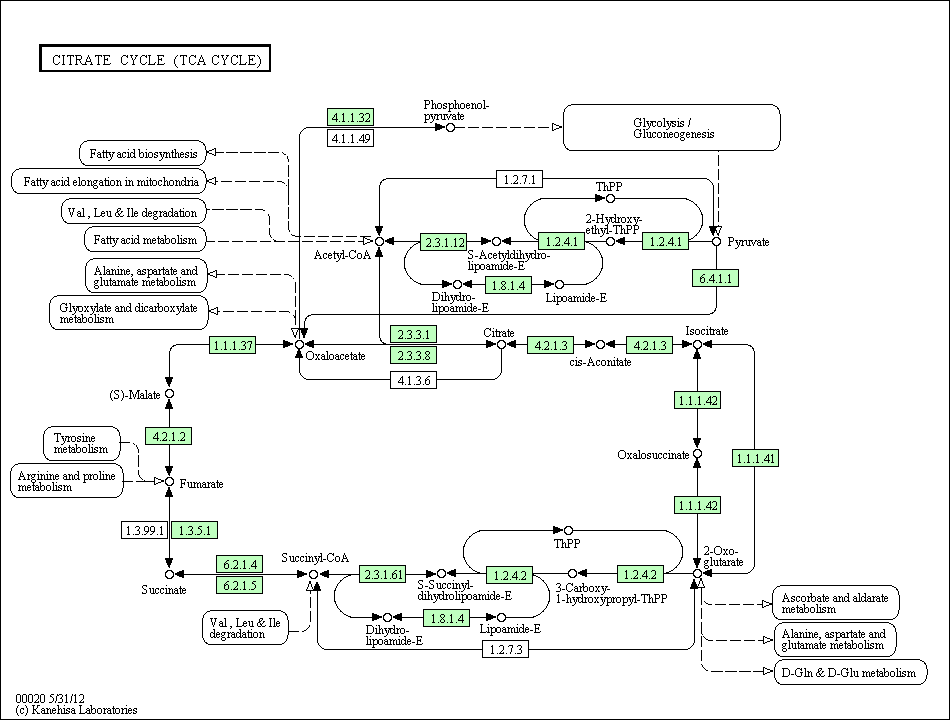

Supplement: Supplementary file 3 — Additional file 3: KEGG classification and functional maps of assembled contigs. Contigs annotated using KEGG Automatic Annotation Server identified sequences in a broad range of functional groups including developmental pathways and cell signaling. (ZIP 11 MB) [file 12864_2013_7026_MOESM3_ESM.zip › KEGG classification/map/map00020.png]

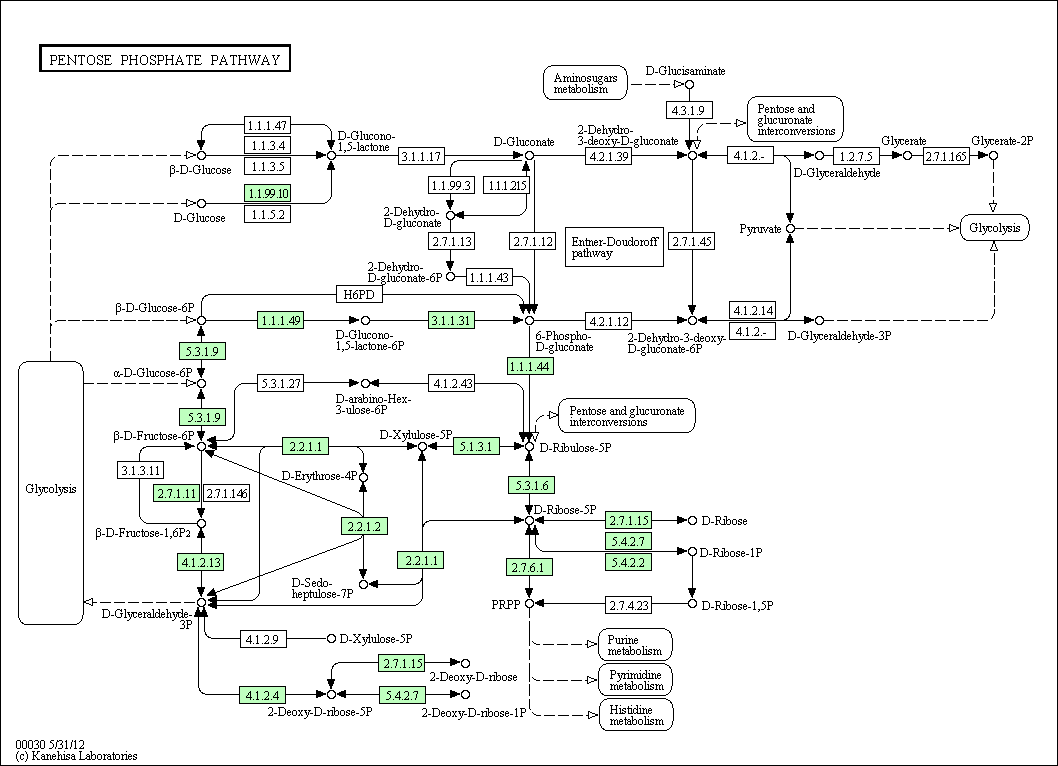

Supplement: Supplementary file 3 — Additional file 3: KEGG classification and functional maps of assembled contigs. Contigs annotated using KEGG Automatic Annotation Server identified sequences in a broad range of functional groups including developmental pathways and cell signaling. (ZIP 11 MB) [file 12864_2013_7026_MOESM3_ESM.zip › KEGG classification/map/map00030.png]

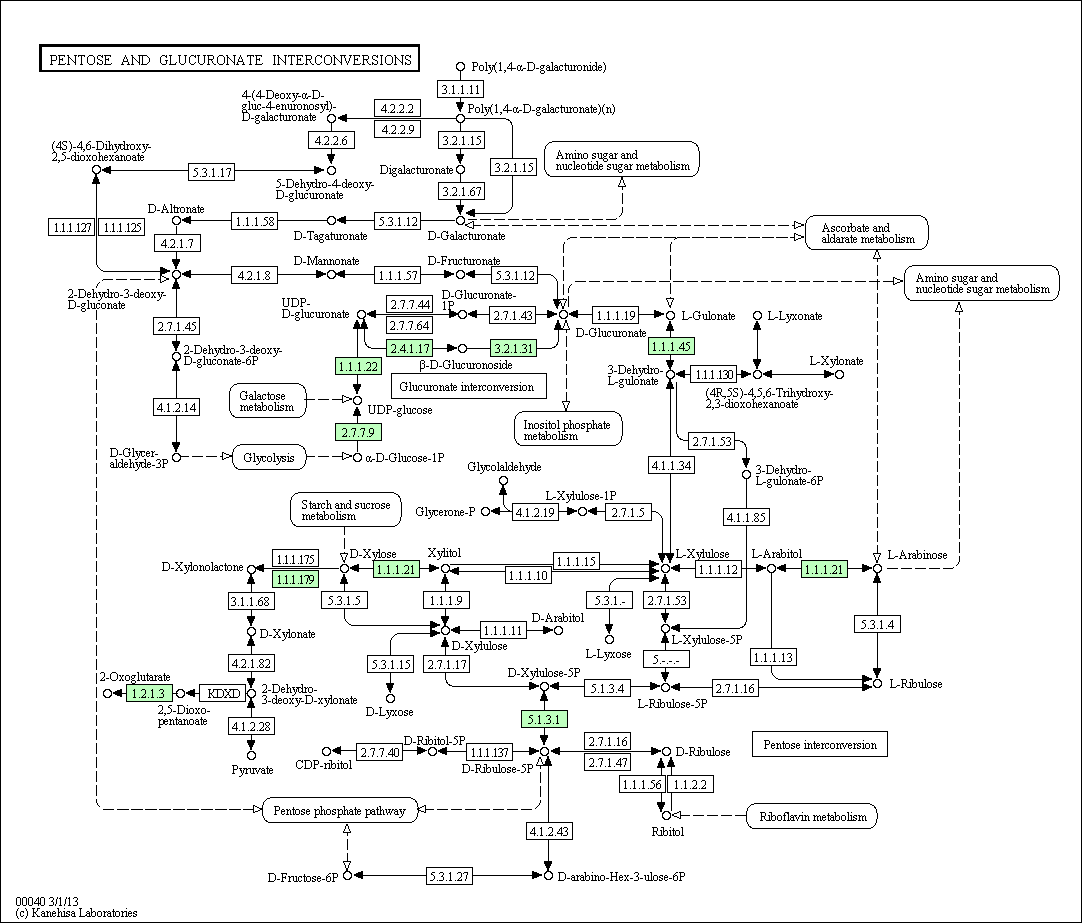

Supplement: Supplementary file 3 — Additional file 3: KEGG classification and functional maps of assembled contigs. Contigs annotated using KEGG Automatic Annotation Server identified sequences in a broad range of functional groups including developmental pathways and cell signaling. (ZIP 11 MB) [file 12864_2013_7026_MOESM3_ESM.zip › KEGG classification/map/map00040.png]

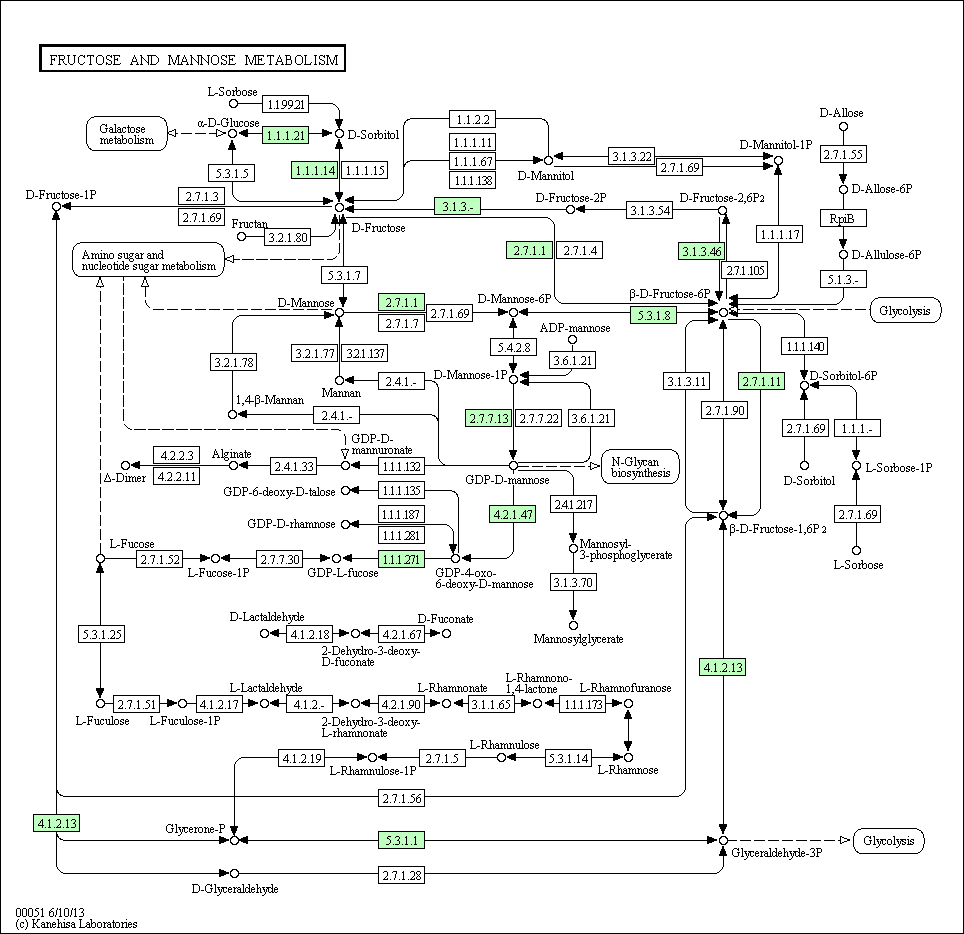

Supplement: Supplementary file 3 — Additional file 3: KEGG classification and functional maps of assembled contigs. Contigs annotated using KEGG Automatic Annotation Server identified sequences in a broad range of functional groups including developmental pathways and cell signaling. (ZIP 11 MB) [file 12864_2013_7026_MOESM3_ESM.zip › KEGG classification/map/map00051.png]

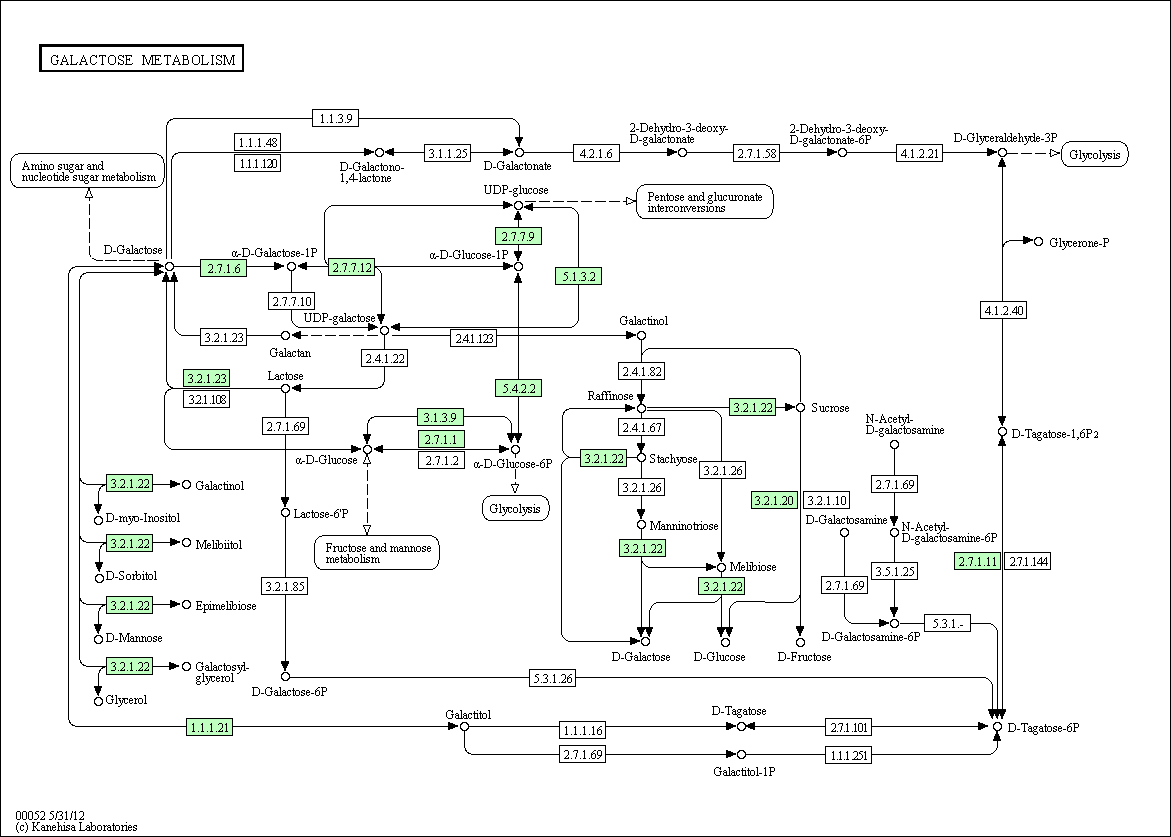

Supplement: Supplementary file 3 — Additional file 3: KEGG classification and functional maps of assembled contigs. Contigs annotated using KEGG Automatic Annotation Server identified sequences in a broad range of functional groups including developmental pathways and cell signaling. (ZIP 11 MB) [file 12864_2013_7026_MOESM3_ESM.zip › KEGG classification/map/map00052.png]

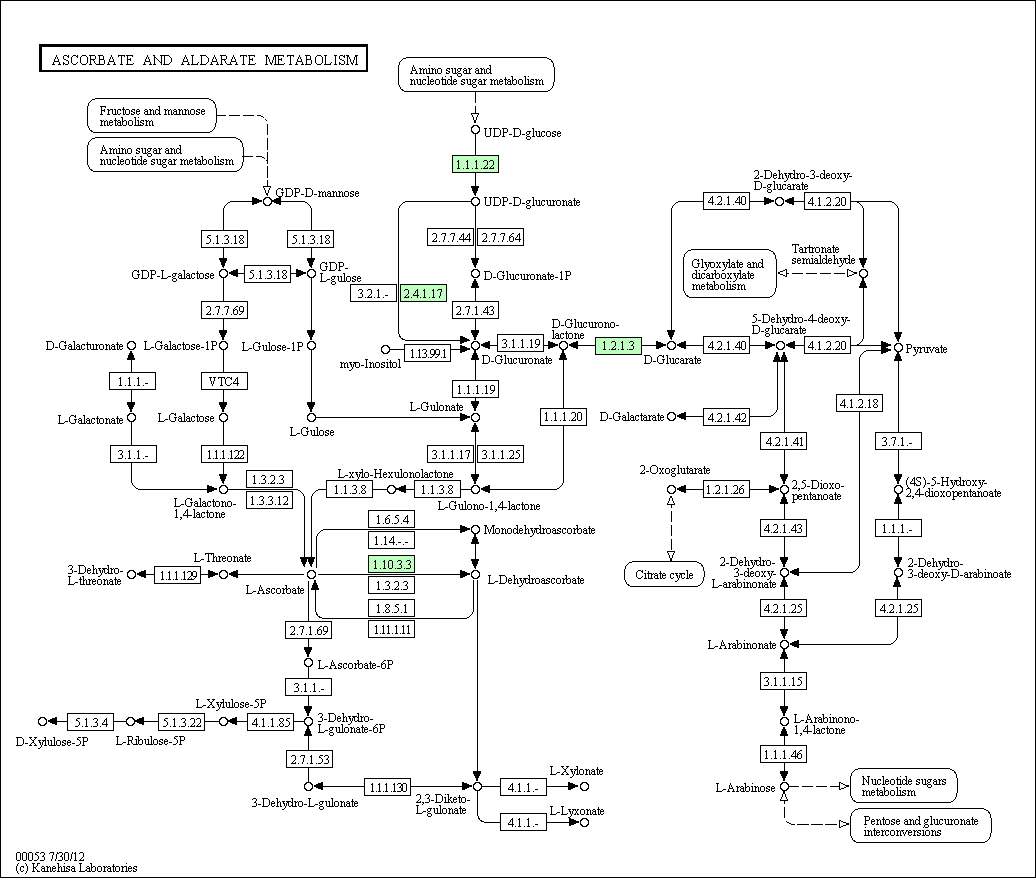

Supplement: Supplementary file 3 — Additional file 3: KEGG classification and functional maps of assembled contigs. Contigs annotated using KEGG Automatic Annotation Server identified sequences in a broad range of functional groups including developmental pathways and cell signaling. (ZIP 11 MB) [file 12864_2013_7026_MOESM3_ESM.zip › KEGG classification/map/map00053.png]

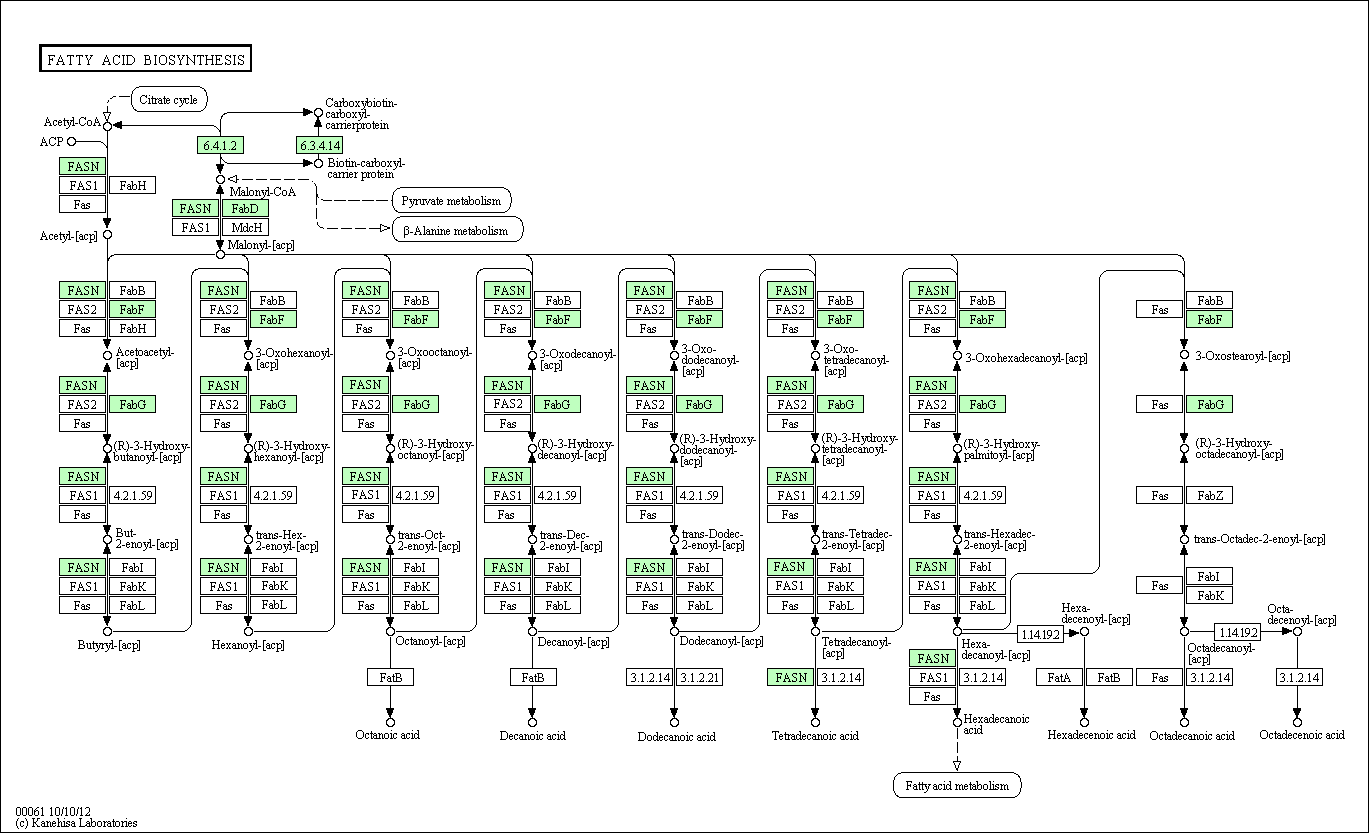

Supplement: Supplementary file 3 — Additional file 3: KEGG classification and functional maps of assembled contigs. Contigs annotated using KEGG Automatic Annotation Server identified sequences in a broad range of functional groups including developmental pathways and cell signaling. (ZIP 11 MB) [file 12864_2013_7026_MOESM3_ESM.zip › KEGG classification/map/map00061.png]

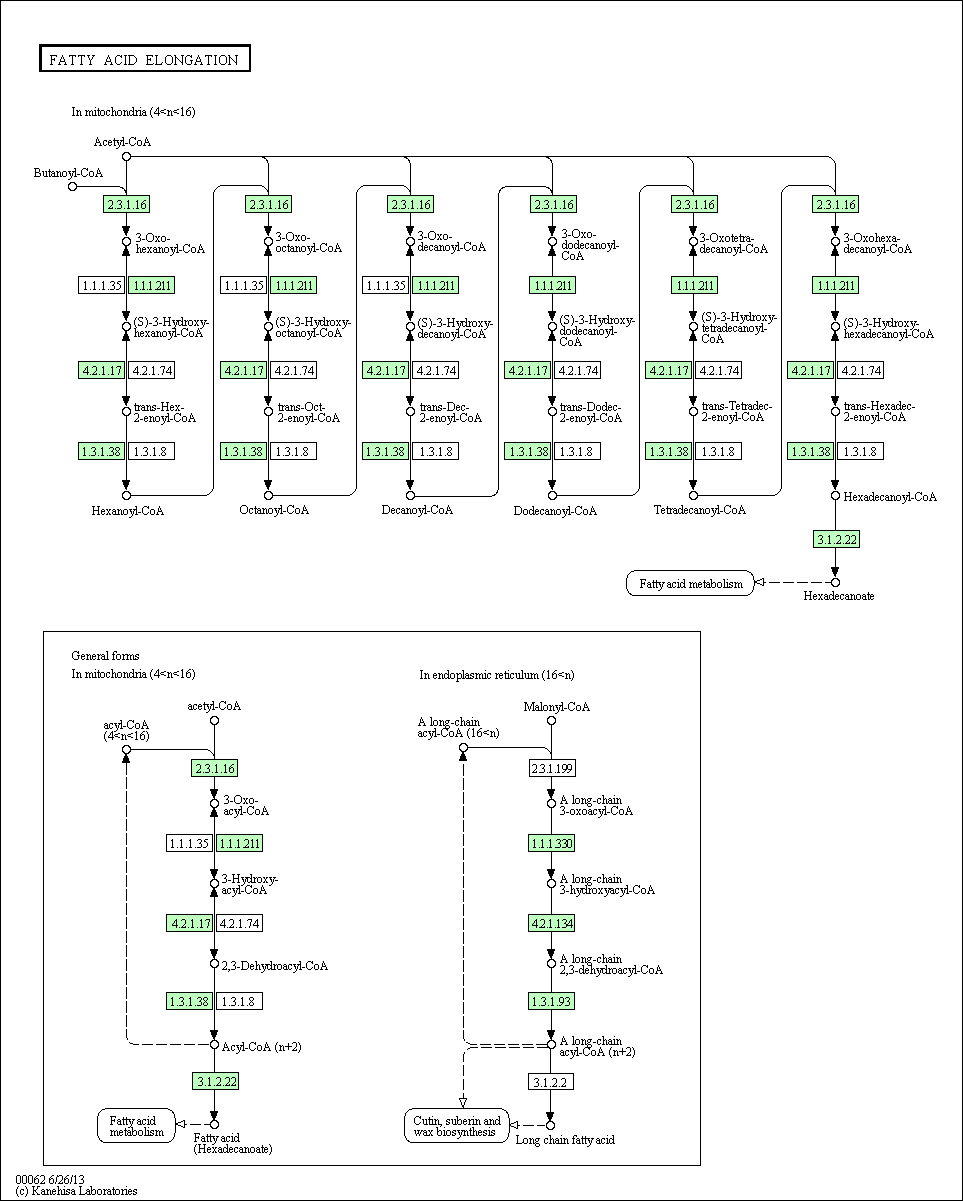

Supplement: Supplementary file 3 — Additional file 3: KEGG classification and functional maps of assembled contigs. Contigs annotated using KEGG Automatic Annotation Server identified sequences in a broad range of functional groups including developmental pathways and cell signaling. (ZIP 11 MB) [file 12864_2013_7026_MOESM3_ESM.zip › KEGG classification/map/map00062.png]

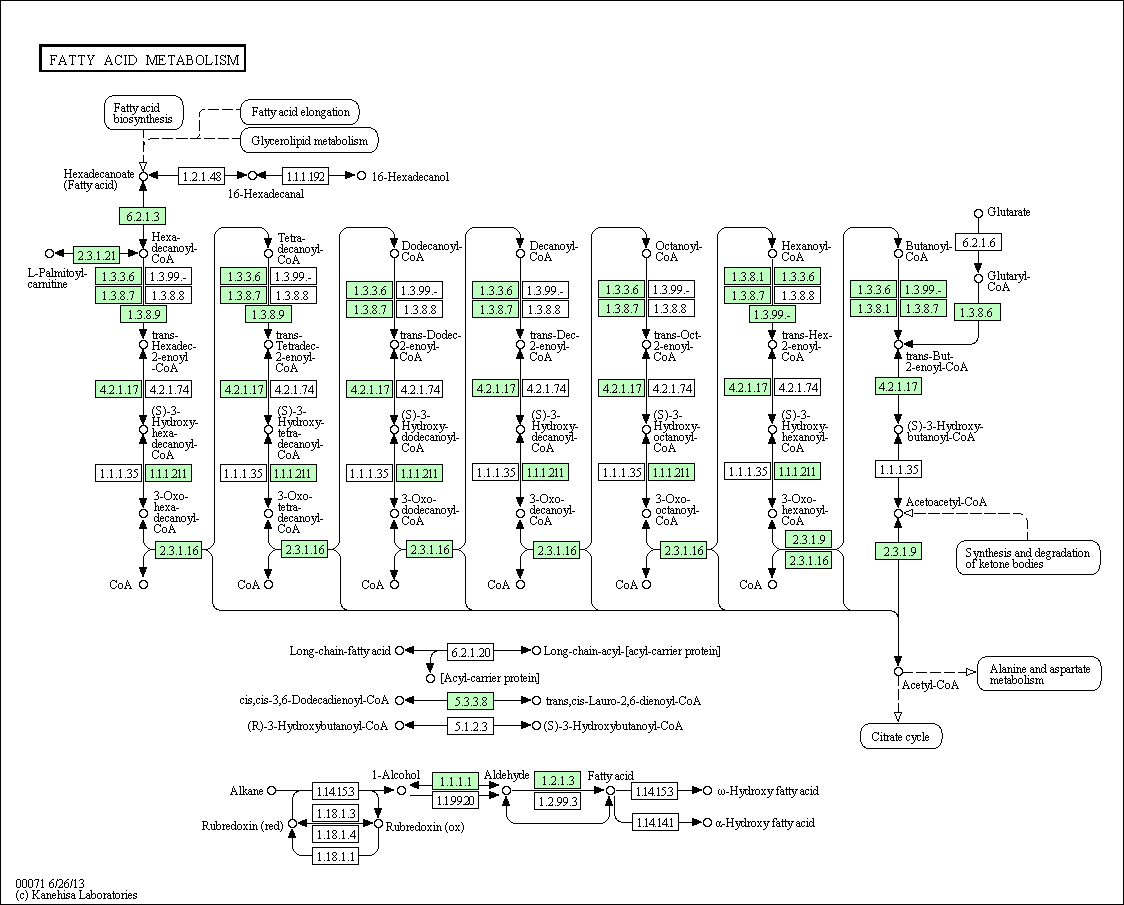

Supplement: Supplementary file 3 — Additional file 3: KEGG classification and functional maps of assembled contigs. Contigs annotated using KEGG Automatic Annotation Server identified sequences in a broad range of functional groups including developmental pathways and cell signaling. (ZIP 11 MB) [file 12864_2013_7026_MOESM3_ESM.zip › KEGG classification/map/map00071.png]

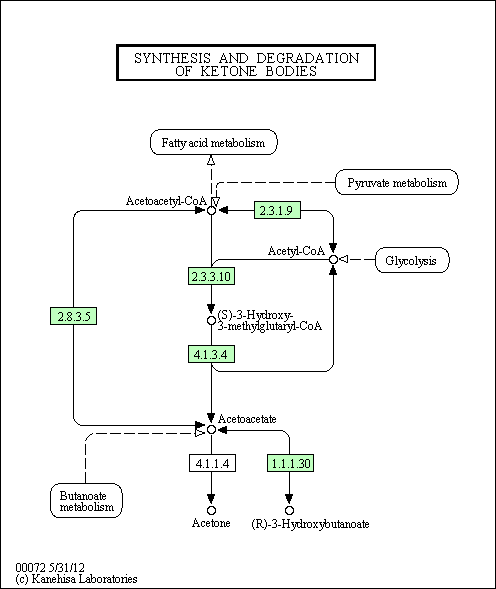

Supplement: Supplementary file 3 — Additional file 3: KEGG classification and functional maps of assembled contigs. Contigs annotated using KEGG Automatic Annotation Server identified sequences in a broad range of functional groups including developmental pathways and cell signaling. (ZIP 11 MB) [file 12864_2013_7026_MOESM3_ESM.zip › KEGG classification/map/map00072.png]

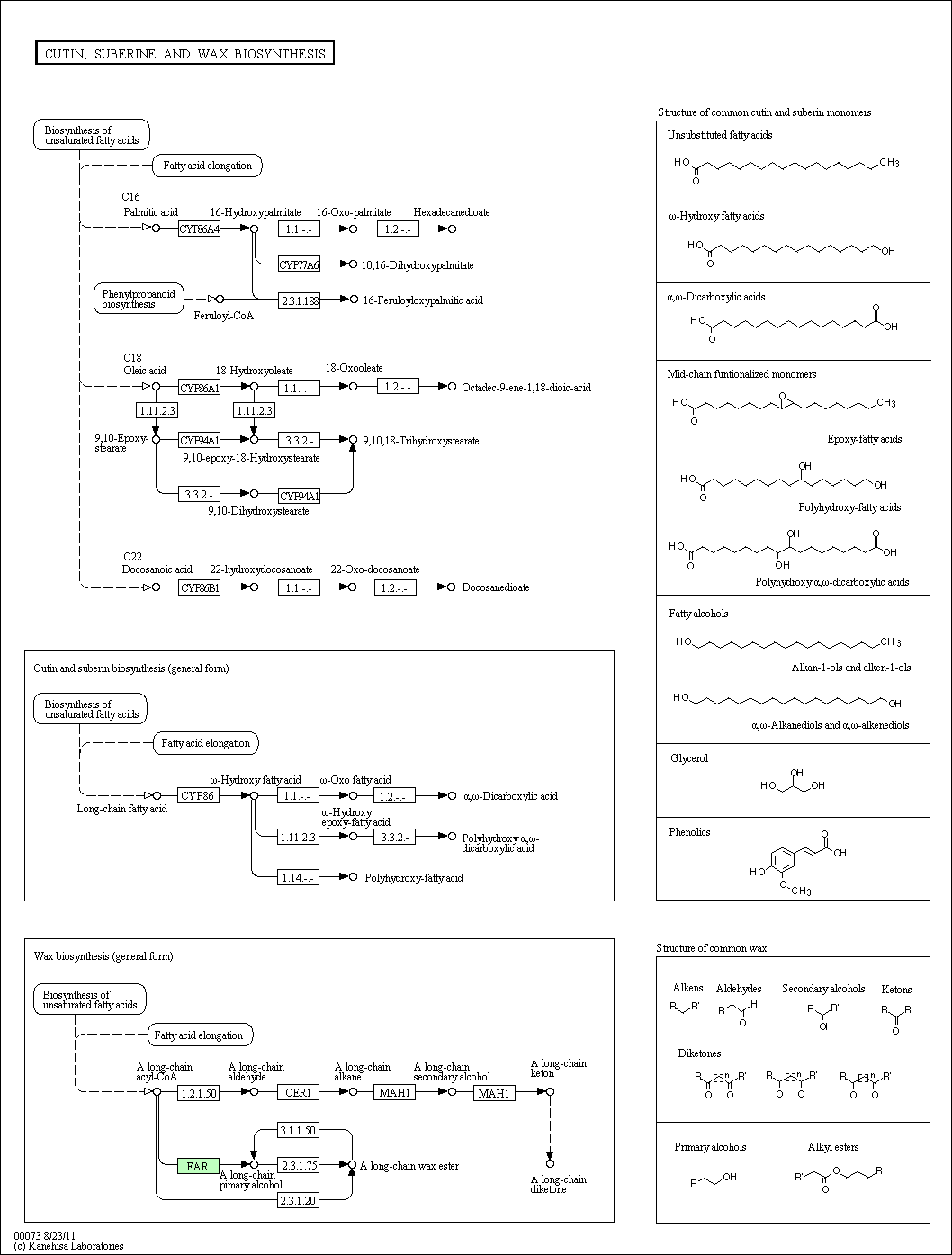

Supplement: Supplementary file 3 — Additional file 3: KEGG classification and functional maps of assembled contigs. Contigs annotated using KEGG Automatic Annotation Server identified sequences in a broad range of functional groups including developmental pathways and cell signaling. (ZIP 11 MB) [file 12864_2013_7026_MOESM3_ESM.zip › KEGG classification/map/map00073.png]

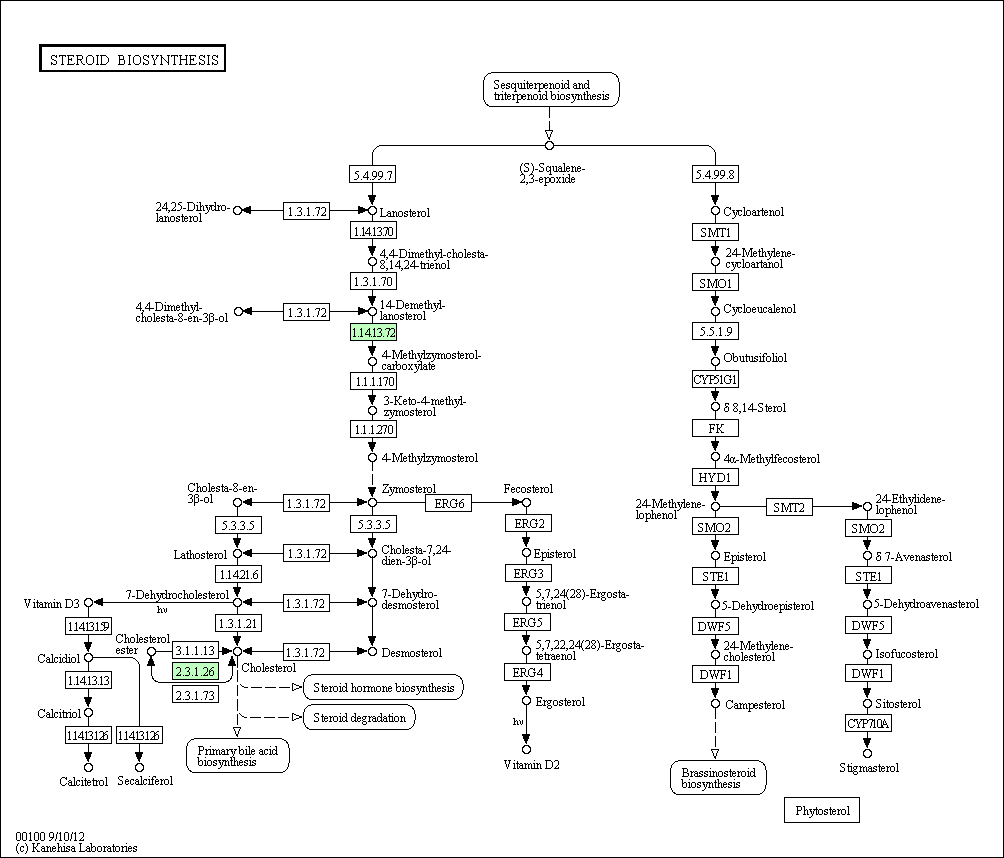

Supplement: Supplementary file 3 — Additional file 3: KEGG classification and functional maps of assembled contigs. Contigs annotated using KEGG Automatic Annotation Server identified sequences in a broad range of functional groups including developmental pathways and cell signaling. (ZIP 11 MB) [file 12864_2013_7026_MOESM3_ESM.zip › KEGG classification/map/map00100.png]

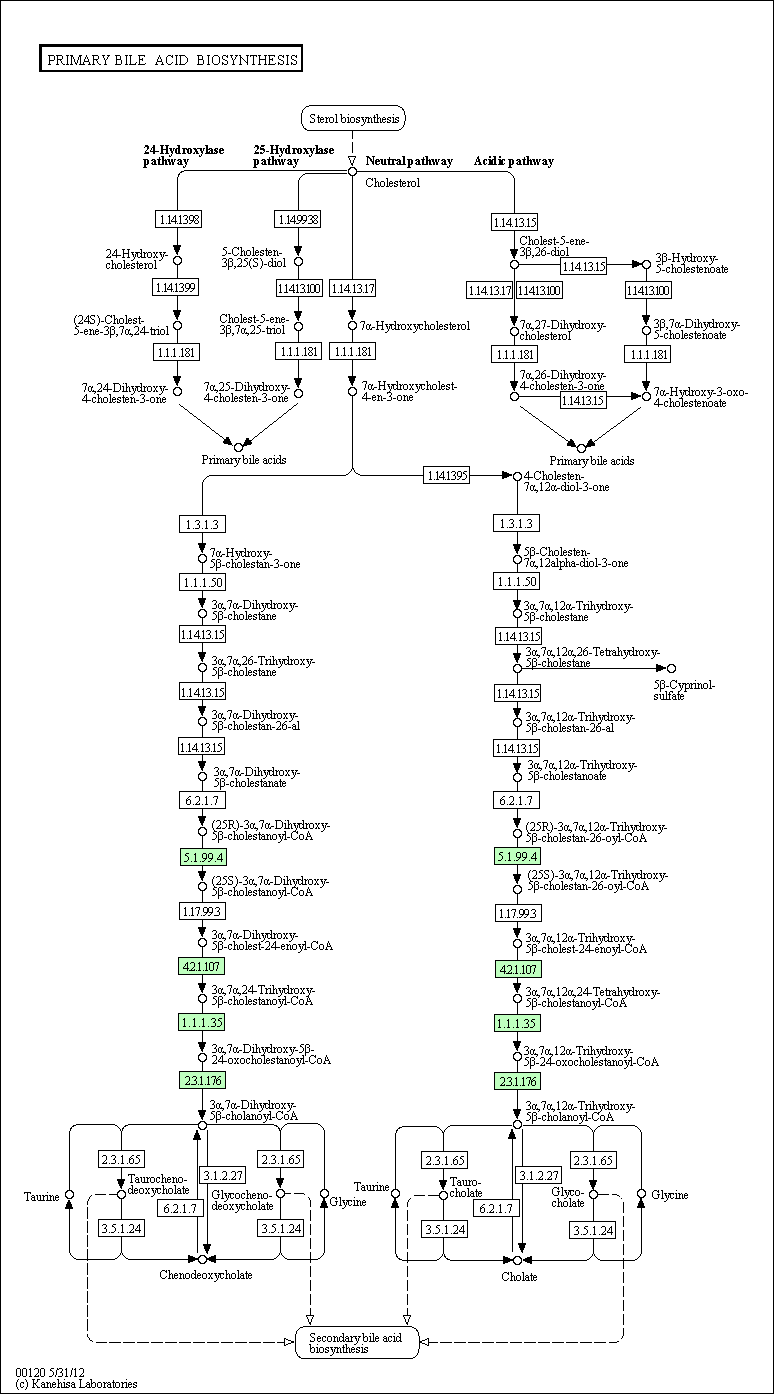

Supplement: Supplementary file 3 — Additional file 3: KEGG classification and functional maps of assembled contigs. Contigs annotated using KEGG Automatic Annotation Server identified sequences in a broad range of functional groups including developmental pathways and cell signaling. (ZIP 11 MB) [file 12864_2013_7026_MOESM3_ESM.zip › KEGG classification/map/map00120.png]

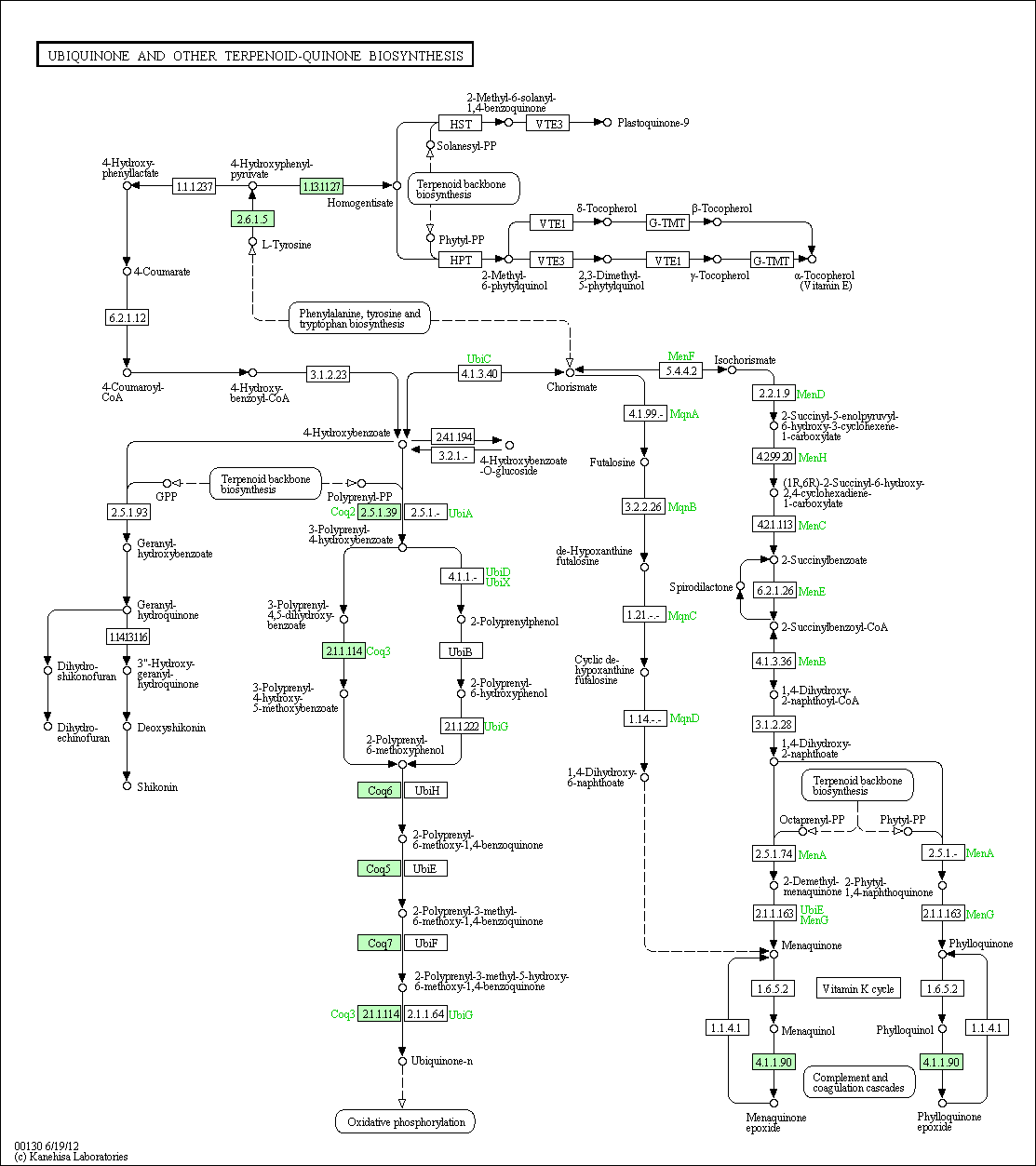

Supplement: Supplementary file 3 — Additional file 3: KEGG classification and functional maps of assembled contigs. Contigs annotated using KEGG Automatic Annotation Server identified sequences in a broad range of functional groups including developmental pathways and cell signaling. (ZIP 11 MB) [file 12864_2013_7026_MOESM3_ESM.zip › KEGG classification/map/map00130.png]

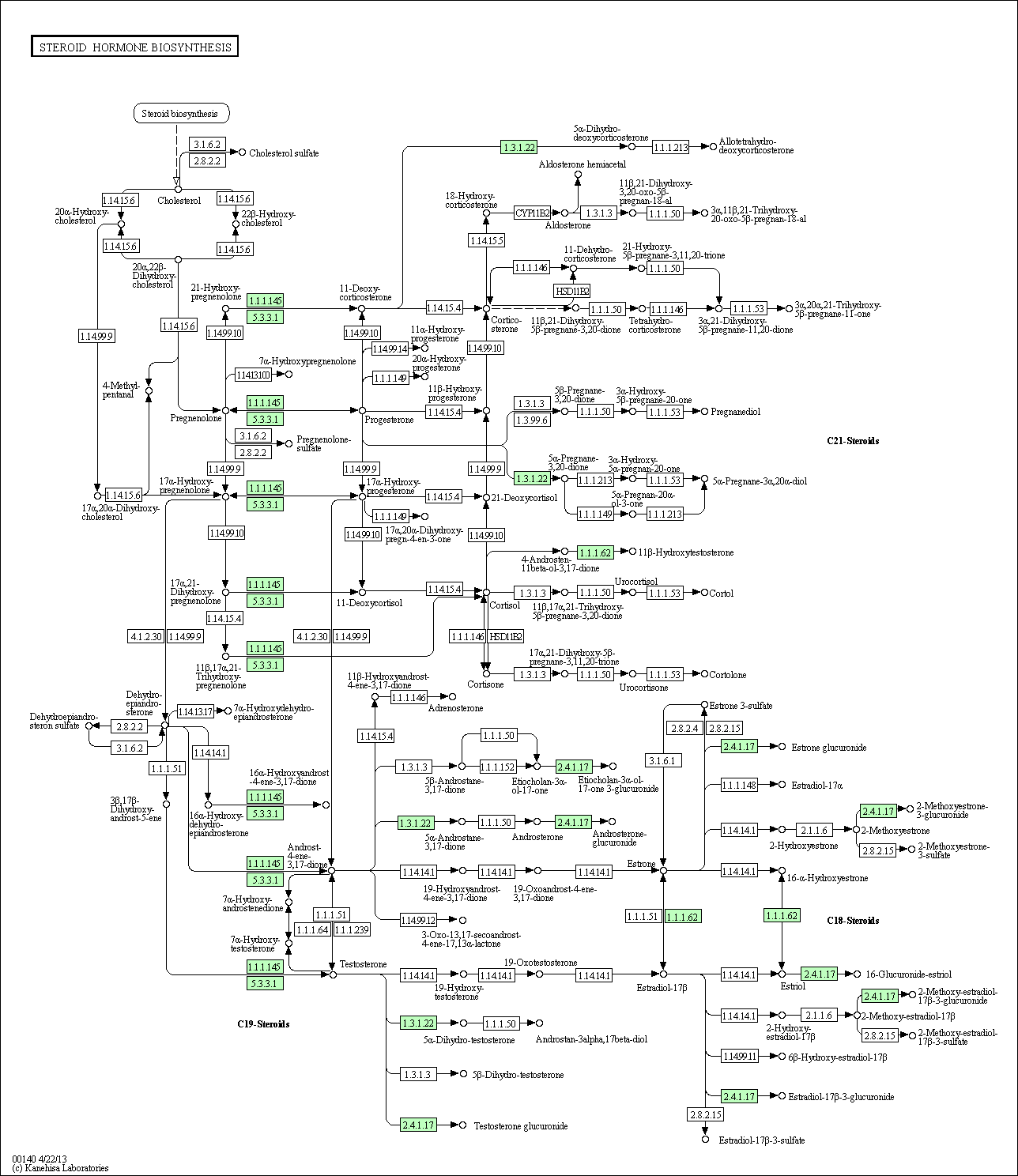

Supplement: Supplementary file 3 — Additional file 3: KEGG classification and functional maps of assembled contigs. Contigs annotated using KEGG Automatic Annotation Server identified sequences in a broad range of functional groups including developmental pathways and cell signaling. (ZIP 11 MB) [file 12864_2013_7026_MOESM3_ESM.zip › KEGG classification/map/map00140.png]

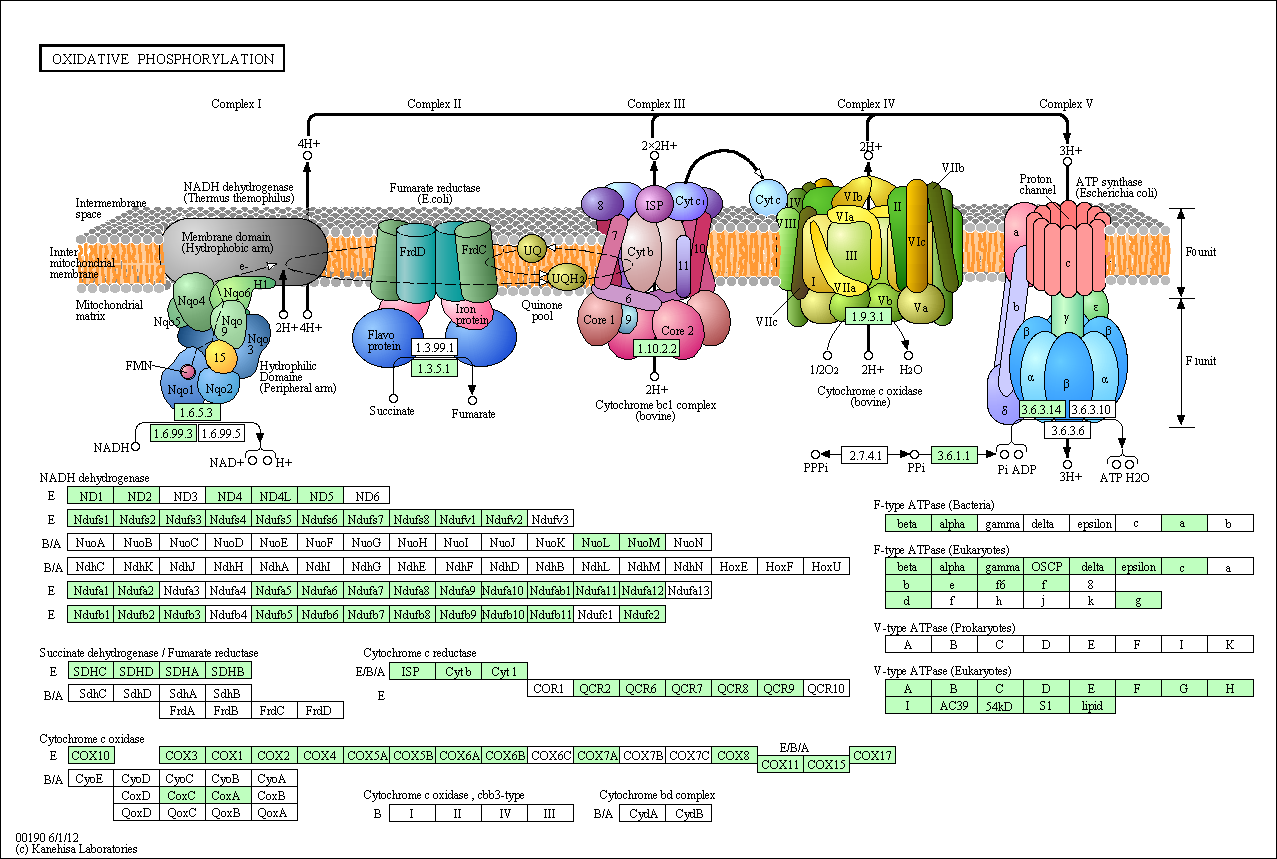

Supplement: Supplementary file 3 — Additional file 3: KEGG classification and functional maps of assembled contigs. Contigs annotated using KEGG Automatic Annotation Server identified sequences in a broad range of functional groups including developmental pathways and cell signaling. (ZIP 11 MB) [file 12864_2013_7026_MOESM3_ESM.zip › KEGG classification/map/map00190.png]

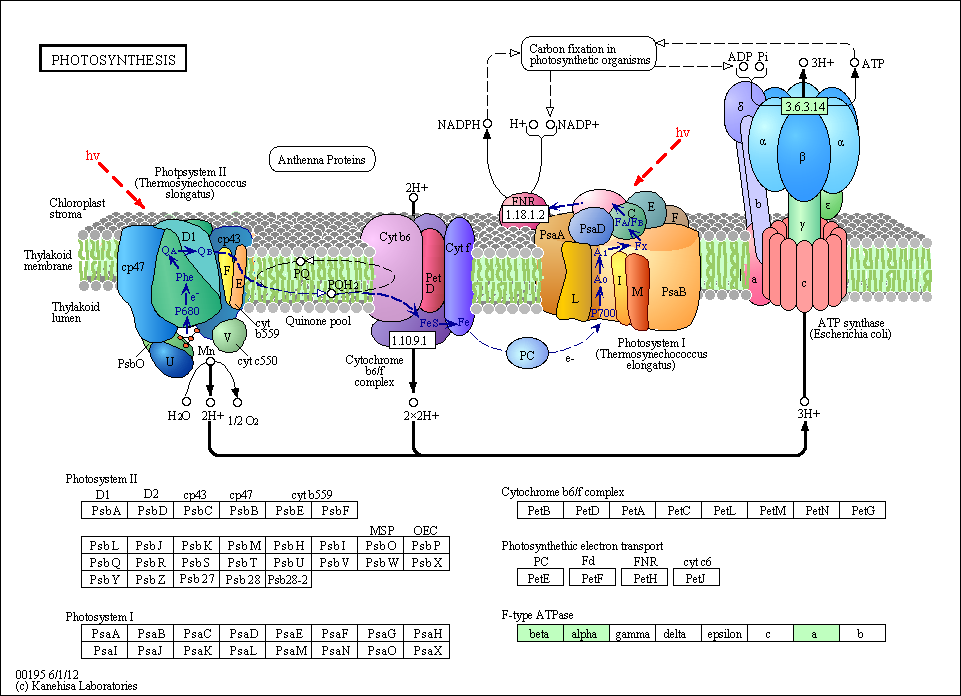

Supplement: Supplementary file 3 — Additional file 3: KEGG classification and functional maps of assembled contigs. Contigs annotated using KEGG Automatic Annotation Server identified sequences in a broad range of functional groups including developmental pathways and cell signaling. (ZIP 11 MB) [file 12864_2013_7026_MOESM3_ESM.zip › KEGG classification/map/map00195.png]

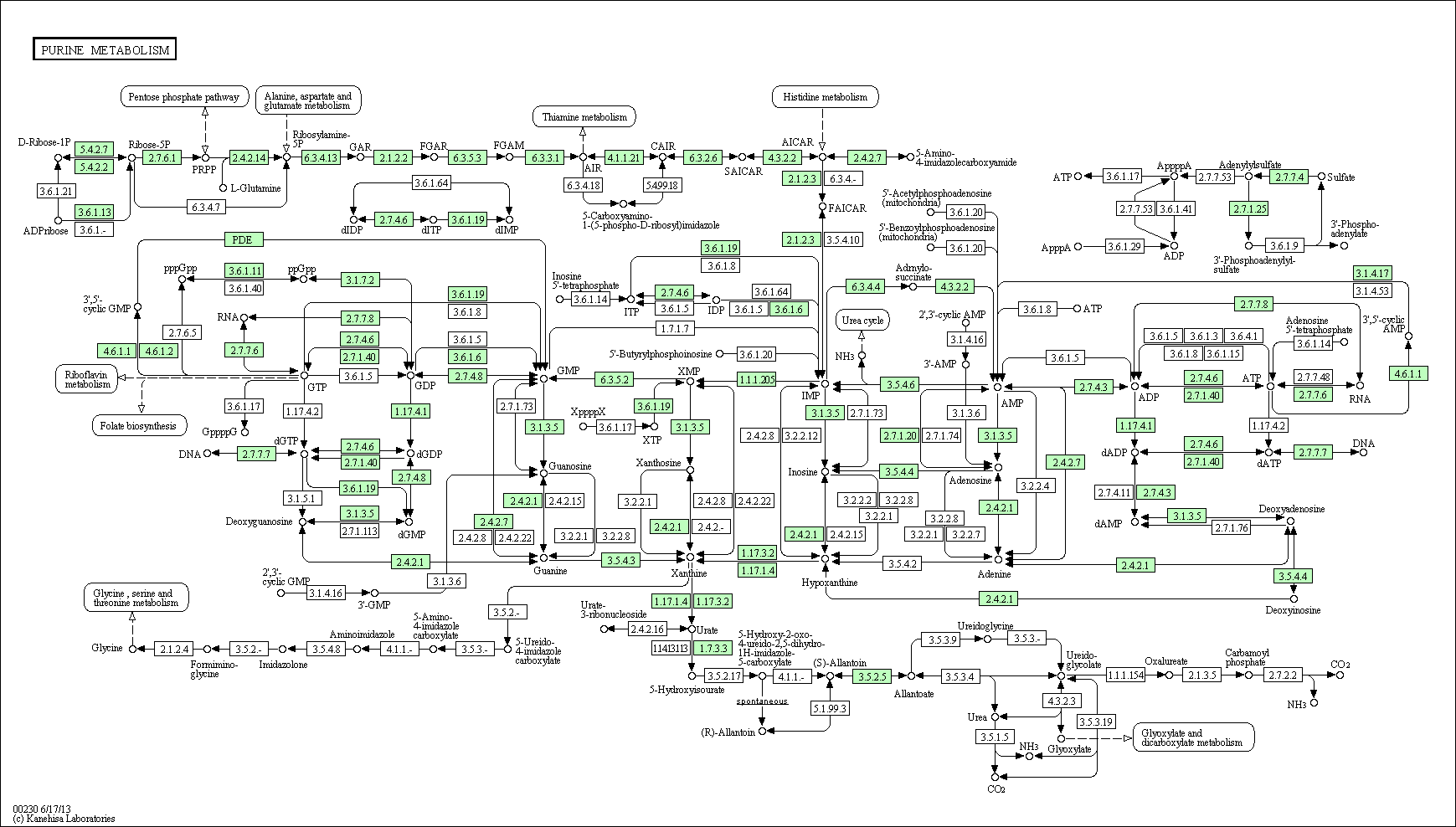

Supplement: Supplementary file 3 — Additional file 3: KEGG classification and functional maps of assembled contigs. Contigs annotated using KEGG Automatic Annotation Server identified sequences in a broad range of functional groups including developmental pathways and cell signaling. (ZIP 11 MB) [file 12864_2013_7026_MOESM3_ESM.zip › KEGG classification/map/map00230.png]

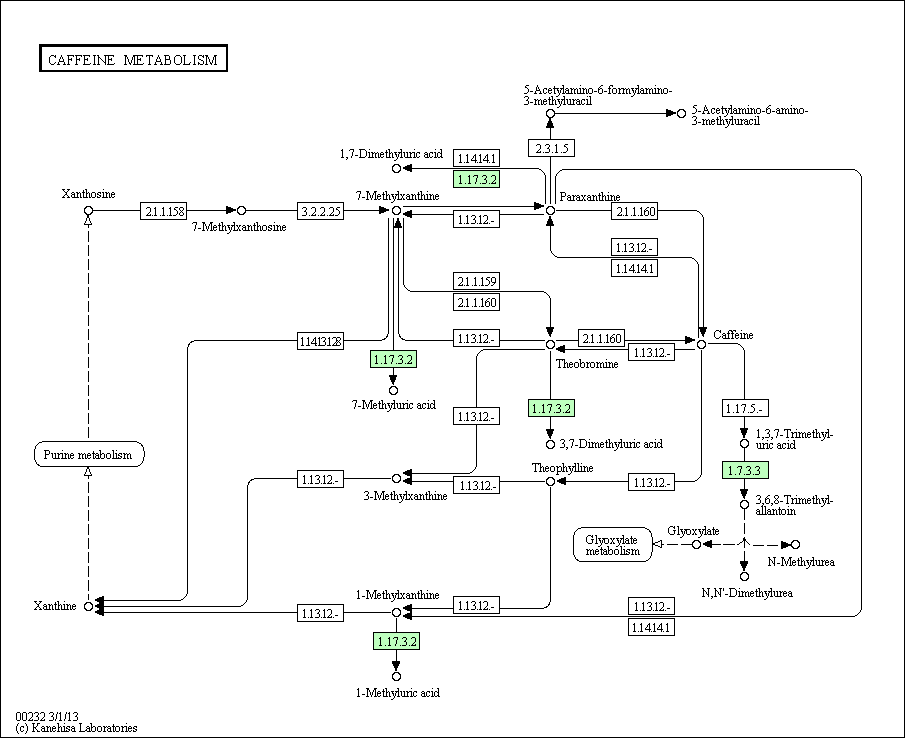

Supplement: Supplementary file 3 — Additional file 3: KEGG classification and functional maps of assembled contigs. Contigs annotated using KEGG Automatic Annotation Server identified sequences in a broad range of functional groups including developmental pathways and cell signaling. (ZIP 11 MB) [file 12864_2013_7026_MOESM3_ESM.zip › KEGG classification/map/map00232.png]

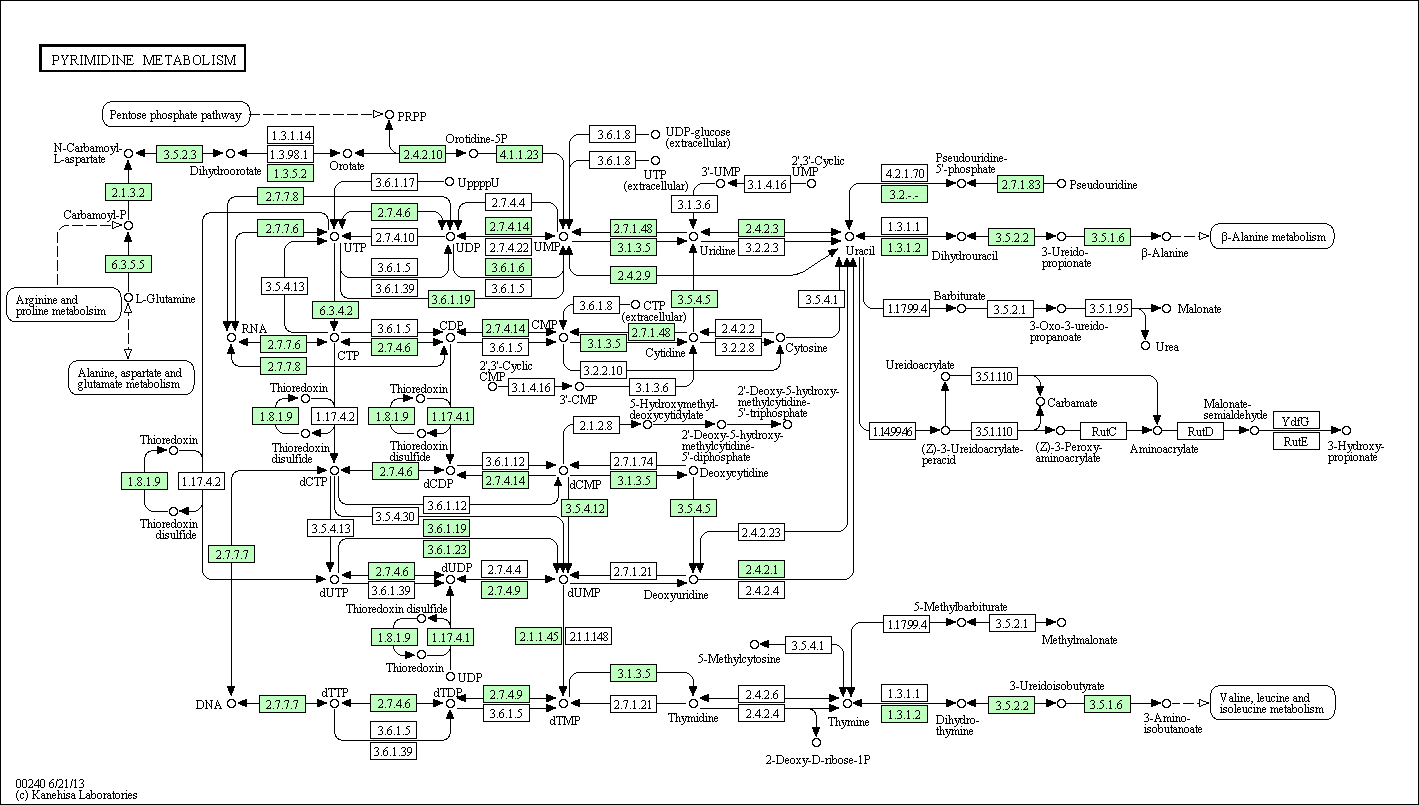

Supplement: Supplementary file 3 — Additional file 3: KEGG classification and functional maps of assembled contigs. Contigs annotated using KEGG Automatic Annotation Server identified sequences in a broad range of functional groups including developmental pathways and cell signaling. (ZIP 11 MB) [file 12864_2013_7026_MOESM3_ESM.zip › KEGG classification/map/map00240.png]

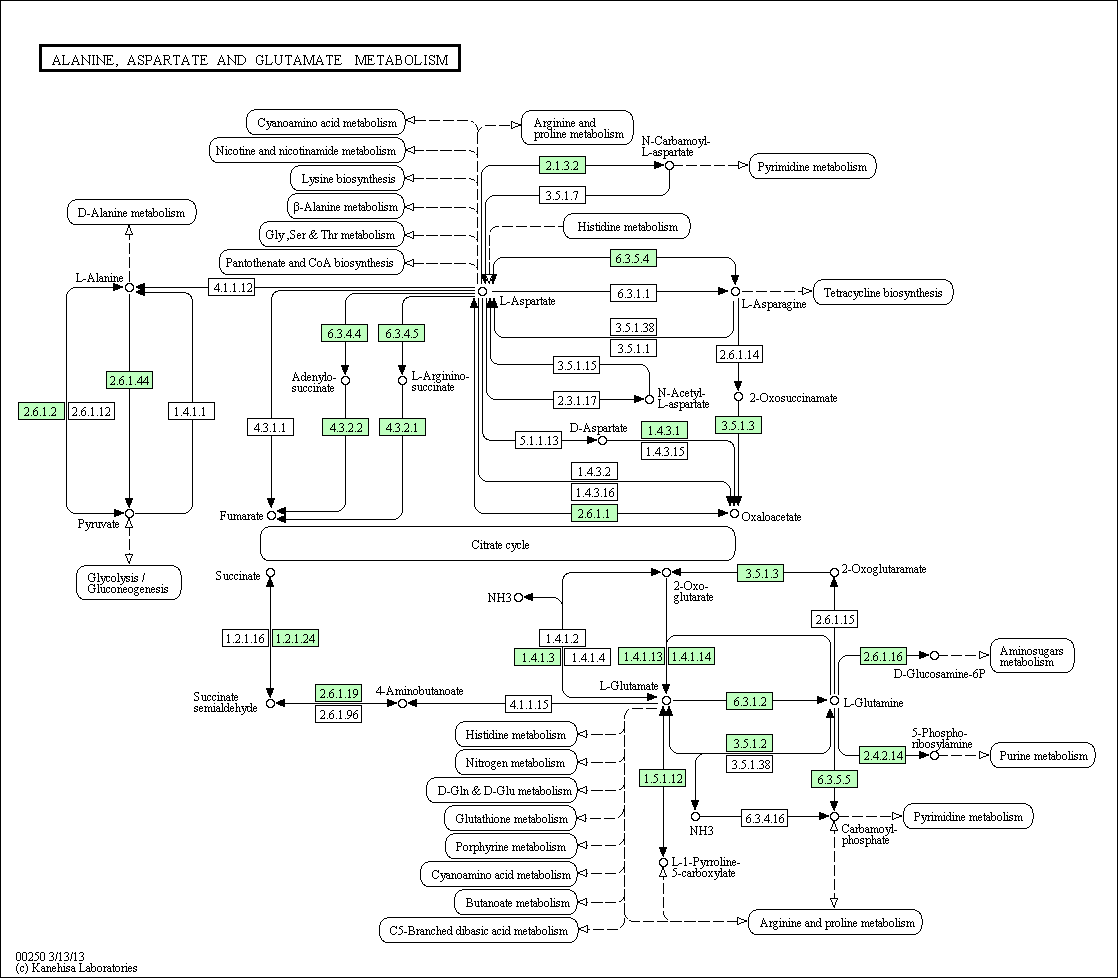

Supplement: Supplementary file 3 — Additional file 3: KEGG classification and functional maps of assembled contigs. Contigs annotated using KEGG Automatic Annotation Server identified sequences in a broad range of functional groups including developmental pathways and cell signaling. (ZIP 11 MB) [file 12864_2013_7026_MOESM3_ESM.zip › KEGG classification/map/map00250.png]

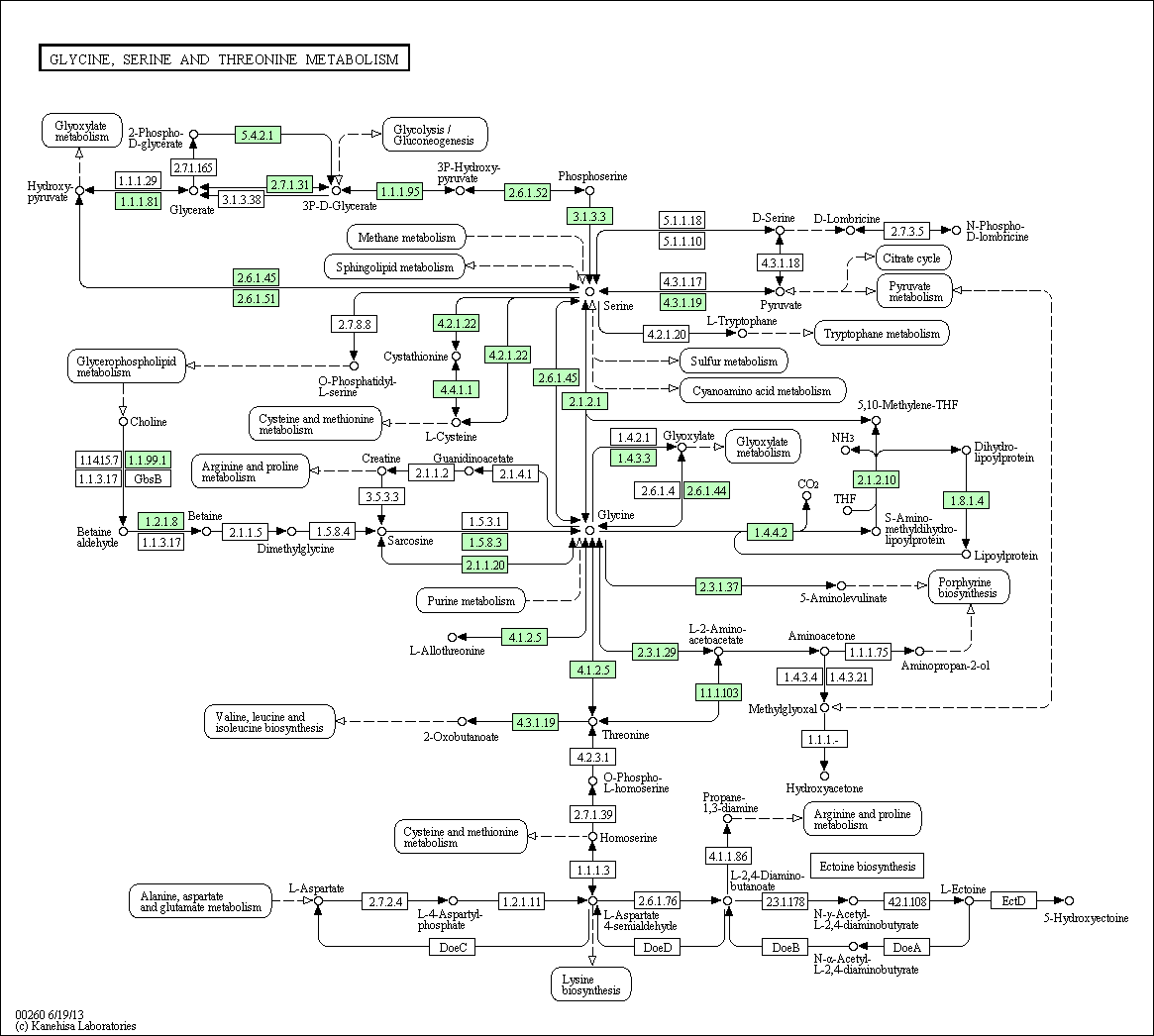

Supplement: Supplementary file 3 — Additional file 3: KEGG classification and functional maps of assembled contigs. Contigs annotated using KEGG Automatic Annotation Server identified sequences in a broad range of functional groups including developmental pathways and cell signaling. (ZIP 11 MB) [file 12864_2013_7026_MOESM3_ESM.zip › KEGG classification/map/map00260.png]

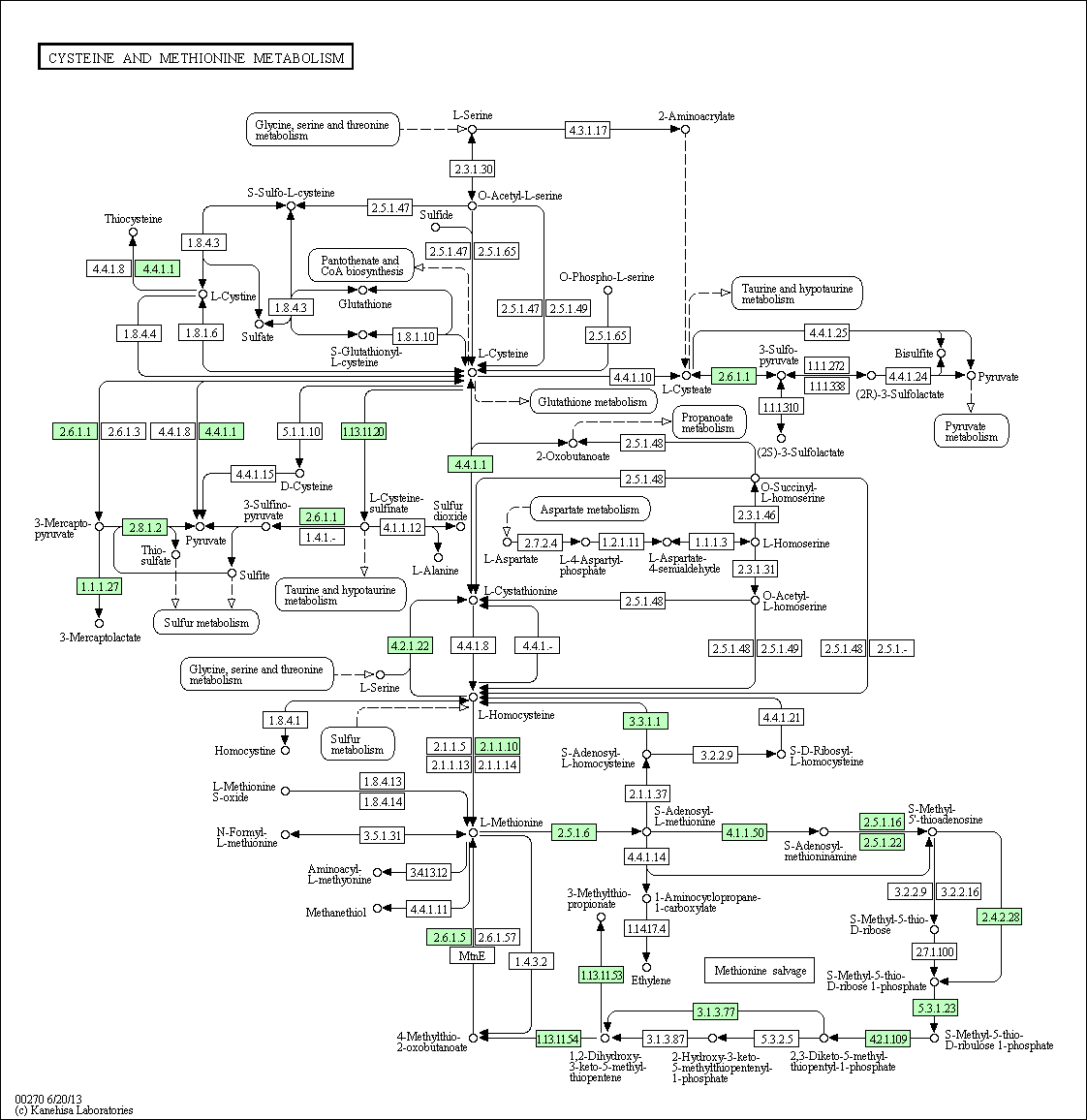

Supplement: Supplementary file 3 — Additional file 3: KEGG classification and functional maps of assembled contigs. Contigs annotated using KEGG Automatic Annotation Server identified sequences in a broad range of functional groups including developmental pathways and cell signaling. (ZIP 11 MB) [file 12864_2013_7026_MOESM3_ESM.zip › KEGG classification/map/map00270.png]

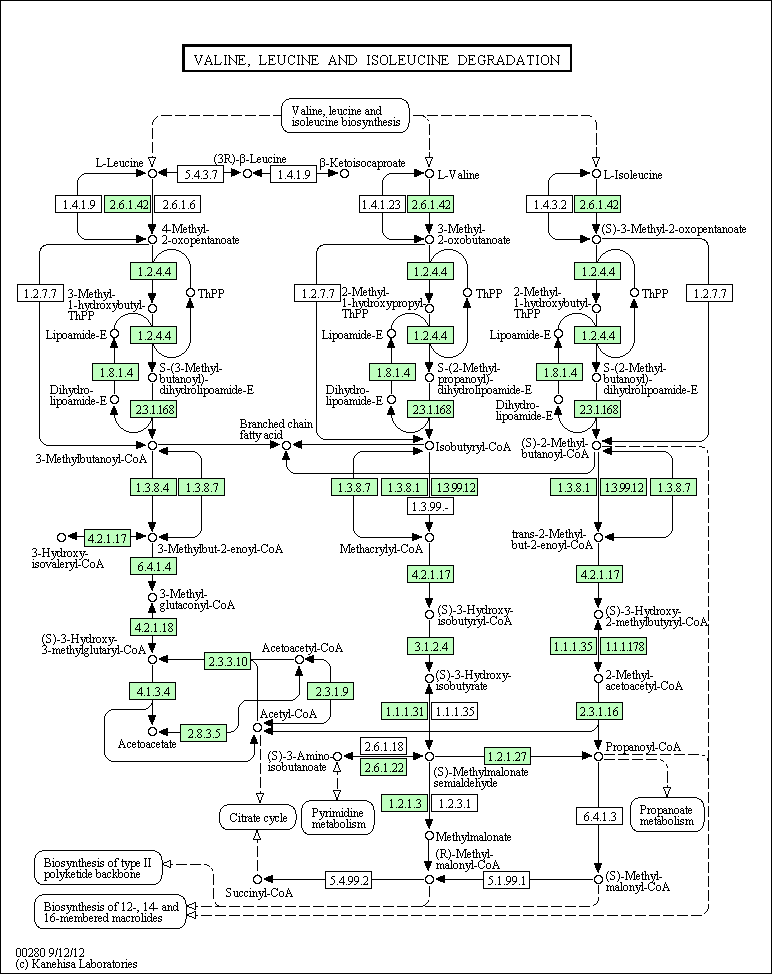

Supplement: Supplementary file 3 — Additional file 3: KEGG classification and functional maps of assembled contigs. Contigs annotated using KEGG Automatic Annotation Server identified sequences in a broad range of functional groups including developmental pathways and cell signaling. (ZIP 11 MB) [file 12864_2013_7026_MOESM3_ESM.zip › KEGG classification/map/map00280.png]

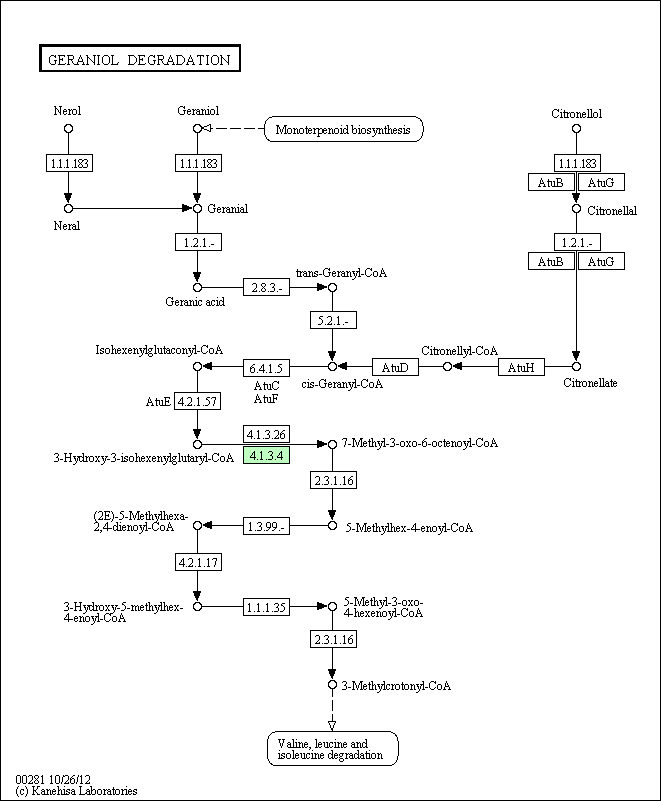

Supplement: Supplementary file 3 — Additional file 3: KEGG classification and functional maps of assembled contigs. Contigs annotated using KEGG Automatic Annotation Server identified sequences in a broad range of functional groups including developmental pathways and cell signaling. (ZIP 11 MB) [file 12864_2013_7026_MOESM3_ESM.zip › KEGG classification/map/map00281.png]

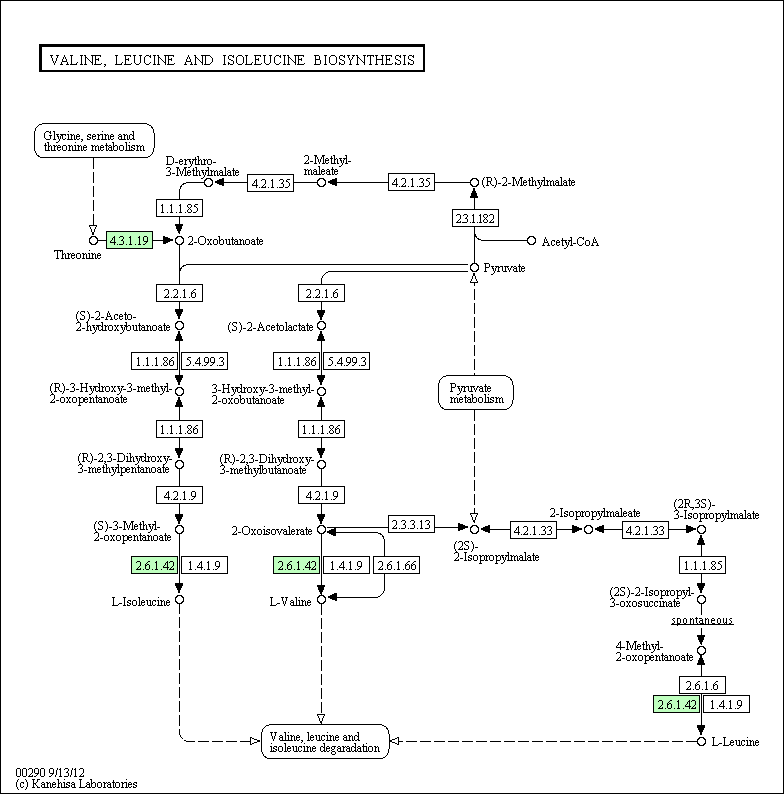

Supplement: Supplementary file 3 — Additional file 3: KEGG classification and functional maps of assembled contigs. Contigs annotated using KEGG Automatic Annotation Server identified sequences in a broad range of functional groups including developmental pathways and cell signaling. (ZIP 11 MB) [file 12864_2013_7026_MOESM3_ESM.zip › KEGG classification/map/map00290.png]

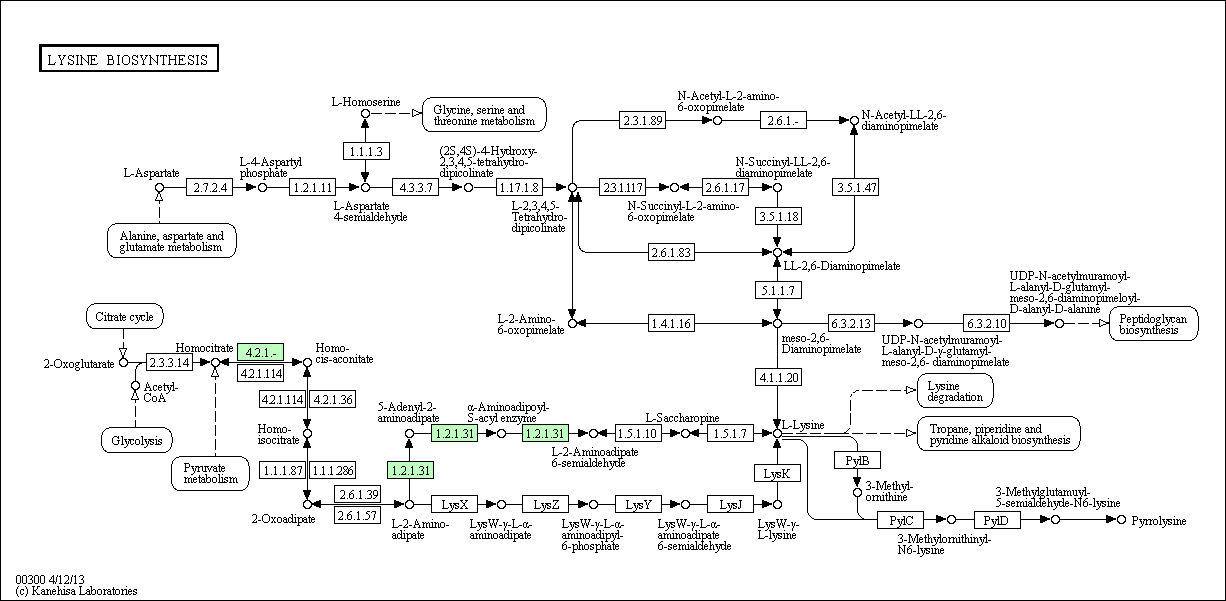

Supplement: Supplementary file 3 — Additional file 3: KEGG classification and functional maps of assembled contigs. Contigs annotated using KEGG Automatic Annotation Server identified sequences in a broad range of functional groups including developmental pathways and cell signaling. (ZIP 11 MB) [file 12864_2013_7026_MOESM3_ESM.zip › KEGG classification/map/map00300.png]

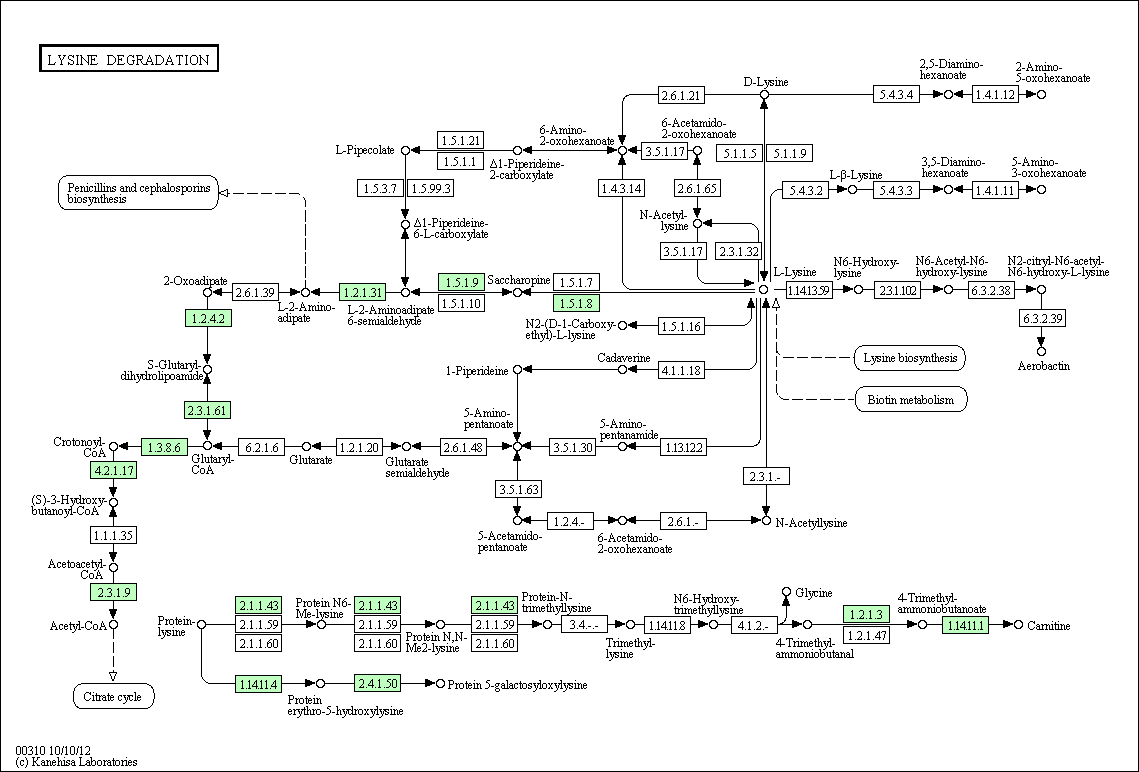

Supplement: Supplementary file 3 — Additional file 3: KEGG classification and functional maps of assembled contigs. Contigs annotated using KEGG Automatic Annotation Server identified sequences in a broad range of functional groups including developmental pathways and cell signaling. (ZIP 11 MB) [file 12864_2013_7026_MOESM3_ESM.zip › KEGG classification/map/map00310.png]

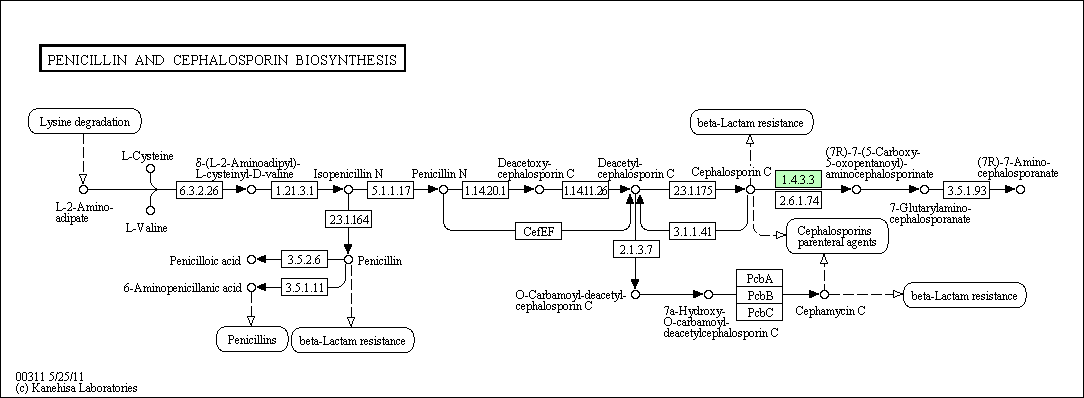

Supplement: Supplementary file 3 — Additional file 3: KEGG classification and functional maps of assembled contigs. Contigs annotated using KEGG Automatic Annotation Server identified sequences in a broad range of functional groups including developmental pathways and cell signaling. (ZIP 11 MB) [file 12864_2013_7026_MOESM3_ESM.zip › KEGG classification/map/map00311.png]

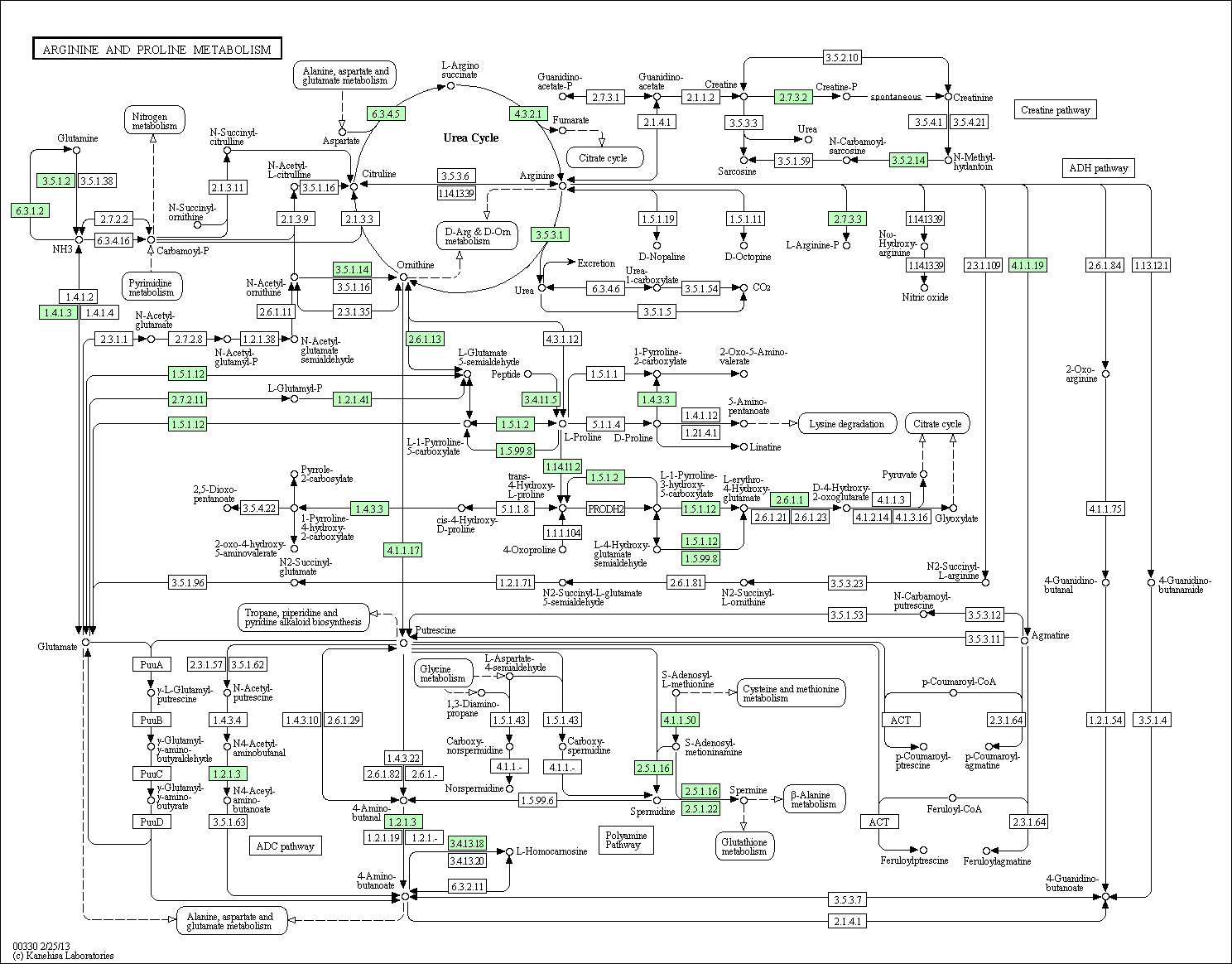

Supplement: Supplementary file 3 — Additional file 3: KEGG classification and functional maps of assembled contigs. Contigs annotated using KEGG Automatic Annotation Server identified sequences in a broad range of functional groups including developmental pathways and cell signaling. (ZIP 11 MB) [file 12864_2013_7026_MOESM3_ESM.zip › KEGG classification/map/map00330.png]

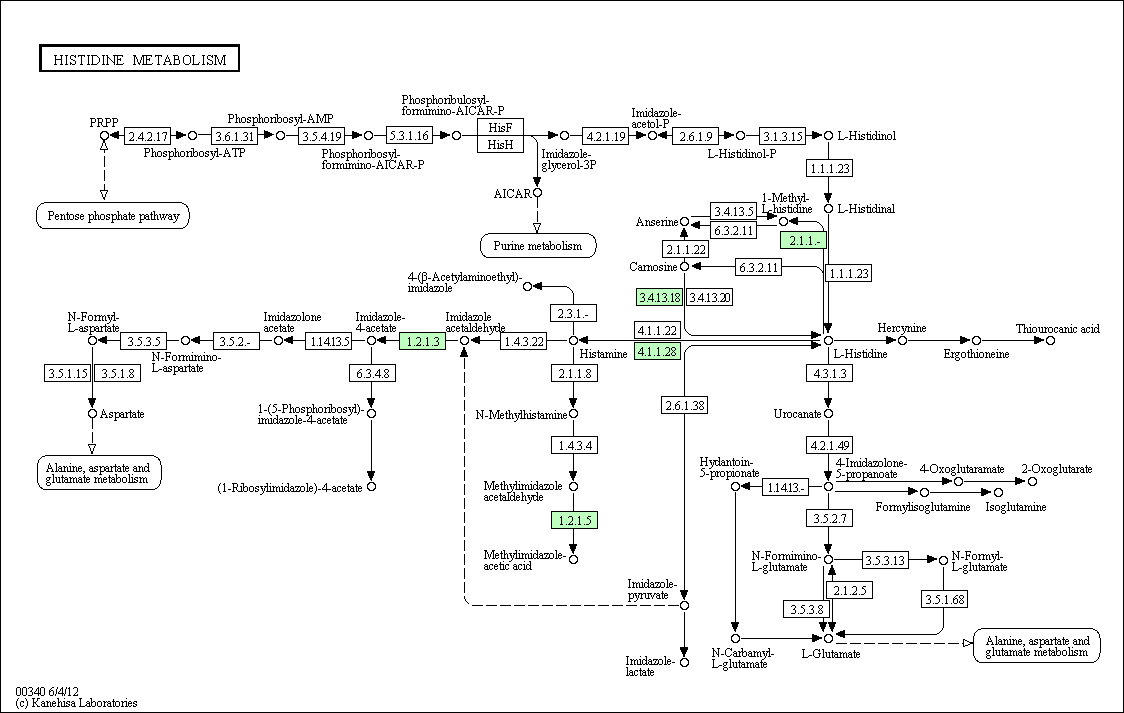

Supplement: Supplementary file 3 — Additional file 3: KEGG classification and functional maps of assembled contigs. Contigs annotated using KEGG Automatic Annotation Server identified sequences in a broad range of functional groups including developmental pathways and cell signaling. (ZIP 11 MB) [file 12864_2013_7026_MOESM3_ESM.zip › KEGG classification/map/map00340.png]

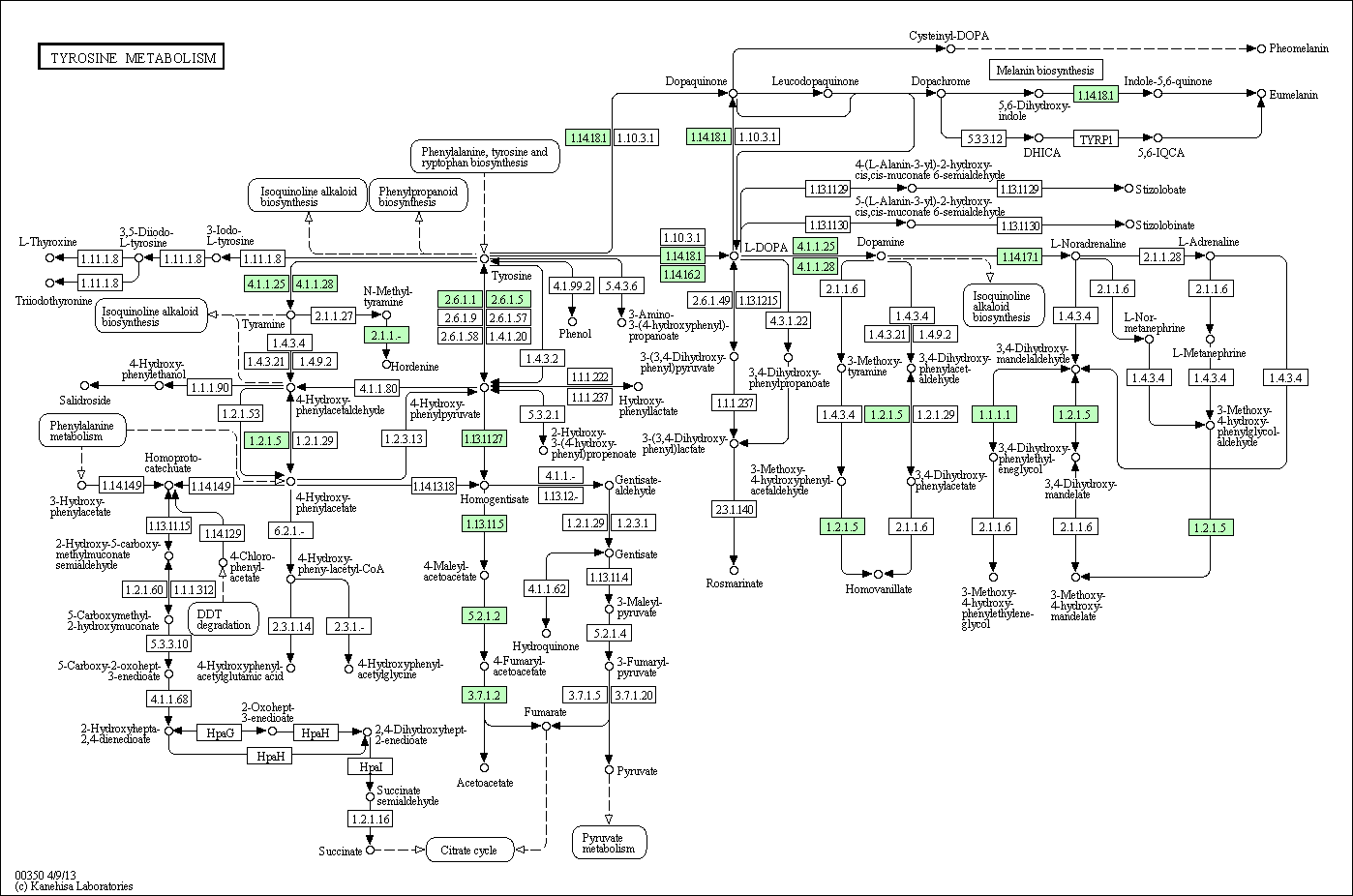

Supplement: Supplementary file 3 — Additional file 3: KEGG classification and functional maps of assembled contigs. Contigs annotated using KEGG Automatic Annotation Server identified sequences in a broad range of functional groups including developmental pathways and cell signaling. (ZIP 11 MB) [file 12864_2013_7026_MOESM3_ESM.zip › KEGG classification/map/map00350.png]

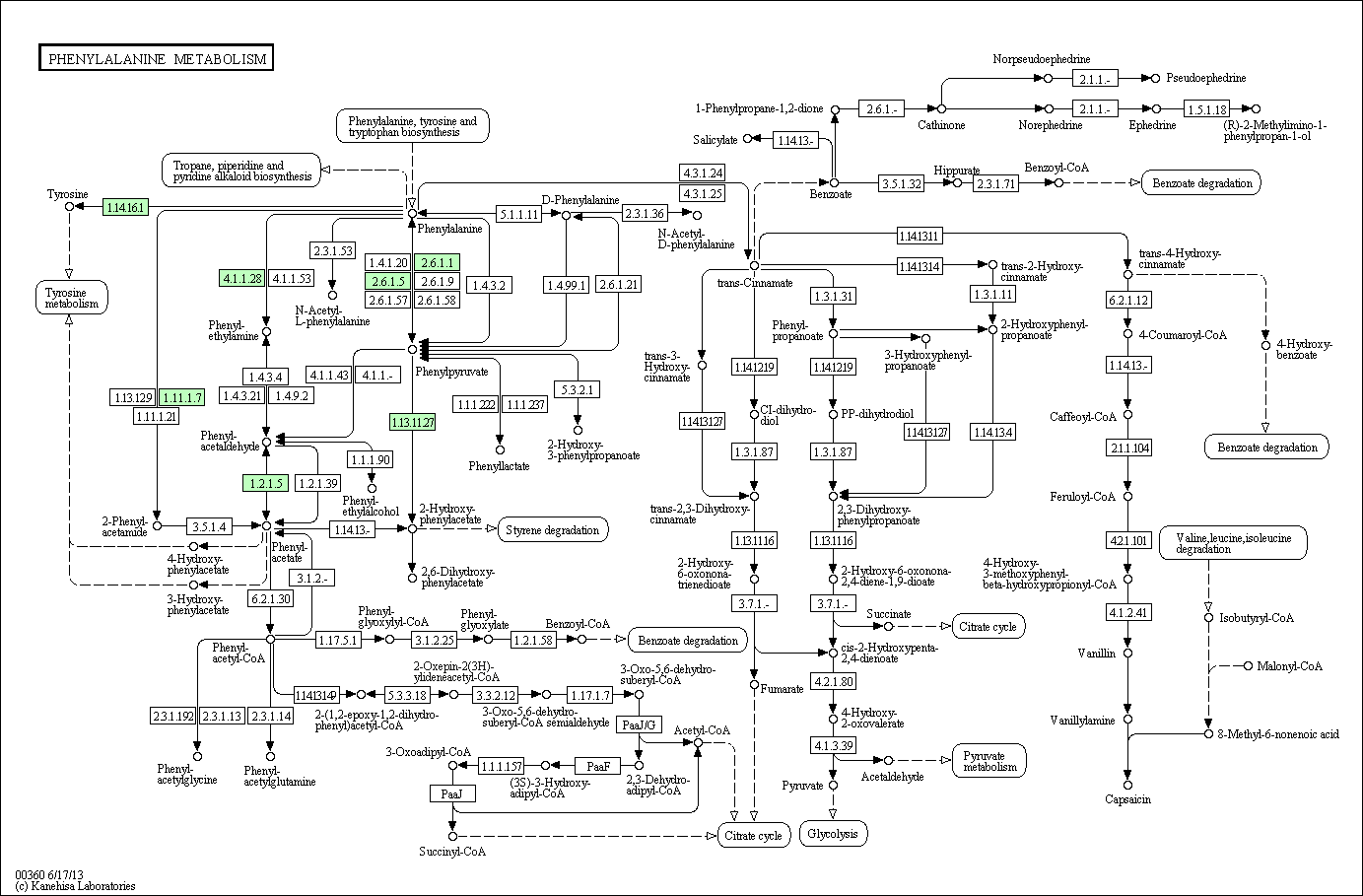

Supplement: Supplementary file 3 — Additional file 3: KEGG classification and functional maps of assembled contigs. Contigs annotated using KEGG Automatic Annotation Server identified sequences in a broad range of functional groups including developmental pathways and cell signaling. (ZIP 11 MB) [file 12864_2013_7026_MOESM3_ESM.zip › KEGG classification/map/map00360.png]

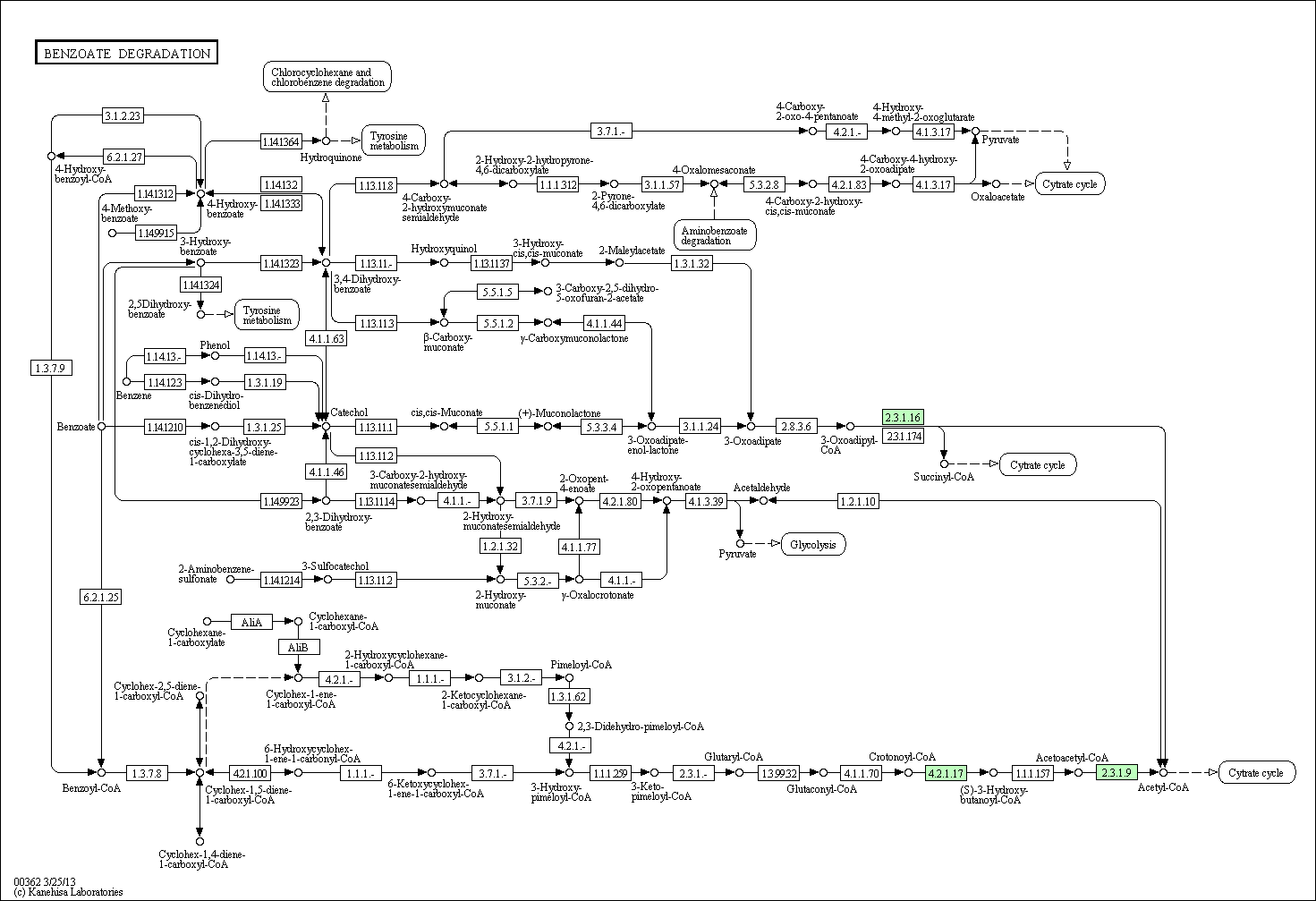

Supplement: Supplementary file 3 — Additional file 3: KEGG classification and functional maps of assembled contigs. Contigs annotated using KEGG Automatic Annotation Server identified sequences in a broad range of functional groups including developmental pathways and cell signaling. (ZIP 11 MB) [file 12864_2013_7026_MOESM3_ESM.zip › KEGG classification/map/map00362.png]

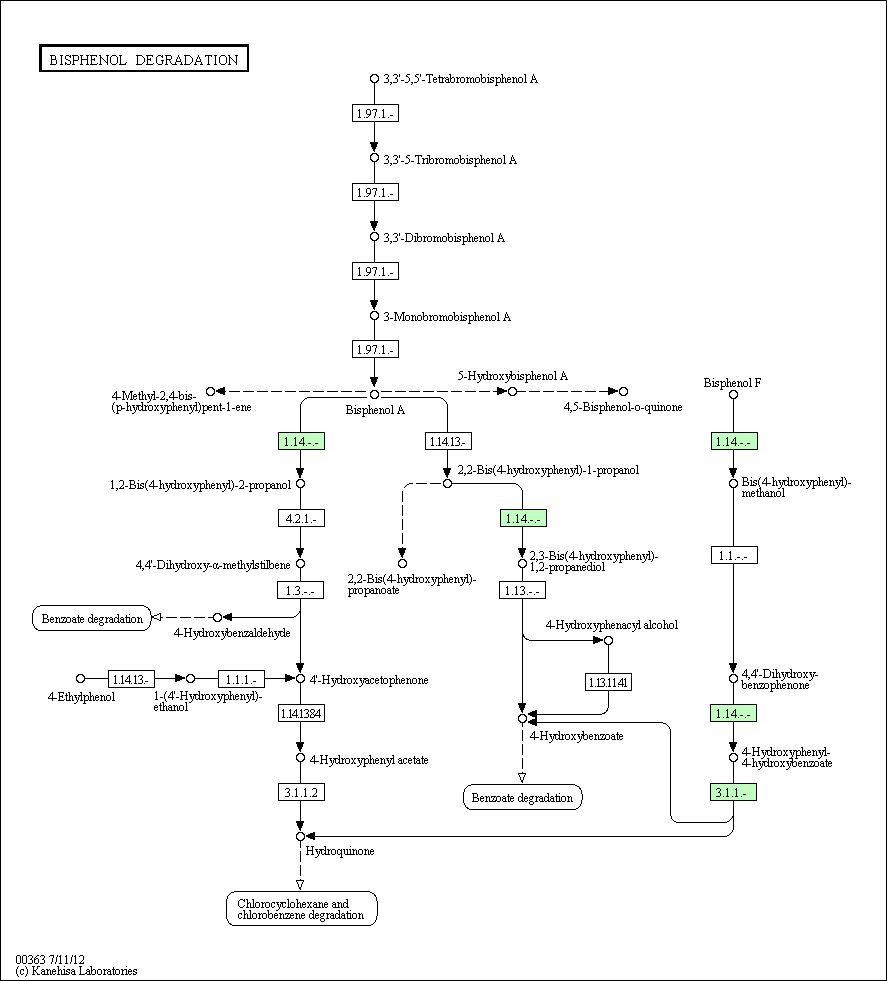

Supplement: Supplementary file 3 — Additional file 3: KEGG classification and functional maps of assembled contigs. Contigs annotated using KEGG Automatic Annotation Server identified sequences in a broad range of functional groups including developmental pathways and cell signaling. (ZIP 11 MB) [file 12864_2013_7026_MOESM3_ESM.zip › KEGG classification/map/map00363.png]

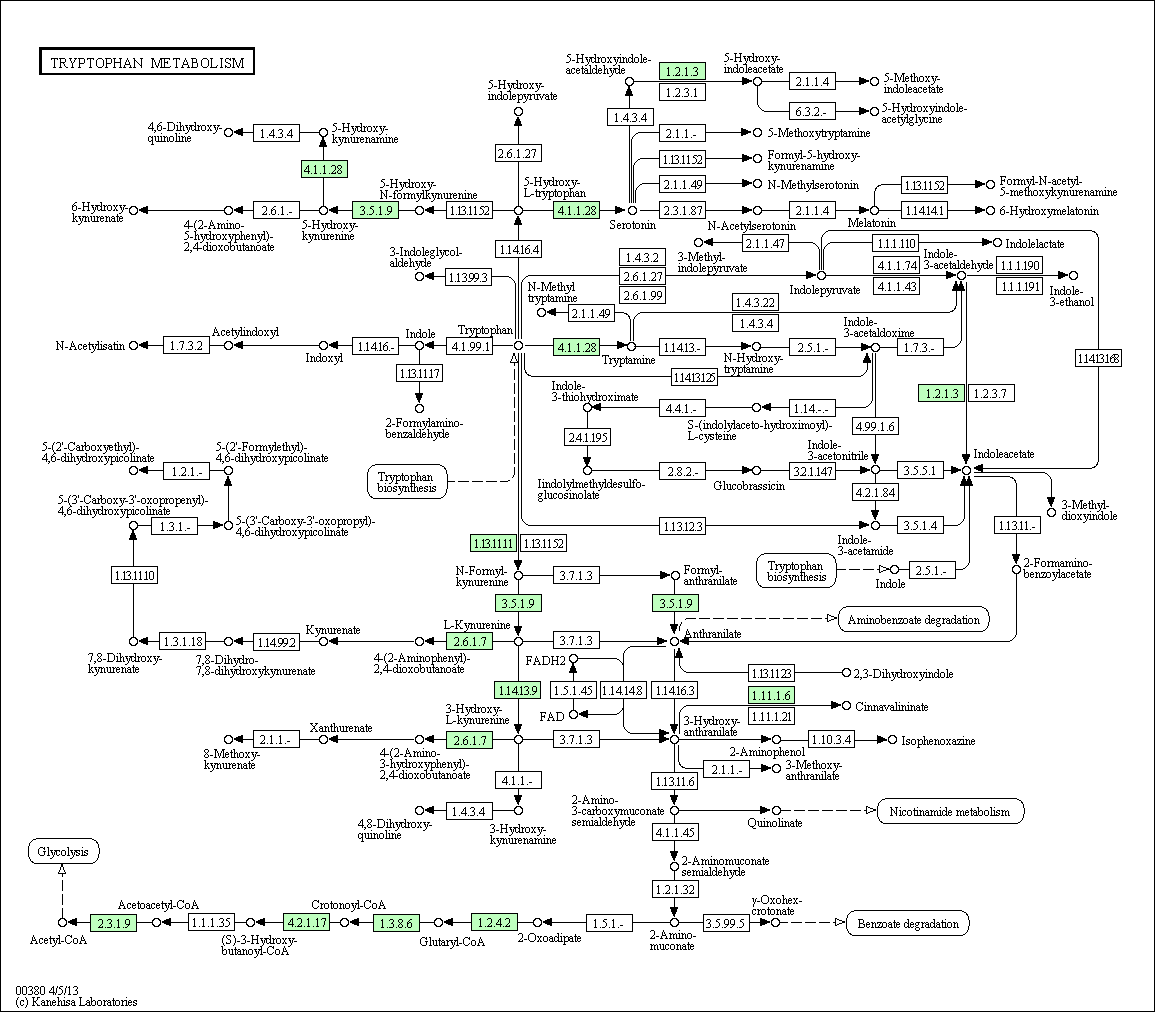

Supplement: Supplementary file 3 — Additional file 3: KEGG classification and functional maps of assembled contigs. Contigs annotated using KEGG Automatic Annotation Server identified sequences in a broad range of functional groups including developmental pathways and cell signaling. (ZIP 11 MB) [file 12864_2013_7026_MOESM3_ESM.zip › KEGG classification/map/map00380.png]

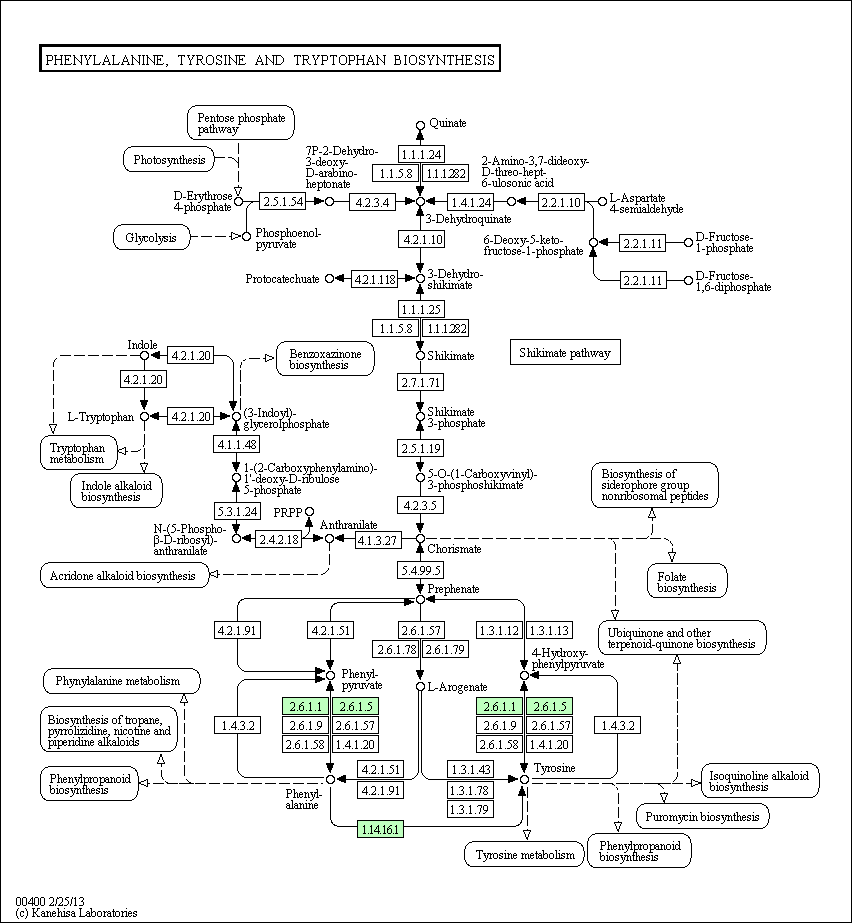

Supplement: Supplementary file 3 — Additional file 3: KEGG classification and functional maps of assembled contigs. Contigs annotated using KEGG Automatic Annotation Server identified sequences in a broad range of functional groups including developmental pathways and cell signaling. (ZIP 11 MB) [file 12864_2013_7026_MOESM3_ESM.zip › KEGG classification/map/map00400.png]

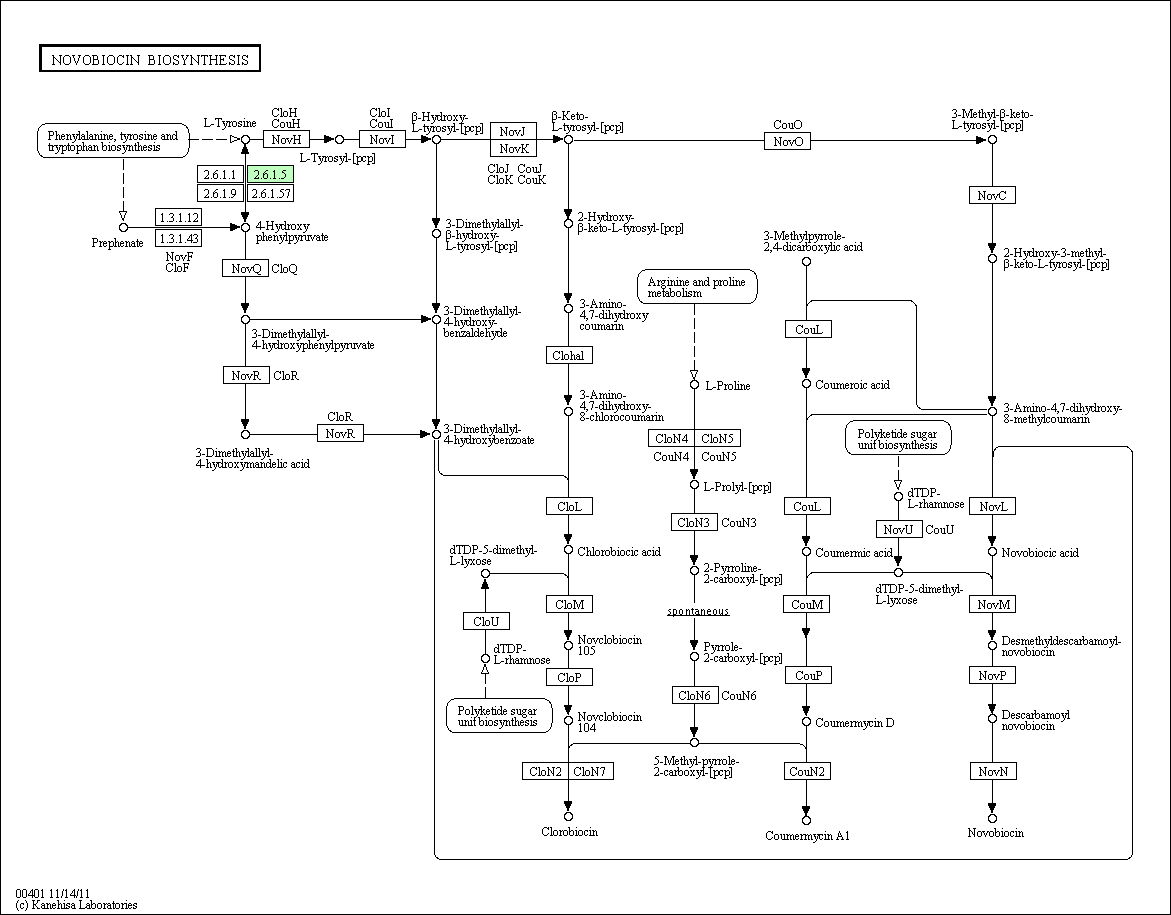

Supplement: Supplementary file 3 — Additional file 3: KEGG classification and functional maps of assembled contigs. Contigs annotated using KEGG Automatic Annotation Server identified sequences in a broad range of functional groups including developmental pathways and cell signaling. (ZIP 11 MB) [file 12864_2013_7026_MOESM3_ESM.zip › KEGG classification/map/map00401.png]

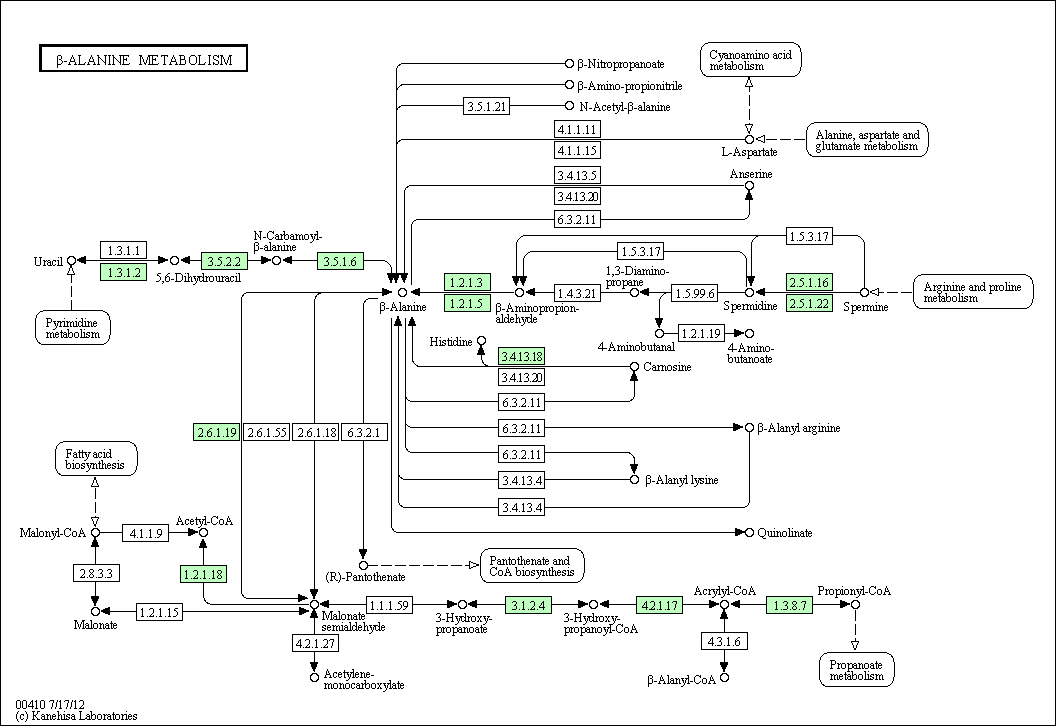

Supplement: Supplementary file 3 — Additional file 3: KEGG classification and functional maps of assembled contigs. Contigs annotated using KEGG Automatic Annotation Server identified sequences in a broad range of functional groups including developmental pathways and cell signaling. (ZIP 11 MB) [file 12864_2013_7026_MOESM3_ESM.zip › KEGG classification/map/map00410.png]

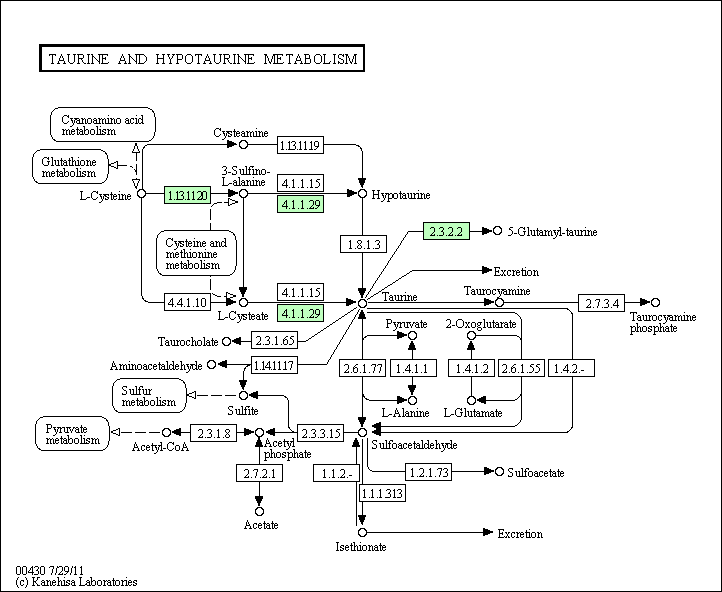

Supplement: Supplementary file 3 — Additional file 3: KEGG classification and functional maps of assembled contigs. Contigs annotated using KEGG Automatic Annotation Server identified sequences in a broad range of functional groups including developmental pathways and cell signaling. (ZIP 11 MB) [file 12864_2013_7026_MOESM3_ESM.zip › KEGG classification/map/map00430.png]

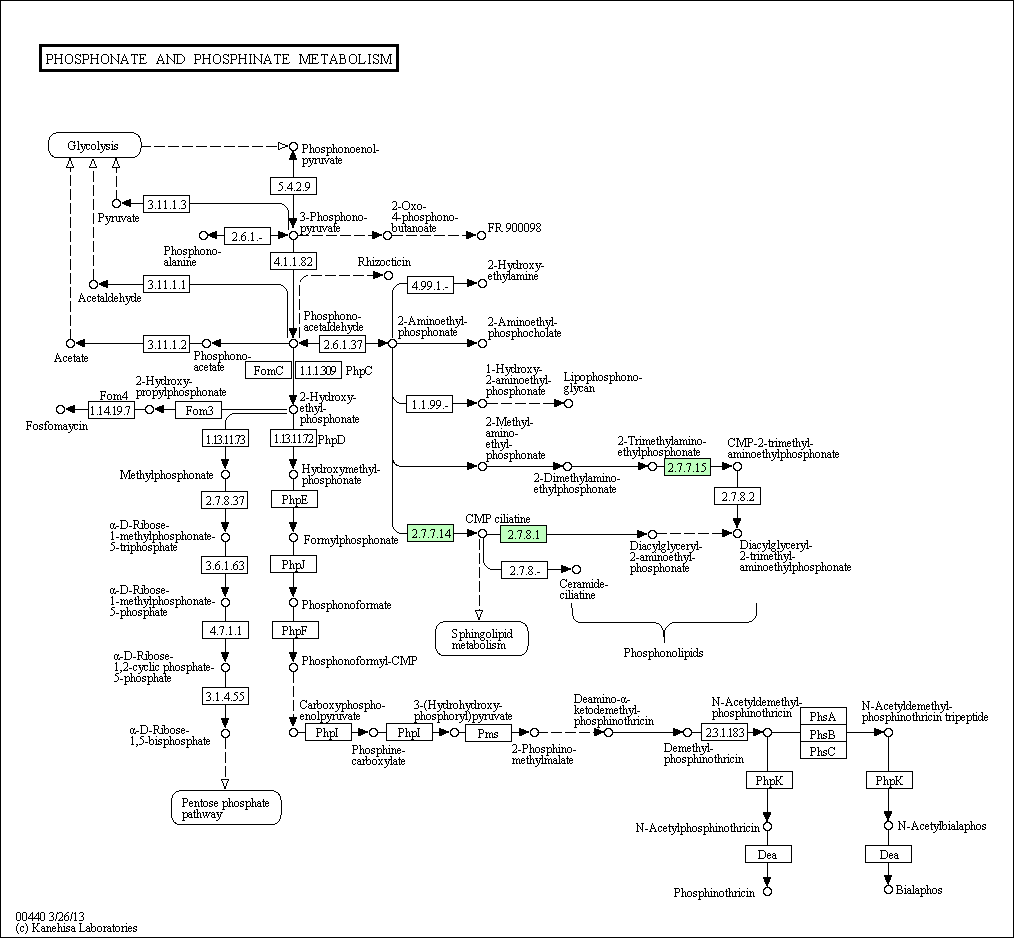

Supplement: Supplementary file 3 — Additional file 3: KEGG classification and functional maps of assembled contigs. Contigs annotated using KEGG Automatic Annotation Server identified sequences in a broad range of functional groups including developmental pathways and cell signaling. (ZIP 11 MB) [file 12864_2013_7026_MOESM3_ESM.zip › KEGG classification/map/map00440.png]

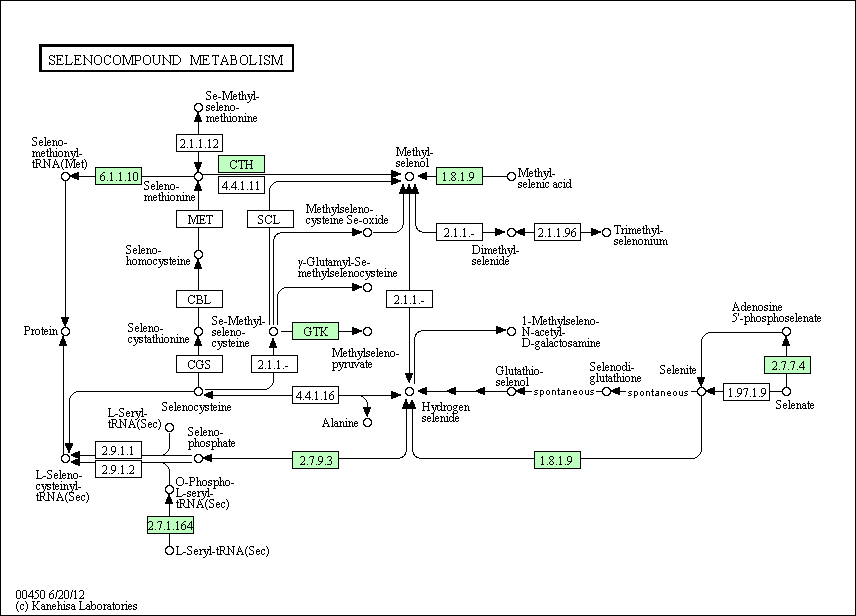

Supplement: Supplementary file 3 — Additional file 3: KEGG classification and functional maps of assembled contigs. Contigs annotated using KEGG Automatic Annotation Server identified sequences in a broad range of functional groups including developmental pathways and cell signaling. (ZIP 11 MB) [file 12864_2013_7026_MOESM3_ESM.zip › KEGG classification/map/map00450.png]

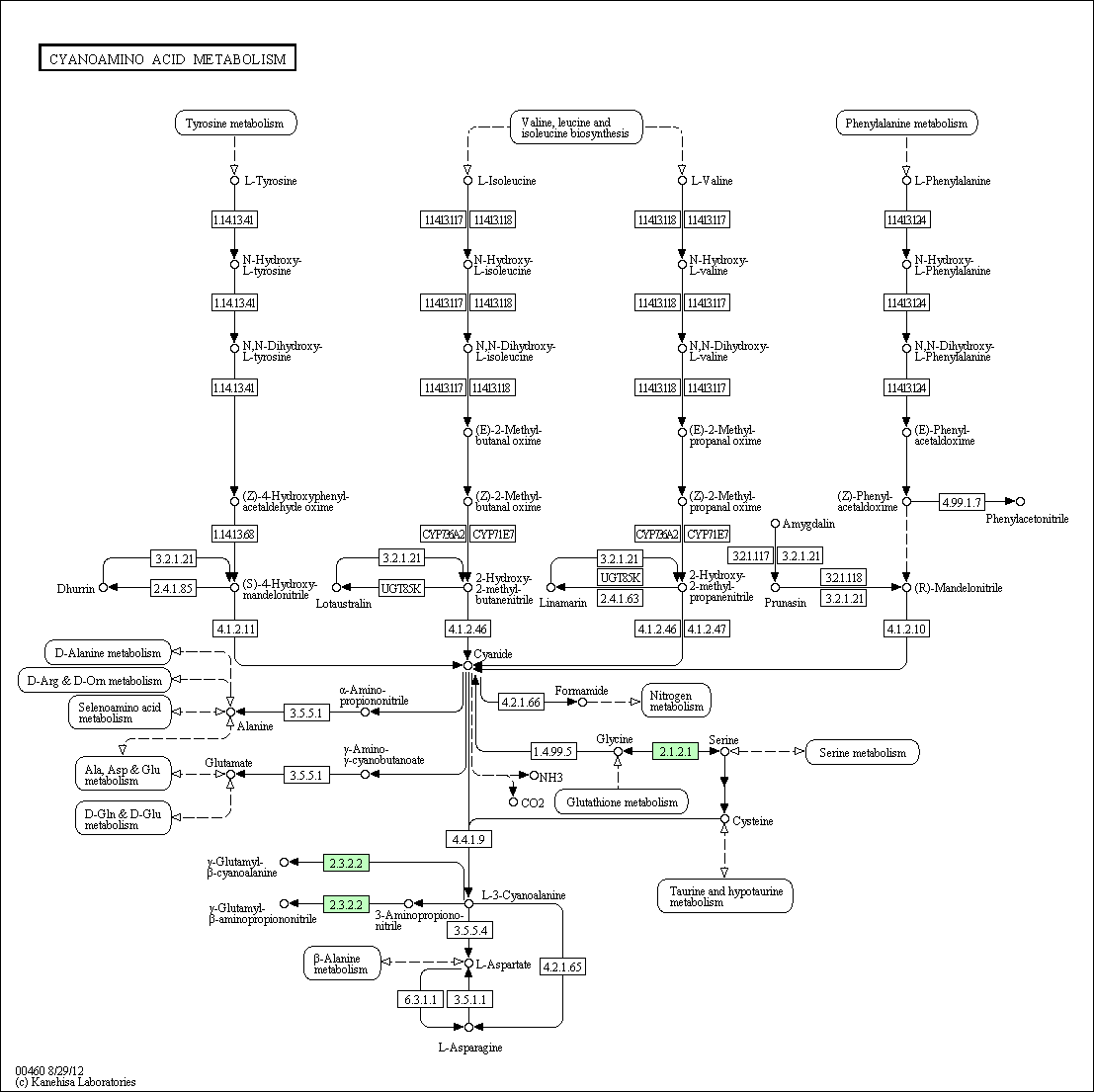

Supplement: Supplementary file 3 — Additional file 3: KEGG classification and functional maps of assembled contigs. Contigs annotated using KEGG Automatic Annotation Server identified sequences in a broad range of functional groups including developmental pathways and cell signaling. (ZIP 11 MB) [file 12864_2013_7026_MOESM3_ESM.zip › KEGG classification/map/map00460.png]

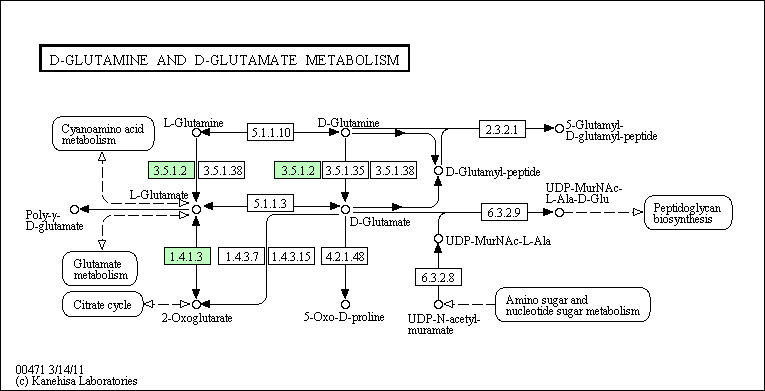

Supplement: Supplementary file 3 — Additional file 3: KEGG classification and functional maps of assembled contigs. Contigs annotated using KEGG Automatic Annotation Server identified sequences in a broad range of functional groups including developmental pathways and cell signaling. (ZIP 11 MB) [file 12864_2013_7026_MOESM3_ESM.zip › KEGG classification/map/map00471.png]

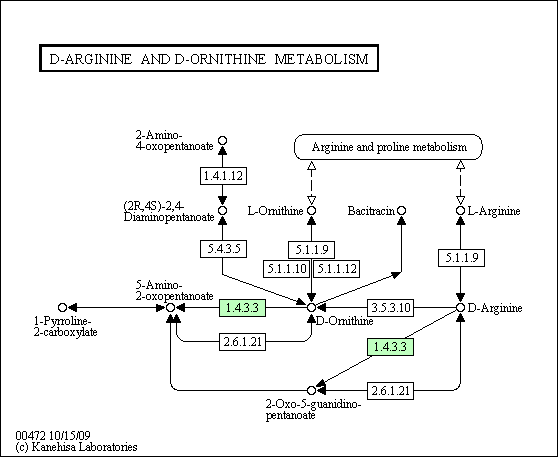

Supplement: Supplementary file 3 — Additional file 3: KEGG classification and functional maps of assembled contigs. Contigs annotated using KEGG Automatic Annotation Server identified sequences in a broad range of functional groups including developmental pathways and cell signaling. (ZIP 11 MB) [file 12864_2013_7026_MOESM3_ESM.zip › KEGG classification/map/map00472.png]

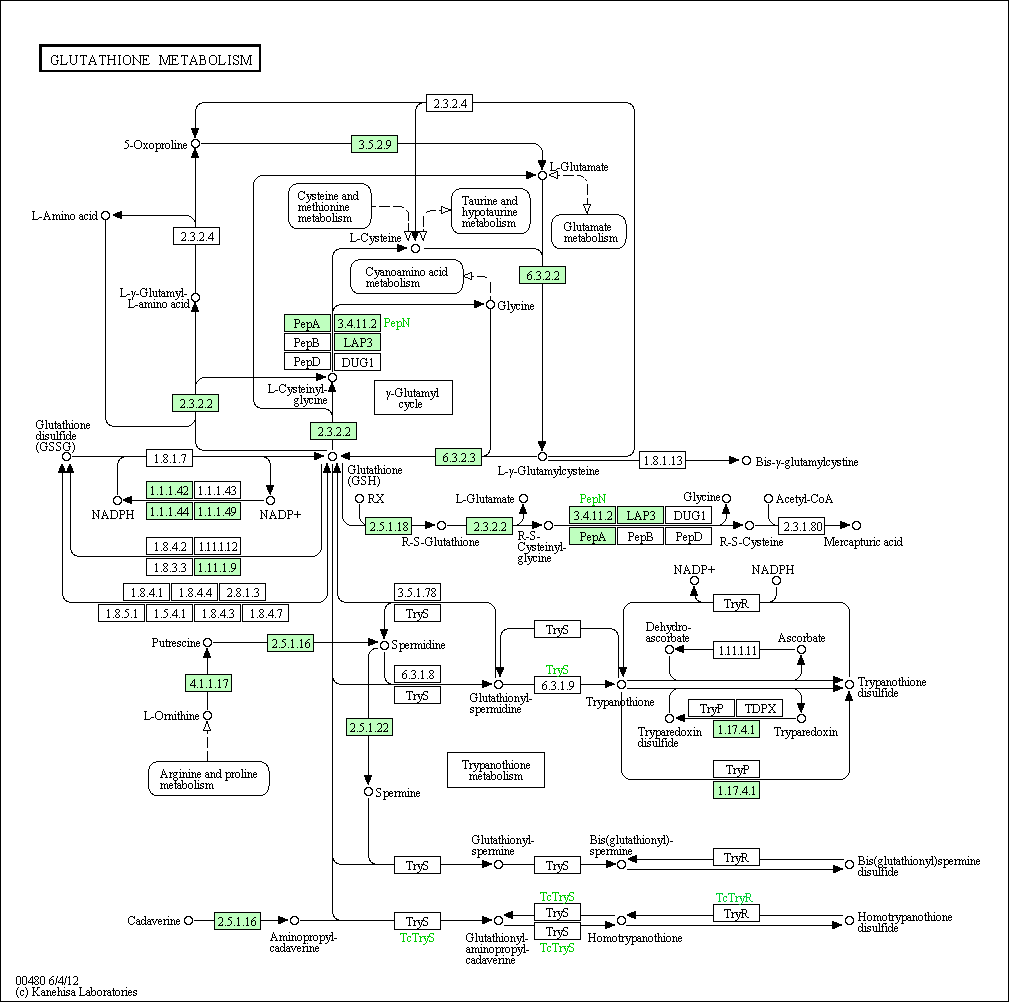

Supplement: Supplementary file 3 — Additional file 3: KEGG classification and functional maps of assembled contigs. Contigs annotated using KEGG Automatic Annotation Server identified sequences in a broad range of functional groups including developmental pathways and cell signaling. (ZIP 11 MB) [file 12864_2013_7026_MOESM3_ESM.zip › KEGG classification/map/map00480.png]

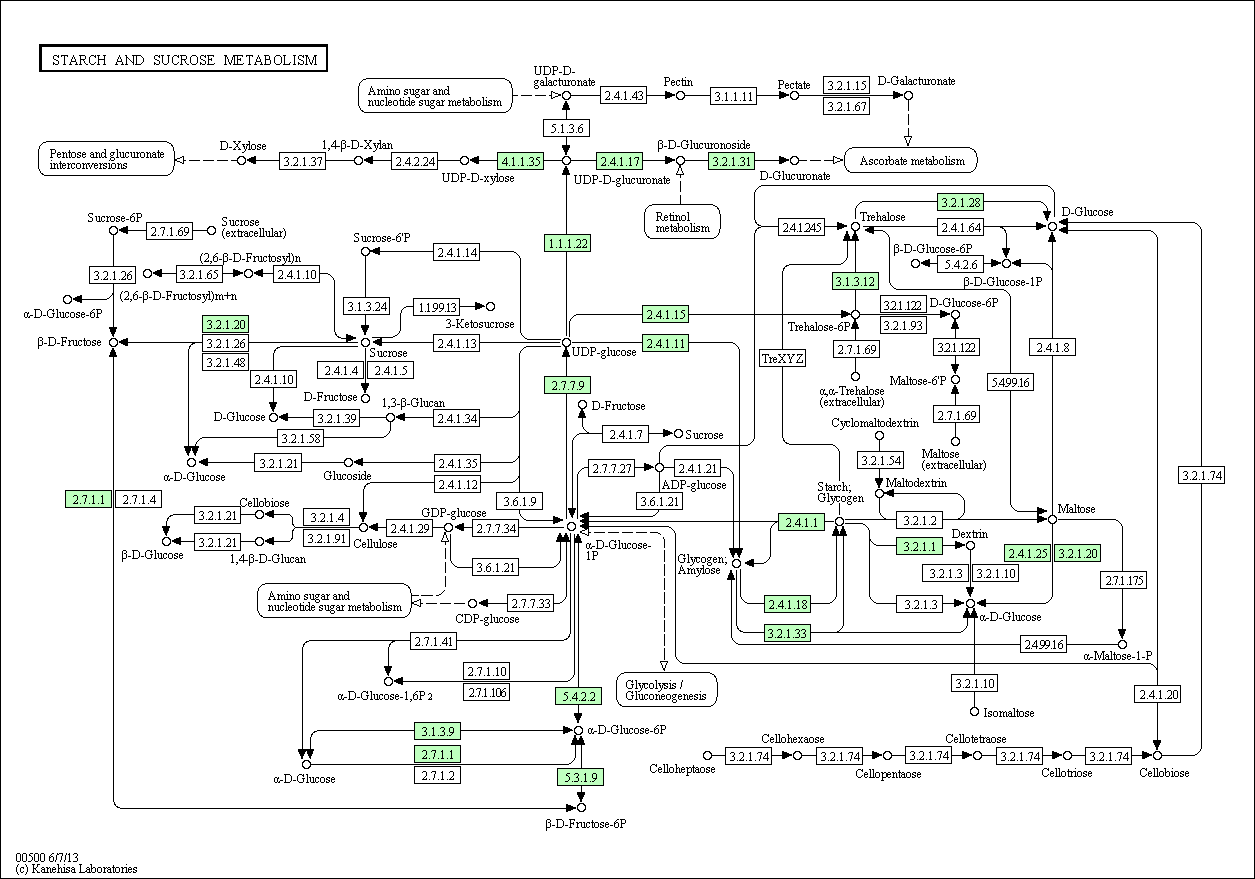

Supplement: Supplementary file 3 — Additional file 3: KEGG classification and functional maps of assembled contigs. Contigs annotated using KEGG Automatic Annotation Server identified sequences in a broad range of functional groups including developmental pathways and cell signaling. (ZIP 11 MB) [file 12864_2013_7026_MOESM3_ESM.zip › KEGG classification/map/map00500.png]

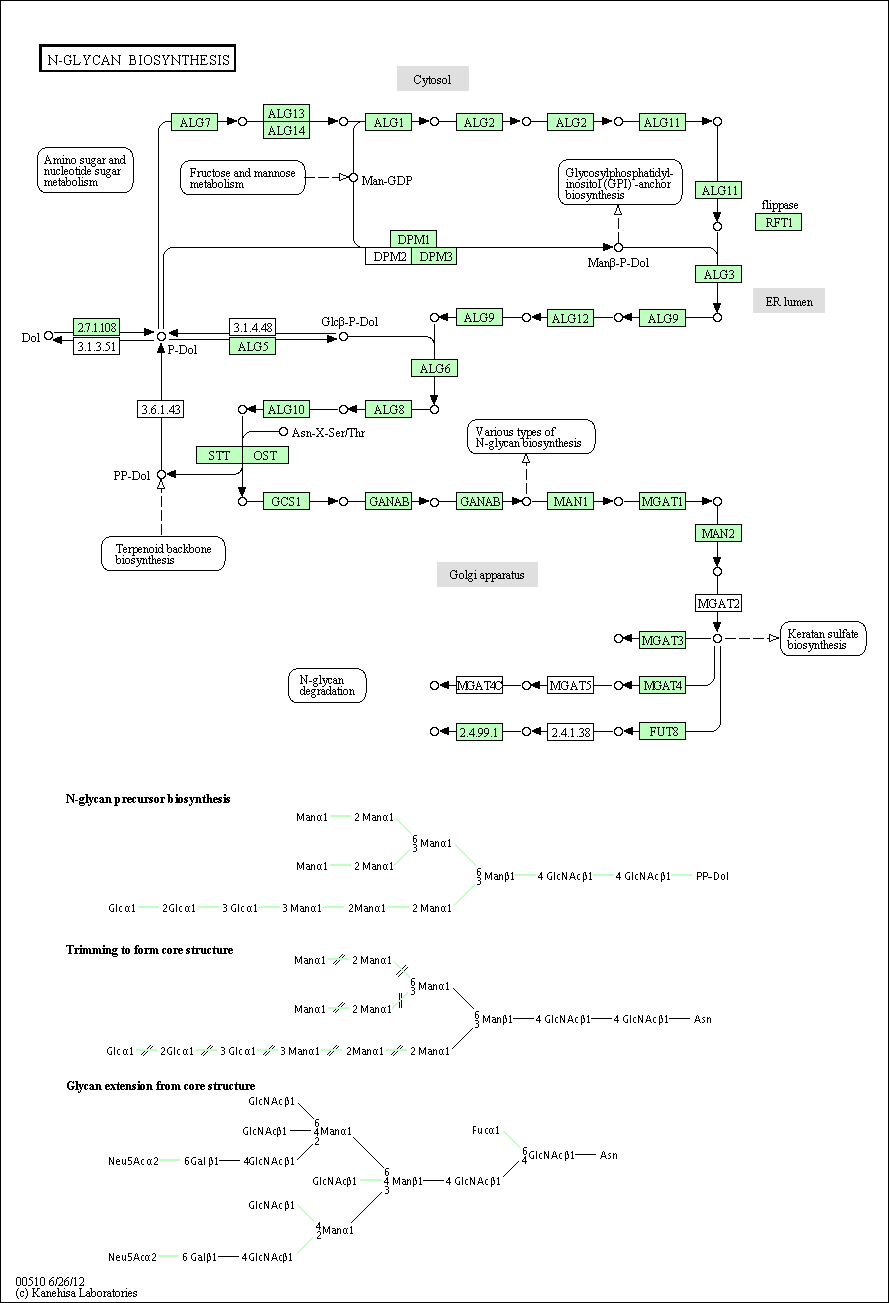

Supplement: Supplementary file 3 — Additional file 3: KEGG classification and functional maps of assembled contigs. Contigs annotated using KEGG Automatic Annotation Server identified sequences in a broad range of functional groups including developmental pathways and cell signaling. (ZIP 11 MB) [file 12864_2013_7026_MOESM3_ESM.zip › KEGG classification/map/map00510.png]

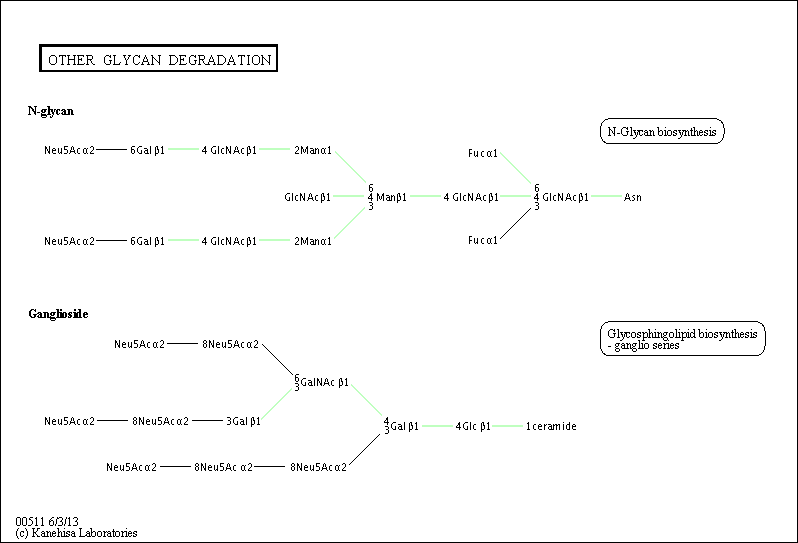

Supplement: Supplementary file 3 — Additional file 3: KEGG classification and functional maps of assembled contigs. Contigs annotated using KEGG Automatic Annotation Server identified sequences in a broad range of functional groups including developmental pathways and cell signaling. (ZIP 11 MB) [file 12864_2013_7026_MOESM3_ESM.zip › KEGG classification/map/map00511.png]

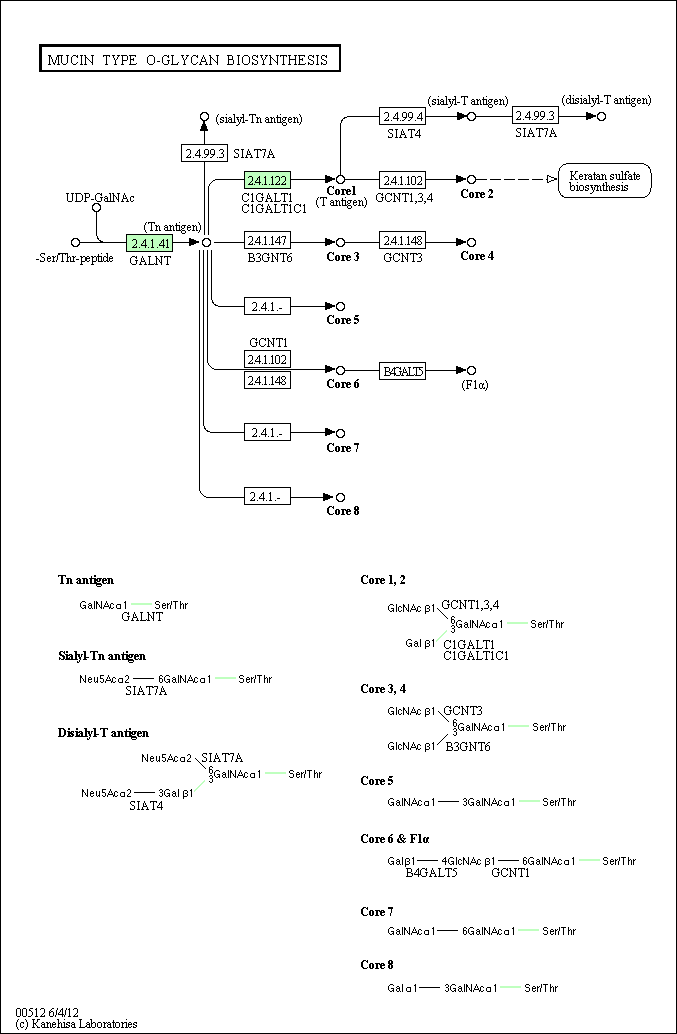

Supplement: Supplementary file 3 — Additional file 3: KEGG classification and functional maps of assembled contigs. Contigs annotated using KEGG Automatic Annotation Server identified sequences in a broad range of functional groups including developmental pathways and cell signaling. (ZIP 11 MB) [file 12864_2013_7026_MOESM3_ESM.zip › KEGG classification/map/map00512.png]

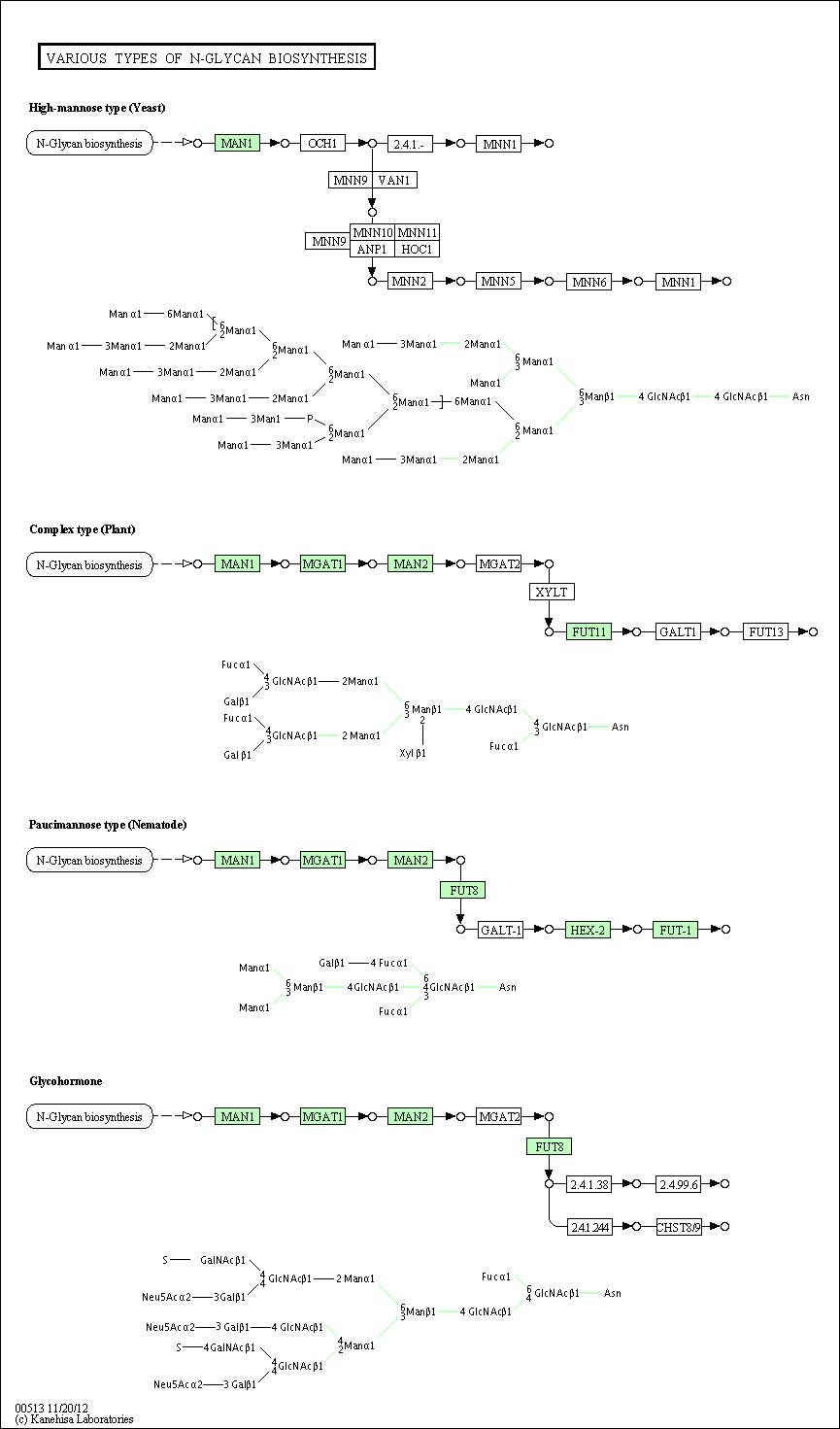

Supplement: Supplementary file 3 — Additional file 3: KEGG classification and functional maps of assembled contigs. Contigs annotated using KEGG Automatic Annotation Server identified sequences in a broad range of functional groups including developmental pathways and cell signaling. (ZIP 11 MB) [file 12864_2013_7026_MOESM3_ESM.zip › KEGG classification/map/map00513.png]

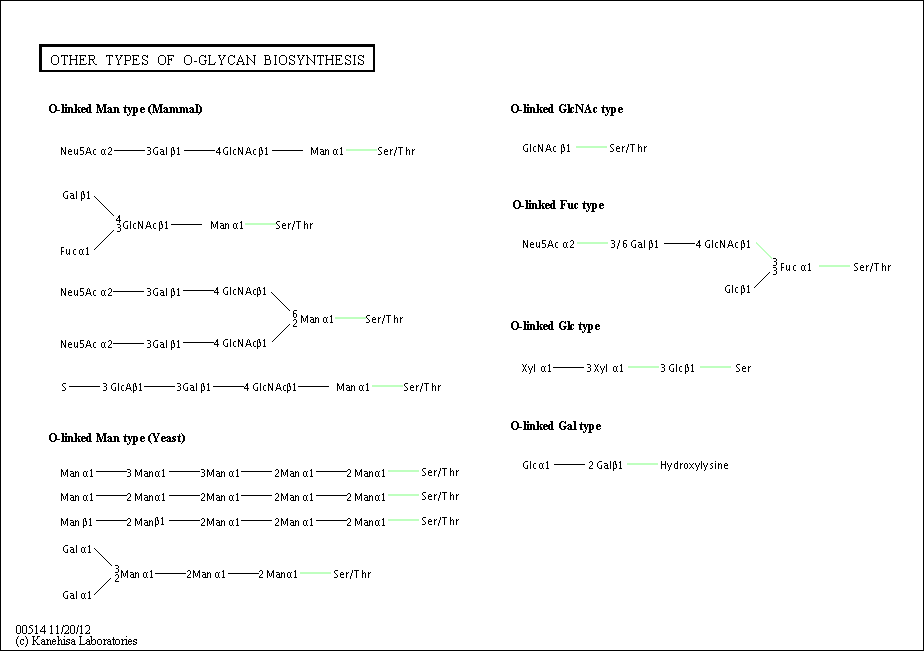

Supplement: Supplementary file 3 — Additional file 3: KEGG classification and functional maps of assembled contigs. Contigs annotated using KEGG Automatic Annotation Server identified sequences in a broad range of functional groups including developmental pathways and cell signaling. (ZIP 11 MB) [file 12864_2013_7026_MOESM3_ESM.zip › KEGG classification/map/map00514.png]

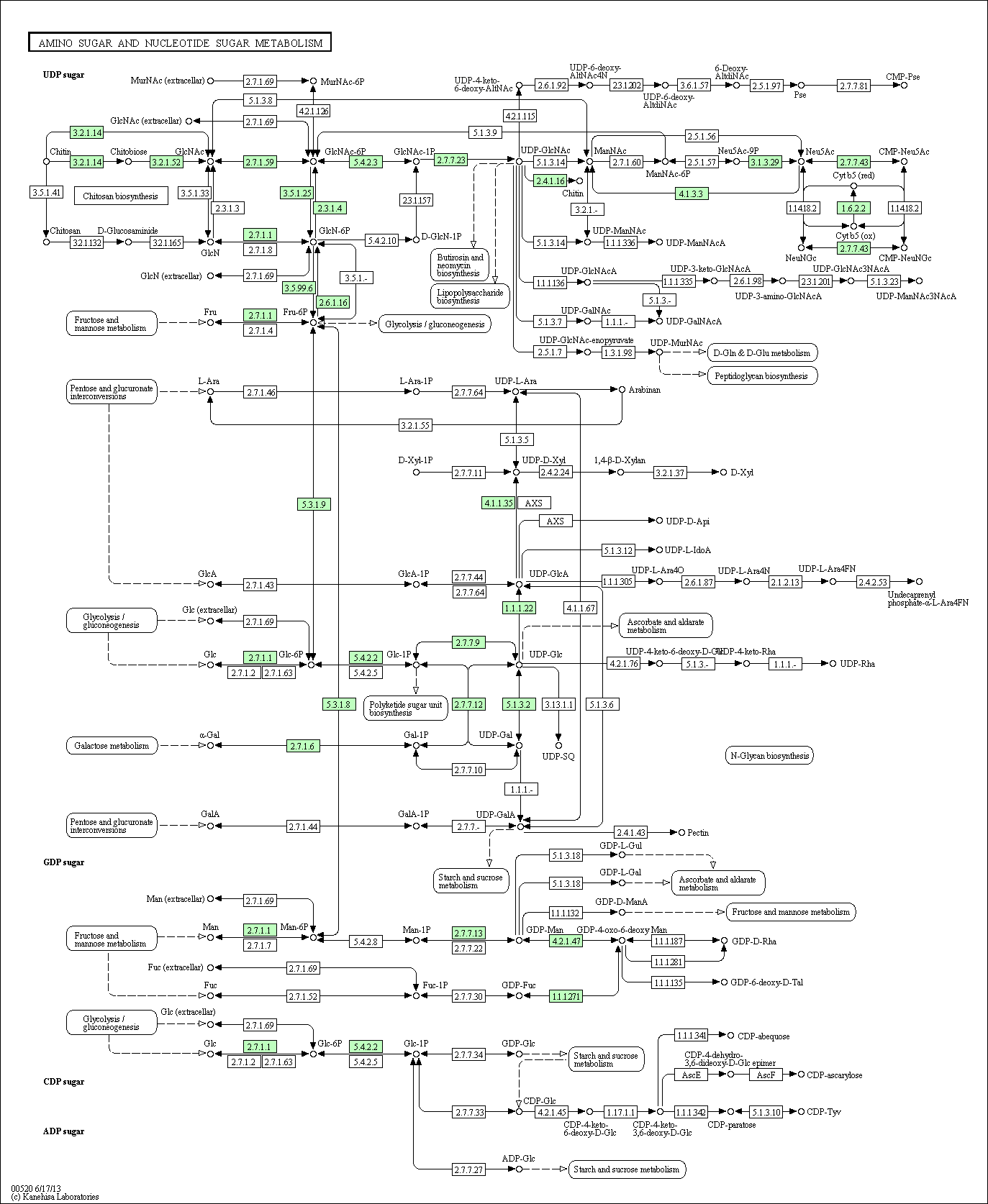

Supplement: Supplementary file 3 — Additional file 3: KEGG classification and functional maps of assembled contigs. Contigs annotated using KEGG Automatic Annotation Server identified sequences in a broad range of functional groups including developmental pathways and cell signaling. (ZIP 11 MB) [file 12864_2013_7026_MOESM3_ESM.zip › KEGG classification/map/map00520.png]

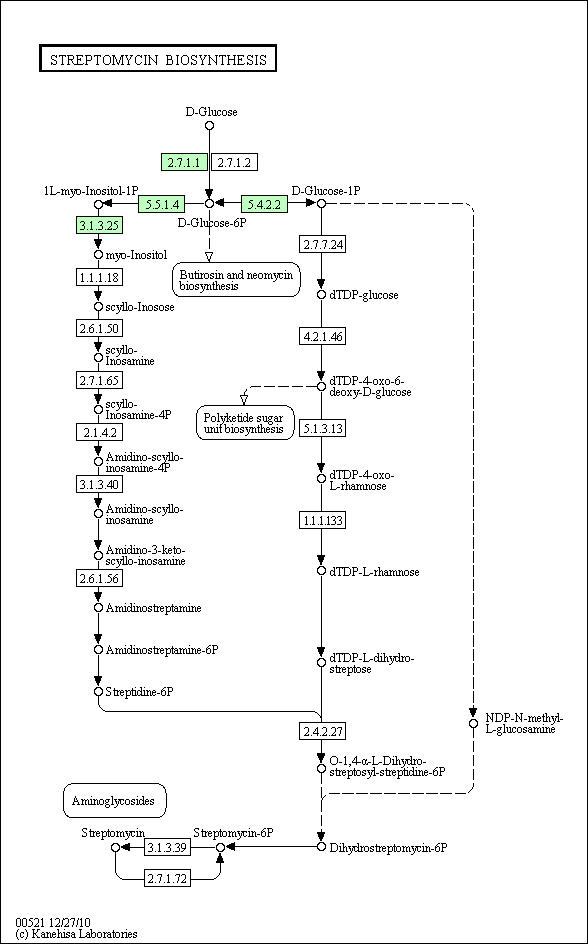

Supplement: Supplementary file 3 — Additional file 3: KEGG classification and functional maps of assembled contigs. Contigs annotated using KEGG Automatic Annotation Server identified sequences in a broad range of functional groups including developmental pathways and cell signaling. (ZIP 11 MB) [file 12864_2013_7026_MOESM3_ESM.zip › KEGG classification/map/map00521.png]

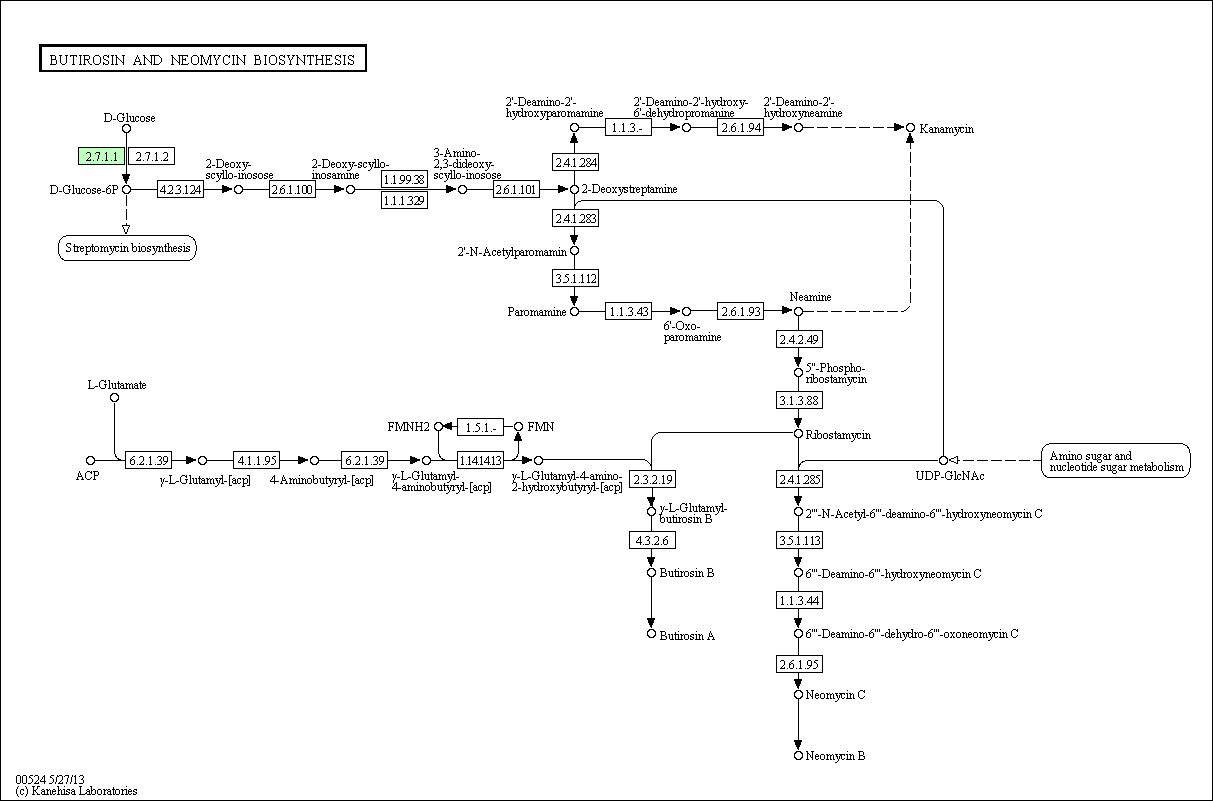

Supplement: Supplementary file 3 — Additional file 3: KEGG classification and functional maps of assembled contigs. Contigs annotated using KEGG Automatic Annotation Server identified sequences in a broad range of functional groups including developmental pathways and cell signaling. (ZIP 11 MB) [file 12864_2013_7026_MOESM3_ESM.zip › KEGG classification/map/map00524.png]

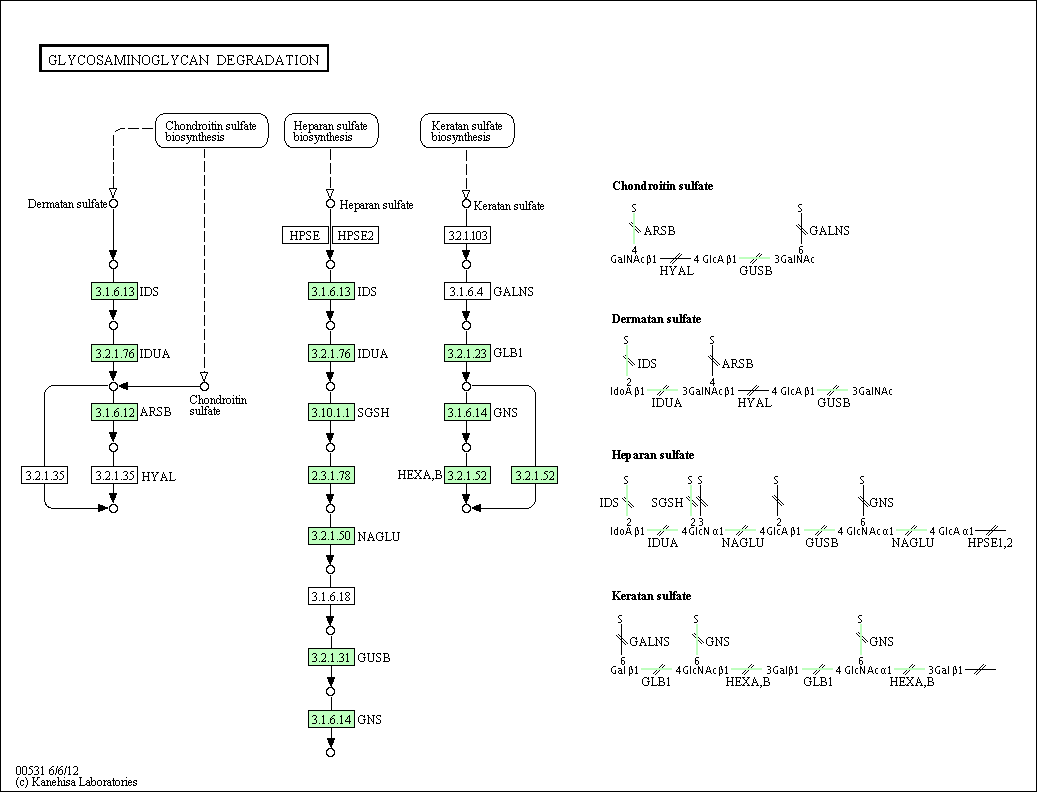

Supplement: Supplementary file 3 — Additional file 3: KEGG classification and functional maps of assembled contigs. Contigs annotated using KEGG Automatic Annotation Server identified sequences in a broad range of functional groups including developmental pathways and cell signaling. (ZIP 11 MB) [file 12864_2013_7026_MOESM3_ESM.zip › KEGG classification/map/map00531.png]

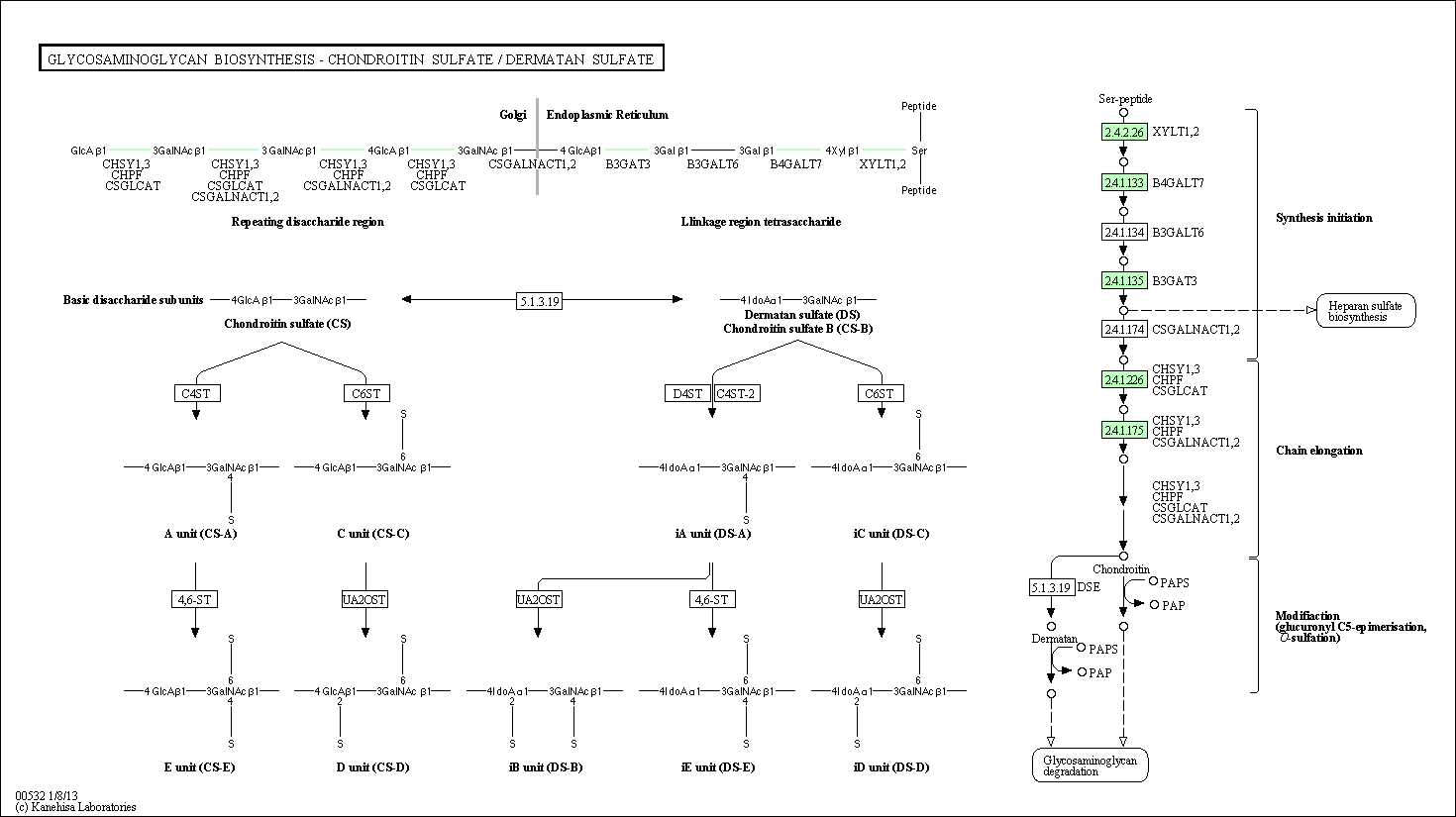

Supplement: Supplementary file 3 — Additional file 3: KEGG classification and functional maps of assembled contigs. Contigs annotated using KEGG Automatic Annotation Server identified sequences in a broad range of functional groups including developmental pathways and cell signaling. (ZIP 11 MB) [file 12864_2013_7026_MOESM3_ESM.zip › KEGG classification/map/map00532.png]

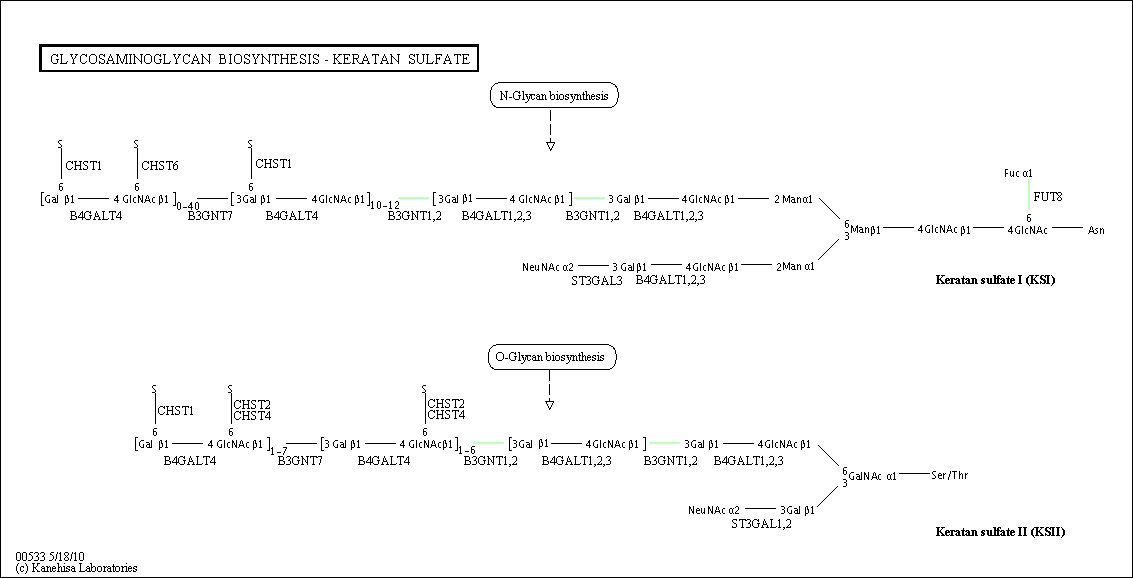

Supplement: Supplementary file 3 — Additional file 3: KEGG classification and functional maps of assembled contigs. Contigs annotated using KEGG Automatic Annotation Server identified sequences in a broad range of functional groups including developmental pathways and cell signaling. (ZIP 11 MB) [file 12864_2013_7026_MOESM3_ESM.zip › KEGG classification/map/map00533.png]
