# Supplementary figures and images for: A pipeline for the de novo assembly of the Themira biloba (Sepsidae: Diptera) transcriptome using a multiple k-mer length approach (part 2 of 2)
Source: BMC Genomics. 2014 Mar 12;15(1):188. doi: 10.1186/1471-2164-15-188 (PMC4008362; doi:10.1186/1471-2164-15-188)

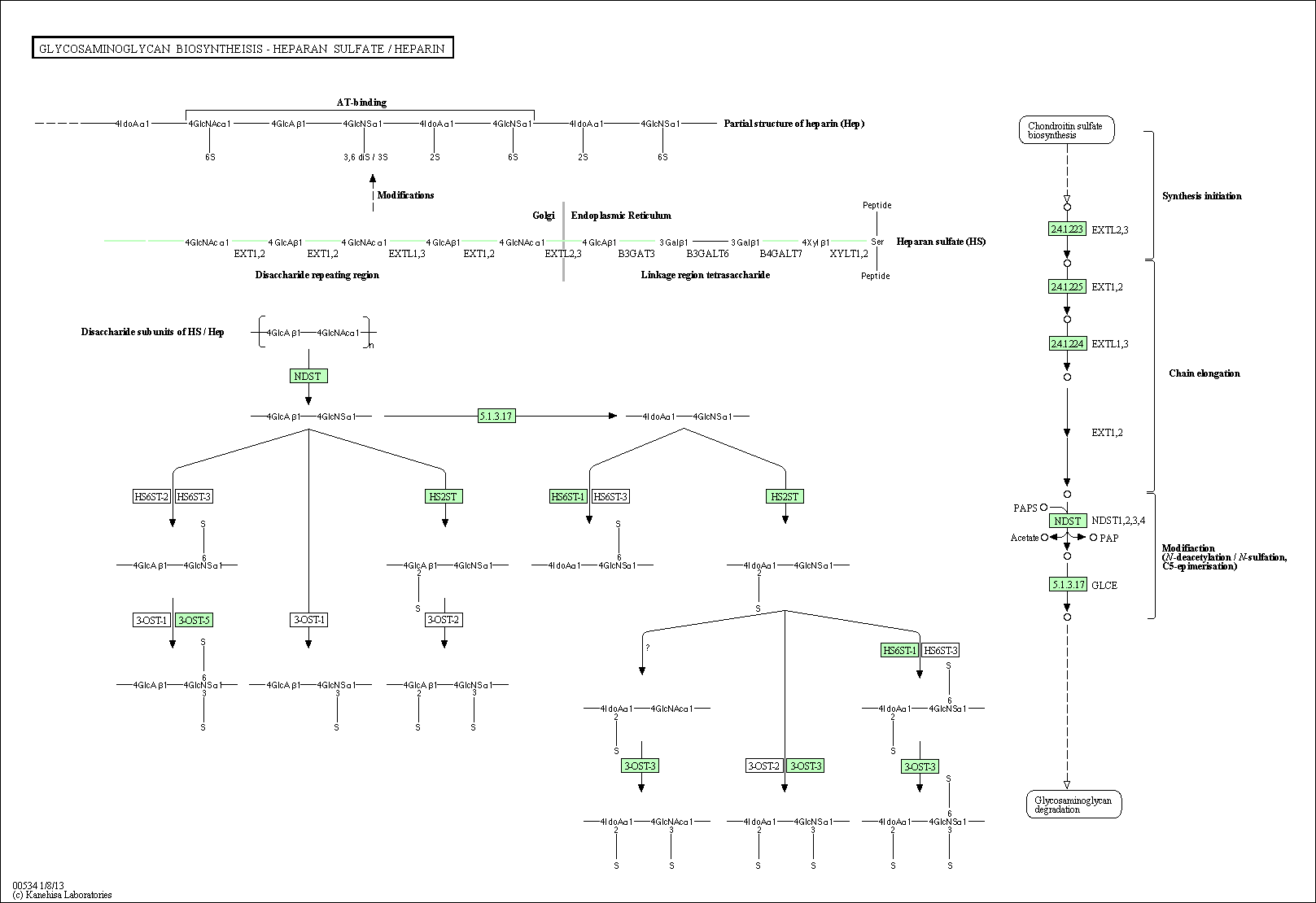

Supplement: Supplementary file 3 — Additional file 3: KEGG classification and functional maps of assembled contigs. Contigs annotated using KEGG Automatic Annotation Server identified sequences in a broad range of functional groups including developmental pathways and cell signaling. (ZIP 11 MB) [file 12864_2013_7026_MOESM3_ESM.zip › KEGG classification/map/map00534.png]

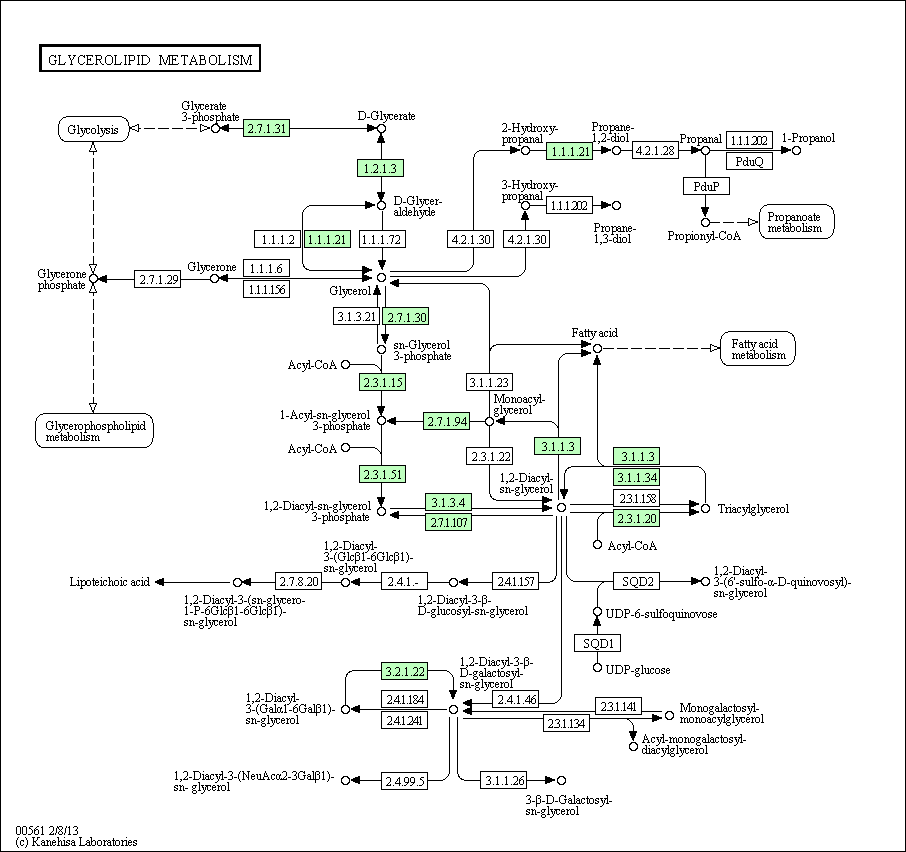

Supplement: Supplementary file 3 — Additional file 3: KEGG classification and functional maps of assembled contigs. Contigs annotated using KEGG Automatic Annotation Server identified sequences in a broad range of functional groups including developmental pathways and cell signaling. (ZIP 11 MB) [file 12864_2013_7026_MOESM3_ESM.zip › KEGG classification/map/map00561.png]

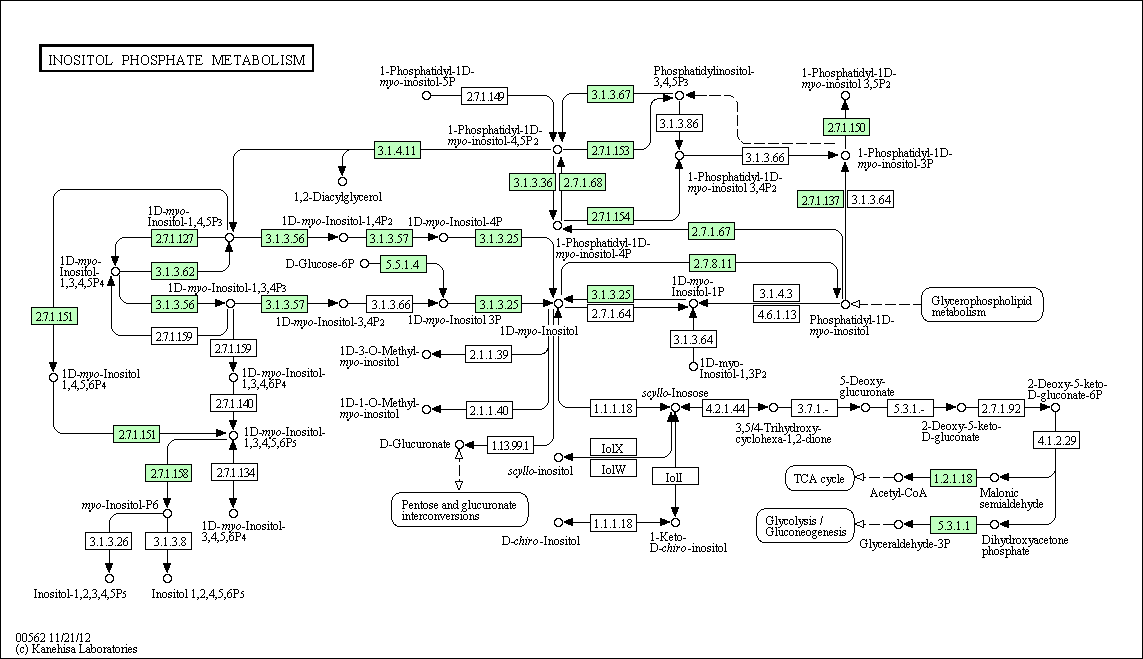

Supplement: Supplementary file 3 — Additional file 3: KEGG classification and functional maps of assembled contigs. Contigs annotated using KEGG Automatic Annotation Server identified sequences in a broad range of functional groups including developmental pathways and cell signaling. (ZIP 11 MB) [file 12864_2013_7026_MOESM3_ESM.zip › KEGG classification/map/map00562.png]

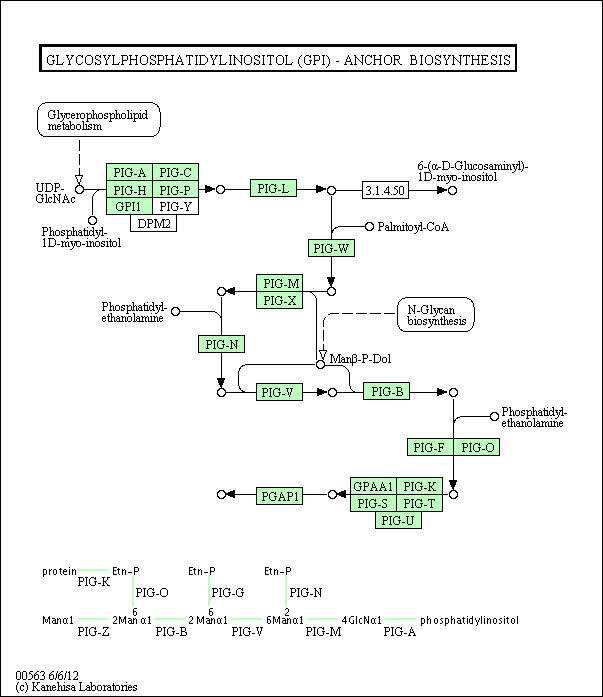

Supplement: Supplementary file 3 — Additional file 3: KEGG classification and functional maps of assembled contigs. Contigs annotated using KEGG Automatic Annotation Server identified sequences in a broad range of functional groups including developmental pathways and cell signaling. (ZIP 11 MB) [file 12864_2013_7026_MOESM3_ESM.zip › KEGG classification/map/map00563.png]

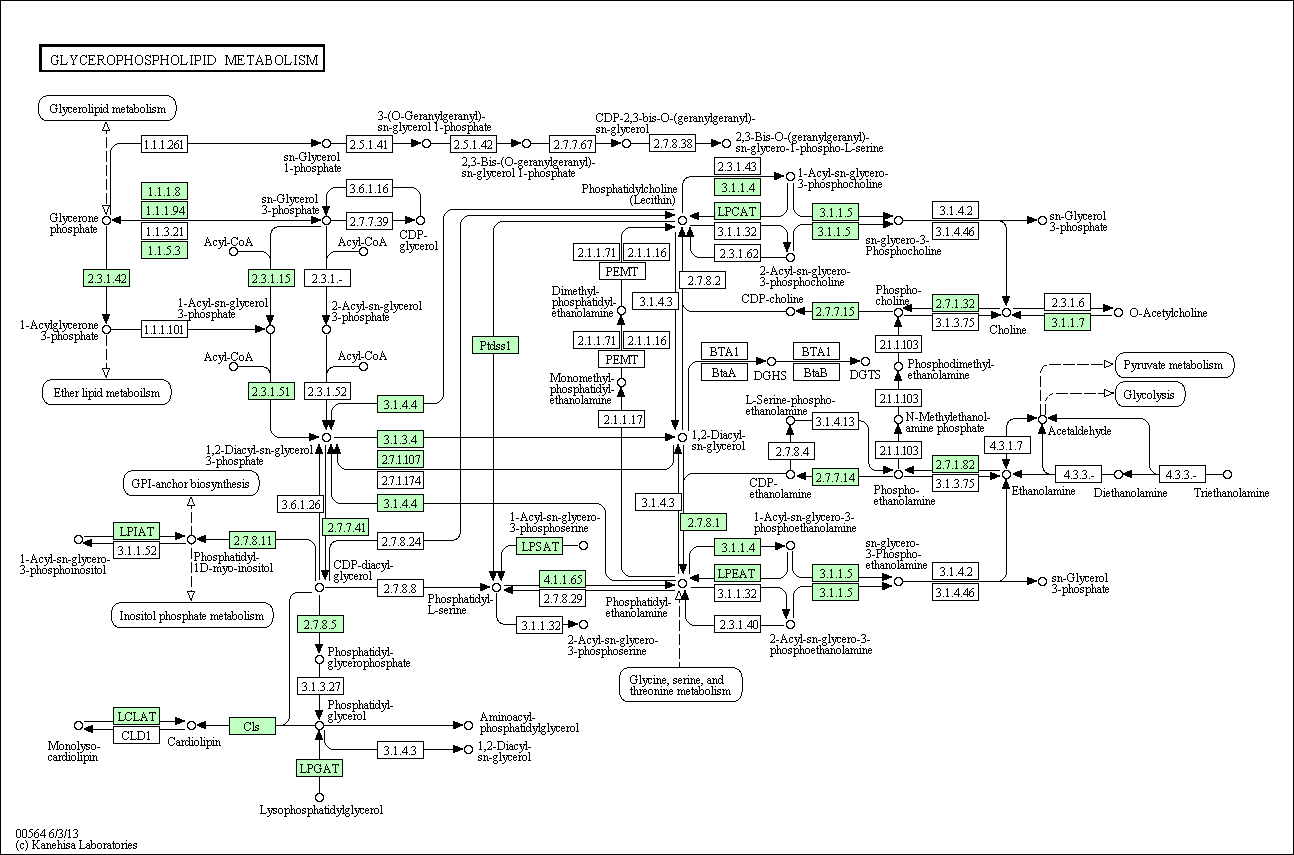

Supplement: Supplementary file 3 — Additional file 3: KEGG classification and functional maps of assembled contigs. Contigs annotated using KEGG Automatic Annotation Server identified sequences in a broad range of functional groups including developmental pathways and cell signaling. (ZIP 11 MB) [file 12864_2013_7026_MOESM3_ESM.zip › KEGG classification/map/map00564.png]

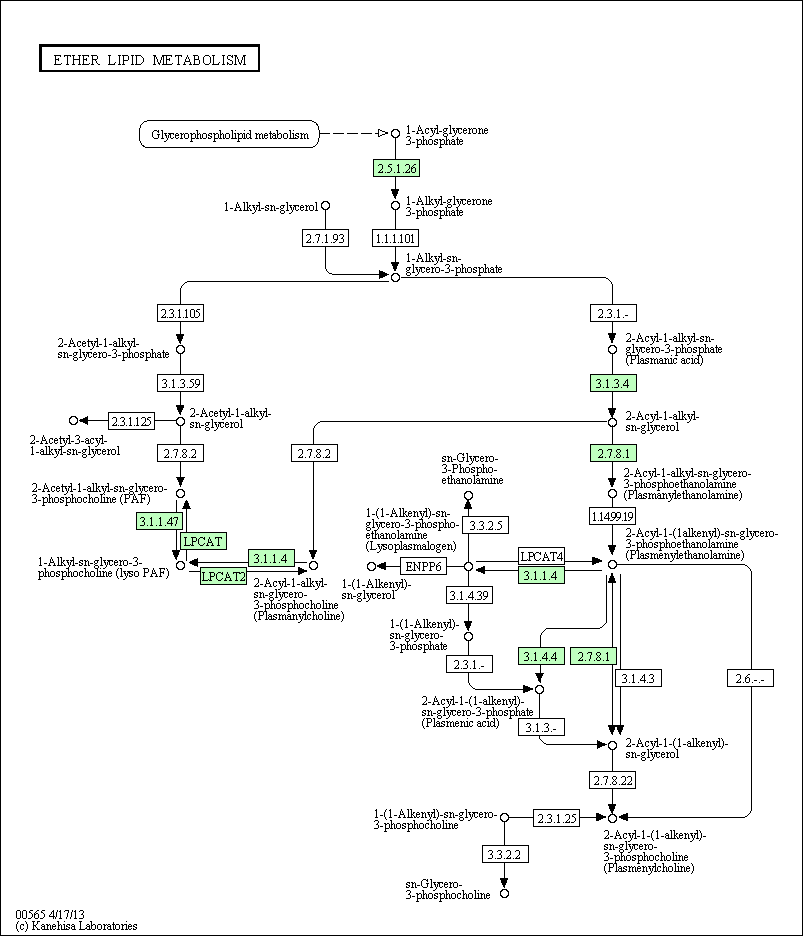

Supplement: Supplementary file 3 — Additional file 3: KEGG classification and functional maps of assembled contigs. Contigs annotated using KEGG Automatic Annotation Server identified sequences in a broad range of functional groups including developmental pathways and cell signaling. (ZIP 11 MB) [file 12864_2013_7026_MOESM3_ESM.zip › KEGG classification/map/map00565.png]

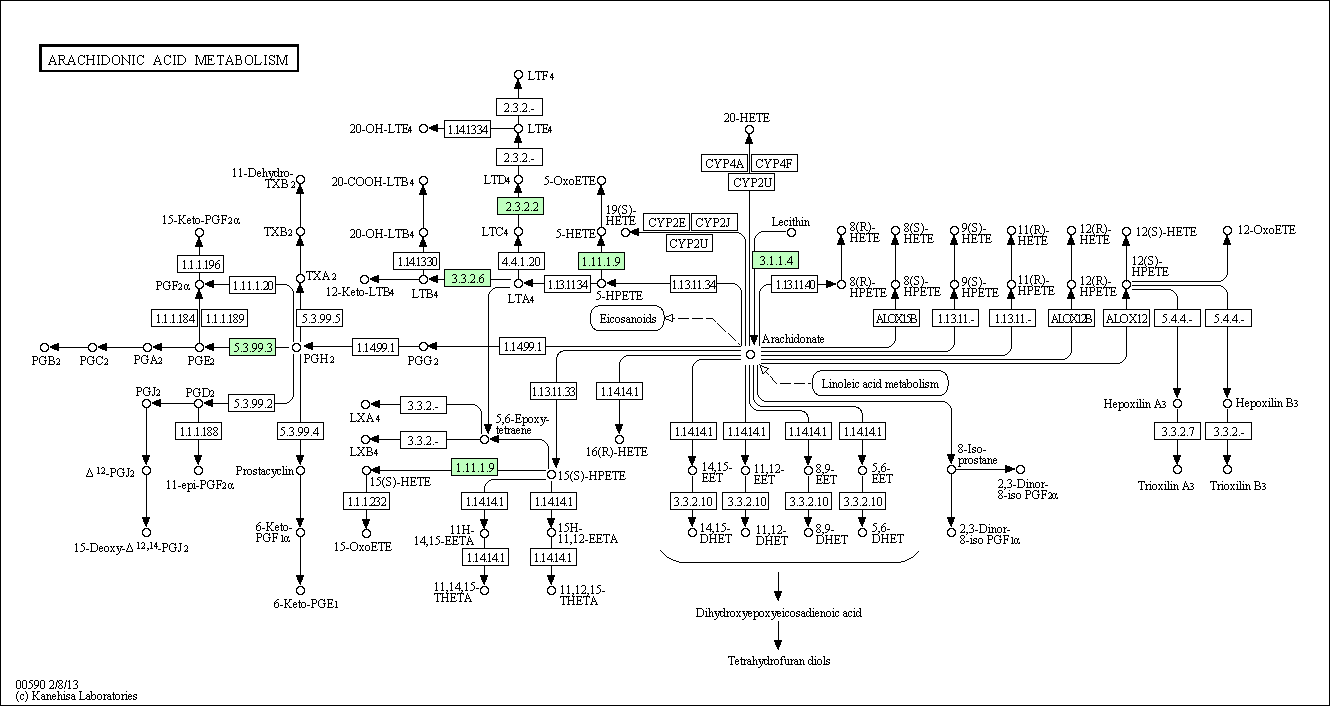

Supplement: Supplementary file 3 — Additional file 3: KEGG classification and functional maps of assembled contigs. Contigs annotated using KEGG Automatic Annotation Server identified sequences in a broad range of functional groups including developmental pathways and cell signaling. (ZIP 11 MB) [file 12864_2013_7026_MOESM3_ESM.zip › KEGG classification/map/map00590.png]

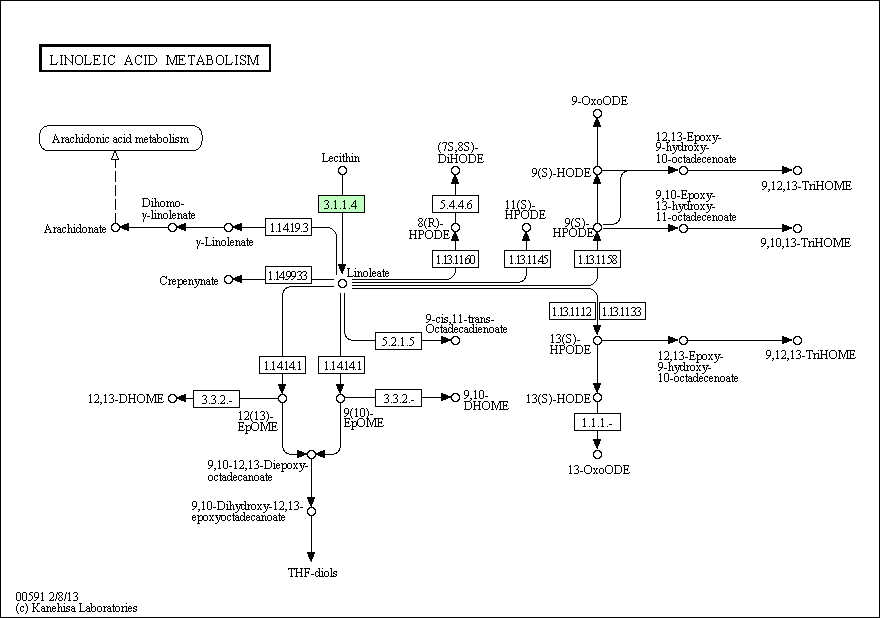

Supplement: Supplementary file 3 — Additional file 3: KEGG classification and functional maps of assembled contigs. Contigs annotated using KEGG Automatic Annotation Server identified sequences in a broad range of functional groups including developmental pathways and cell signaling. (ZIP 11 MB) [file 12864_2013_7026_MOESM3_ESM.zip › KEGG classification/map/map00591.png]

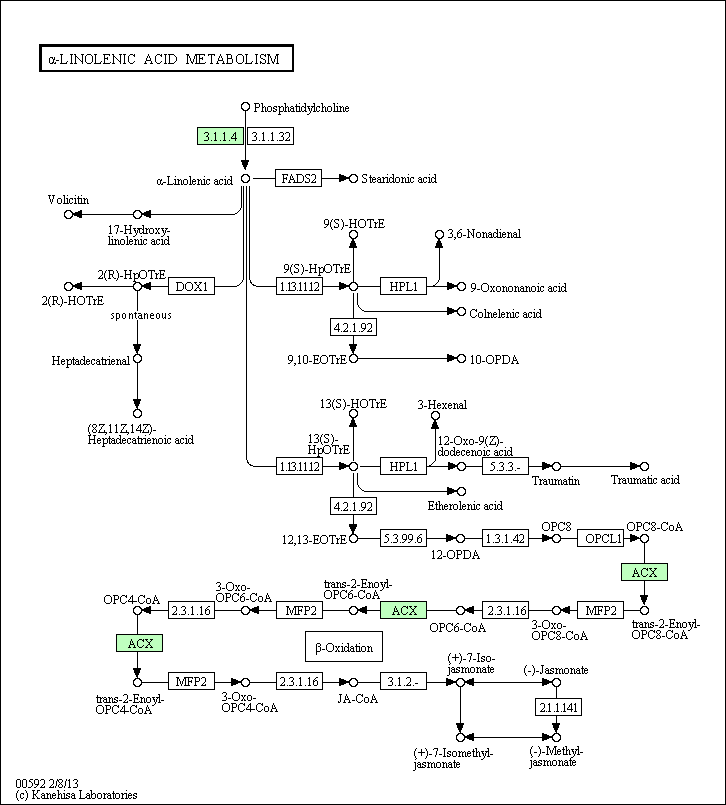

Supplement: Supplementary file 3 — Additional file 3: KEGG classification and functional maps of assembled contigs. Contigs annotated using KEGG Automatic Annotation Server identified sequences in a broad range of functional groups including developmental pathways and cell signaling. (ZIP 11 MB) [file 12864_2013_7026_MOESM3_ESM.zip › KEGG classification/map/map00592.png]

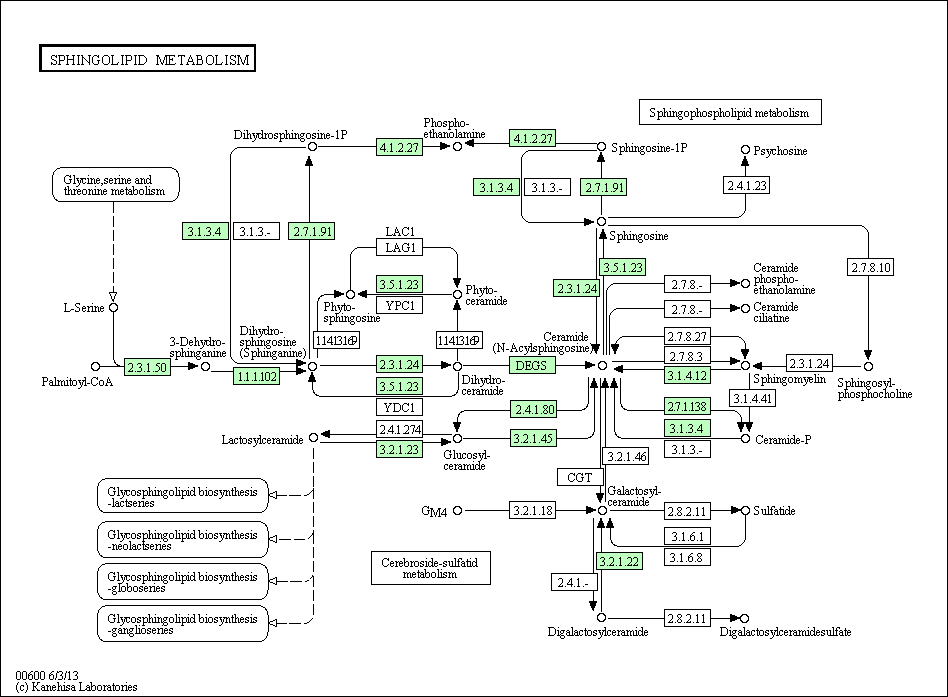

Supplement: Supplementary file 3 — Additional file 3: KEGG classification and functional maps of assembled contigs. Contigs annotated using KEGG Automatic Annotation Server identified sequences in a broad range of functional groups including developmental pathways and cell signaling. (ZIP 11 MB) [file 12864_2013_7026_MOESM3_ESM.zip › KEGG classification/map/map00600.png]

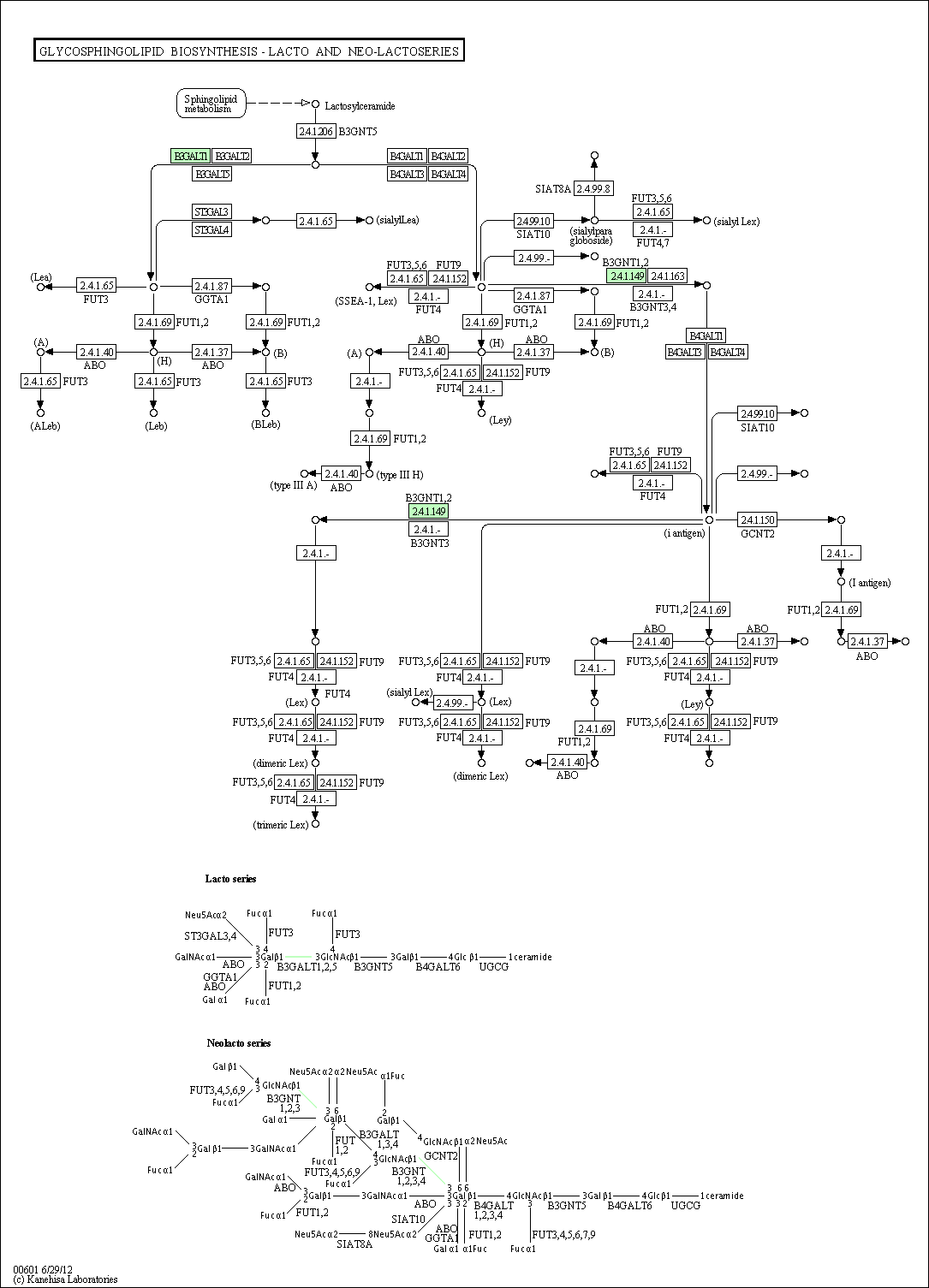

Supplement: Supplementary file 3 — Additional file 3: KEGG classification and functional maps of assembled contigs. Contigs annotated using KEGG Automatic Annotation Server identified sequences in a broad range of functional groups including developmental pathways and cell signaling. (ZIP 11 MB) [file 12864_2013_7026_MOESM3_ESM.zip › KEGG classification/map/map00601.png]

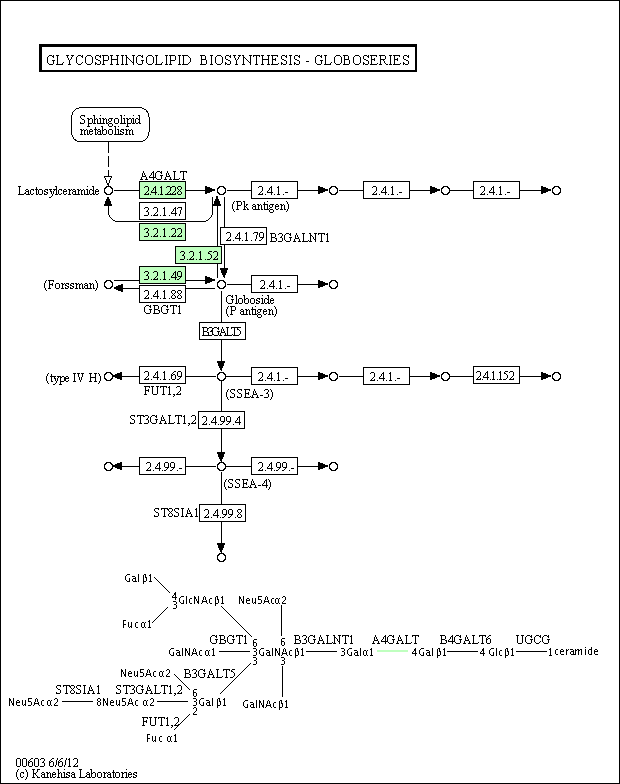

Supplement: Supplementary file 3 — Additional file 3: KEGG classification and functional maps of assembled contigs. Contigs annotated using KEGG Automatic Annotation Server identified sequences in a broad range of functional groups including developmental pathways and cell signaling. (ZIP 11 MB) [file 12864_2013_7026_MOESM3_ESM.zip › KEGG classification/map/map00603.png]

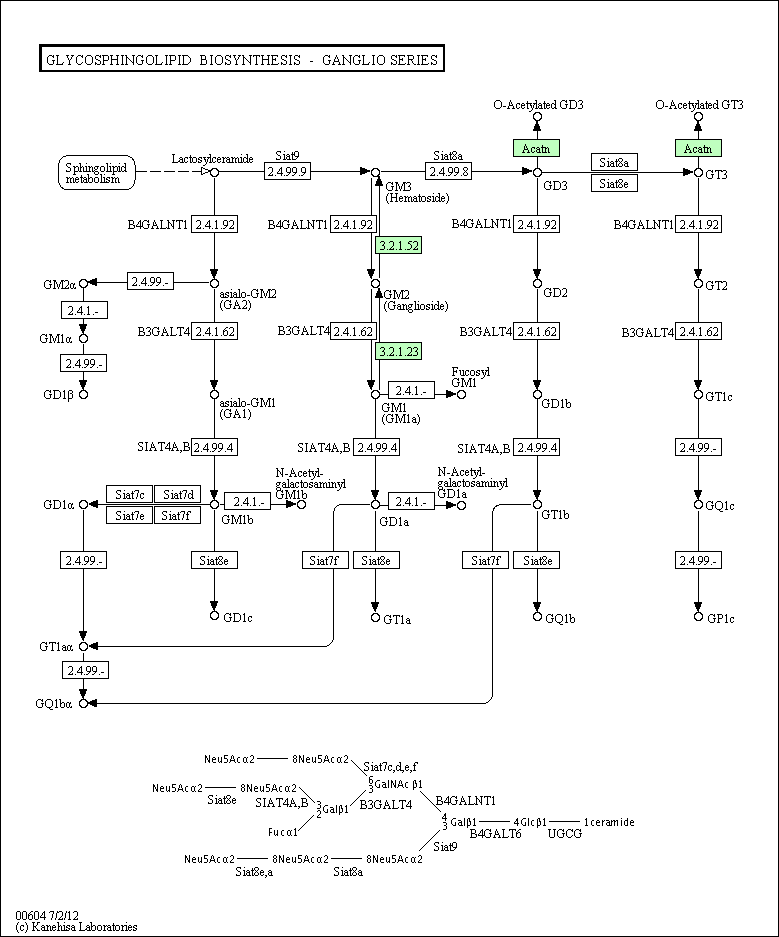

Supplement: Supplementary file 3 — Additional file 3: KEGG classification and functional maps of assembled contigs. Contigs annotated using KEGG Automatic Annotation Server identified sequences in a broad range of functional groups including developmental pathways and cell signaling. (ZIP 11 MB) [file 12864_2013_7026_MOESM3_ESM.zip › KEGG classification/map/map00604.png]

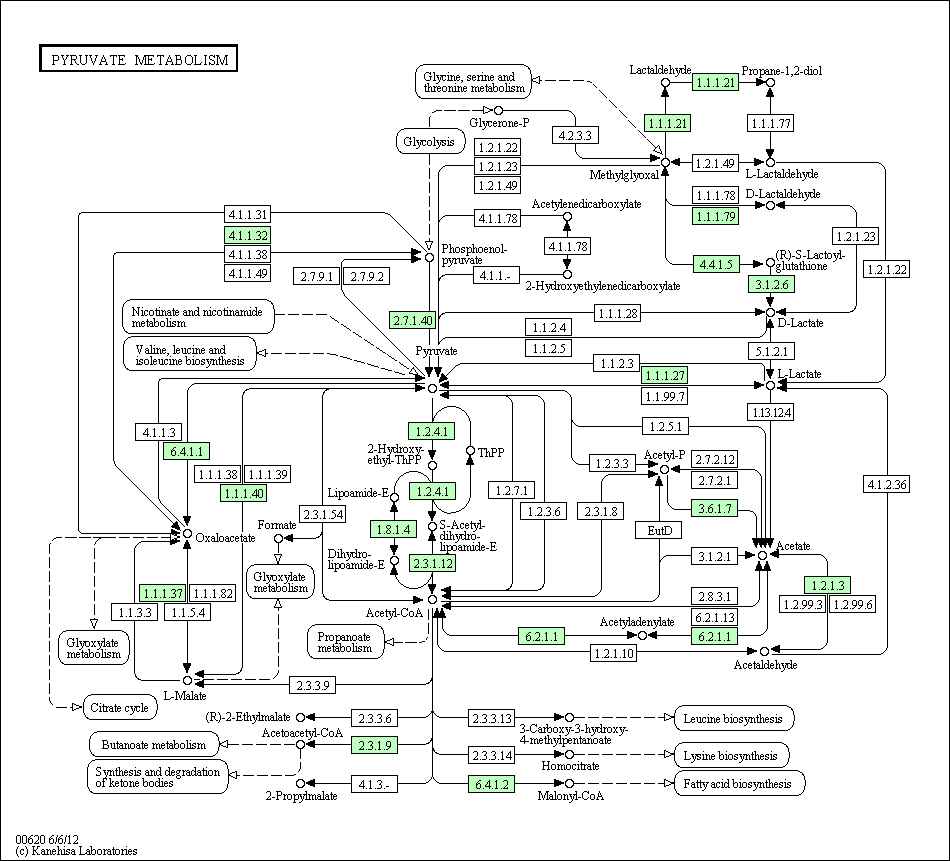

Supplement: Supplementary file 3 — Additional file 3: KEGG classification and functional maps of assembled contigs. Contigs annotated using KEGG Automatic Annotation Server identified sequences in a broad range of functional groups including developmental pathways and cell signaling. (ZIP 11 MB) [file 12864_2013_7026_MOESM3_ESM.zip › KEGG classification/map/map00620.png]

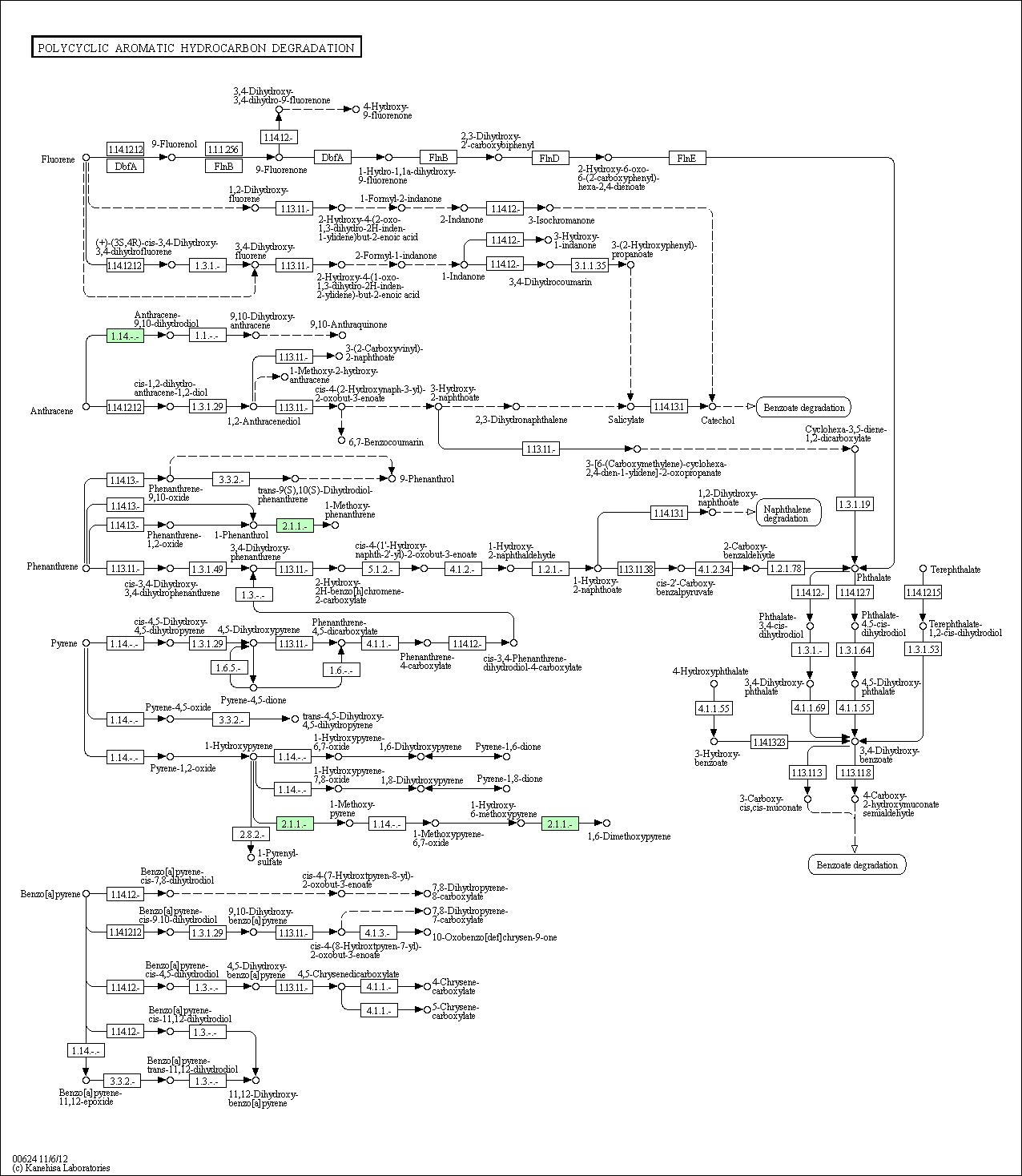

Supplement: Supplementary file 3 — Additional file 3: KEGG classification and functional maps of assembled contigs. Contigs annotated using KEGG Automatic Annotation Server identified sequences in a broad range of functional groups including developmental pathways and cell signaling. (ZIP 11 MB) [file 12864_2013_7026_MOESM3_ESM.zip › KEGG classification/map/map00624.png]

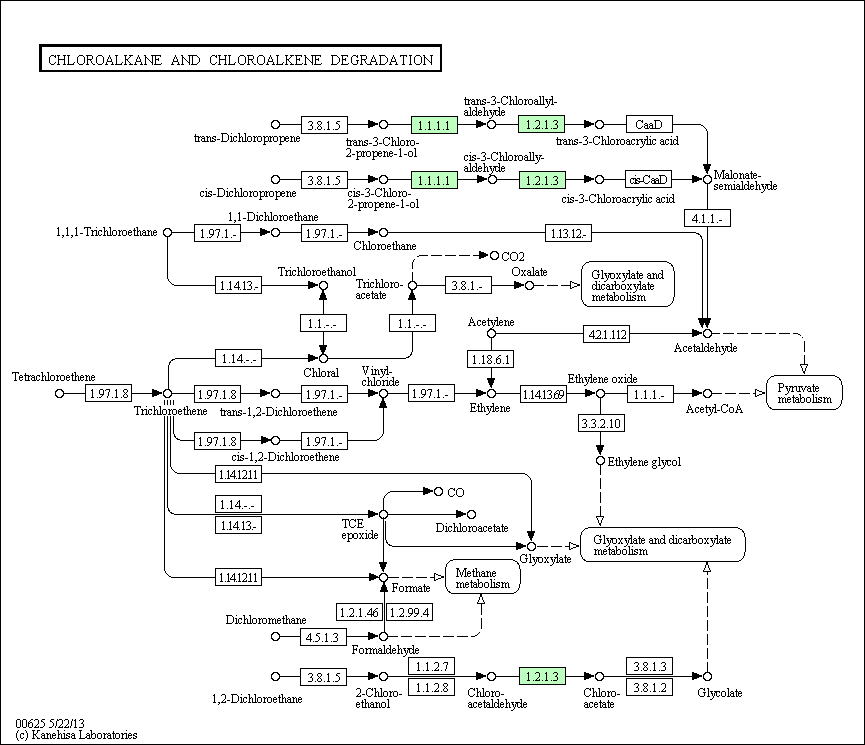

Supplement: Supplementary file 3 — Additional file 3: KEGG classification and functional maps of assembled contigs. Contigs annotated using KEGG Automatic Annotation Server identified sequences in a broad range of functional groups including developmental pathways and cell signaling. (ZIP 11 MB) [file 12864_2013_7026_MOESM3_ESM.zip › KEGG classification/map/map00625.png]

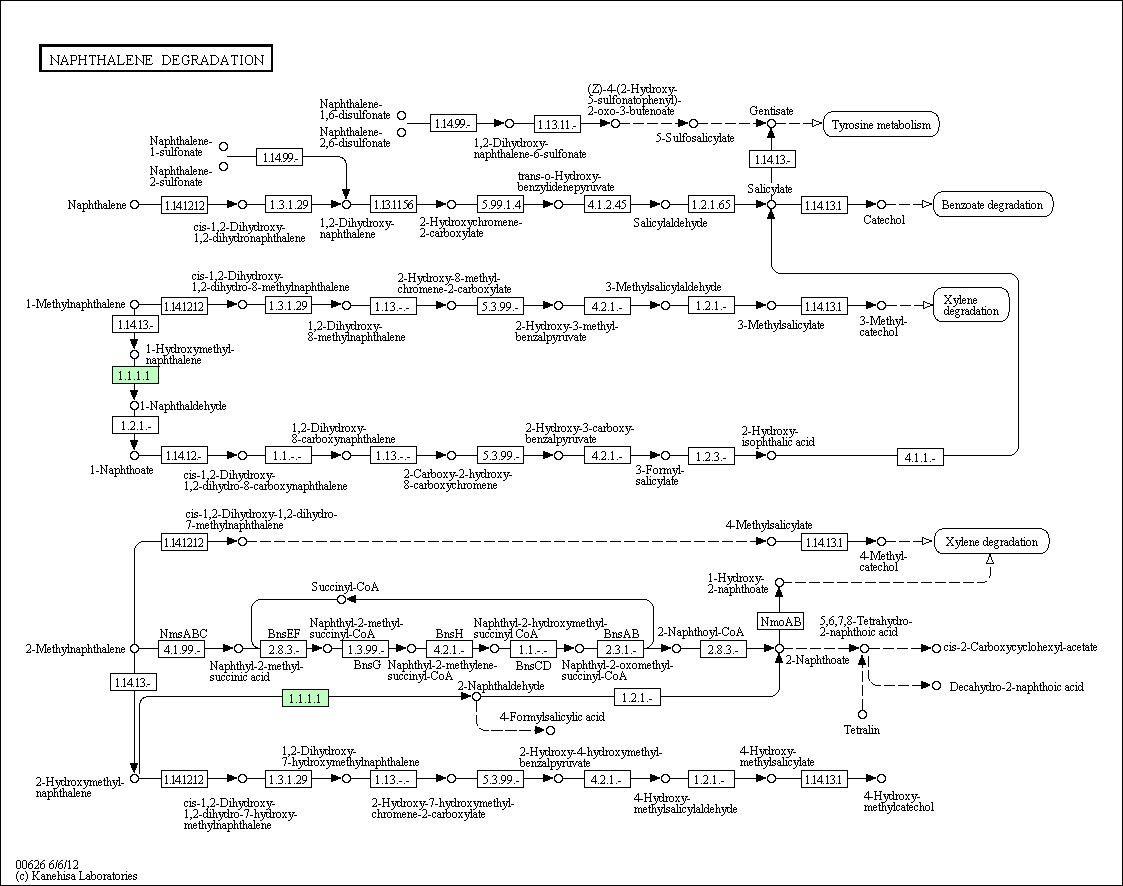

Supplement: Supplementary file 3 — Additional file 3: KEGG classification and functional maps of assembled contigs. Contigs annotated using KEGG Automatic Annotation Server identified sequences in a broad range of functional groups including developmental pathways and cell signaling. (ZIP 11 MB) [file 12864_2013_7026_MOESM3_ESM.zip › KEGG classification/map/map00626.png]

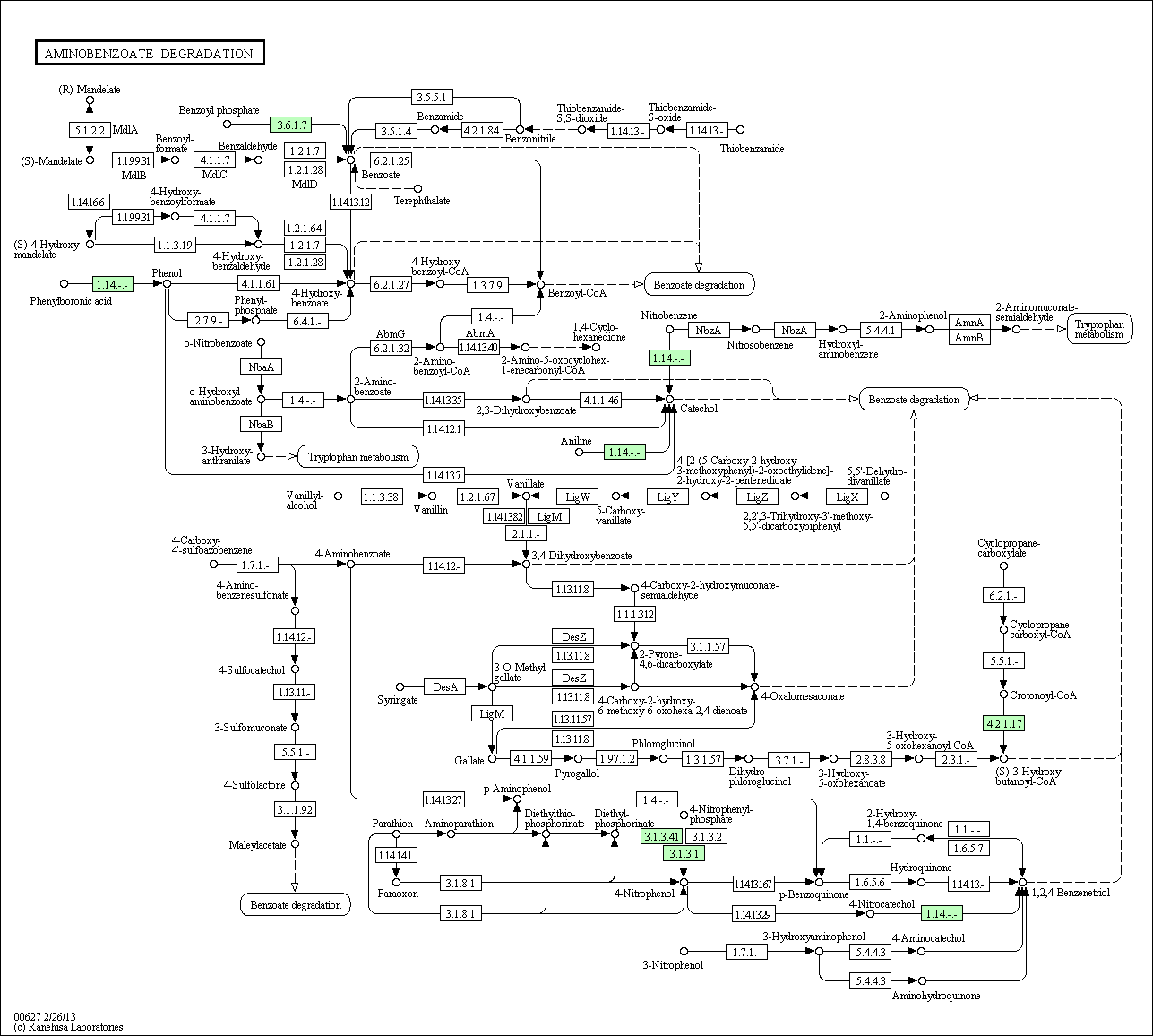

Supplement: Supplementary file 3 — Additional file 3: KEGG classification and functional maps of assembled contigs. Contigs annotated using KEGG Automatic Annotation Server identified sequences in a broad range of functional groups including developmental pathways and cell signaling. (ZIP 11 MB) [file 12864_2013_7026_MOESM3_ESM.zip › KEGG classification/map/map00627.png]

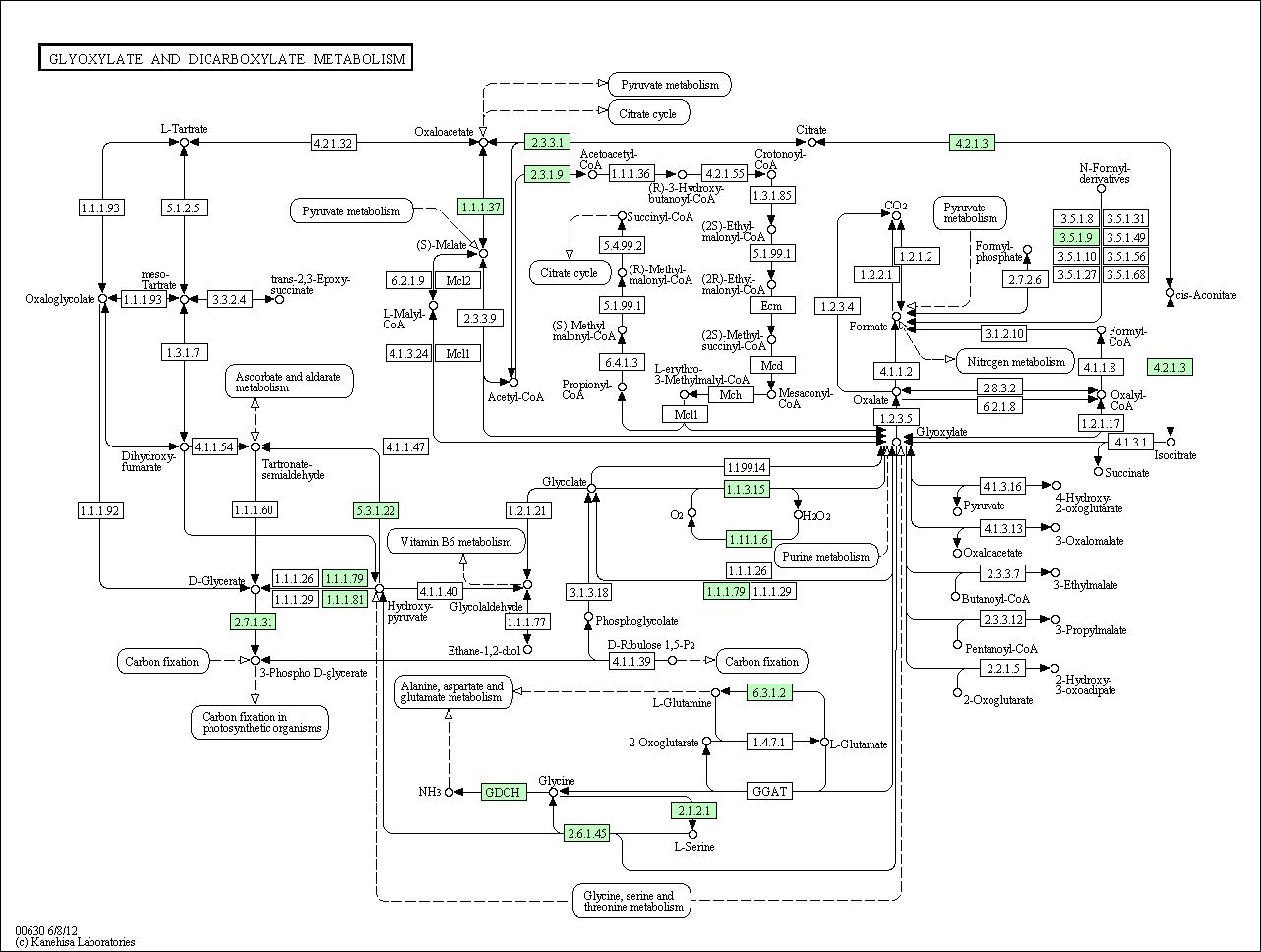

Supplement: Supplementary file 3 — Additional file 3: KEGG classification and functional maps of assembled contigs. Contigs annotated using KEGG Automatic Annotation Server identified sequences in a broad range of functional groups including developmental pathways and cell signaling. (ZIP 11 MB) [file 12864_2013_7026_MOESM3_ESM.zip › KEGG classification/map/map00630.png]

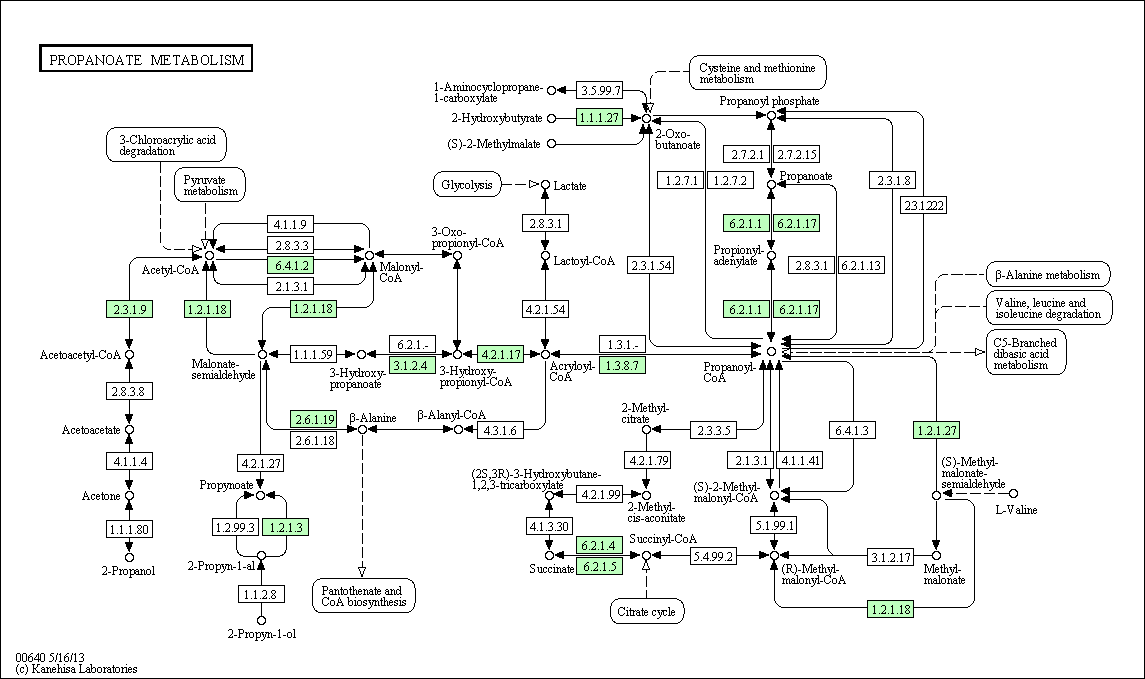

Supplement: Supplementary file 3 — Additional file 3: KEGG classification and functional maps of assembled contigs. Contigs annotated using KEGG Automatic Annotation Server identified sequences in a broad range of functional groups including developmental pathways and cell signaling. (ZIP 11 MB) [file 12864_2013_7026_MOESM3_ESM.zip › KEGG classification/map/map00640.png]

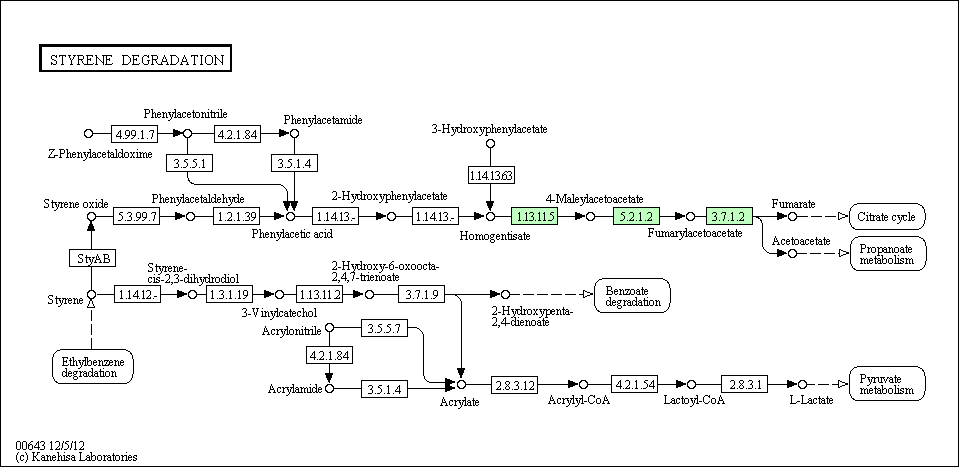

Supplement: Supplementary file 3 — Additional file 3: KEGG classification and functional maps of assembled contigs. Contigs annotated using KEGG Automatic Annotation Server identified sequences in a broad range of functional groups including developmental pathways and cell signaling. (ZIP 11 MB) [file 12864_2013_7026_MOESM3_ESM.zip › KEGG classification/map/map00643.png]

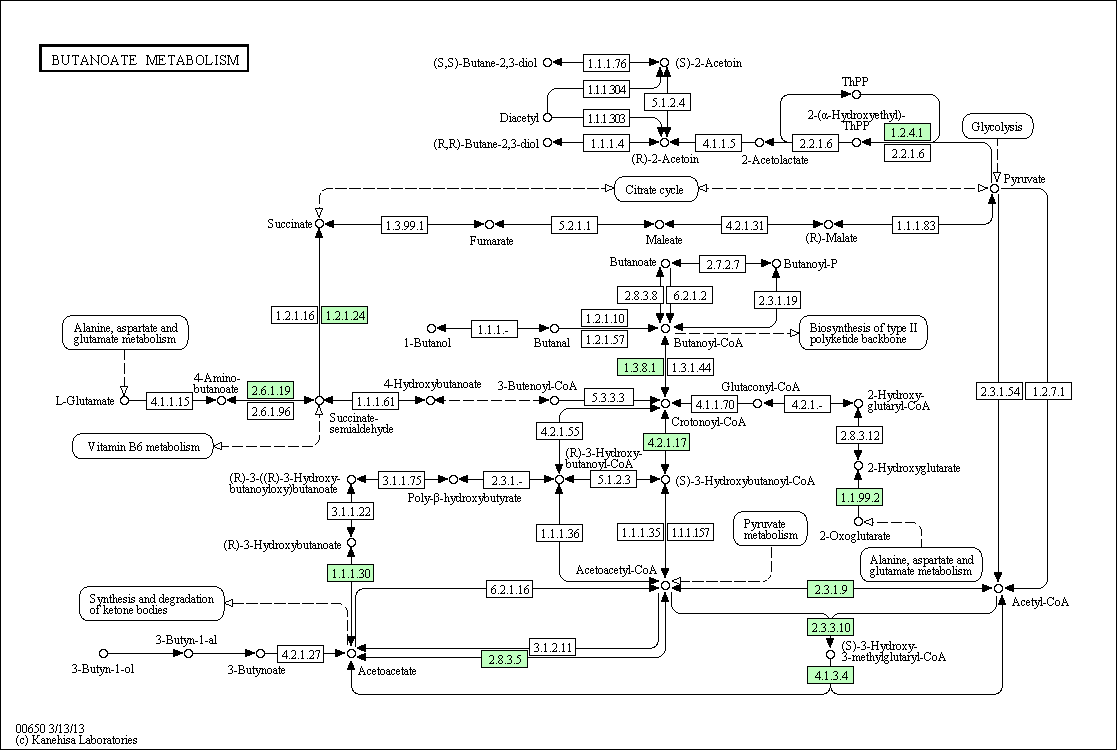

Supplement: Supplementary file 3 — Additional file 3: KEGG classification and functional maps of assembled contigs. Contigs annotated using KEGG Automatic Annotation Server identified sequences in a broad range of functional groups including developmental pathways and cell signaling. (ZIP 11 MB) [file 12864_2013_7026_MOESM3_ESM.zip › KEGG classification/map/map00650.png]

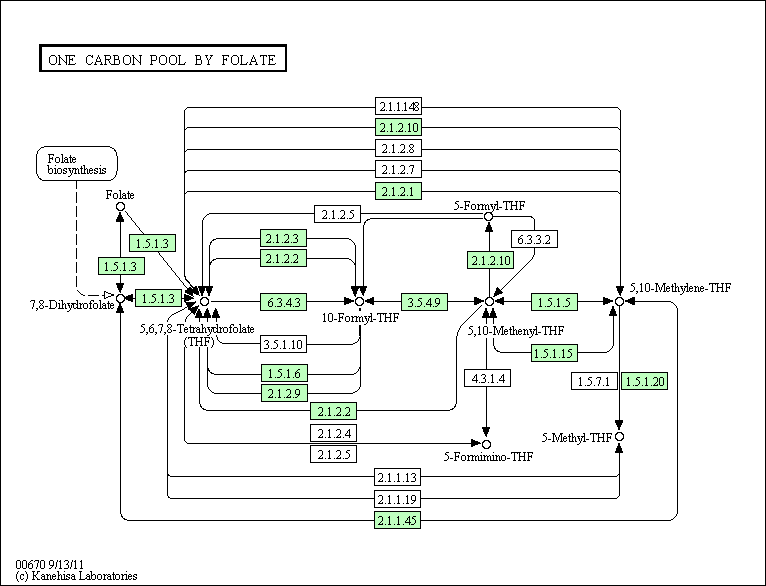

Supplement: Supplementary file 3 — Additional file 3: KEGG classification and functional maps of assembled contigs. Contigs annotated using KEGG Automatic Annotation Server identified sequences in a broad range of functional groups including developmental pathways and cell signaling. (ZIP 11 MB) [file 12864_2013_7026_MOESM3_ESM.zip › KEGG classification/map/map00670.png]

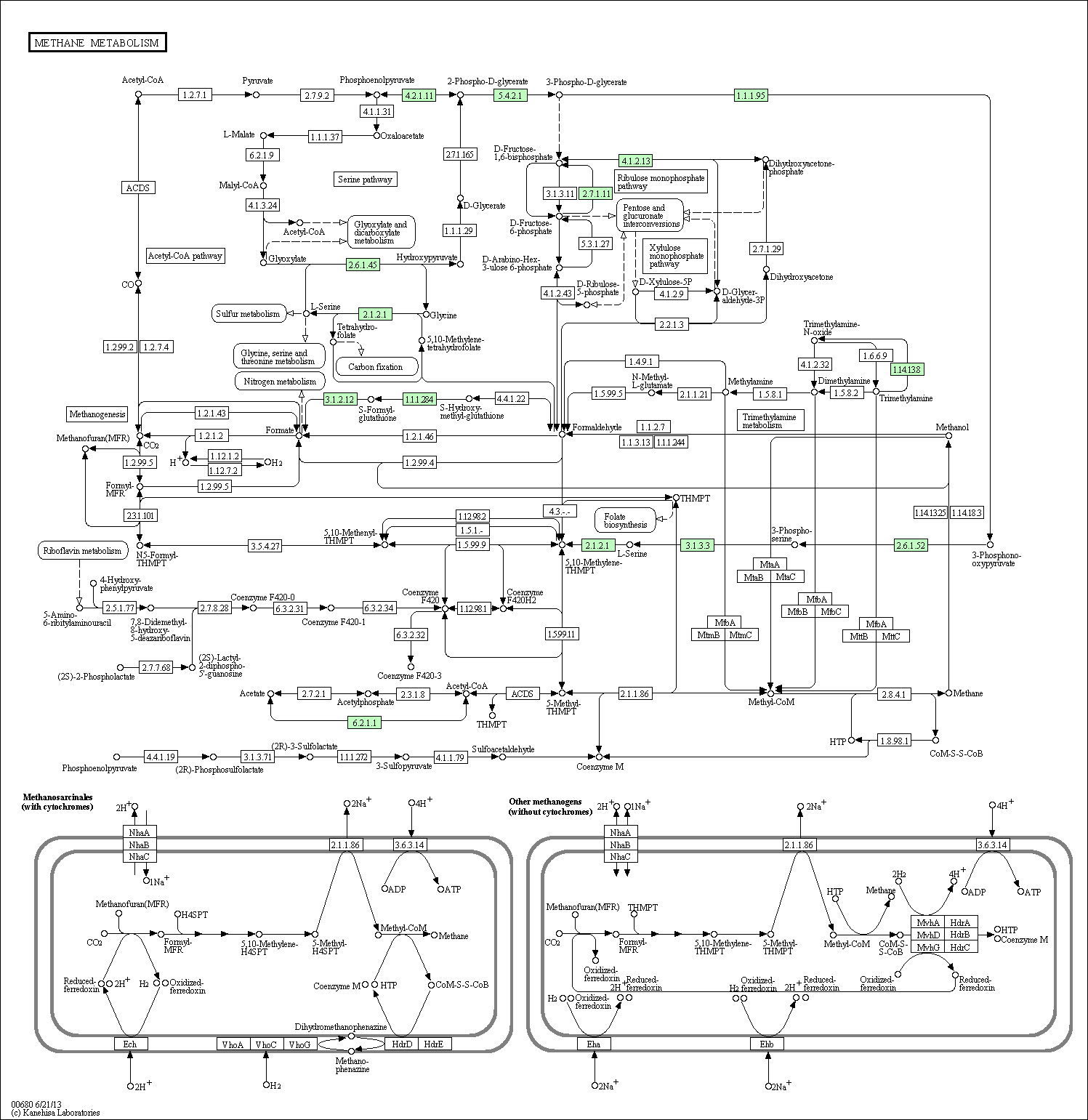

Supplement: Supplementary file 3 — Additional file 3: KEGG classification and functional maps of assembled contigs. Contigs annotated using KEGG Automatic Annotation Server identified sequences in a broad range of functional groups including developmental pathways and cell signaling. (ZIP 11 MB) [file 12864_2013_7026_MOESM3_ESM.zip › KEGG classification/map/map00680.png]

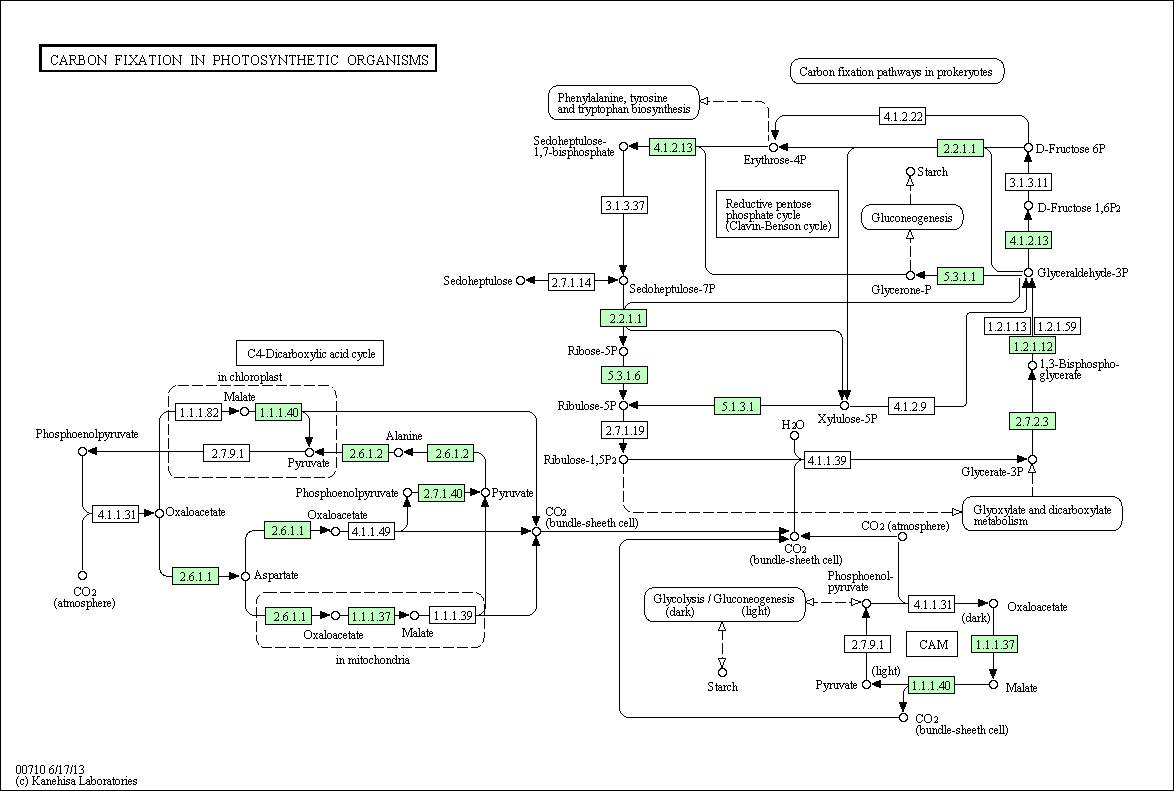

Supplement: Supplementary file 3 — Additional file 3: KEGG classification and functional maps of assembled contigs. Contigs annotated using KEGG Automatic Annotation Server identified sequences in a broad range of functional groups including developmental pathways and cell signaling. (ZIP 11 MB) [file 12864_2013_7026_MOESM3_ESM.zip › KEGG classification/map/map00710.png]

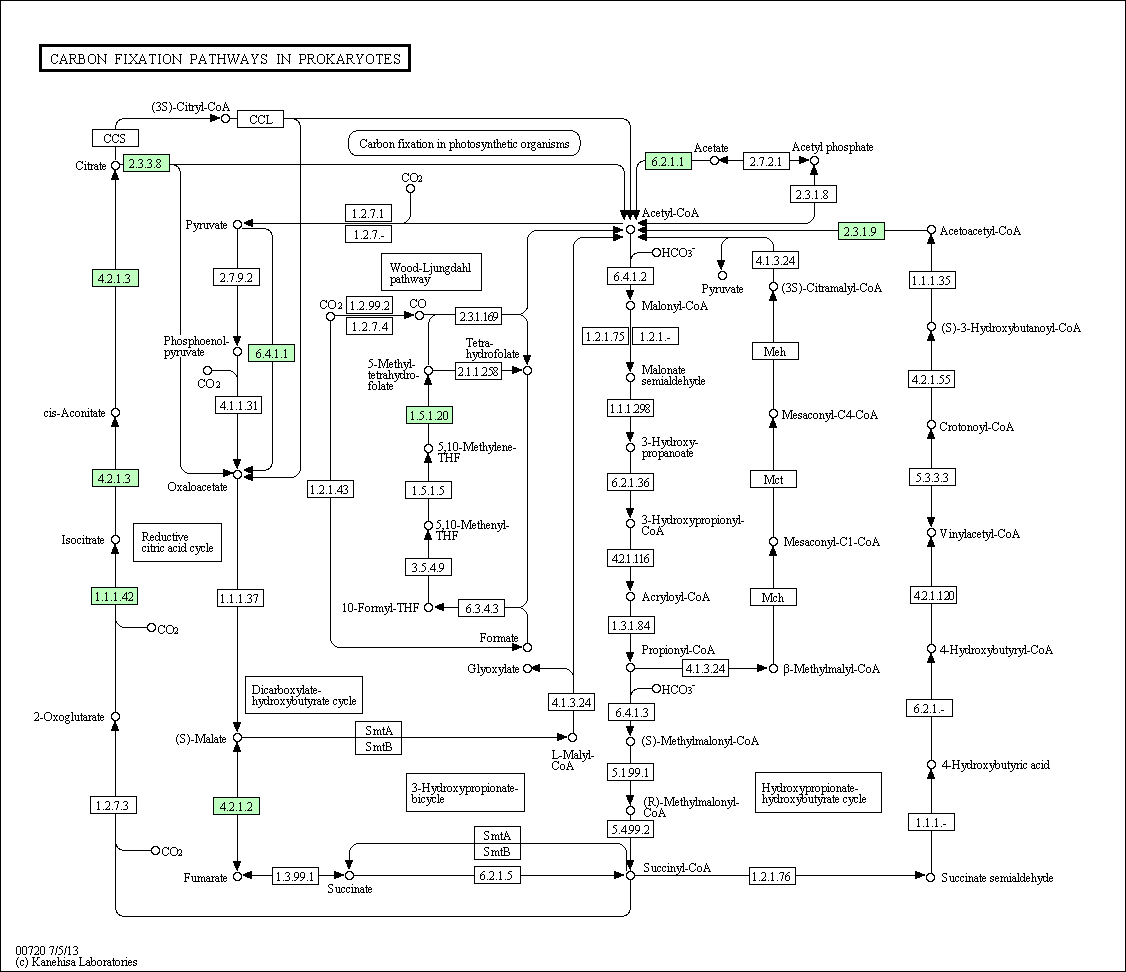

Supplement: Supplementary file 3 — Additional file 3: KEGG classification and functional maps of assembled contigs. Contigs annotated using KEGG Automatic Annotation Server identified sequences in a broad range of functional groups including developmental pathways and cell signaling. (ZIP 11 MB) [file 12864_2013_7026_MOESM3_ESM.zip › KEGG classification/map/map00720.png]

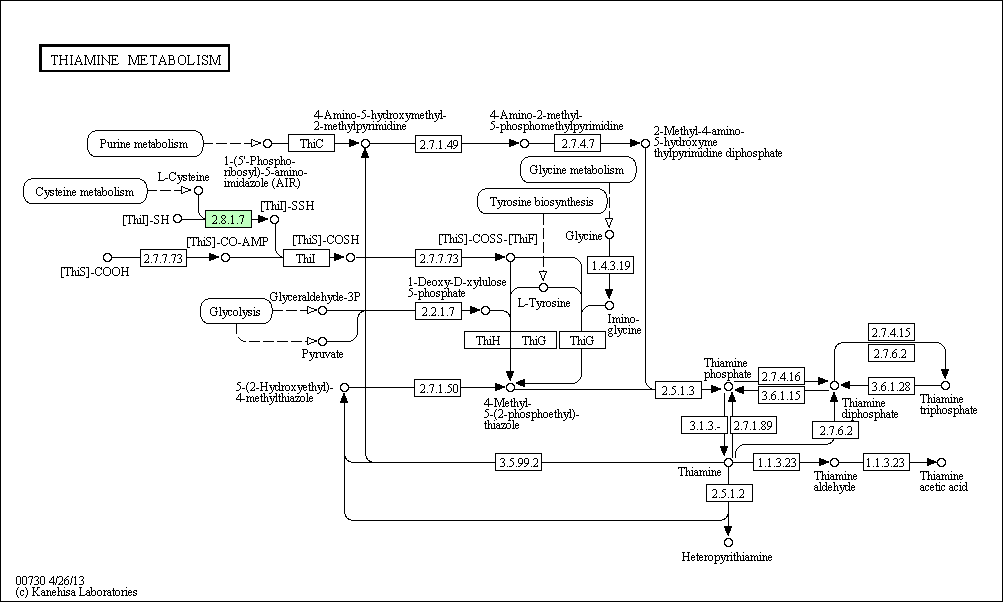

Supplement: Supplementary file 3 — Additional file 3: KEGG classification and functional maps of assembled contigs. Contigs annotated using KEGG Automatic Annotation Server identified sequences in a broad range of functional groups including developmental pathways and cell signaling. (ZIP 11 MB) [file 12864_2013_7026_MOESM3_ESM.zip › KEGG classification/map/map00730.png]

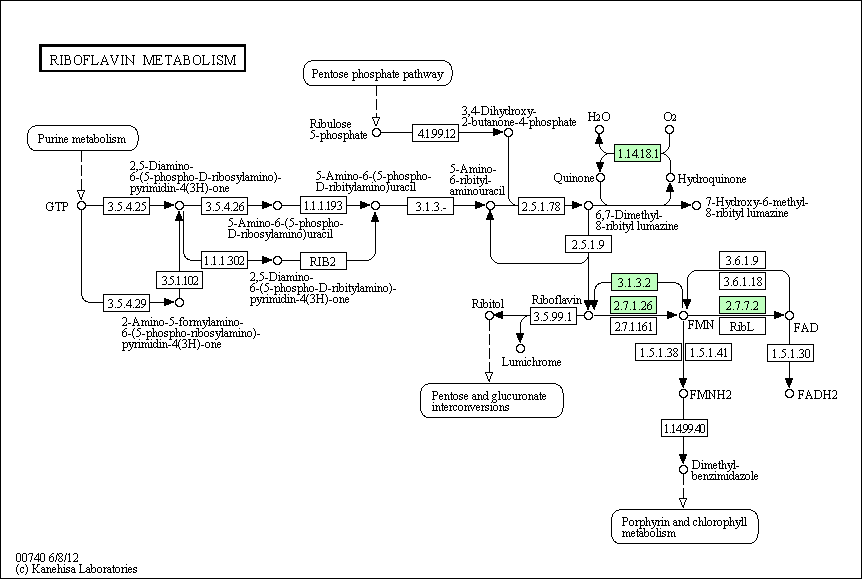

Supplement: Supplementary file 3 — Additional file 3: KEGG classification and functional maps of assembled contigs. Contigs annotated using KEGG Automatic Annotation Server identified sequences in a broad range of functional groups including developmental pathways and cell signaling. (ZIP 11 MB) [file 12864_2013_7026_MOESM3_ESM.zip › KEGG classification/map/map00740.png]

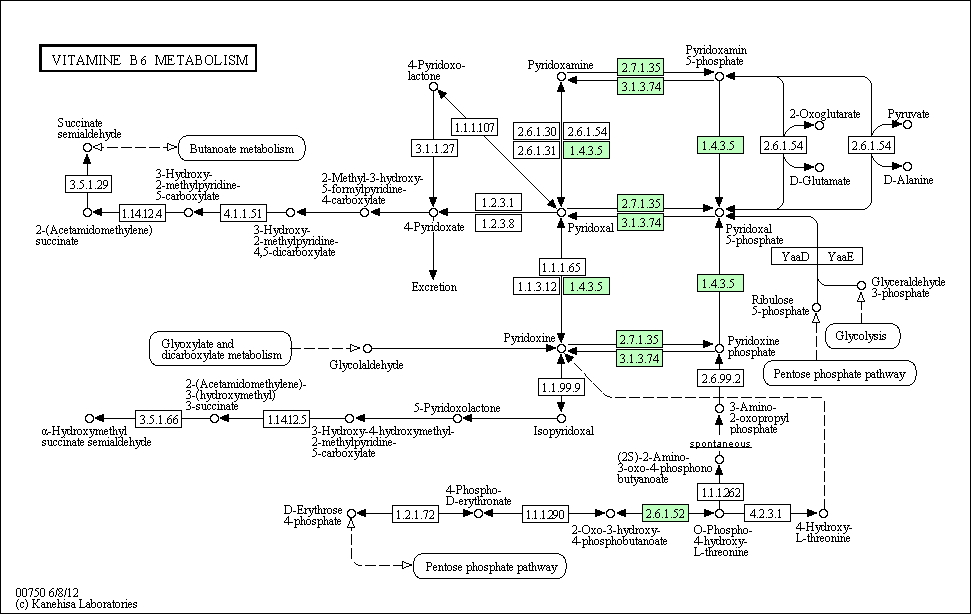

Supplement: Supplementary file 3 — Additional file 3: KEGG classification and functional maps of assembled contigs. Contigs annotated using KEGG Automatic Annotation Server identified sequences in a broad range of functional groups including developmental pathways and cell signaling. (ZIP 11 MB) [file 12864_2013_7026_MOESM3_ESM.zip › KEGG classification/map/map00750.png]

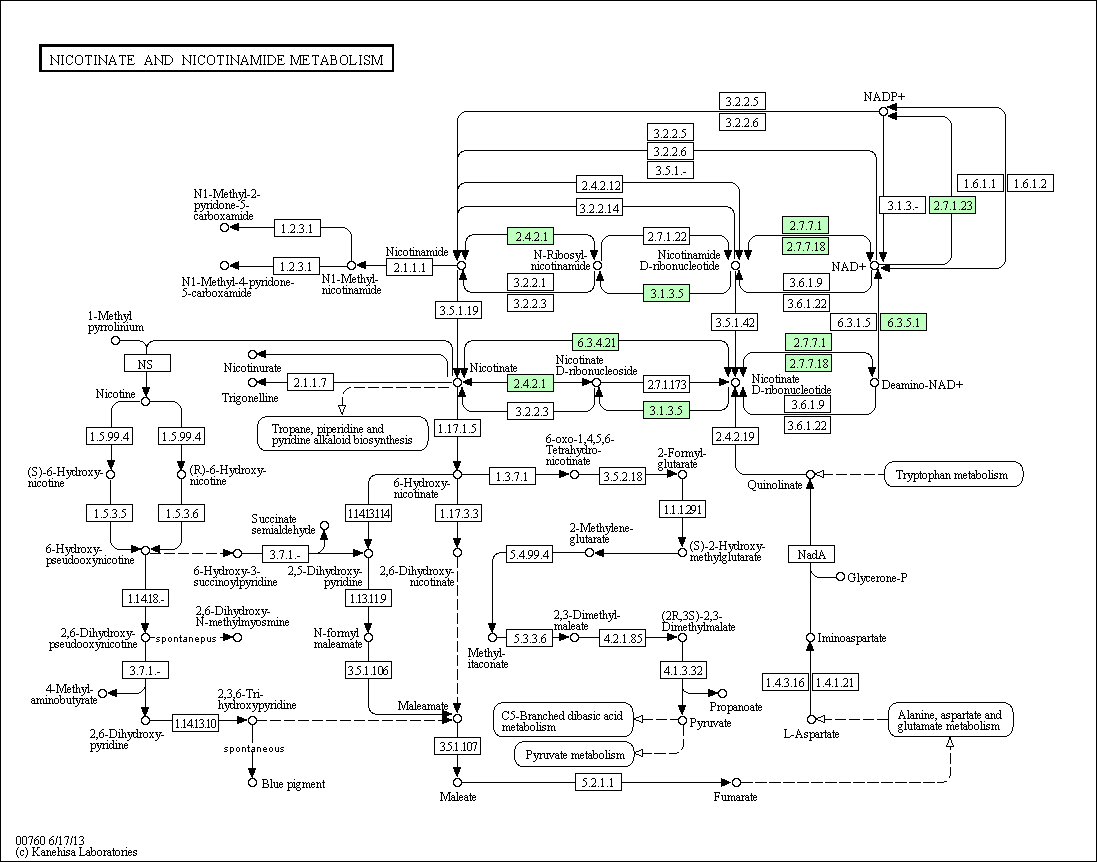

Supplement: Supplementary file 3 — Additional file 3: KEGG classification and functional maps of assembled contigs. Contigs annotated using KEGG Automatic Annotation Server identified sequences in a broad range of functional groups including developmental pathways and cell signaling. (ZIP 11 MB) [file 12864_2013_7026_MOESM3_ESM.zip › KEGG classification/map/map00760.png]

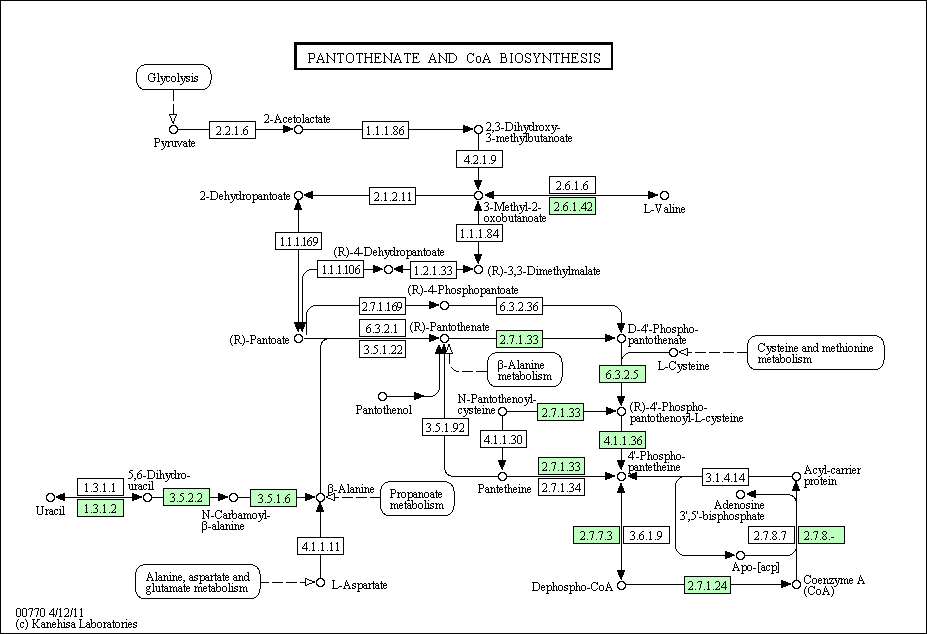

Supplement: Supplementary file 3 — Additional file 3: KEGG classification and functional maps of assembled contigs. Contigs annotated using KEGG Automatic Annotation Server identified sequences in a broad range of functional groups including developmental pathways and cell signaling. (ZIP 11 MB) [file 12864_2013_7026_MOESM3_ESM.zip › KEGG classification/map/map00770.png]

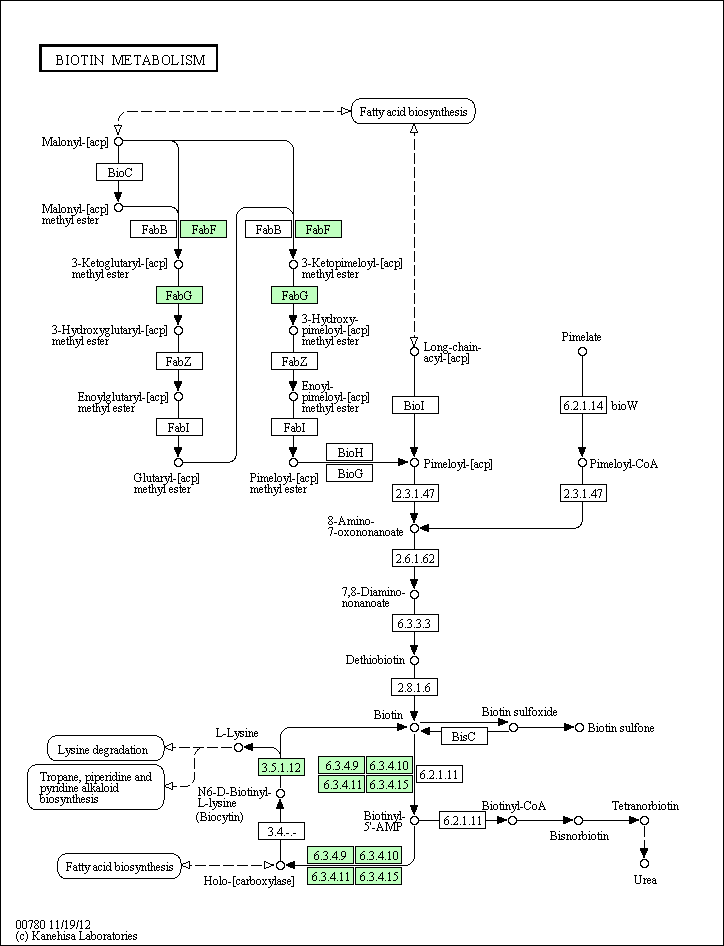

Supplement: Supplementary file 3 — Additional file 3: KEGG classification and functional maps of assembled contigs. Contigs annotated using KEGG Automatic Annotation Server identified sequences in a broad range of functional groups including developmental pathways and cell signaling. (ZIP 11 MB) [file 12864_2013_7026_MOESM3_ESM.zip › KEGG classification/map/map00780.png]

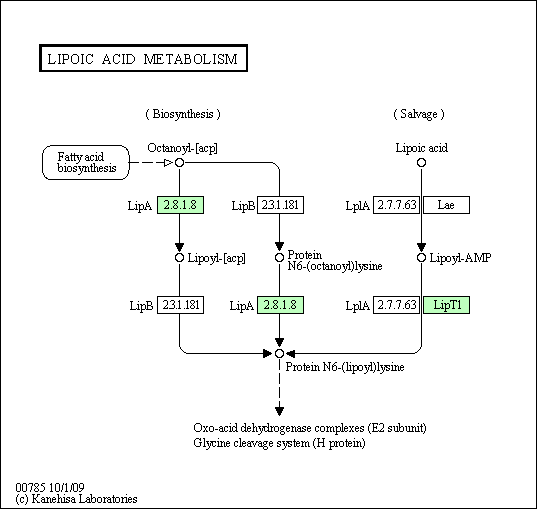

Supplement: Supplementary file 3 — Additional file 3: KEGG classification and functional maps of assembled contigs. Contigs annotated using KEGG Automatic Annotation Server identified sequences in a broad range of functional groups including developmental pathways and cell signaling. (ZIP 11 MB) [file 12864_2013_7026_MOESM3_ESM.zip › KEGG classification/map/map00785.png]

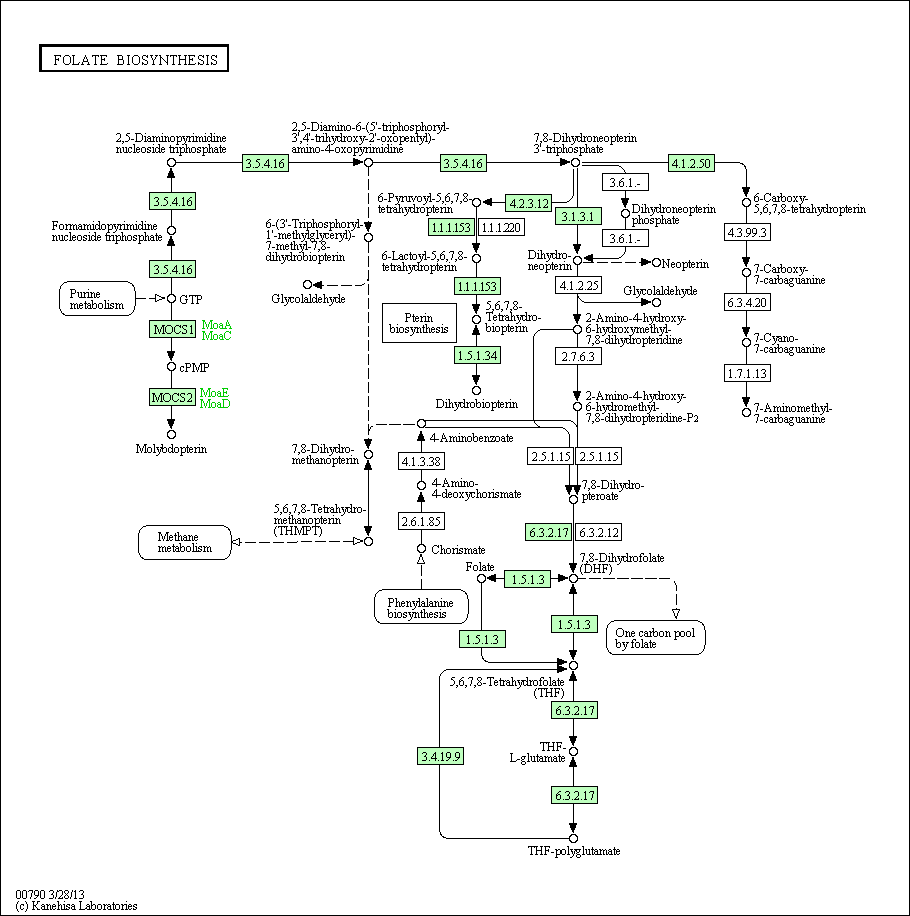

Supplement: Supplementary file 3 — Additional file 3: KEGG classification and functional maps of assembled contigs. Contigs annotated using KEGG Automatic Annotation Server identified sequences in a broad range of functional groups including developmental pathways and cell signaling. (ZIP 11 MB) [file 12864_2013_7026_MOESM3_ESM.zip › KEGG classification/map/map00790.png]

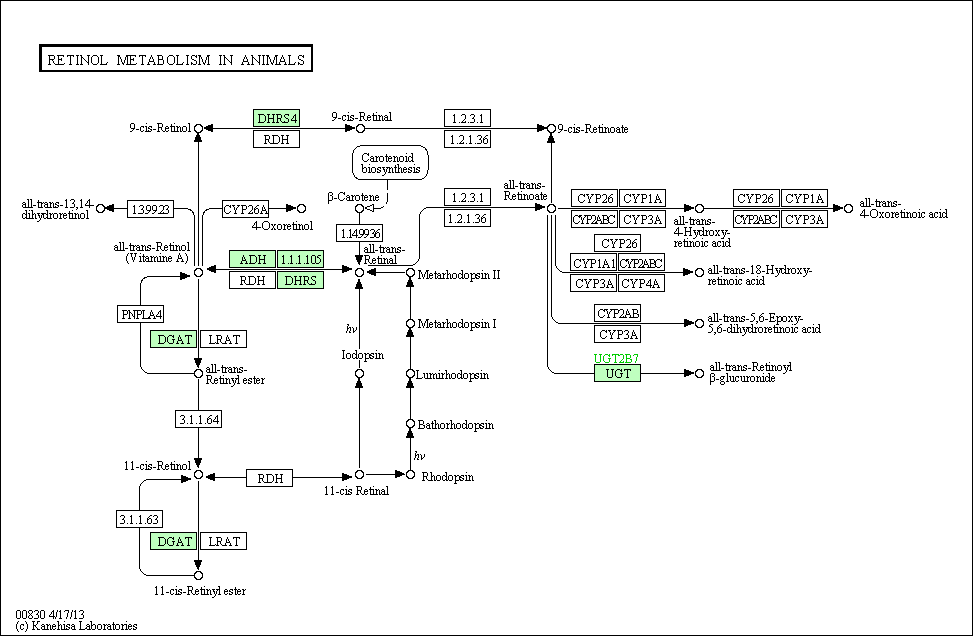

Supplement: Supplementary file 3 — Additional file 3: KEGG classification and functional maps of assembled contigs. Contigs annotated using KEGG Automatic Annotation Server identified sequences in a broad range of functional groups including developmental pathways and cell signaling. (ZIP 11 MB) [file 12864_2013_7026_MOESM3_ESM.zip › KEGG classification/map/map00830.png]

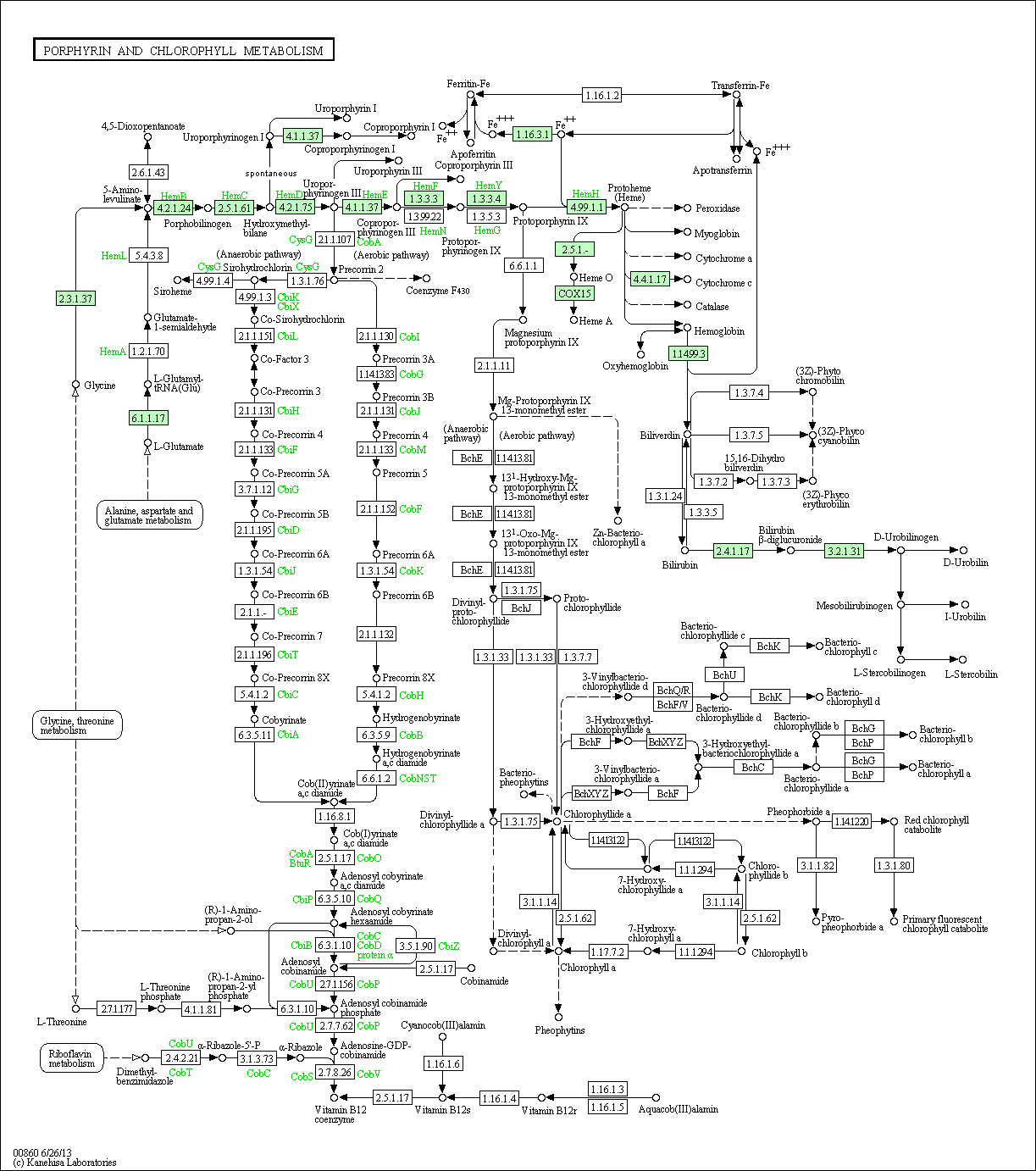

Supplement: Supplementary file 3 — Additional file 3: KEGG classification and functional maps of assembled contigs. Contigs annotated using KEGG Automatic Annotation Server identified sequences in a broad range of functional groups including developmental pathways and cell signaling. (ZIP 11 MB) [file 12864_2013_7026_MOESM3_ESM.zip › KEGG classification/map/map00860.png]

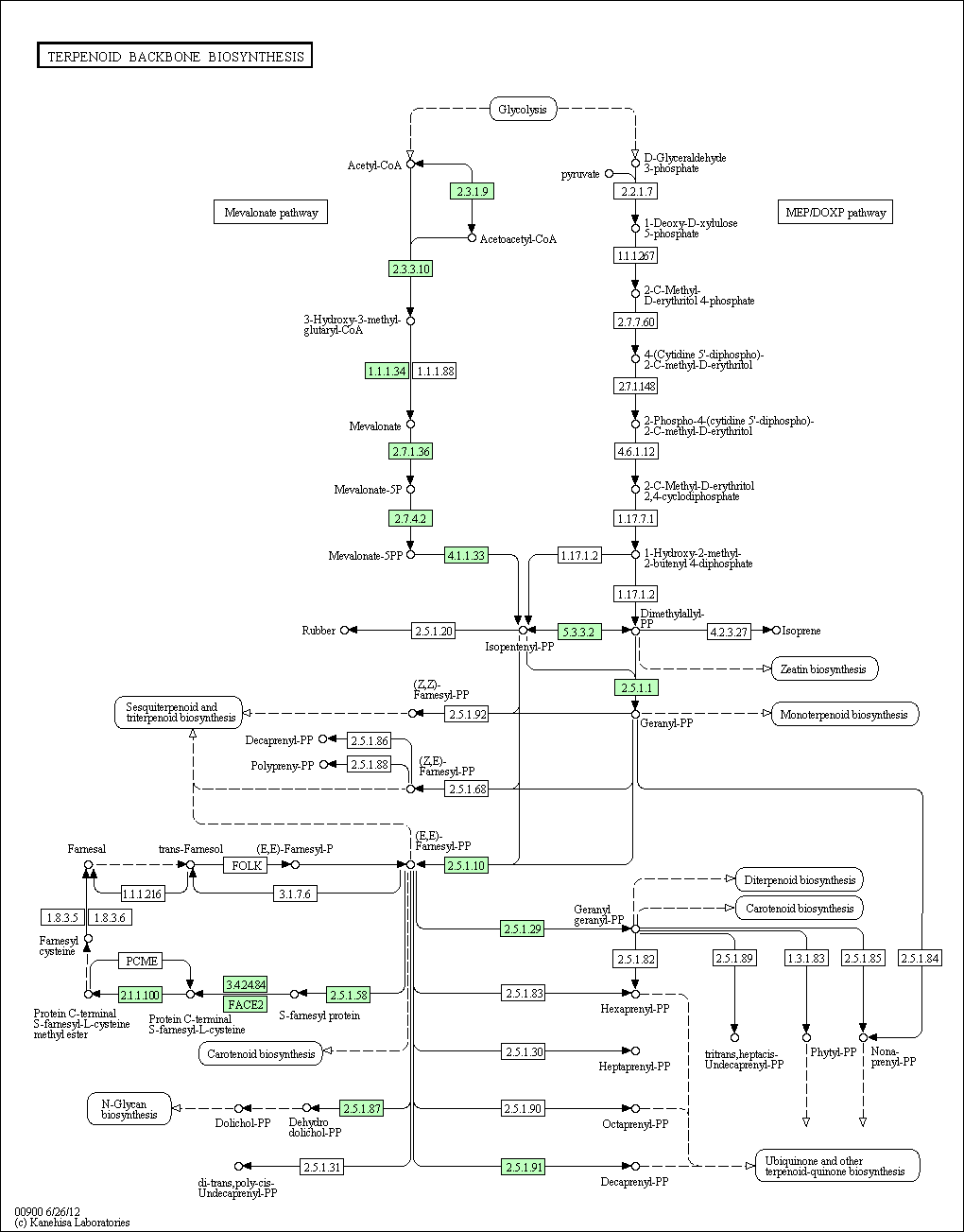

Supplement: Supplementary file 3 — Additional file 3: KEGG classification and functional maps of assembled contigs. Contigs annotated using KEGG Automatic Annotation Server identified sequences in a broad range of functional groups including developmental pathways and cell signaling. (ZIP 11 MB) [file 12864_2013_7026_MOESM3_ESM.zip › KEGG classification/map/map00900.png]

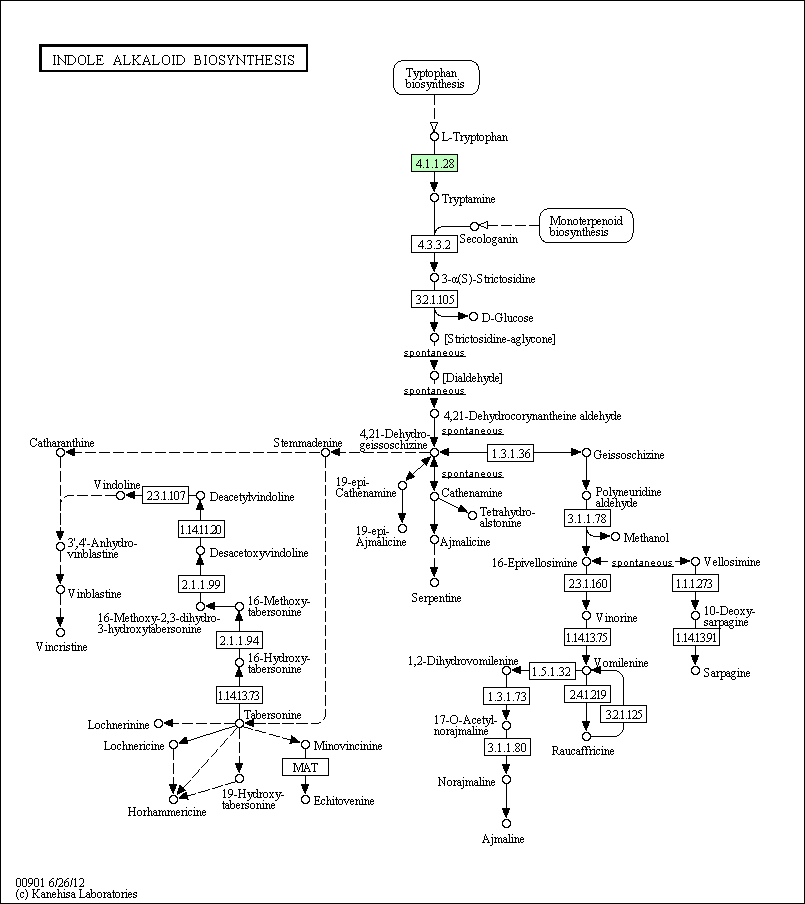

Supplement: Supplementary file 3 — Additional file 3: KEGG classification and functional maps of assembled contigs. Contigs annotated using KEGG Automatic Annotation Server identified sequences in a broad range of functional groups including developmental pathways and cell signaling. (ZIP 11 MB) [file 12864_2013_7026_MOESM3_ESM.zip › KEGG classification/map/map00901.png]

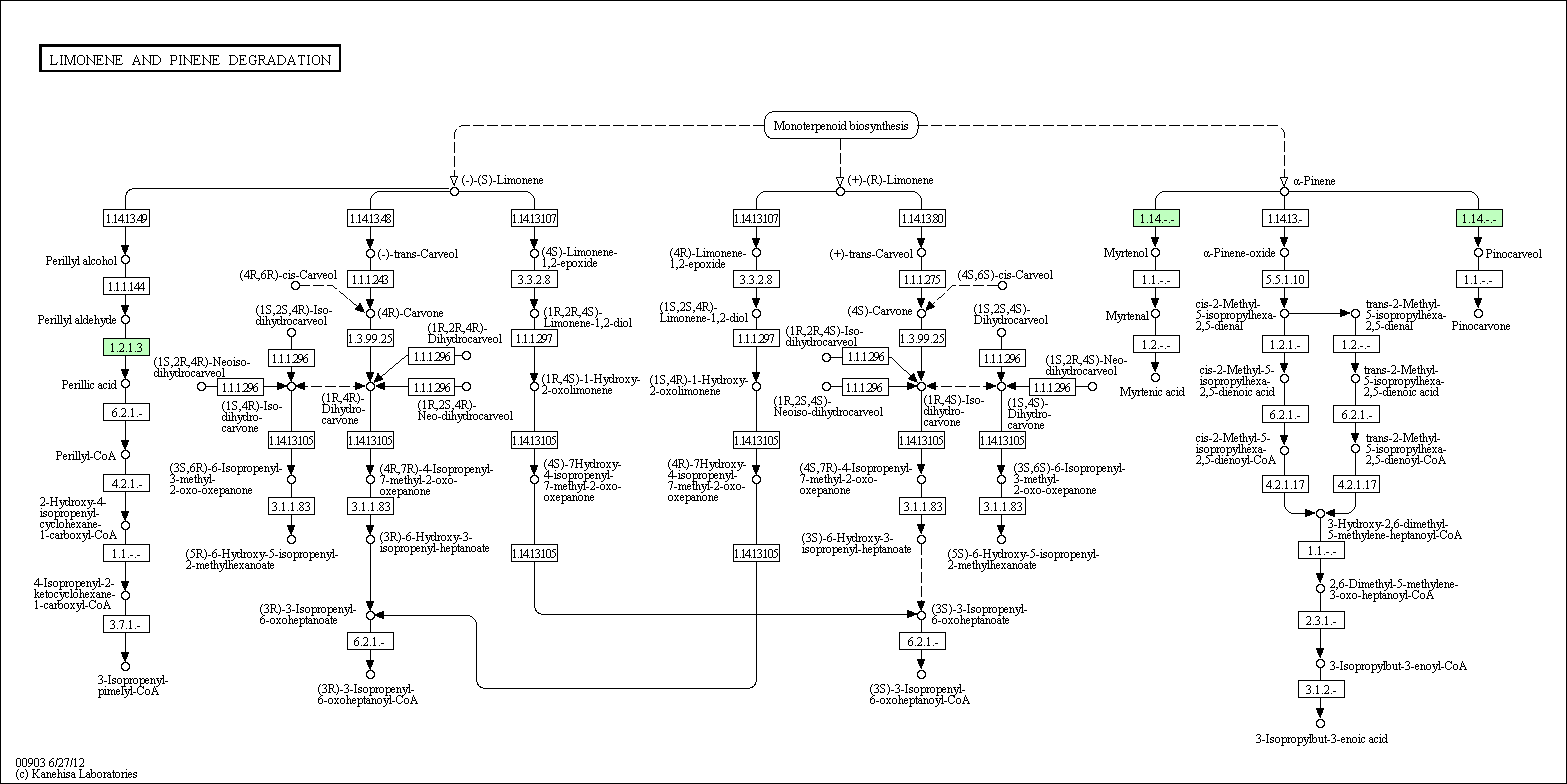

Supplement: Supplementary file 3 — Additional file 3: KEGG classification and functional maps of assembled contigs. Contigs annotated using KEGG Automatic Annotation Server identified sequences in a broad range of functional groups including developmental pathways and cell signaling. (ZIP 11 MB) [file 12864_2013_7026_MOESM3_ESM.zip › KEGG classification/map/map00903.png]

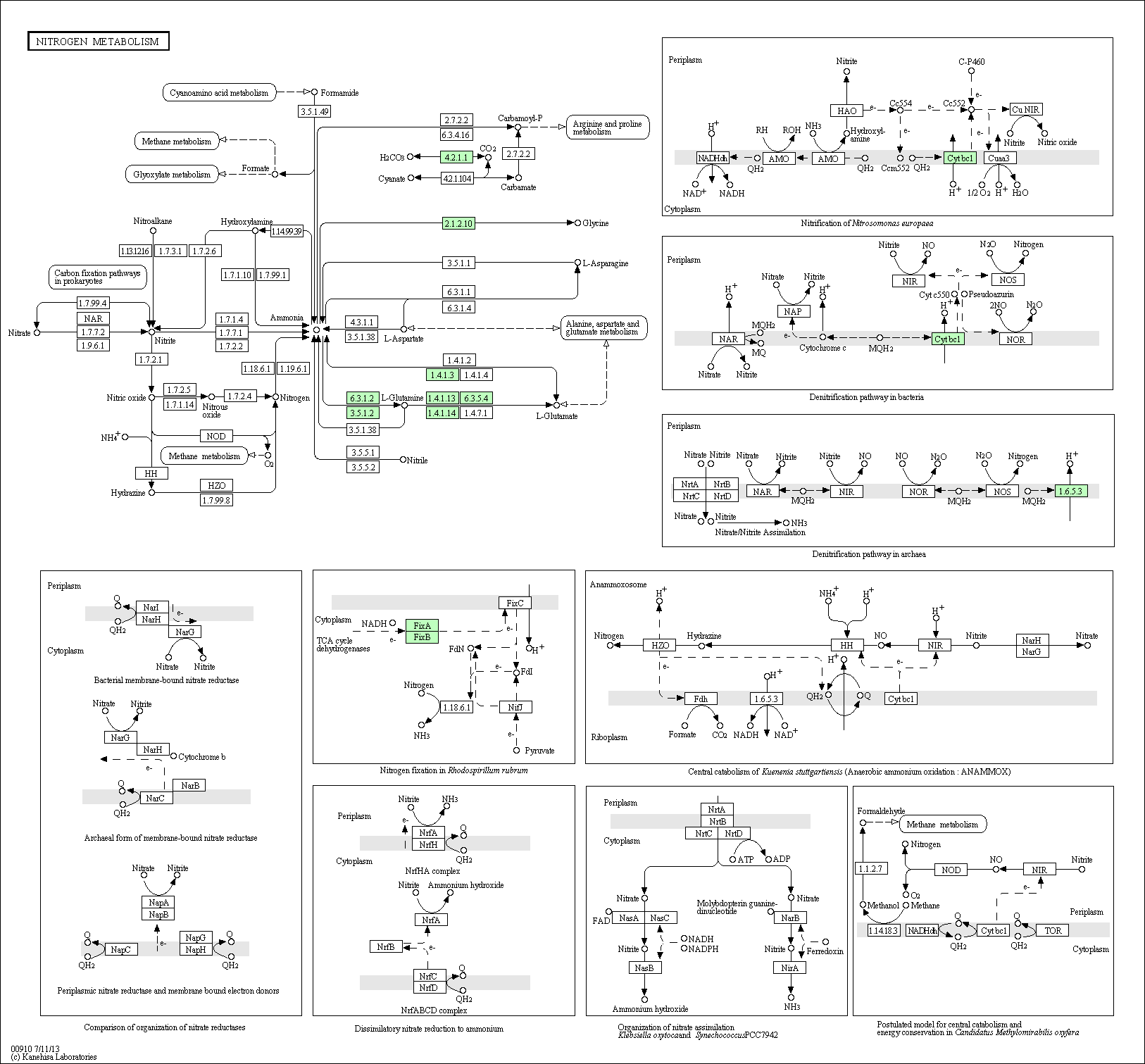

Supplement: Supplementary file 3 — Additional file 3: KEGG classification and functional maps of assembled contigs. Contigs annotated using KEGG Automatic Annotation Server identified sequences in a broad range of functional groups including developmental pathways and cell signaling. (ZIP 11 MB) [file 12864_2013_7026_MOESM3_ESM.zip › KEGG classification/map/map00910.png]

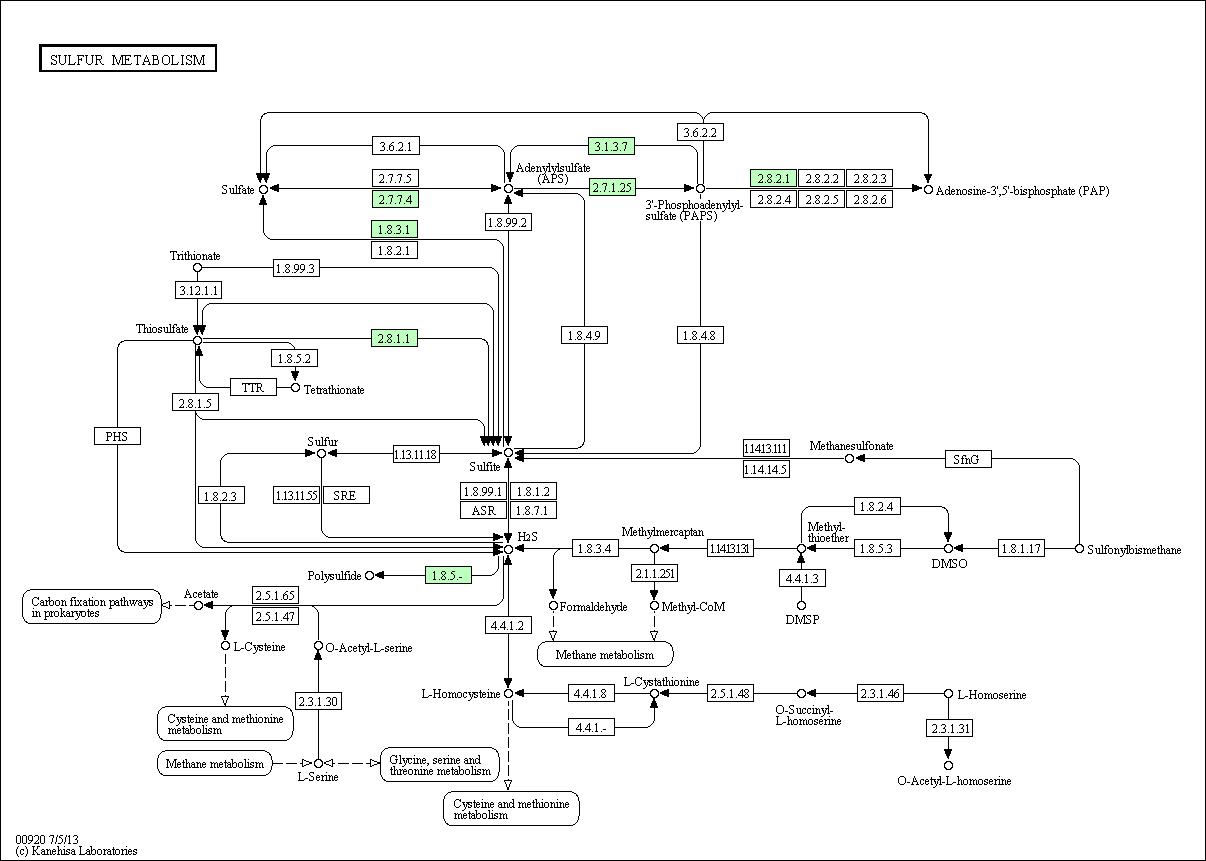

Supplement: Supplementary file 3 — Additional file 3: KEGG classification and functional maps of assembled contigs. Contigs annotated using KEGG Automatic Annotation Server identified sequences in a broad range of functional groups including developmental pathways and cell signaling. (ZIP 11 MB) [file 12864_2013_7026_MOESM3_ESM.zip › KEGG classification/map/map00920.png]

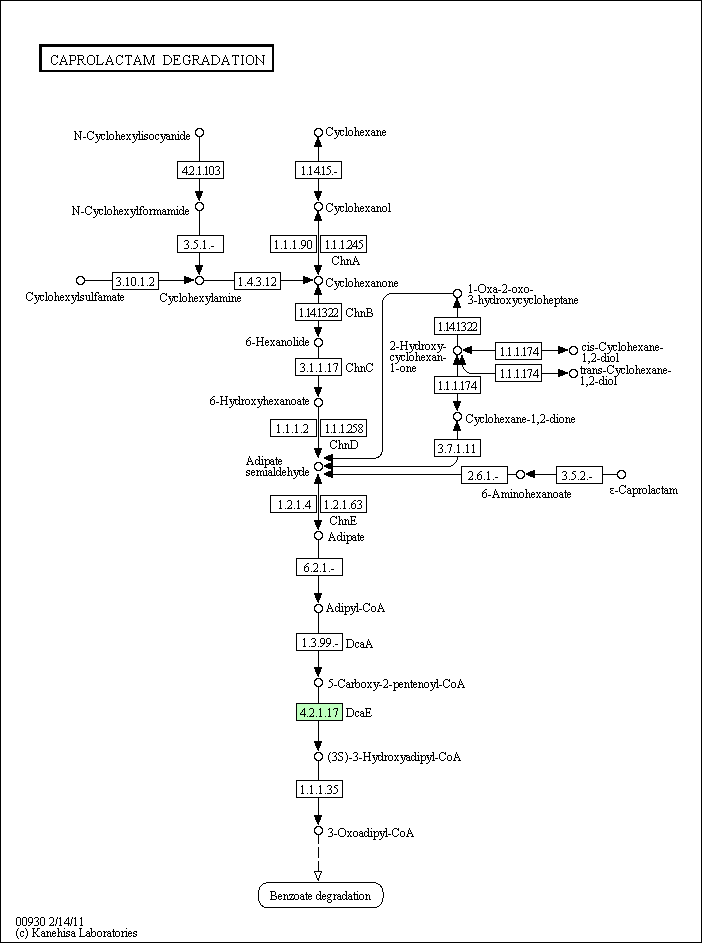

Supplement: Supplementary file 3 — Additional file 3: KEGG classification and functional maps of assembled contigs. Contigs annotated using KEGG Automatic Annotation Server identified sequences in a broad range of functional groups including developmental pathways and cell signaling. (ZIP 11 MB) [file 12864_2013_7026_MOESM3_ESM.zip › KEGG classification/map/map00930.png]

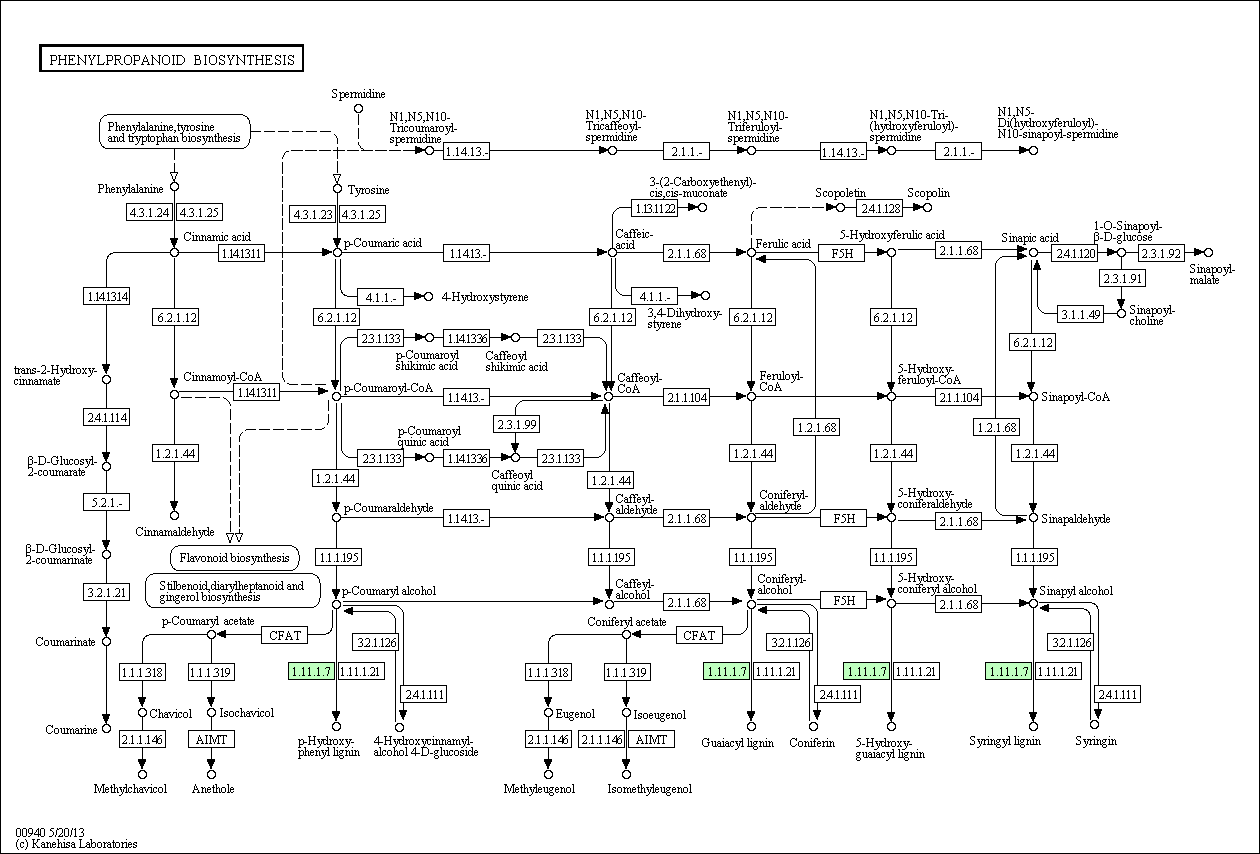

Supplement: Supplementary file 3 — Additional file 3: KEGG classification and functional maps of assembled contigs. Contigs annotated using KEGG Automatic Annotation Server identified sequences in a broad range of functional groups including developmental pathways and cell signaling. (ZIP 11 MB) [file 12864_2013_7026_MOESM3_ESM.zip › KEGG classification/map/map00940.png]

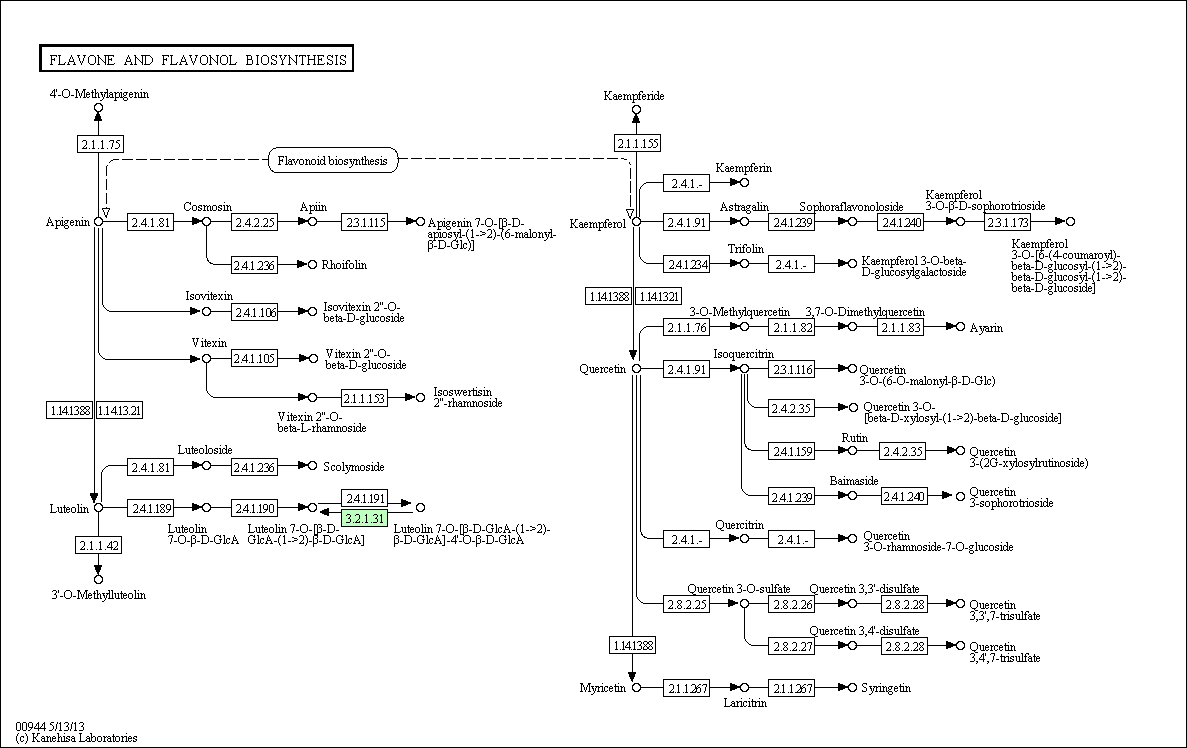

Supplement: Supplementary file 3 — Additional file 3: KEGG classification and functional maps of assembled contigs. Contigs annotated using KEGG Automatic Annotation Server identified sequences in a broad range of functional groups including developmental pathways and cell signaling. (ZIP 11 MB) [file 12864_2013_7026_MOESM3_ESM.zip › KEGG classification/map/map00944.png]

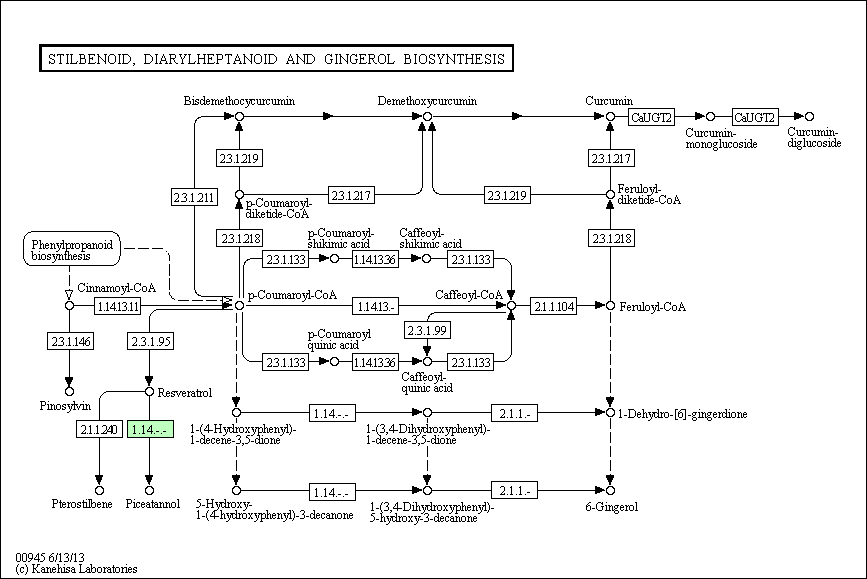

Supplement: Supplementary file 3 — Additional file 3: KEGG classification and functional maps of assembled contigs. Contigs annotated using KEGG Automatic Annotation Server identified sequences in a broad range of functional groups including developmental pathways and cell signaling. (ZIP 11 MB) [file 12864_2013_7026_MOESM3_ESM.zip › KEGG classification/map/map00945.png]

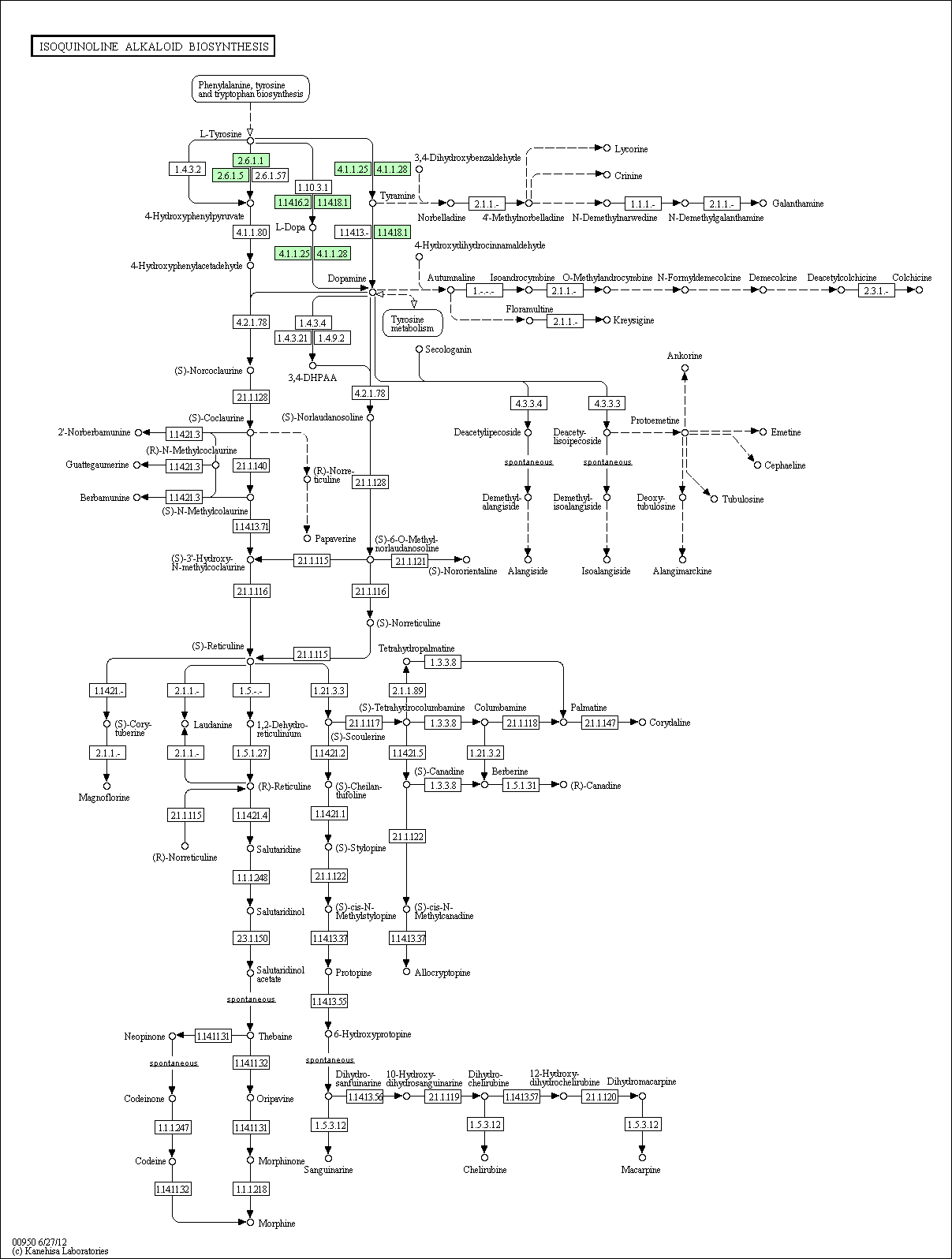

Supplement: Supplementary file 3 — Additional file 3: KEGG classification and functional maps of assembled contigs. Contigs annotated using KEGG Automatic Annotation Server identified sequences in a broad range of functional groups including developmental pathways and cell signaling. (ZIP 11 MB) [file 12864_2013_7026_MOESM3_ESM.zip › KEGG classification/map/map00950.png]

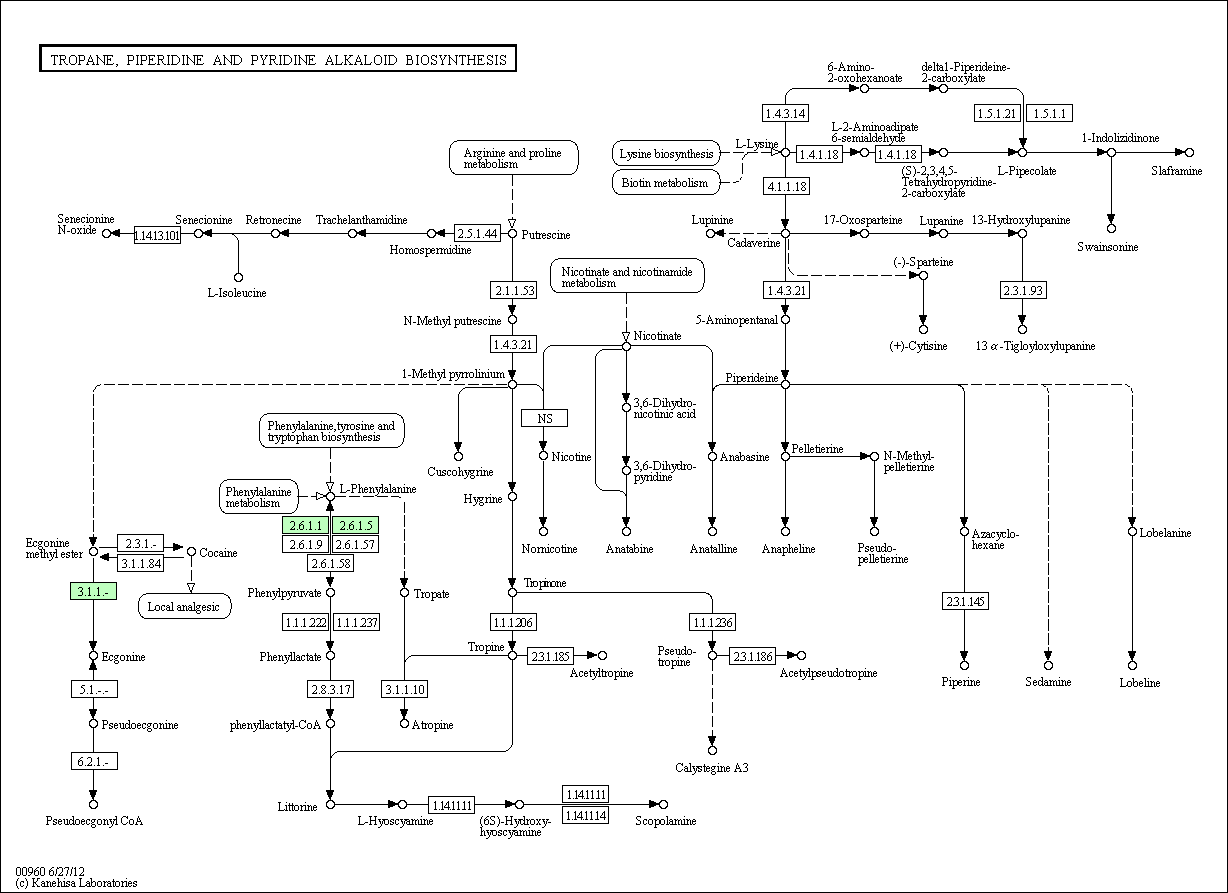

Supplement: Supplementary file 3 — Additional file 3: KEGG classification and functional maps of assembled contigs. Contigs annotated using KEGG Automatic Annotation Server identified sequences in a broad range of functional groups including developmental pathways and cell signaling. (ZIP 11 MB) [file 12864_2013_7026_MOESM3_ESM.zip › KEGG classification/map/map00960.png]

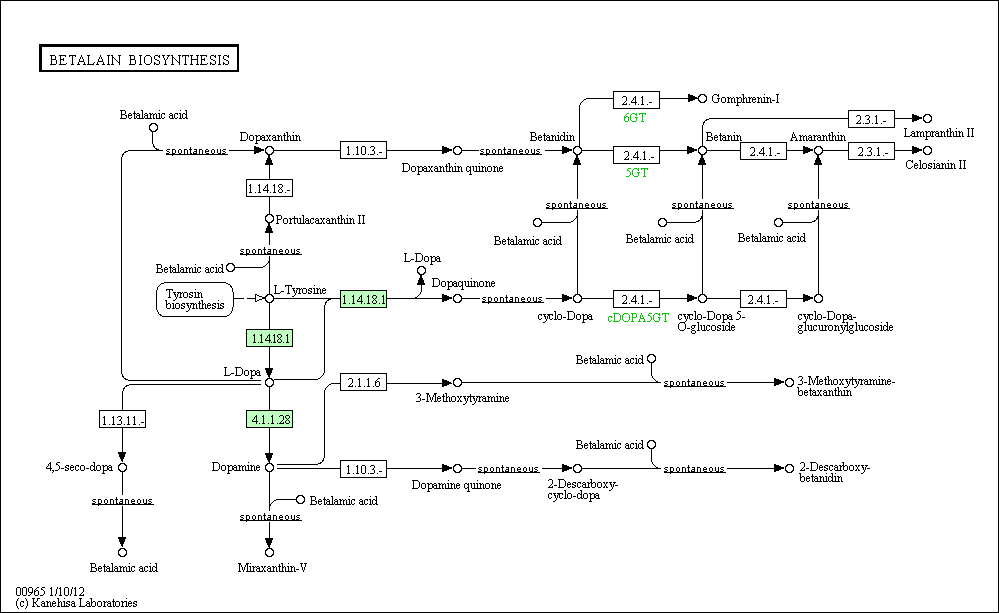

Supplement: Supplementary file 3 — Additional file 3: KEGG classification and functional maps of assembled contigs. Contigs annotated using KEGG Automatic Annotation Server identified sequences in a broad range of functional groups including developmental pathways and cell signaling. (ZIP 11 MB) [file 12864_2013_7026_MOESM3_ESM.zip › KEGG classification/map/map00965.png]

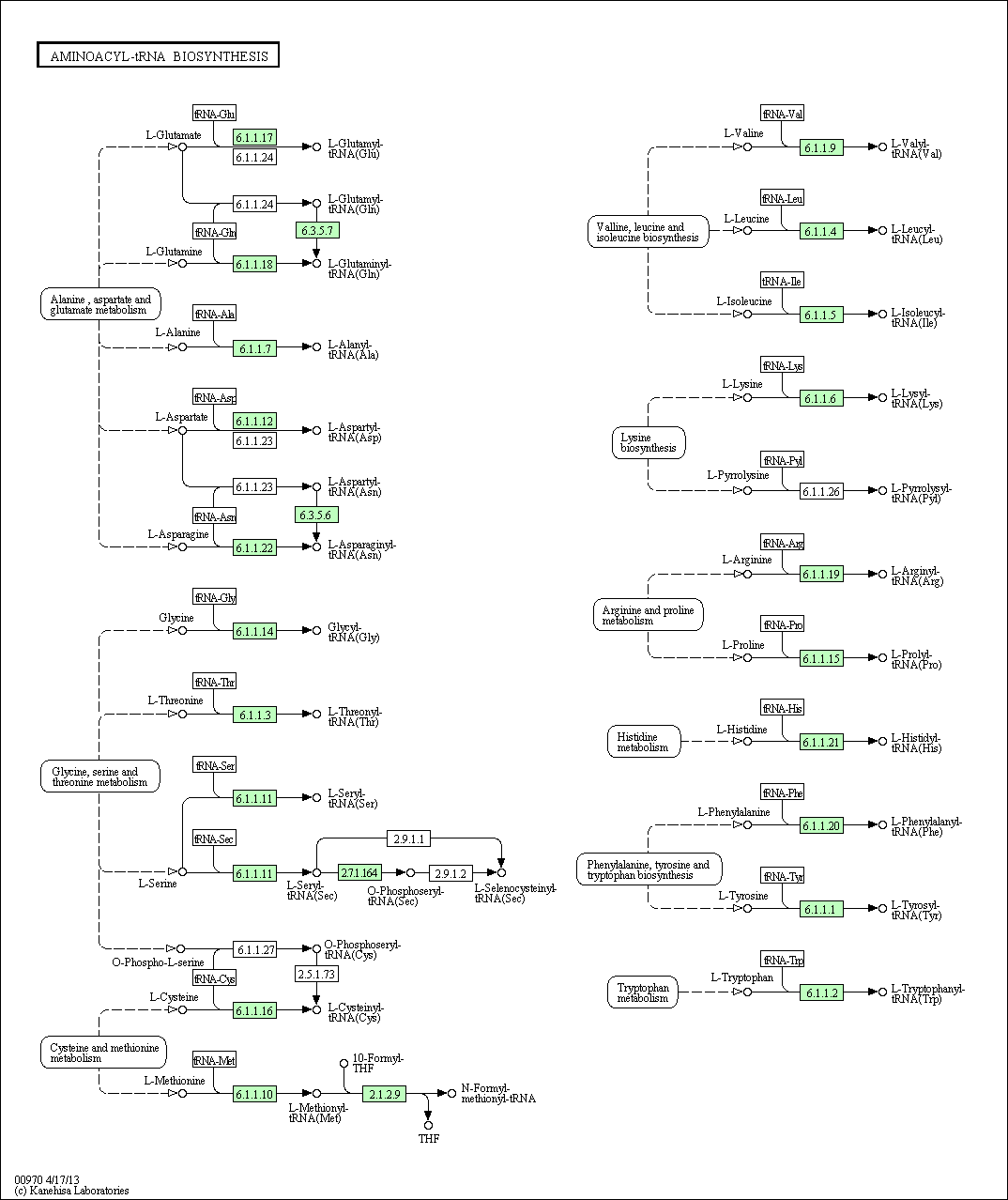

Supplement: Supplementary file 3 — Additional file 3: KEGG classification and functional maps of assembled contigs. Contigs annotated using KEGG Automatic Annotation Server identified sequences in a broad range of functional groups including developmental pathways and cell signaling. (ZIP 11 MB) [file 12864_2013_7026_MOESM3_ESM.zip › KEGG classification/map/map00970.png]

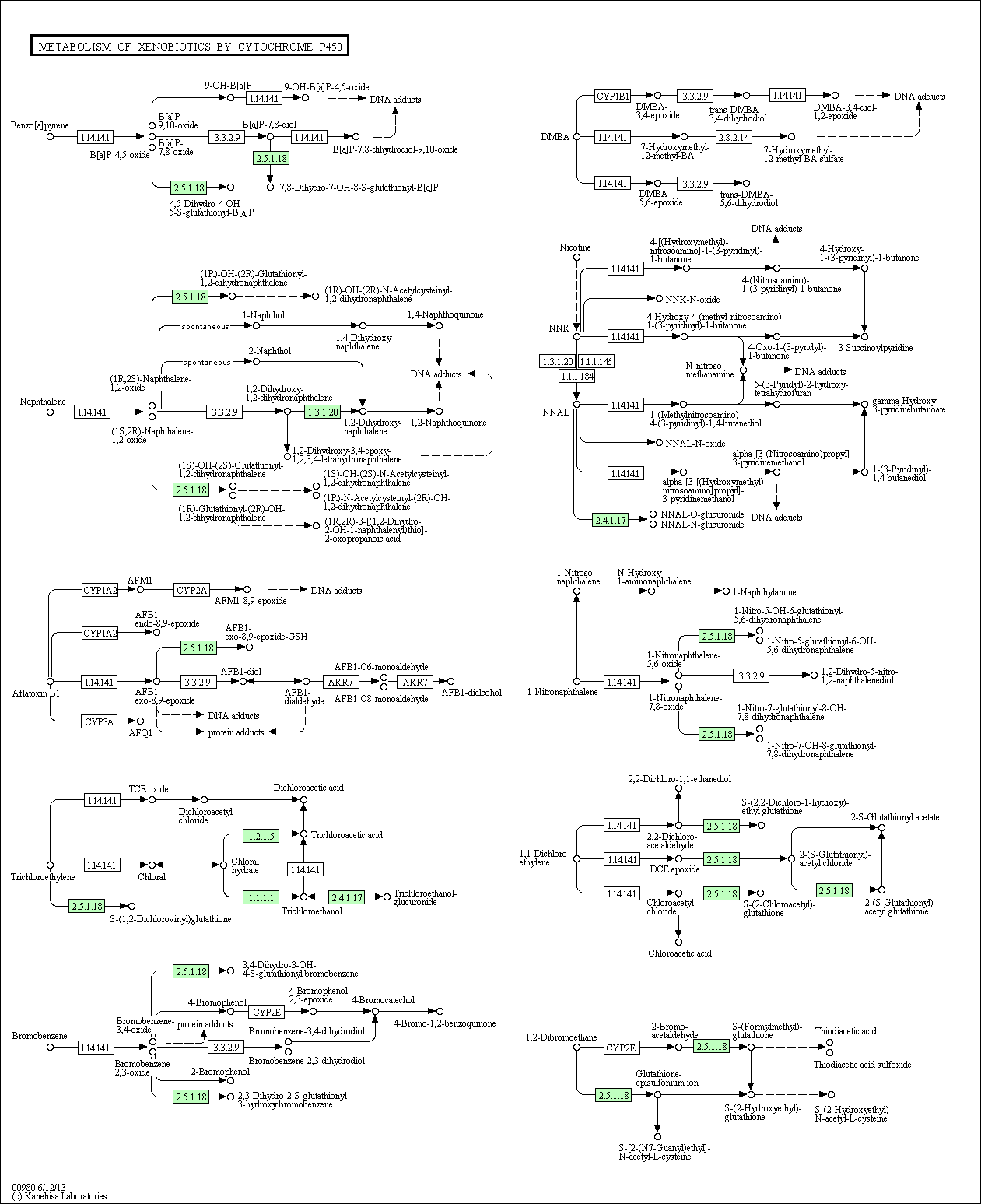

Supplement: Supplementary file 3 — Additional file 3: KEGG classification and functional maps of assembled contigs. Contigs annotated using KEGG Automatic Annotation Server identified sequences in a broad range of functional groups including developmental pathways and cell signaling. (ZIP 11 MB) [file 12864_2013_7026_MOESM3_ESM.zip › KEGG classification/map/map00980.png]

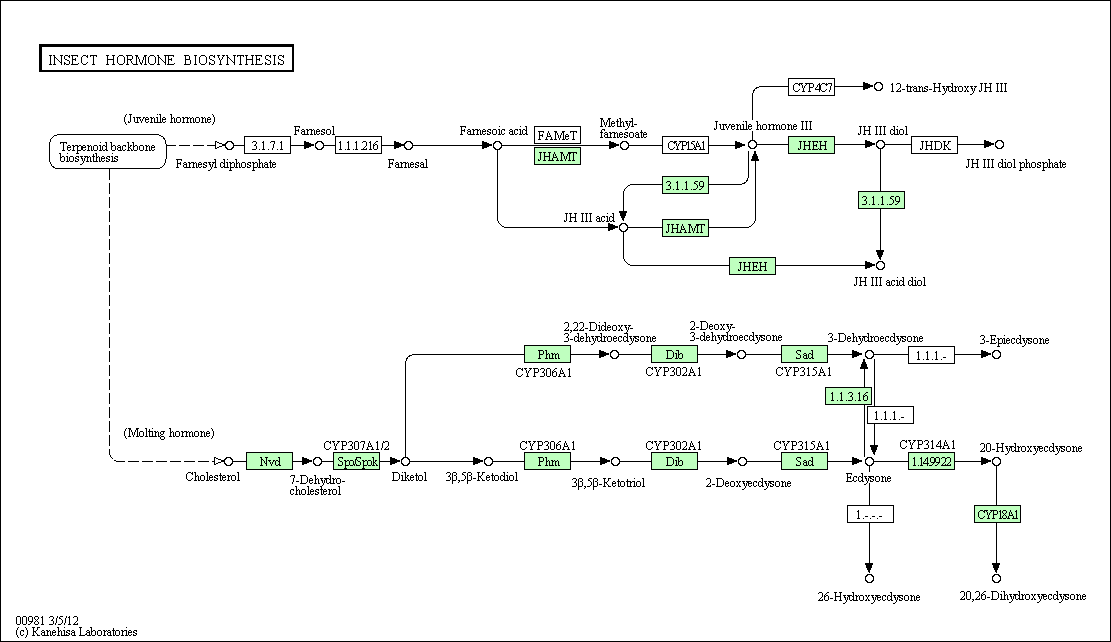

Supplement: Supplementary file 3 — Additional file 3: KEGG classification and functional maps of assembled contigs. Contigs annotated using KEGG Automatic Annotation Server identified sequences in a broad range of functional groups including developmental pathways and cell signaling. (ZIP 11 MB) [file 12864_2013_7026_MOESM3_ESM.zip › KEGG classification/map/map00981.png]

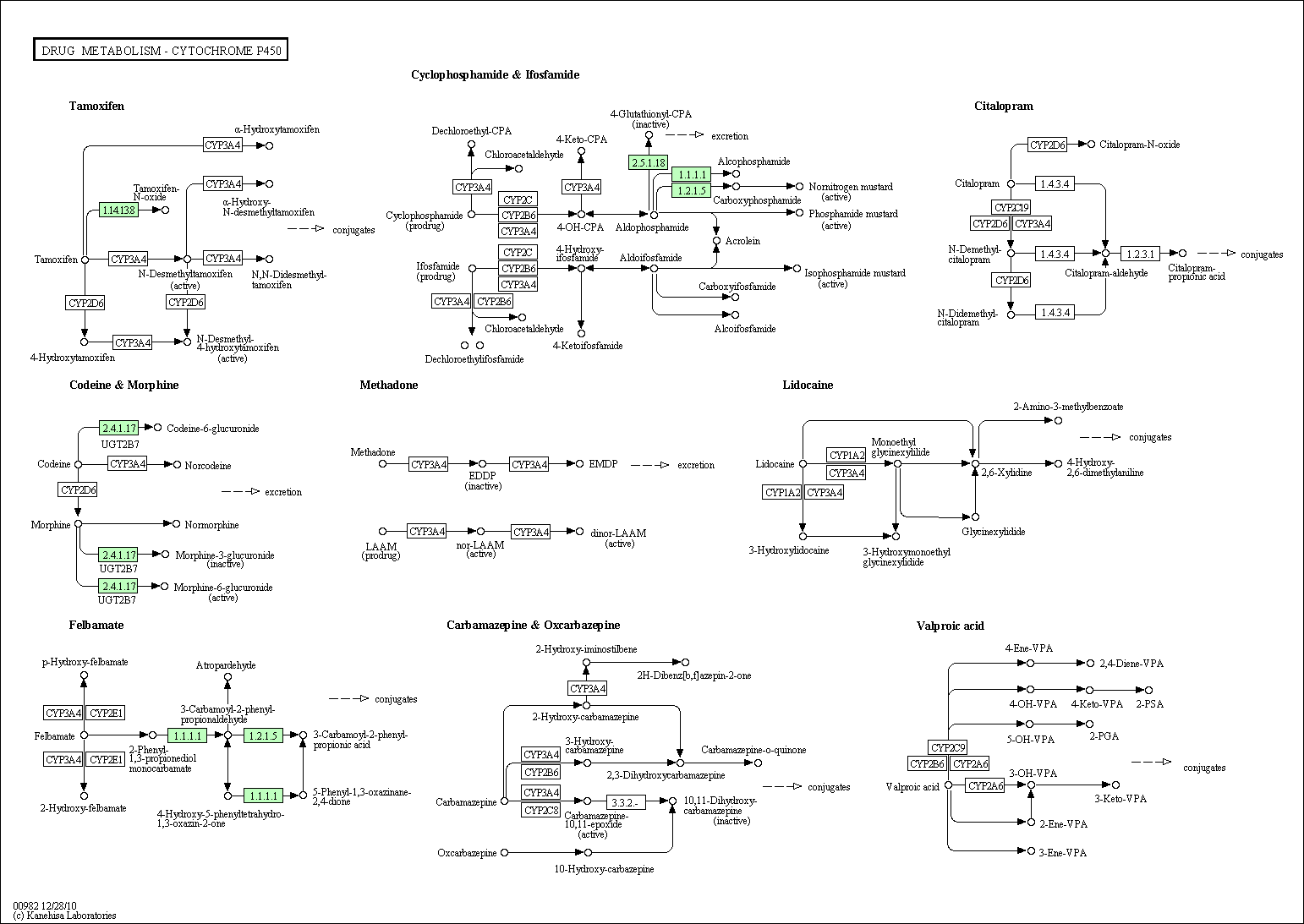

Supplement: Supplementary file 3 — Additional file 3: KEGG classification and functional maps of assembled contigs. Contigs annotated using KEGG Automatic Annotation Server identified sequences in a broad range of functional groups including developmental pathways and cell signaling. (ZIP 11 MB) [file 12864_2013_7026_MOESM3_ESM.zip › KEGG classification/map/map00982.png]

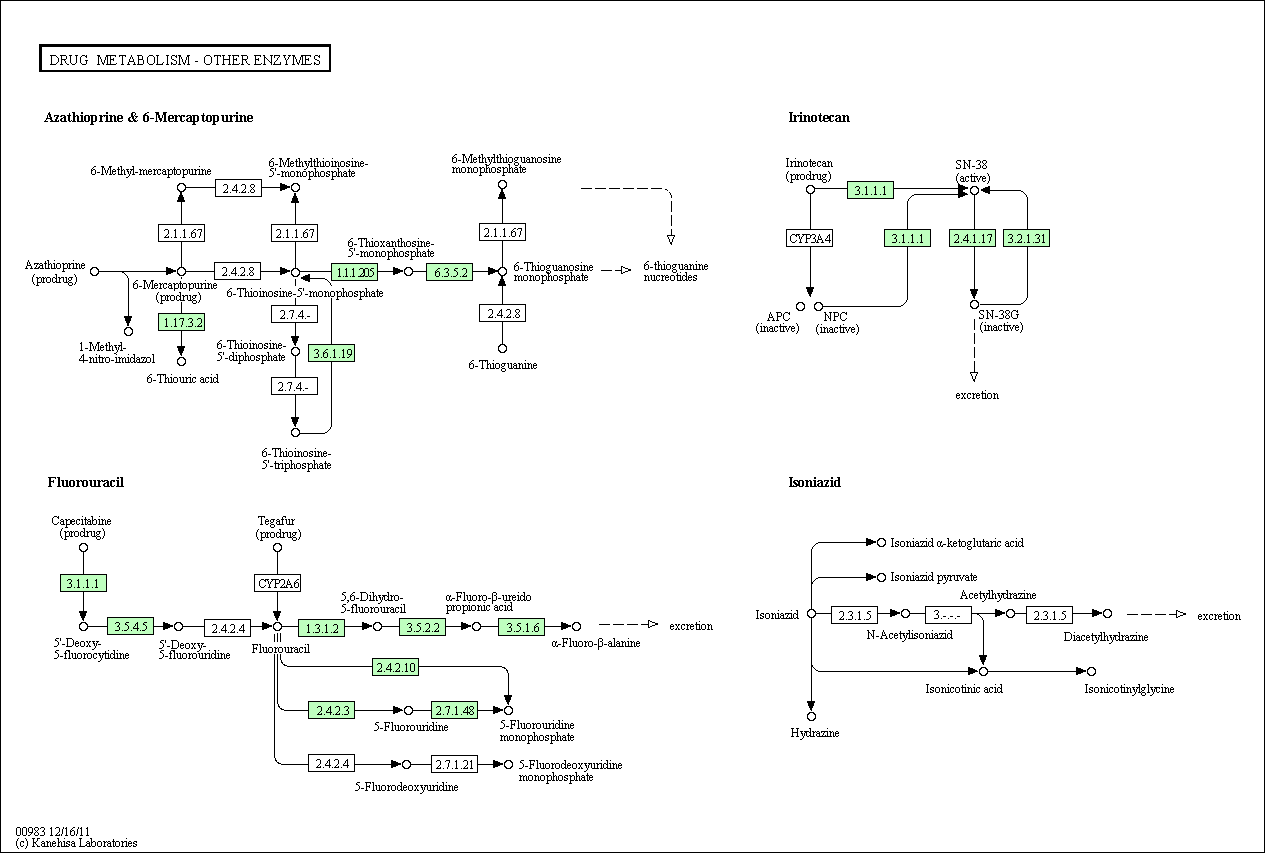

Supplement: Supplementary file 3 — Additional file 3: KEGG classification and functional maps of assembled contigs. Contigs annotated using KEGG Automatic Annotation Server identified sequences in a broad range of functional groups including developmental pathways and cell signaling. (ZIP 11 MB) [file 12864_2013_7026_MOESM3_ESM.zip › KEGG classification/map/map00983.png]

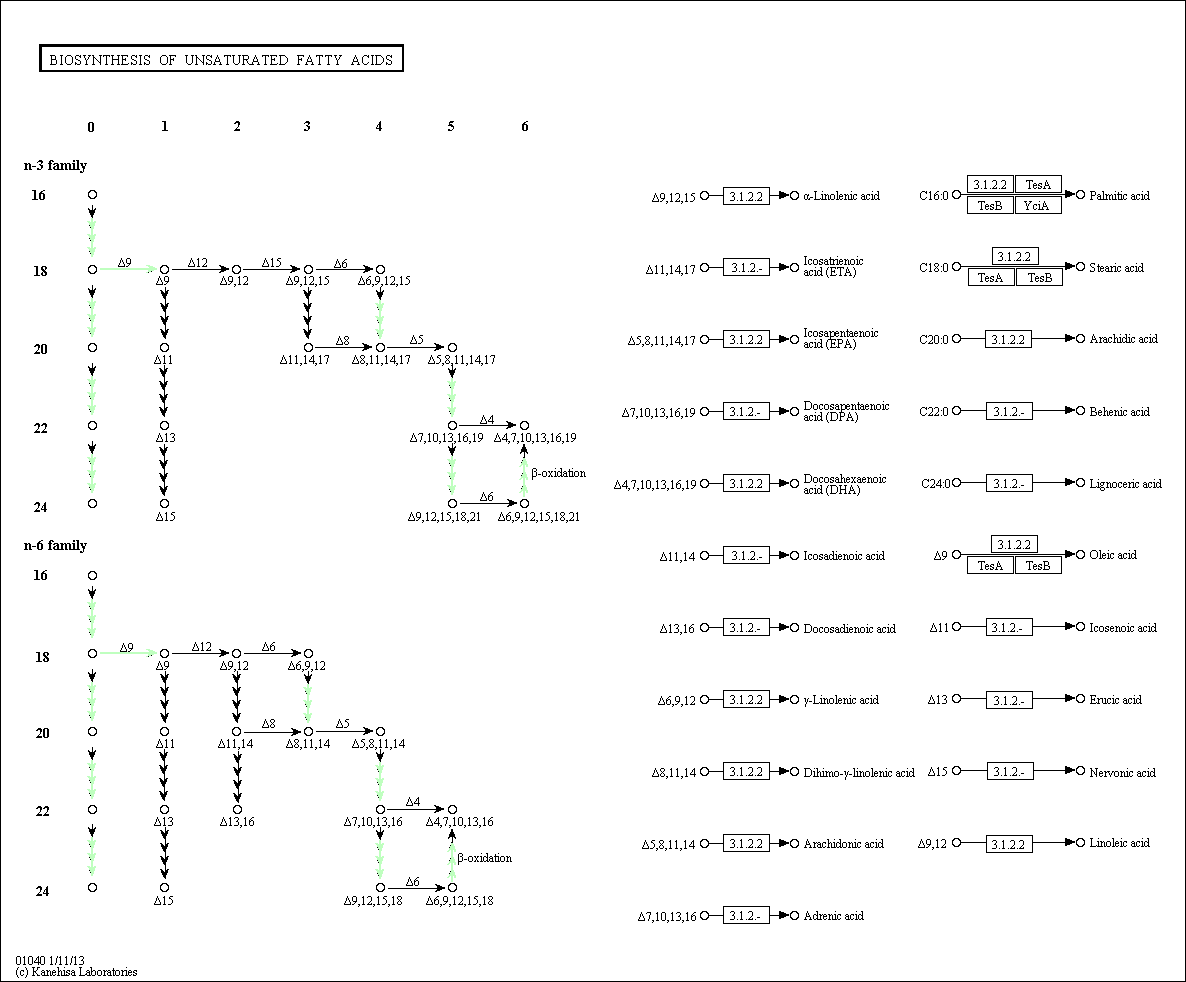

Supplement: Supplementary file 3 — Additional file 3: KEGG classification and functional maps of assembled contigs. Contigs annotated using KEGG Automatic Annotation Server identified sequences in a broad range of functional groups including developmental pathways and cell signaling. (ZIP 11 MB) [file 12864_2013_7026_MOESM3_ESM.zip › KEGG classification/map/map01040.png]

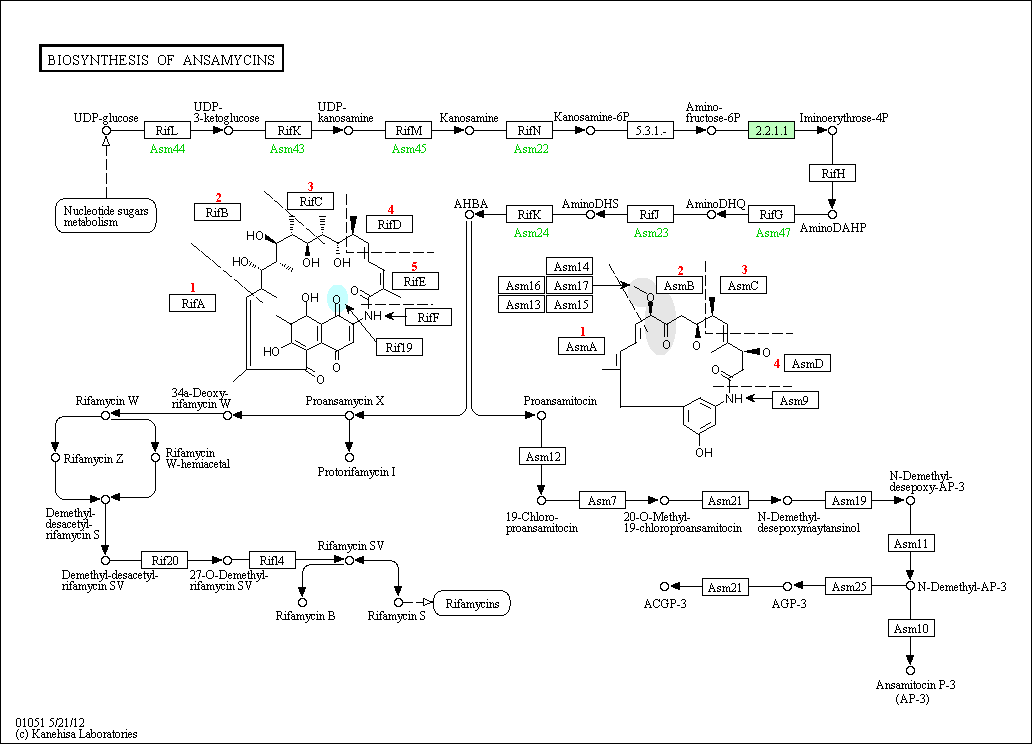

Supplement: Supplementary file 3 — Additional file 3: KEGG classification and functional maps of assembled contigs. Contigs annotated using KEGG Automatic Annotation Server identified sequences in a broad range of functional groups including developmental pathways and cell signaling. (ZIP 11 MB) [file 12864_2013_7026_MOESM3_ESM.zip › KEGG classification/map/map01051.png]

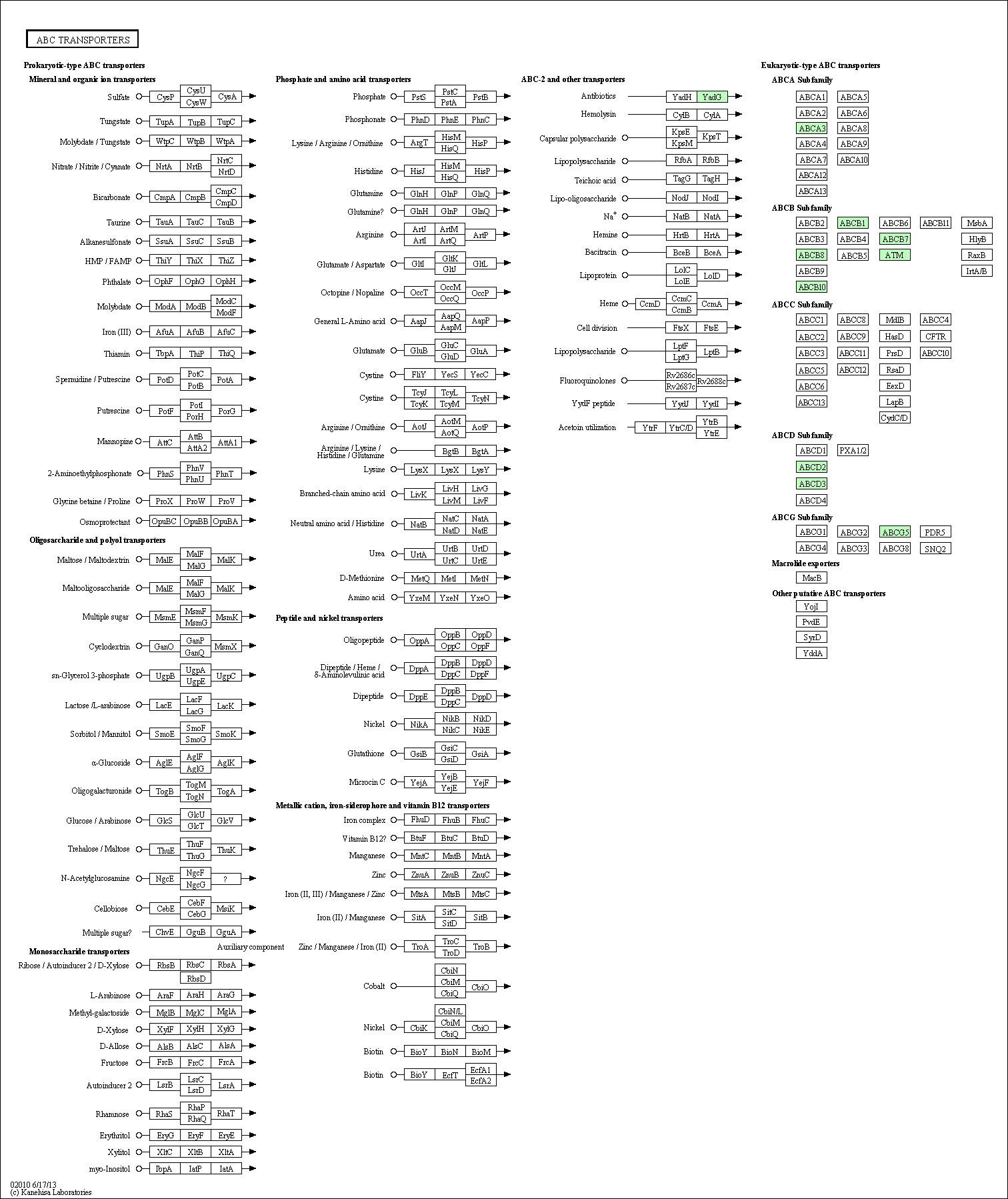

Supplement: Supplementary file 3 — Additional file 3: KEGG classification and functional maps of assembled contigs. Contigs annotated using KEGG Automatic Annotation Server identified sequences in a broad range of functional groups including developmental pathways and cell signaling. (ZIP 11 MB) [file 12864_2013_7026_MOESM3_ESM.zip › KEGG classification/map/map02010.png]

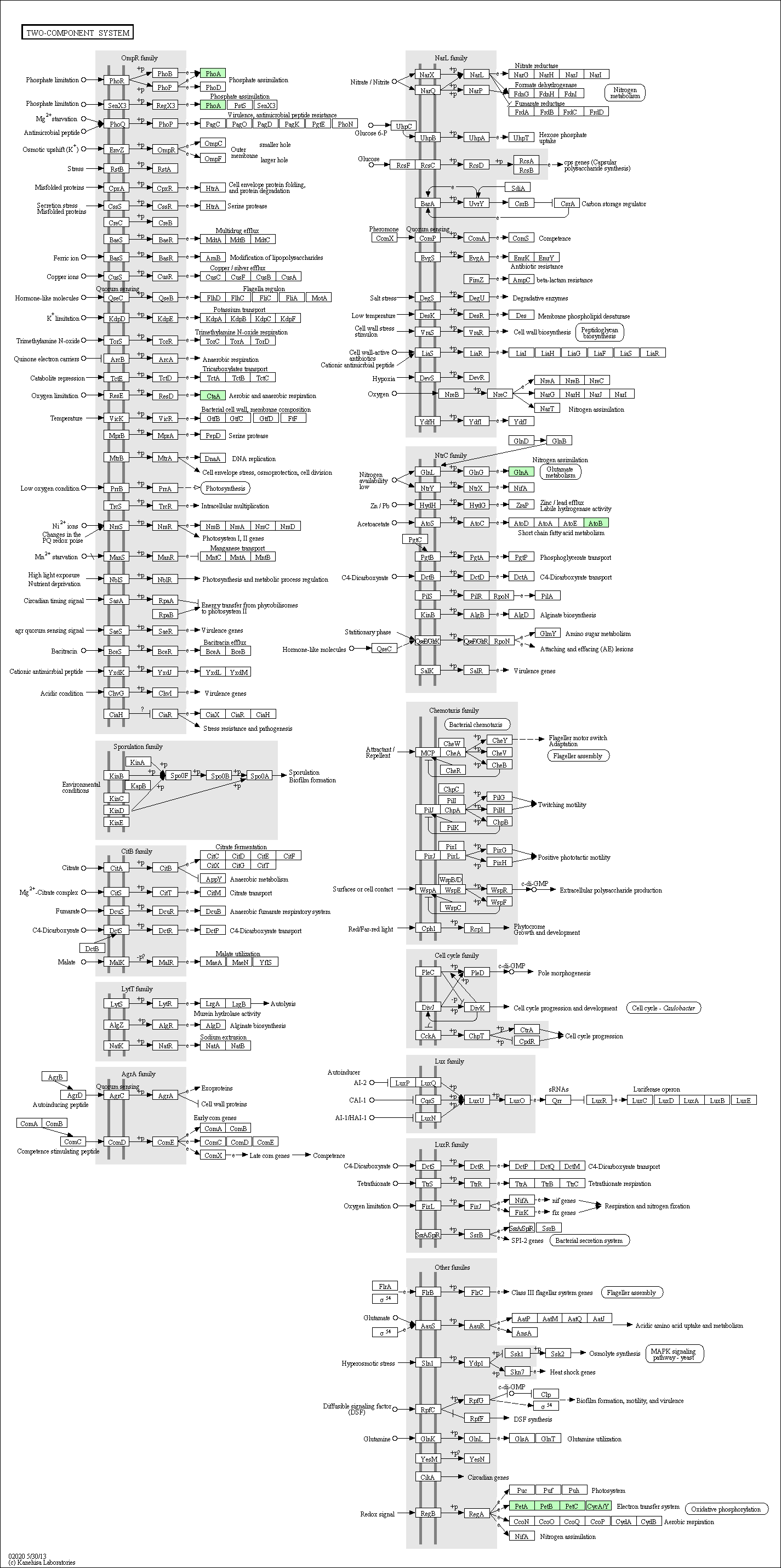

Supplement: Supplementary file 3 — Additional file 3: KEGG classification and functional maps of assembled contigs. Contigs annotated using KEGG Automatic Annotation Server identified sequences in a broad range of functional groups including developmental pathways and cell signaling. (ZIP 11 MB) [file 12864_2013_7026_MOESM3_ESM.zip › KEGG classification/map/map02020.png]

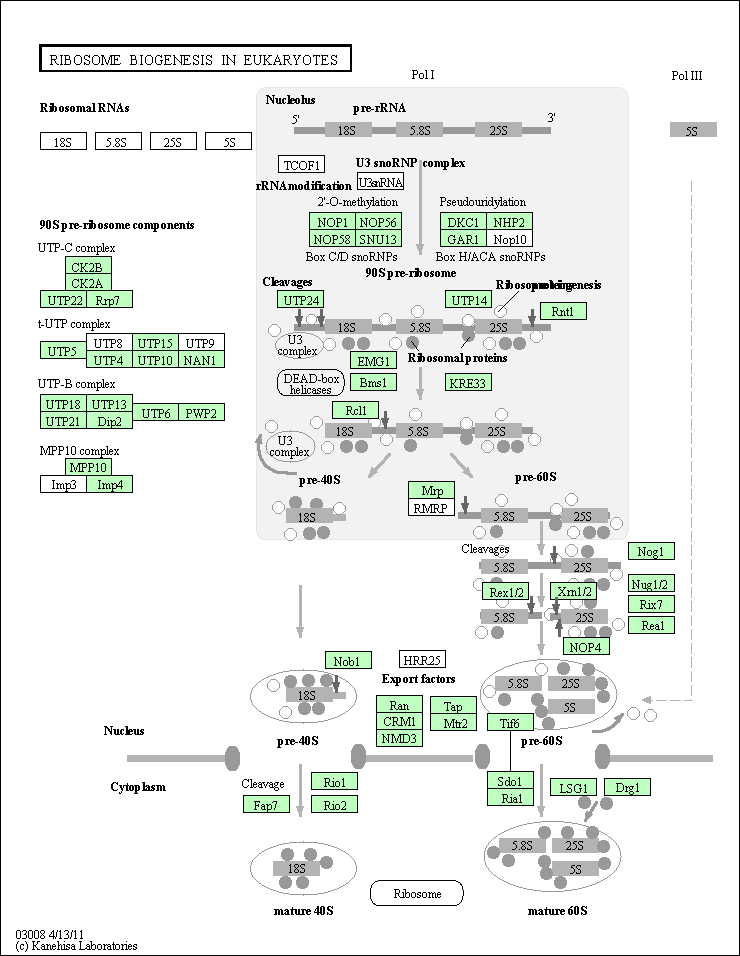

Supplement: Supplementary file 3 — Additional file 3: KEGG classification and functional maps of assembled contigs. Contigs annotated using KEGG Automatic Annotation Server identified sequences in a broad range of functional groups including developmental pathways and cell signaling. (ZIP 11 MB) [file 12864_2013_7026_MOESM3_ESM.zip › KEGG classification/map/map03008.png]

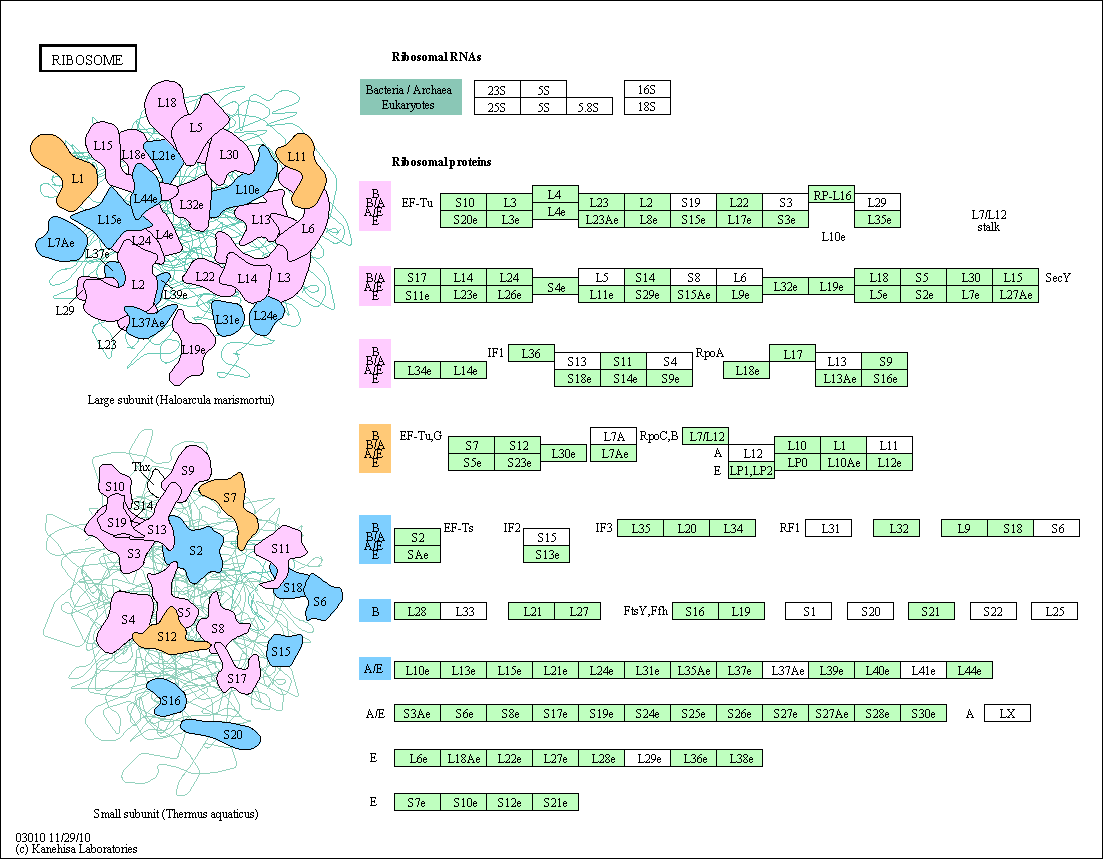

Supplement: Supplementary file 3 — Additional file 3: KEGG classification and functional maps of assembled contigs. Contigs annotated using KEGG Automatic Annotation Server identified sequences in a broad range of functional groups including developmental pathways and cell signaling. (ZIP 11 MB) [file 12864_2013_7026_MOESM3_ESM.zip › KEGG classification/map/map03010.png]

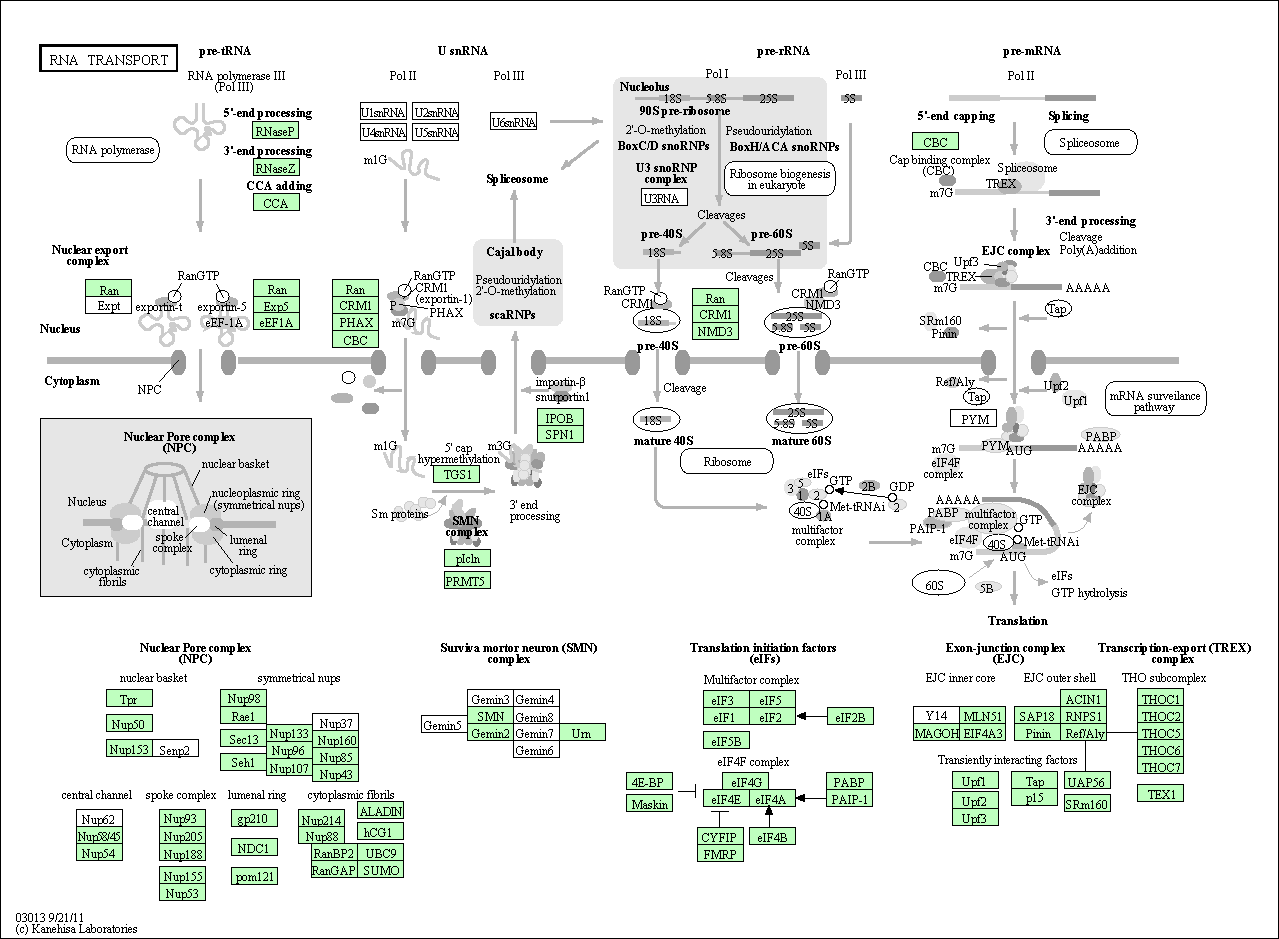

Supplement: Supplementary file 3 — Additional file 3: KEGG classification and functional maps of assembled contigs. Contigs annotated using KEGG Automatic Annotation Server identified sequences in a broad range of functional groups including developmental pathways and cell signaling. (ZIP 11 MB) [file 12864_2013_7026_MOESM3_ESM.zip › KEGG classification/map/map03013.png]

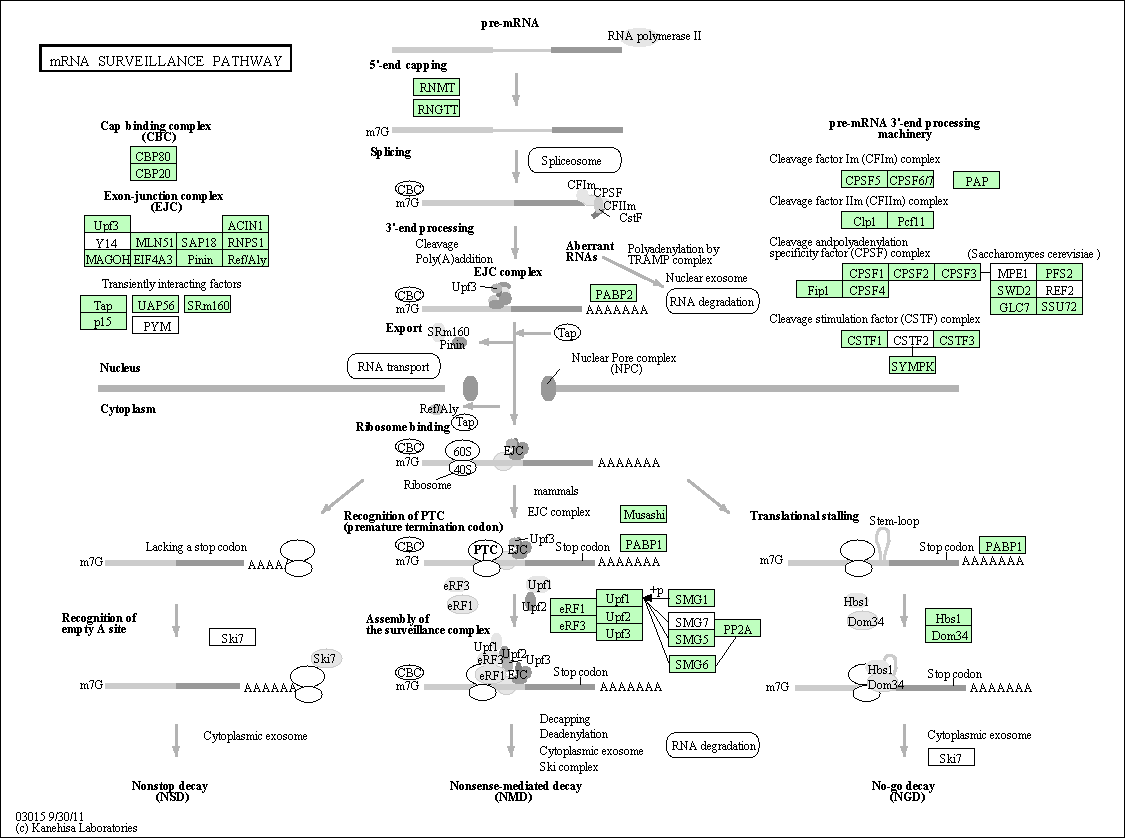

Supplement: Supplementary file 3 — Additional file 3: KEGG classification and functional maps of assembled contigs. Contigs annotated using KEGG Automatic Annotation Server identified sequences in a broad range of functional groups including developmental pathways and cell signaling. (ZIP 11 MB) [file 12864_2013_7026_MOESM3_ESM.zip › KEGG classification/map/map03015.png]

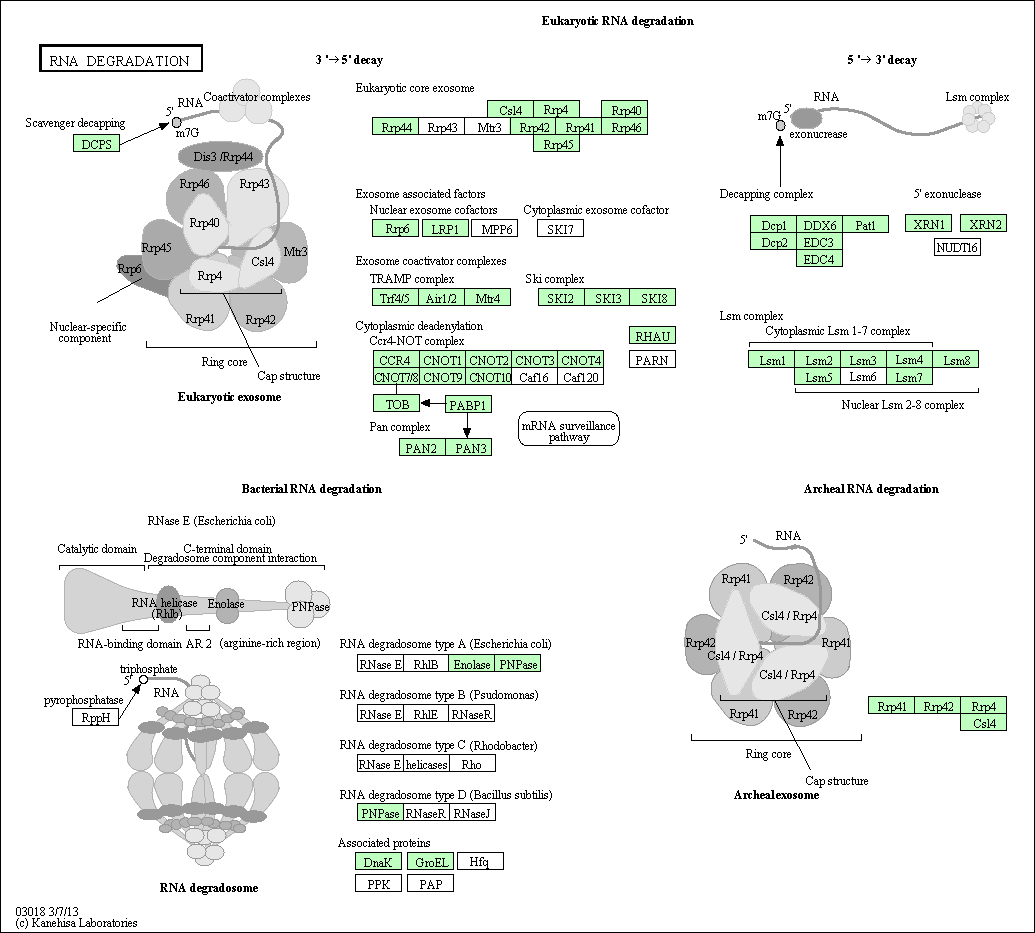

Supplement: Supplementary file 3 — Additional file 3: KEGG classification and functional maps of assembled contigs. Contigs annotated using KEGG Automatic Annotation Server identified sequences in a broad range of functional groups including developmental pathways and cell signaling. (ZIP 11 MB) [file 12864_2013_7026_MOESM3_ESM.zip › KEGG classification/map/map03018.png]

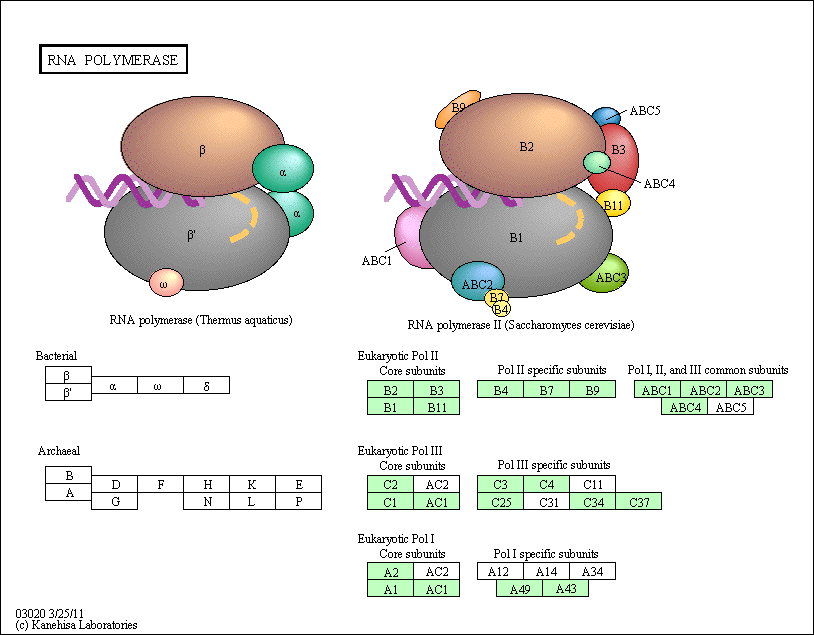

Supplement: Supplementary file 3 — Additional file 3: KEGG classification and functional maps of assembled contigs. Contigs annotated using KEGG Automatic Annotation Server identified sequences in a broad range of functional groups including developmental pathways and cell signaling. (ZIP 11 MB) [file 12864_2013_7026_MOESM3_ESM.zip › KEGG classification/map/map03020.png]

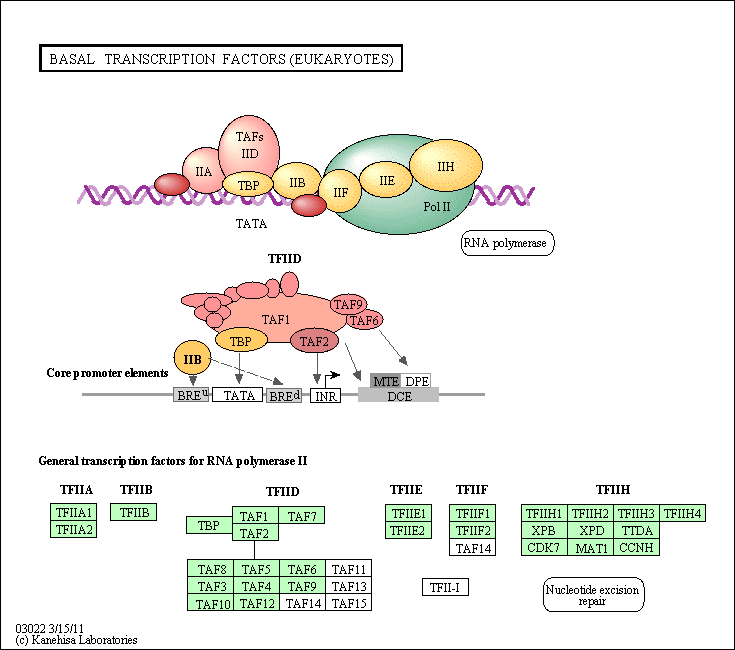

Supplement: Supplementary file 3 — Additional file 3: KEGG classification and functional maps of assembled contigs. Contigs annotated using KEGG Automatic Annotation Server identified sequences in a broad range of functional groups including developmental pathways and cell signaling. (ZIP 11 MB) [file 12864_2013_7026_MOESM3_ESM.zip › KEGG classification/map/map03022.png]

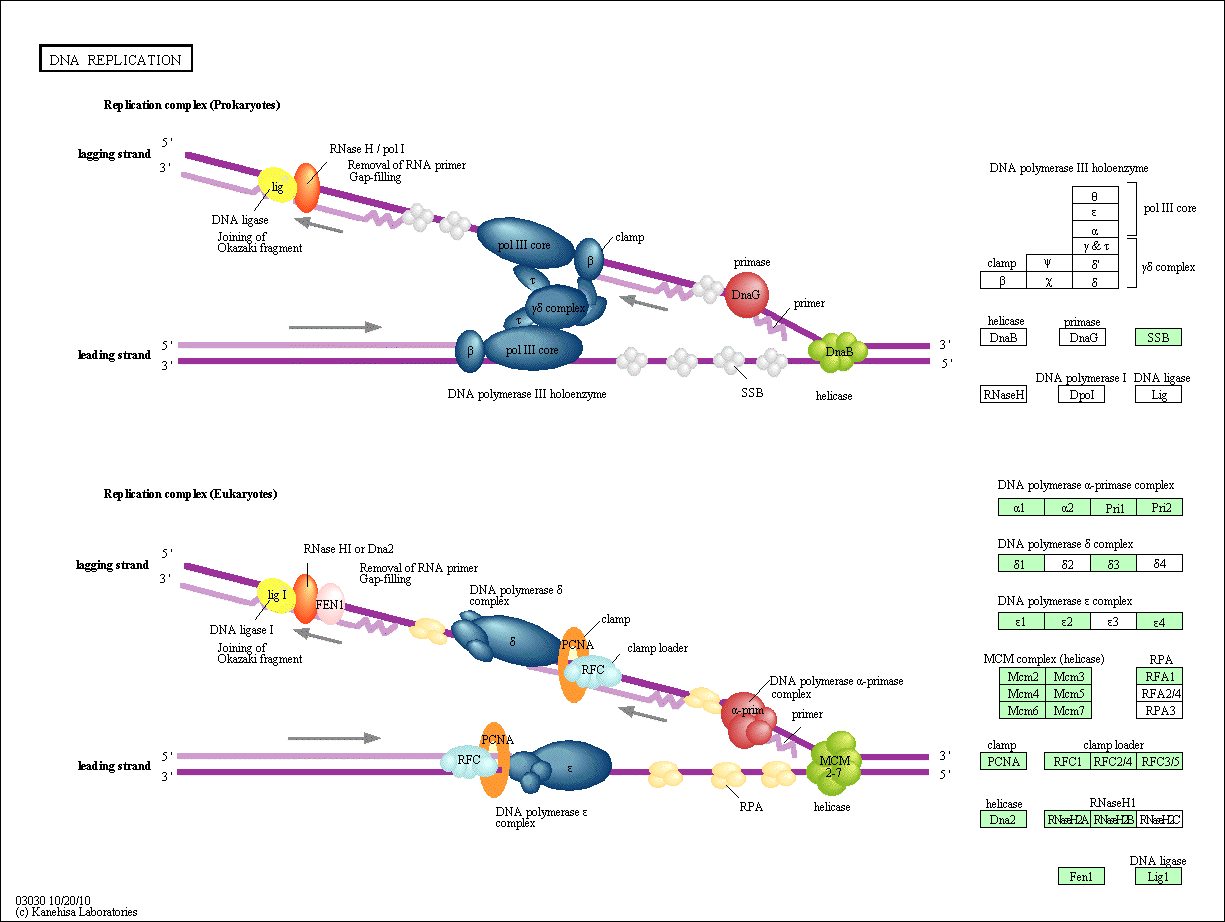

Supplement: Supplementary file 3 — Additional file 3: KEGG classification and functional maps of assembled contigs. Contigs annotated using KEGG Automatic Annotation Server identified sequences in a broad range of functional groups including developmental pathways and cell signaling. (ZIP 11 MB) [file 12864_2013_7026_MOESM3_ESM.zip › KEGG classification/map/map03030.png]

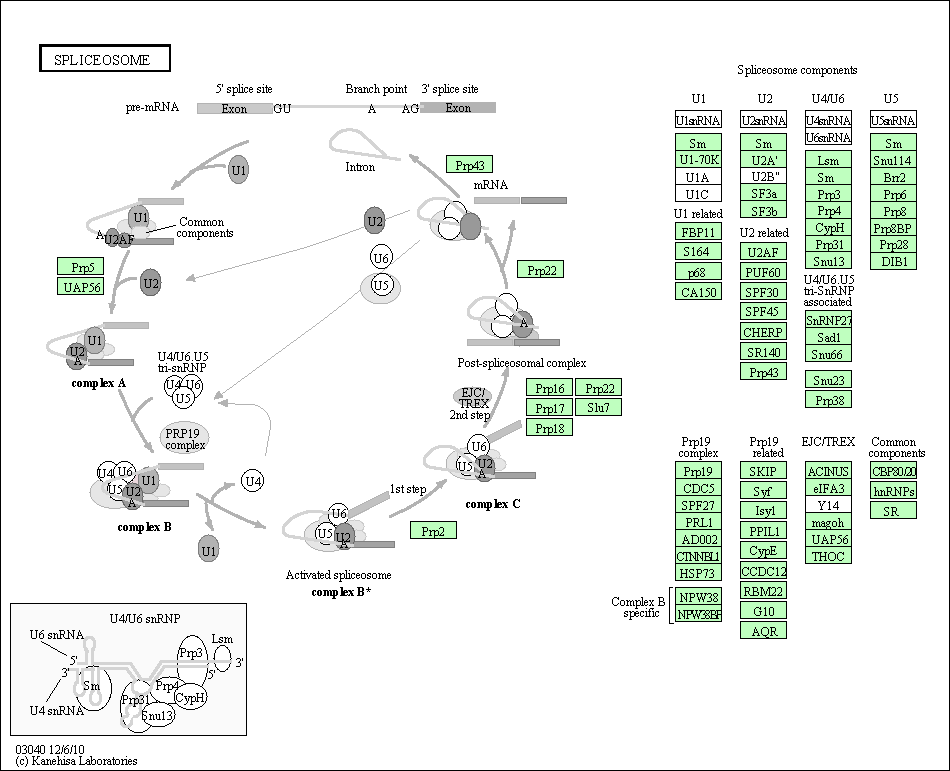

Supplement: Supplementary file 3 — Additional file 3: KEGG classification and functional maps of assembled contigs. Contigs annotated using KEGG Automatic Annotation Server identified sequences in a broad range of functional groups including developmental pathways and cell signaling. (ZIP 11 MB) [file 12864_2013_7026_MOESM3_ESM.zip › KEGG classification/map/map03040.png]

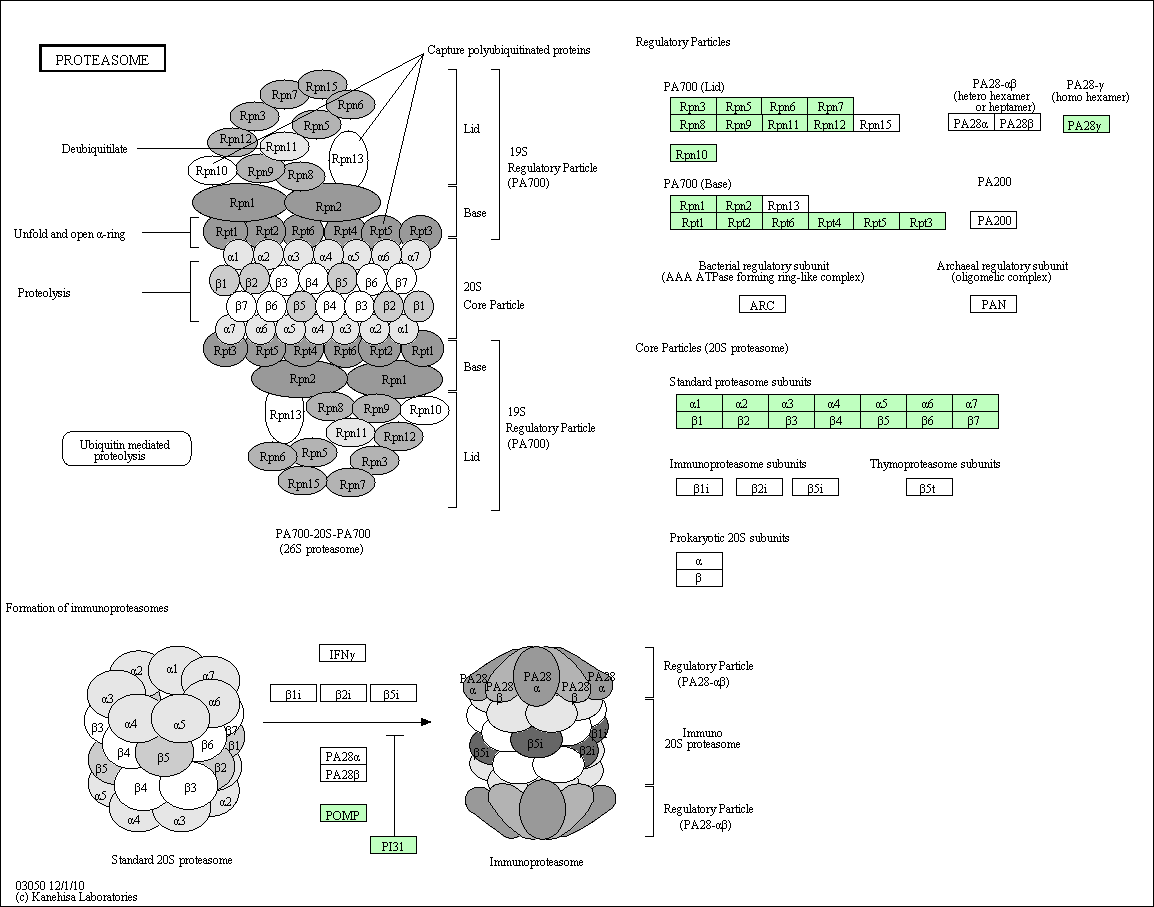

Supplement: Supplementary file 3 — Additional file 3: KEGG classification and functional maps of assembled contigs. Contigs annotated using KEGG Automatic Annotation Server identified sequences in a broad range of functional groups including developmental pathways and cell signaling. (ZIP 11 MB) [file 12864_2013_7026_MOESM3_ESM.zip › KEGG classification/map/map03050.png]

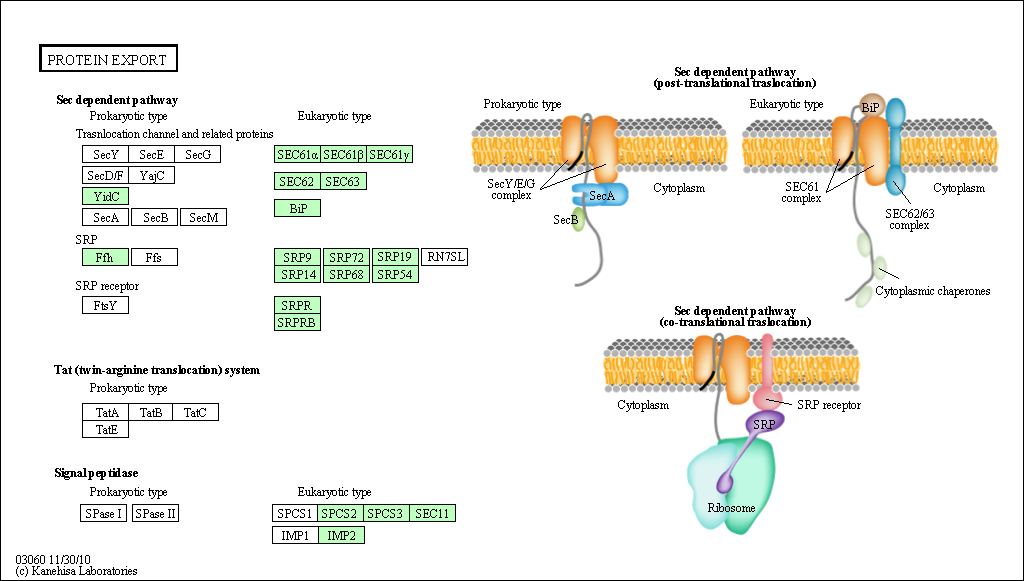

Supplement: Supplementary file 3 — Additional file 3: KEGG classification and functional maps of assembled contigs. Contigs annotated using KEGG Automatic Annotation Server identified sequences in a broad range of functional groups including developmental pathways and cell signaling. (ZIP 11 MB) [file 12864_2013_7026_MOESM3_ESM.zip › KEGG classification/map/map03060.png]

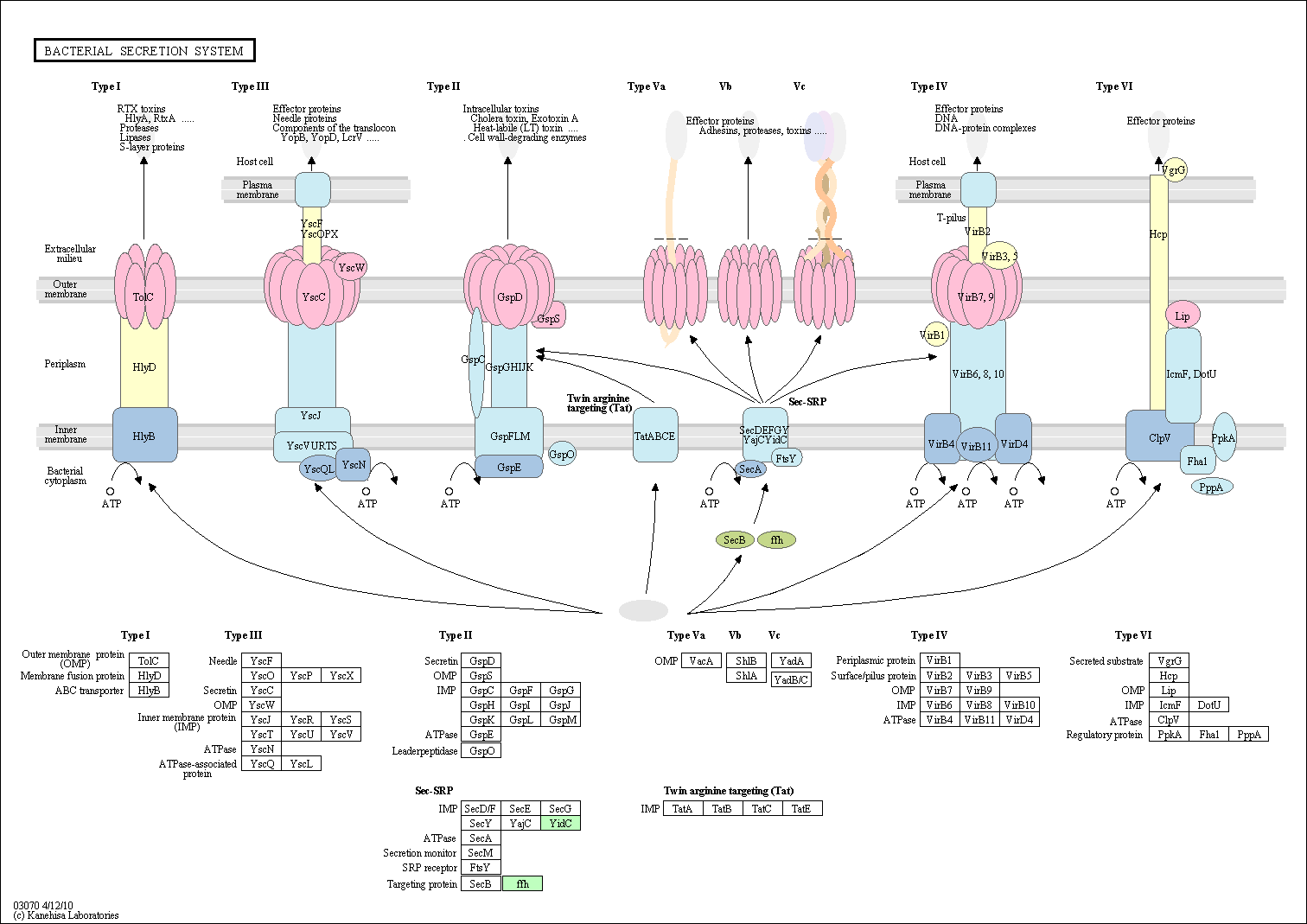

Supplement: Supplementary file 3 — Additional file 3: KEGG classification and functional maps of assembled contigs. Contigs annotated using KEGG Automatic Annotation Server identified sequences in a broad range of functional groups including developmental pathways and cell signaling. (ZIP 11 MB) [file 12864_2013_7026_MOESM3_ESM.zip › KEGG classification/map/map03070.png]

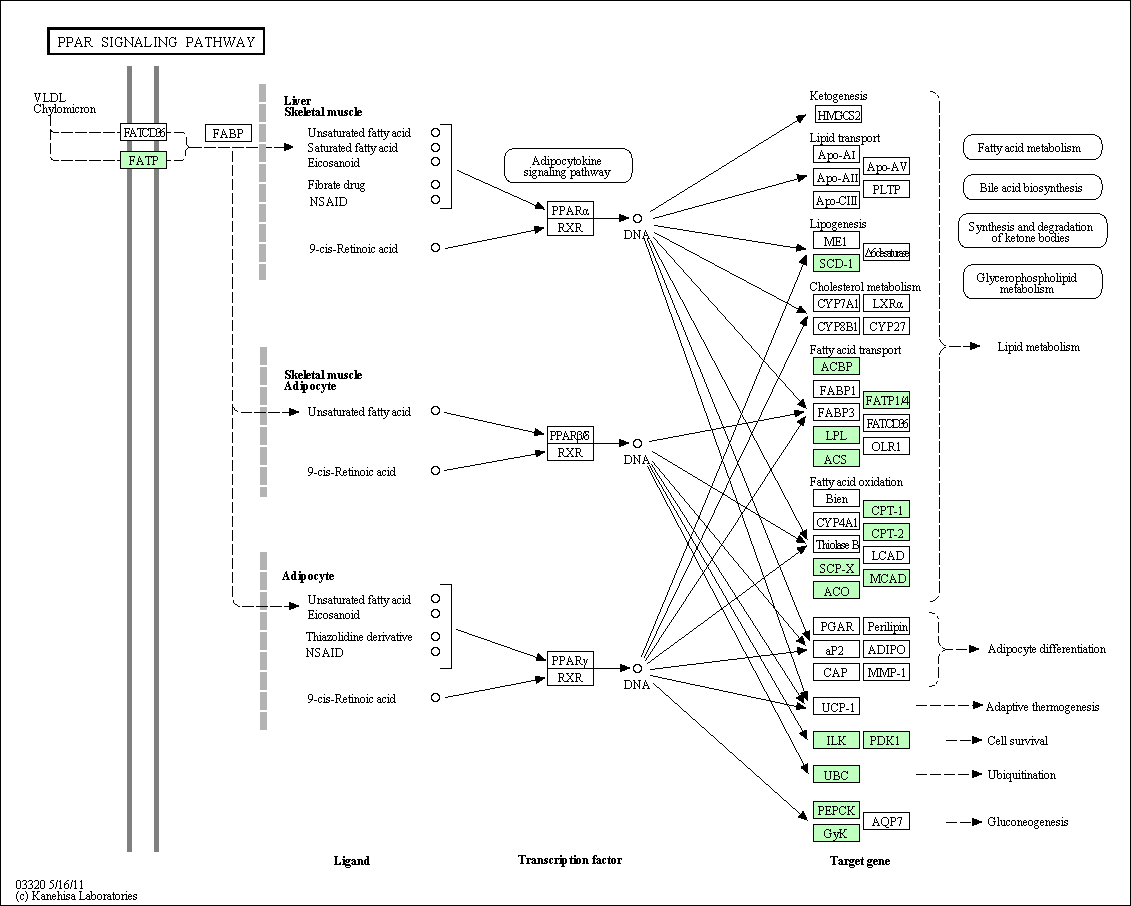

Supplement: Supplementary file 3 — Additional file 3: KEGG classification and functional maps of assembled contigs. Contigs annotated using KEGG Automatic Annotation Server identified sequences in a broad range of functional groups including developmental pathways and cell signaling. (ZIP 11 MB) [file 12864_2013_7026_MOESM3_ESM.zip › KEGG classification/map/map03320.png]

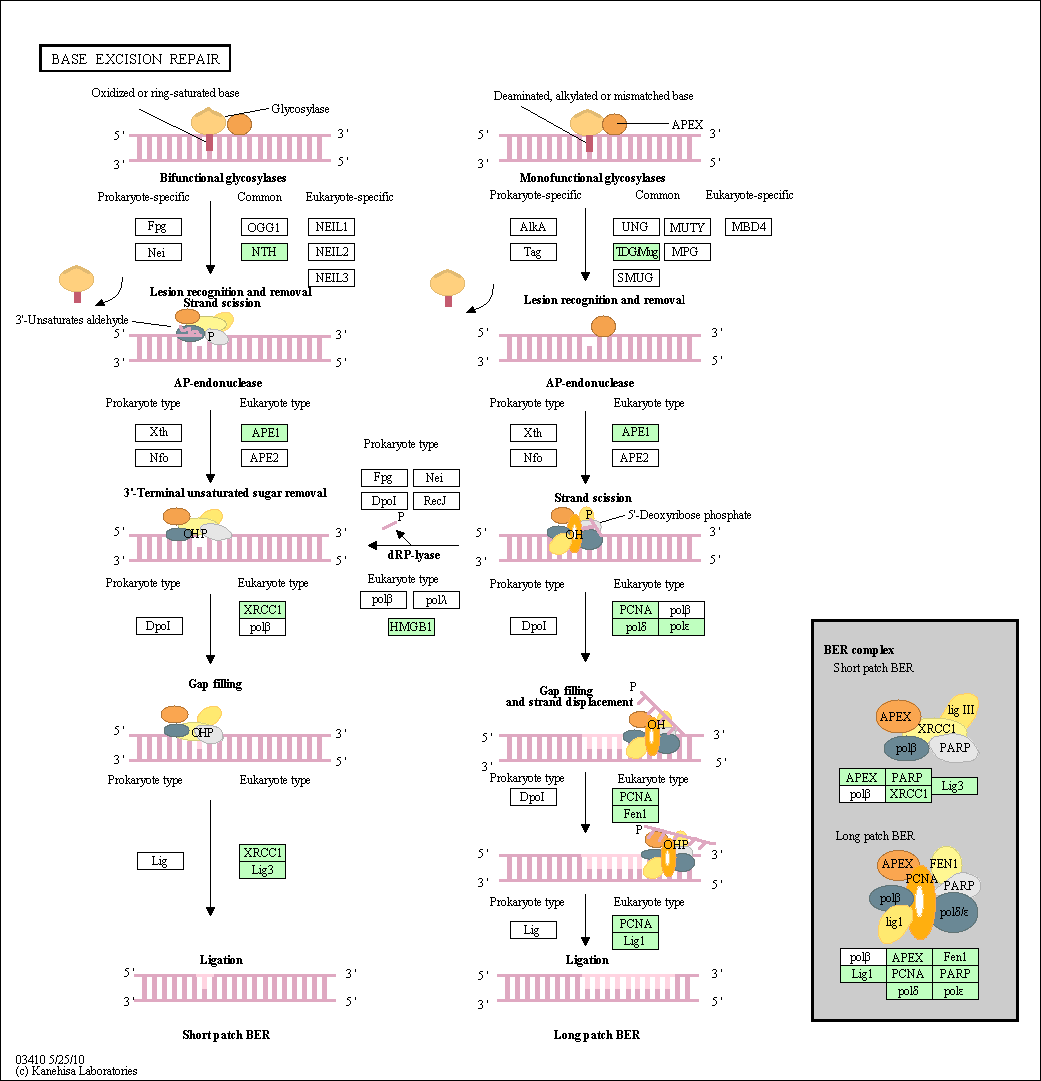

Supplement: Supplementary file 3 — Additional file 3: KEGG classification and functional maps of assembled contigs. Contigs annotated using KEGG Automatic Annotation Server identified sequences in a broad range of functional groups including developmental pathways and cell signaling. (ZIP 11 MB) [file 12864_2013_7026_MOESM3_ESM.zip › KEGG classification/map/map03410.png]

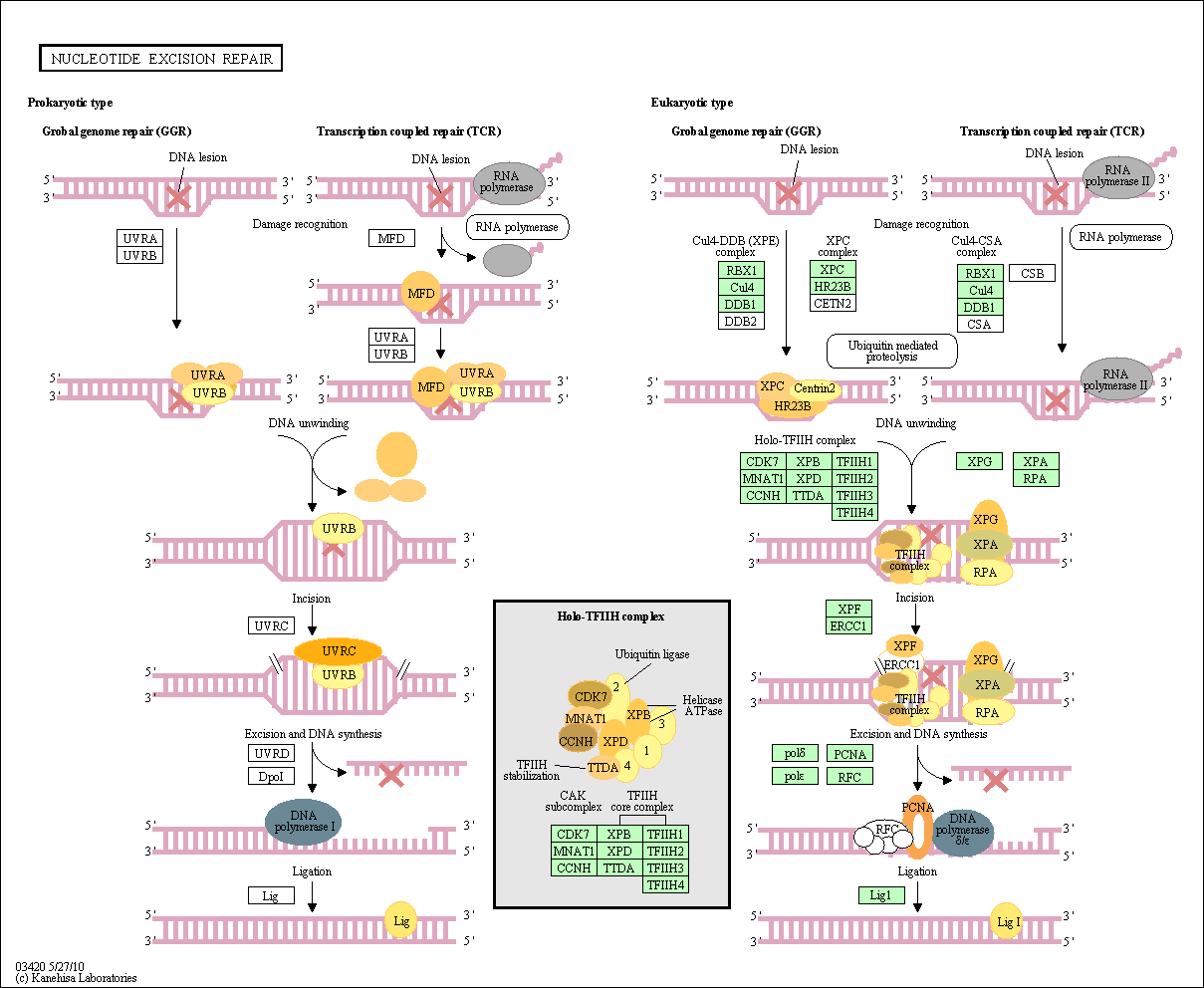

Supplement: Supplementary file 3 — Additional file 3: KEGG classification and functional maps of assembled contigs. Contigs annotated using KEGG Automatic Annotation Server identified sequences in a broad range of functional groups including developmental pathways and cell signaling. (ZIP 11 MB) [file 12864_2013_7026_MOESM3_ESM.zip › KEGG classification/map/map03420.png]

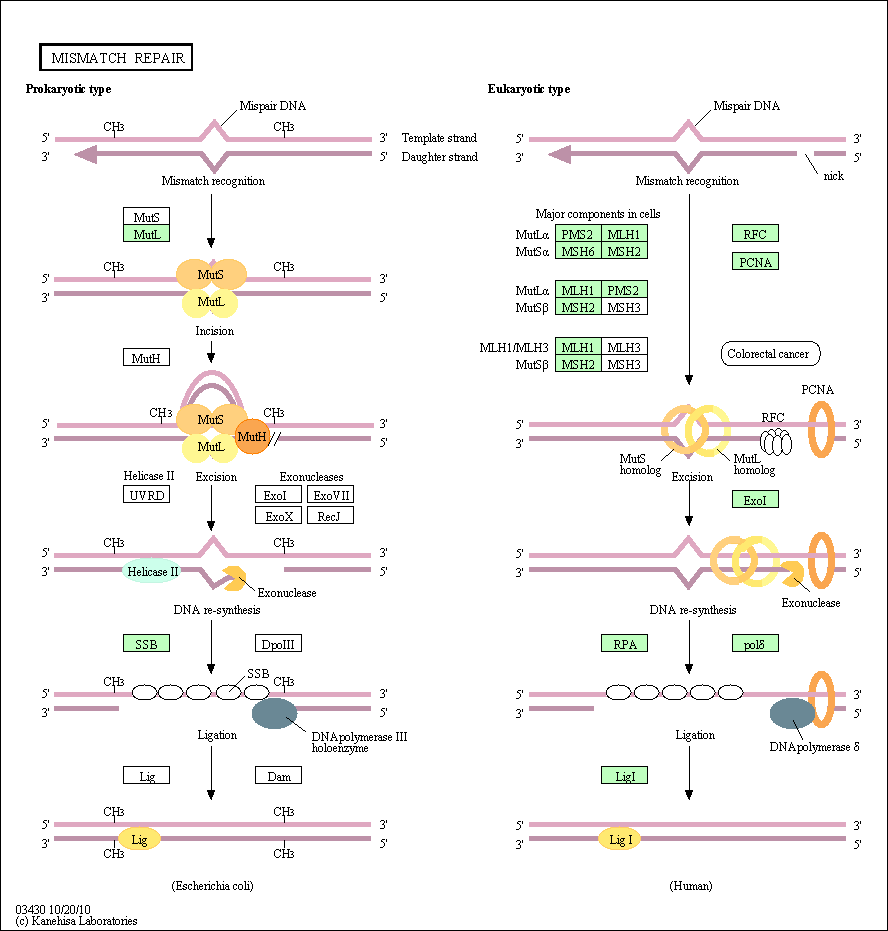

Supplement: Supplementary file 3 — Additional file 3: KEGG classification and functional maps of assembled contigs. Contigs annotated using KEGG Automatic Annotation Server identified sequences in a broad range of functional groups including developmental pathways and cell signaling. (ZIP 11 MB) [file 12864_2013_7026_MOESM3_ESM.zip › KEGG classification/map/map03430.png]

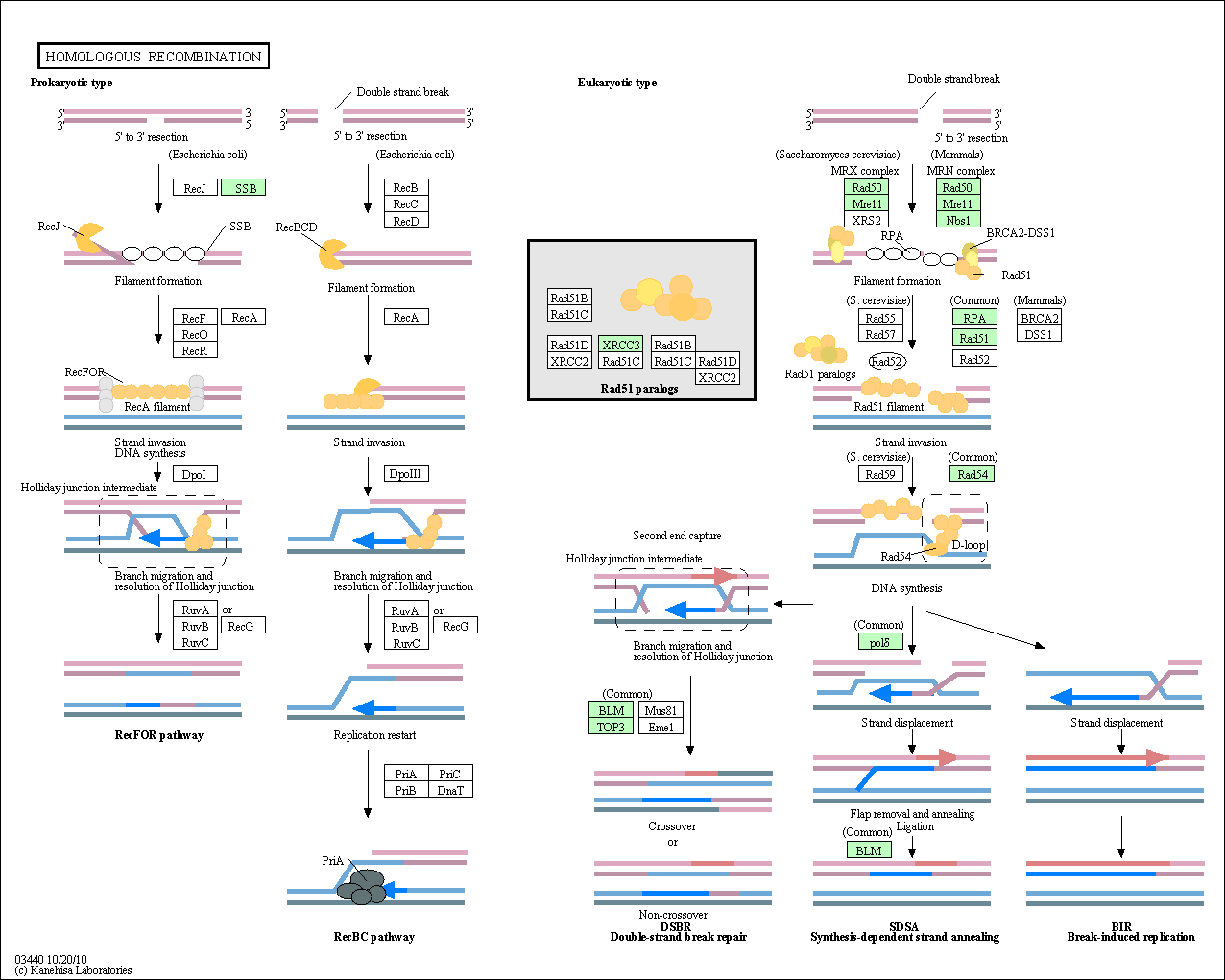

Supplement: Supplementary file 3 — Additional file 3: KEGG classification and functional maps of assembled contigs. Contigs annotated using KEGG Automatic Annotation Server identified sequences in a broad range of functional groups including developmental pathways and cell signaling. (ZIP 11 MB) [file 12864_2013_7026_MOESM3_ESM.zip › KEGG classification/map/map03440.png]

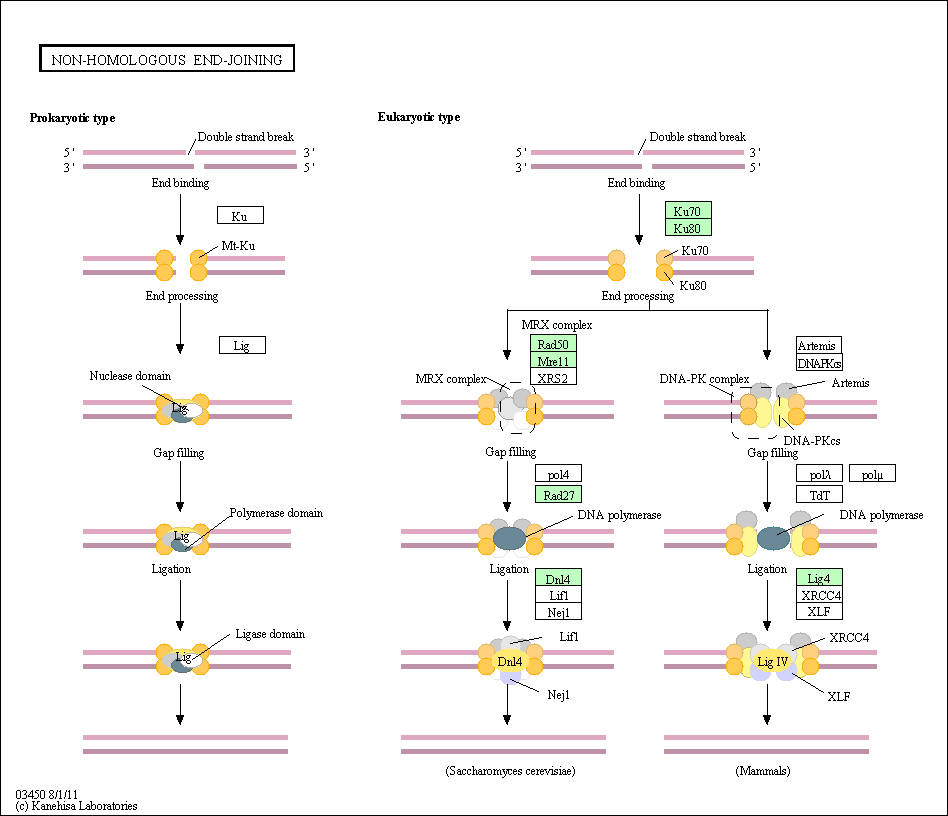

Supplement: Supplementary file 3 — Additional file 3: KEGG classification and functional maps of assembled contigs. Contigs annotated using KEGG Automatic Annotation Server identified sequences in a broad range of functional groups including developmental pathways and cell signaling. (ZIP 11 MB) [file 12864_2013_7026_MOESM3_ESM.zip › KEGG classification/map/map03450.png]

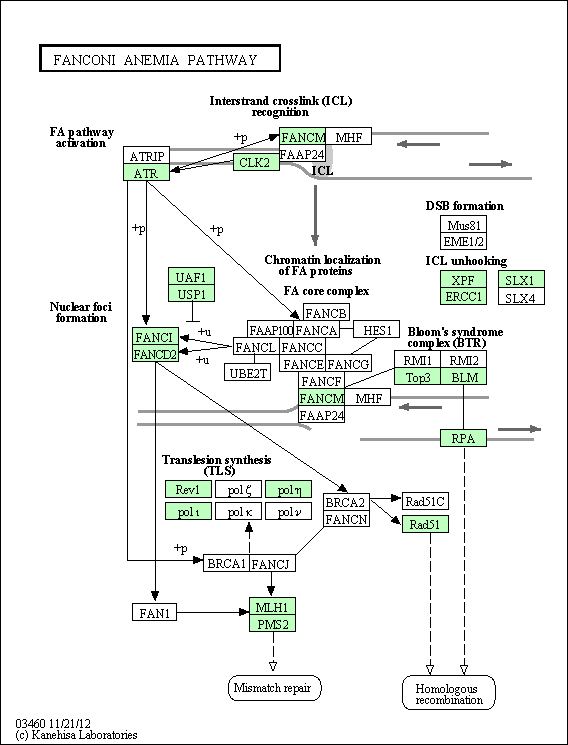

Supplement: Supplementary file 3 — Additional file 3: KEGG classification and functional maps of assembled contigs. Contigs annotated using KEGG Automatic Annotation Server identified sequences in a broad range of functional groups including developmental pathways and cell signaling. (ZIP 11 MB) [file 12864_2013_7026_MOESM3_ESM.zip › KEGG classification/map/map03460.png]

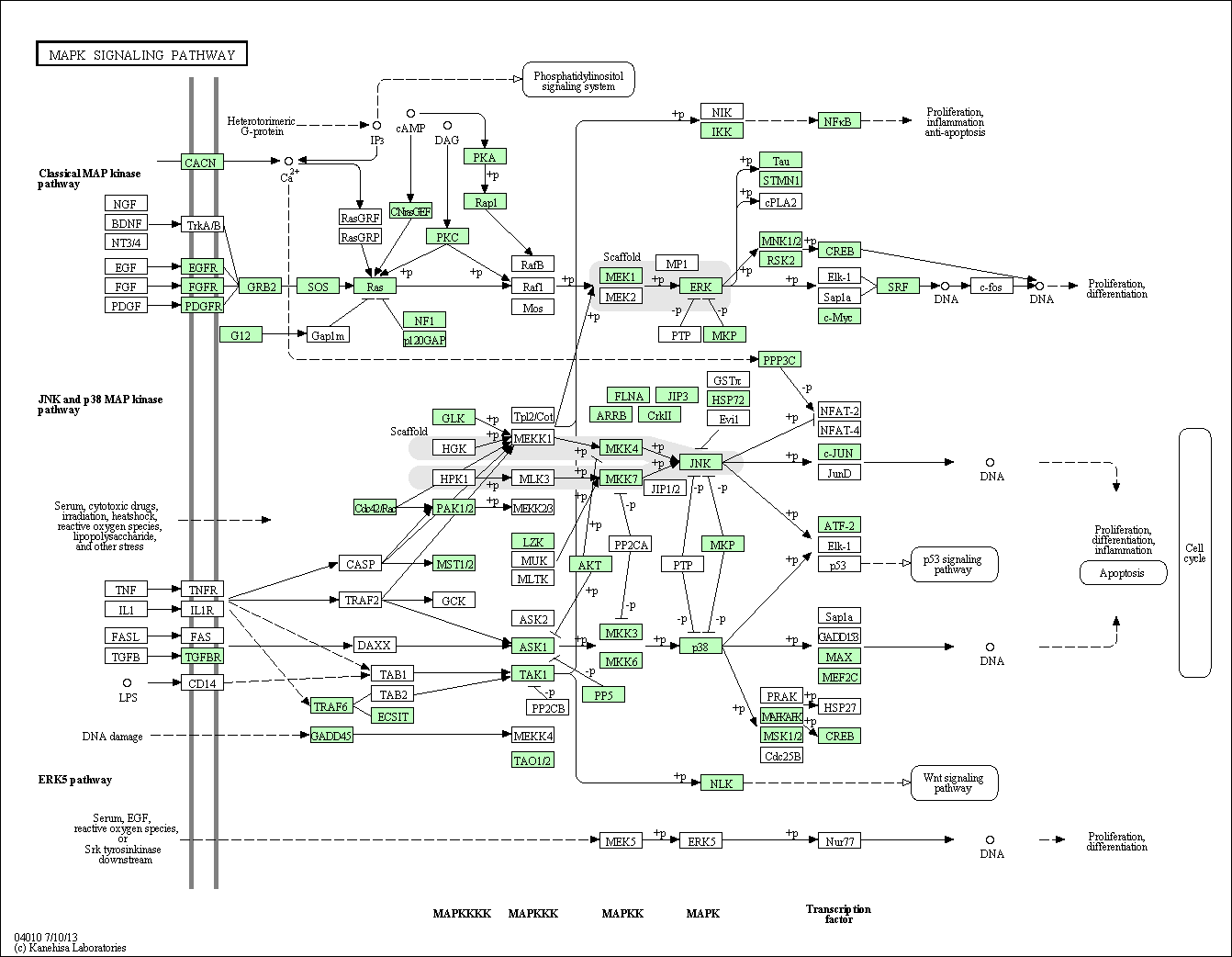

Supplement: Supplementary file 3 — Additional file 3: KEGG classification and functional maps of assembled contigs. Contigs annotated using KEGG Automatic Annotation Server identified sequences in a broad range of functional groups including developmental pathways and cell signaling. (ZIP 11 MB) [file 12864_2013_7026_MOESM3_ESM.zip › KEGG classification/map/map04010.png]

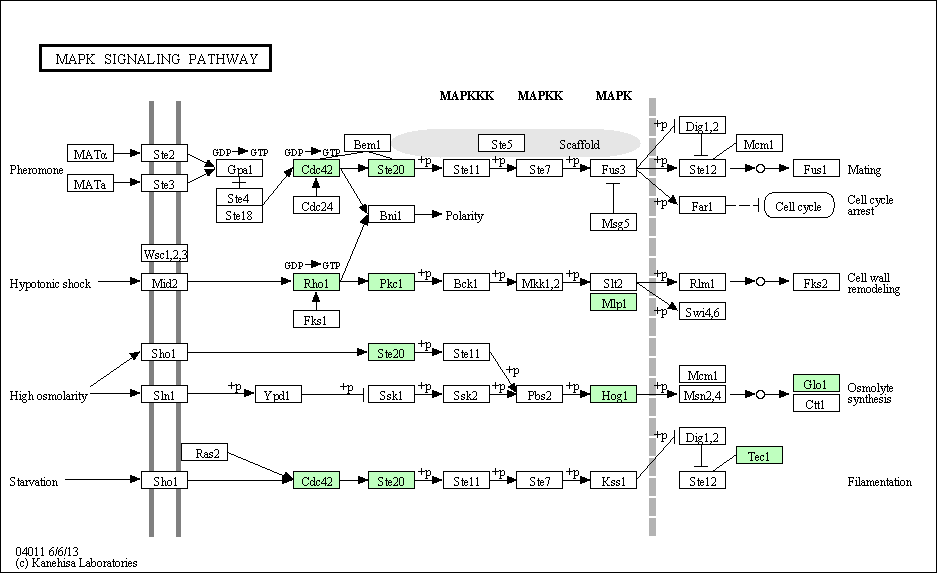

Supplement: Supplementary file 3 — Additional file 3: KEGG classification and functional maps of assembled contigs. Contigs annotated using KEGG Automatic Annotation Server identified sequences in a broad range of functional groups including developmental pathways and cell signaling. (ZIP 11 MB) [file 12864_2013_7026_MOESM3_ESM.zip › KEGG classification/map/map04011.png]

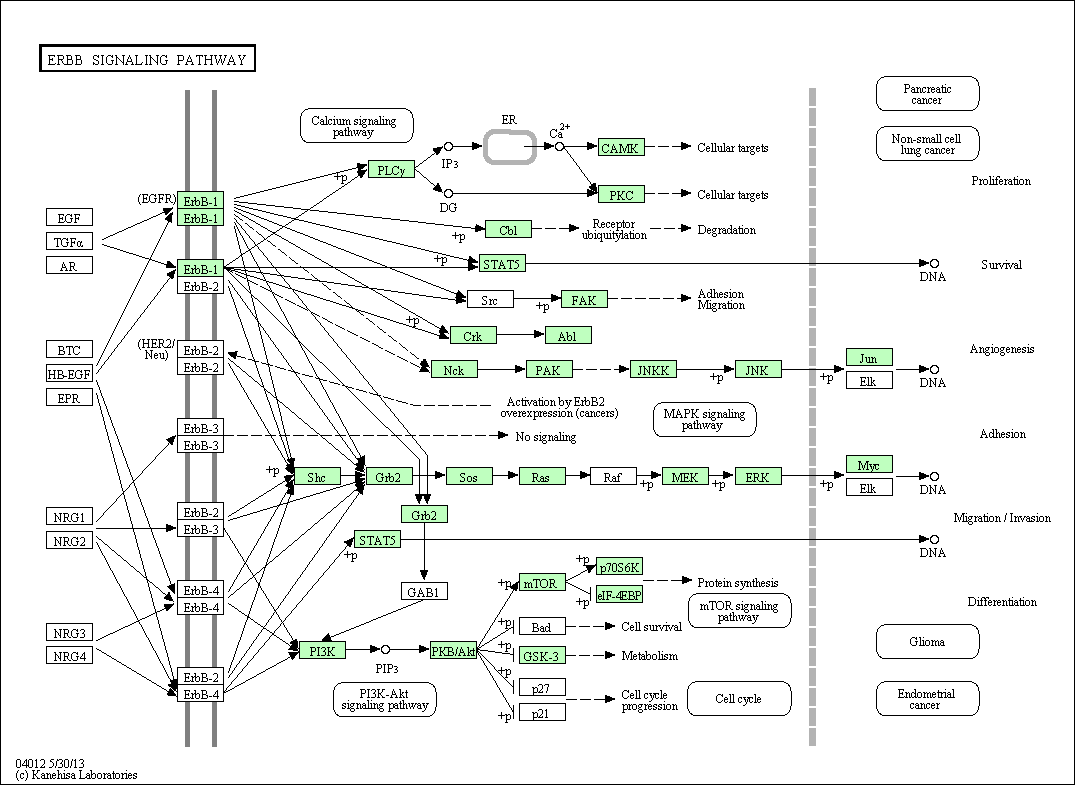

Supplement: Supplementary file 3 — Additional file 3: KEGG classification and functional maps of assembled contigs. Contigs annotated using KEGG Automatic Annotation Server identified sequences in a broad range of functional groups including developmental pathways and cell signaling. (ZIP 11 MB) [file 12864_2013_7026_MOESM3_ESM.zip › KEGG classification/map/map04012.png]

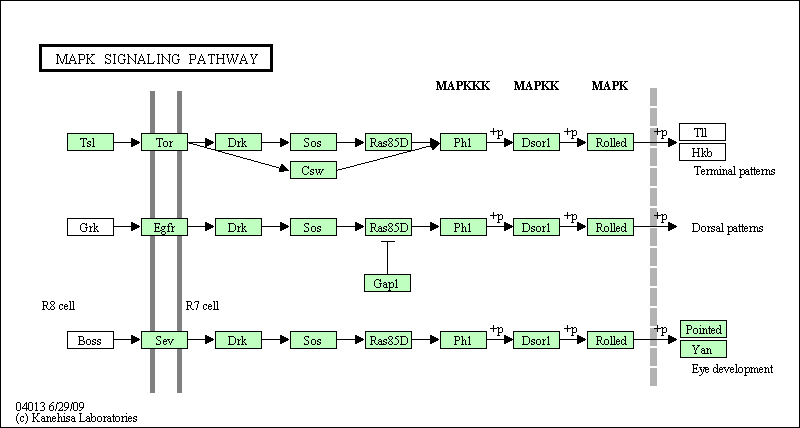

Supplement: Supplementary file 3 — Additional file 3: KEGG classification and functional maps of assembled contigs. Contigs annotated using KEGG Automatic Annotation Server identified sequences in a broad range of functional groups including developmental pathways and cell signaling. (ZIP 11 MB) [file 12864_2013_7026_MOESM3_ESM.zip › KEGG classification/map/map04013.png]

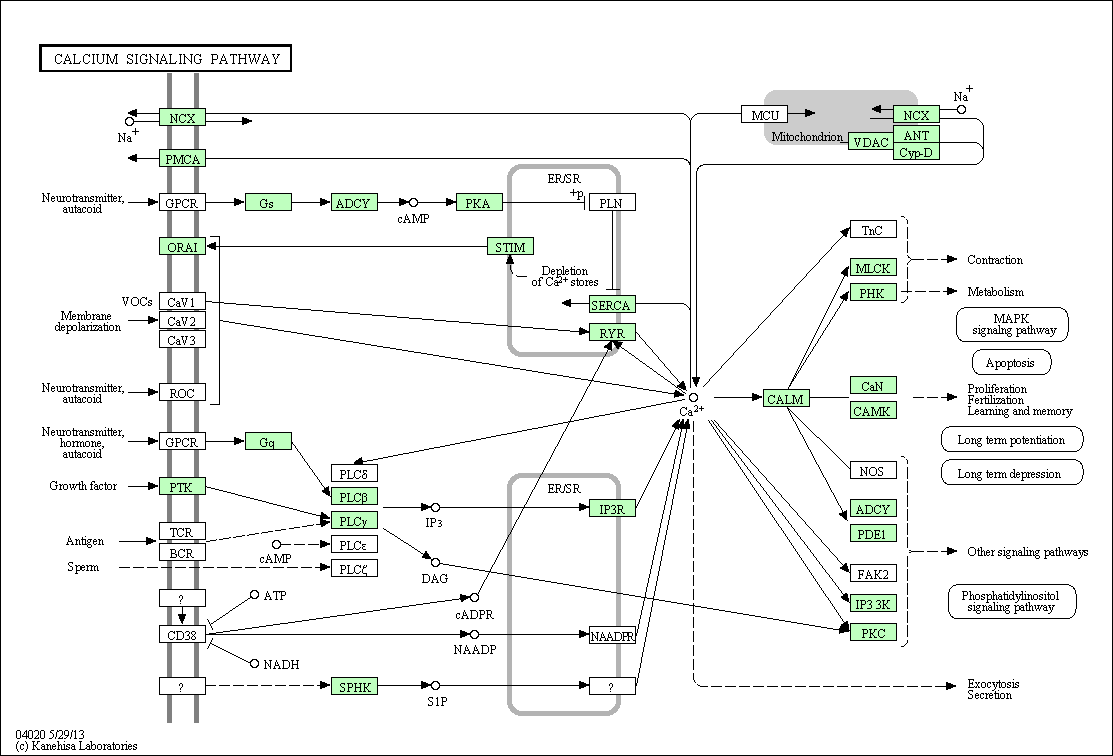

Supplement: Supplementary file 3 — Additional file 3: KEGG classification and functional maps of assembled contigs. Contigs annotated using KEGG Automatic Annotation Server identified sequences in a broad range of functional groups including developmental pathways and cell signaling. (ZIP 11 MB) [file 12864_2013_7026_MOESM3_ESM.zip › KEGG classification/map/map04020.png]

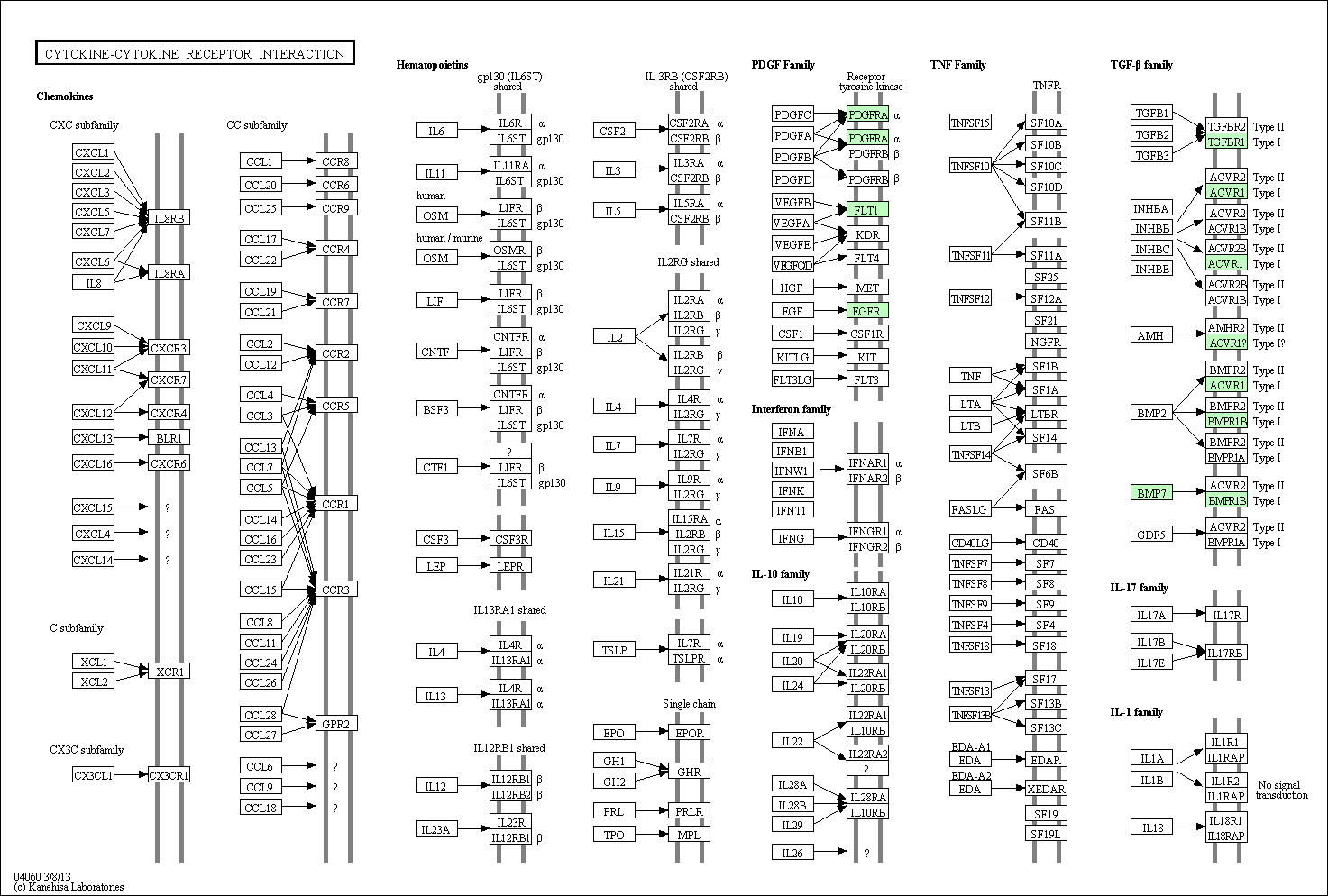

Supplement: Supplementary file 3 — Additional file 3: KEGG classification and functional maps of assembled contigs. Contigs annotated using KEGG Automatic Annotation Server identified sequences in a broad range of functional groups including developmental pathways and cell signaling. (ZIP 11 MB) [file 12864_2013_7026_MOESM3_ESM.zip › KEGG classification/map/map04060.png]

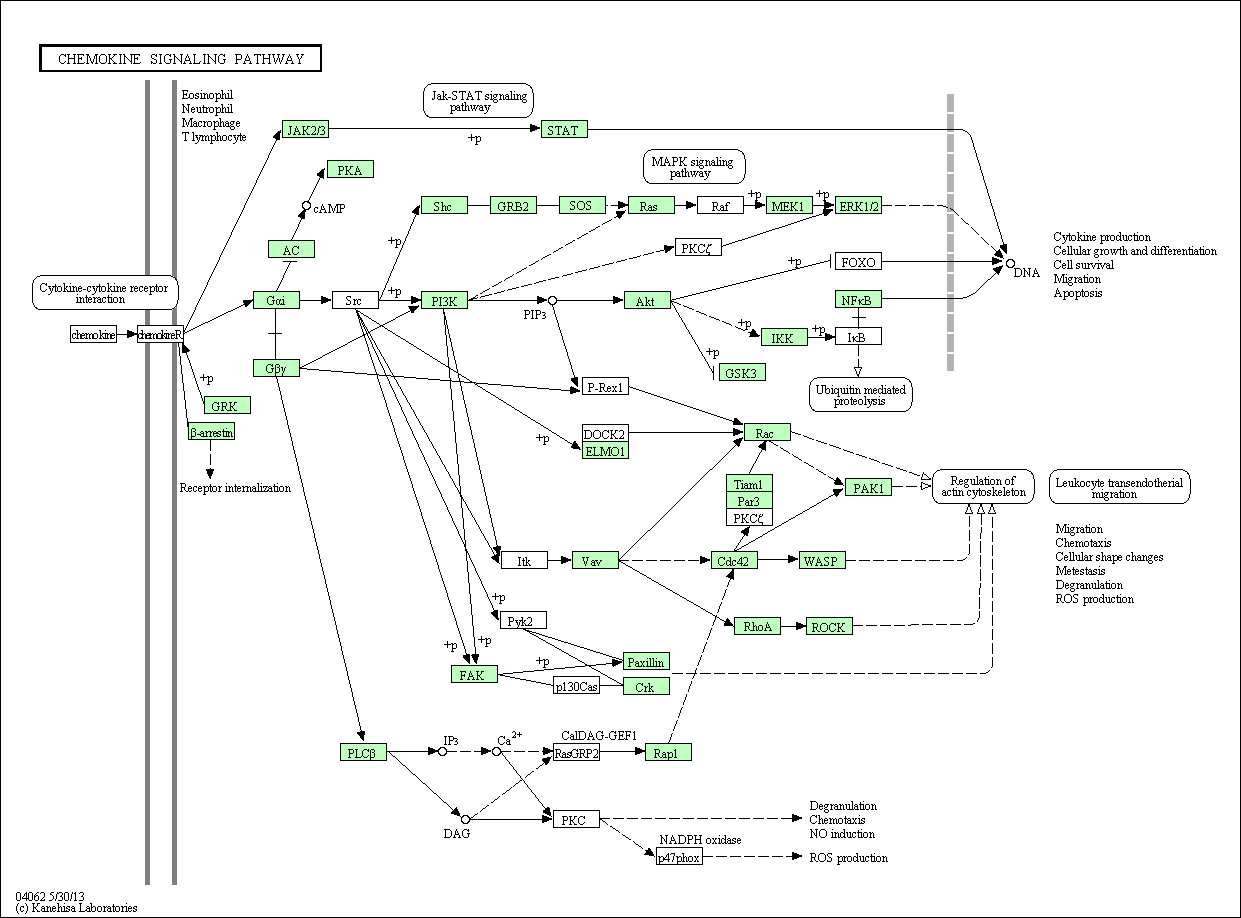

Supplement: Supplementary file 3 — Additional file 3: KEGG classification and functional maps of assembled contigs. Contigs annotated using KEGG Automatic Annotation Server identified sequences in a broad range of functional groups including developmental pathways and cell signaling. (ZIP 11 MB) [file 12864_2013_7026_MOESM3_ESM.zip › KEGG classification/map/map04062.png]

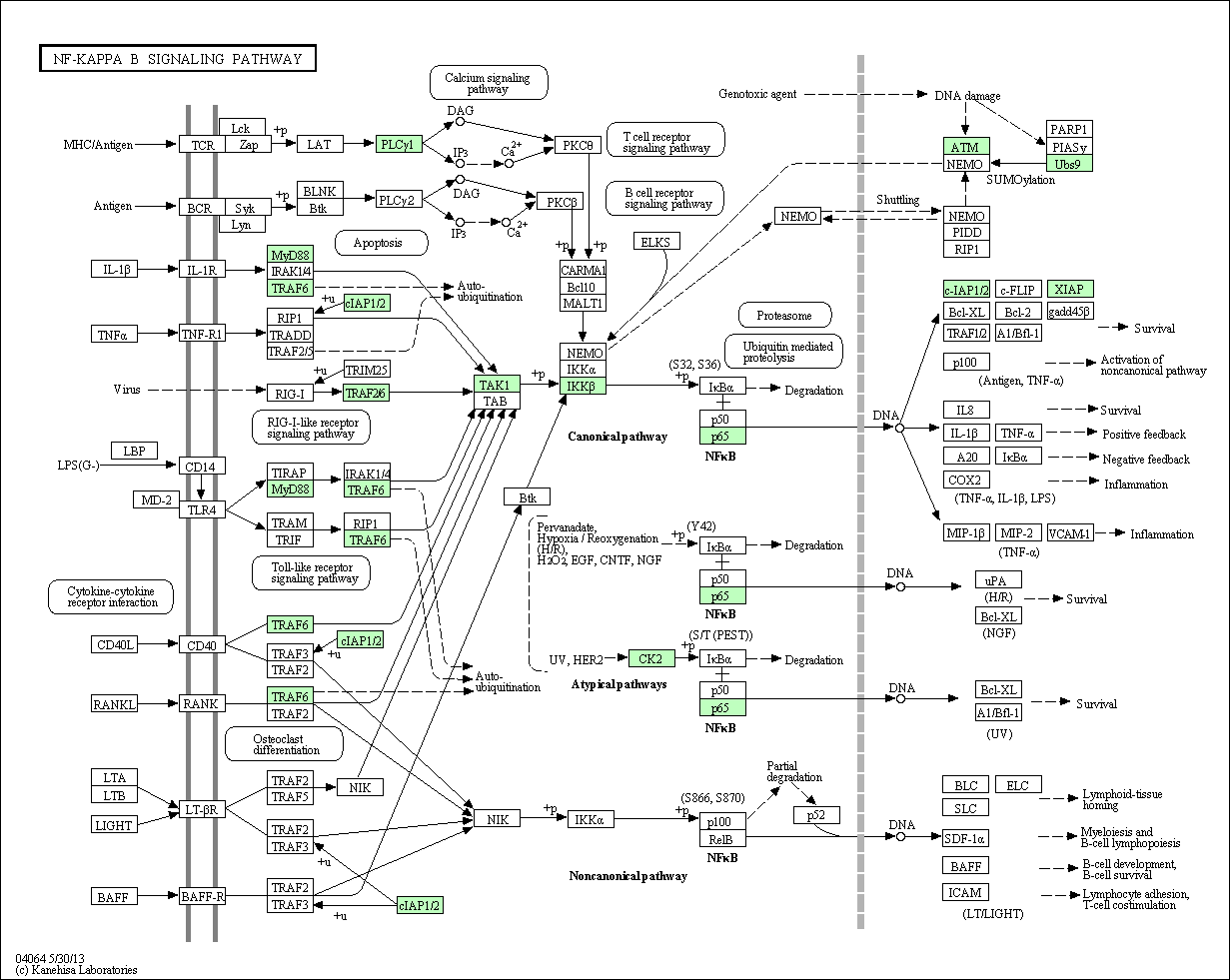

Supplement: Supplementary file 3 — Additional file 3: KEGG classification and functional maps of assembled contigs. Contigs annotated using KEGG Automatic Annotation Server identified sequences in a broad range of functional groups including developmental pathways and cell signaling. (ZIP 11 MB) [file 12864_2013_7026_MOESM3_ESM.zip › KEGG classification/map/map04064.png]

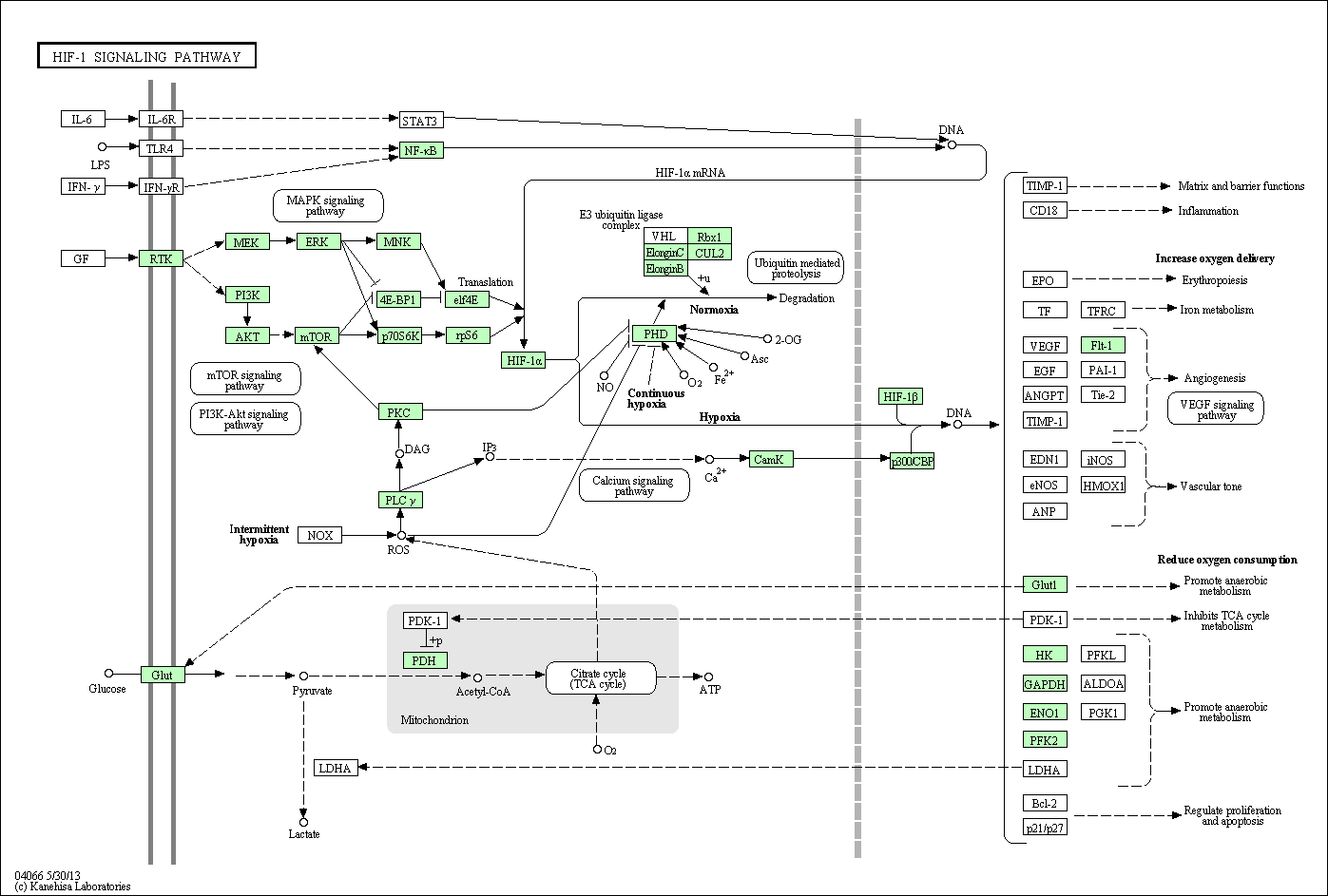

Supplement: Supplementary file 3 — Additional file 3: KEGG classification and functional maps of assembled contigs. Contigs annotated using KEGG Automatic Annotation Server identified sequences in a broad range of functional groups including developmental pathways and cell signaling. (ZIP 11 MB) [file 12864_2013_7026_MOESM3_ESM.zip › KEGG classification/map/map04066.png]

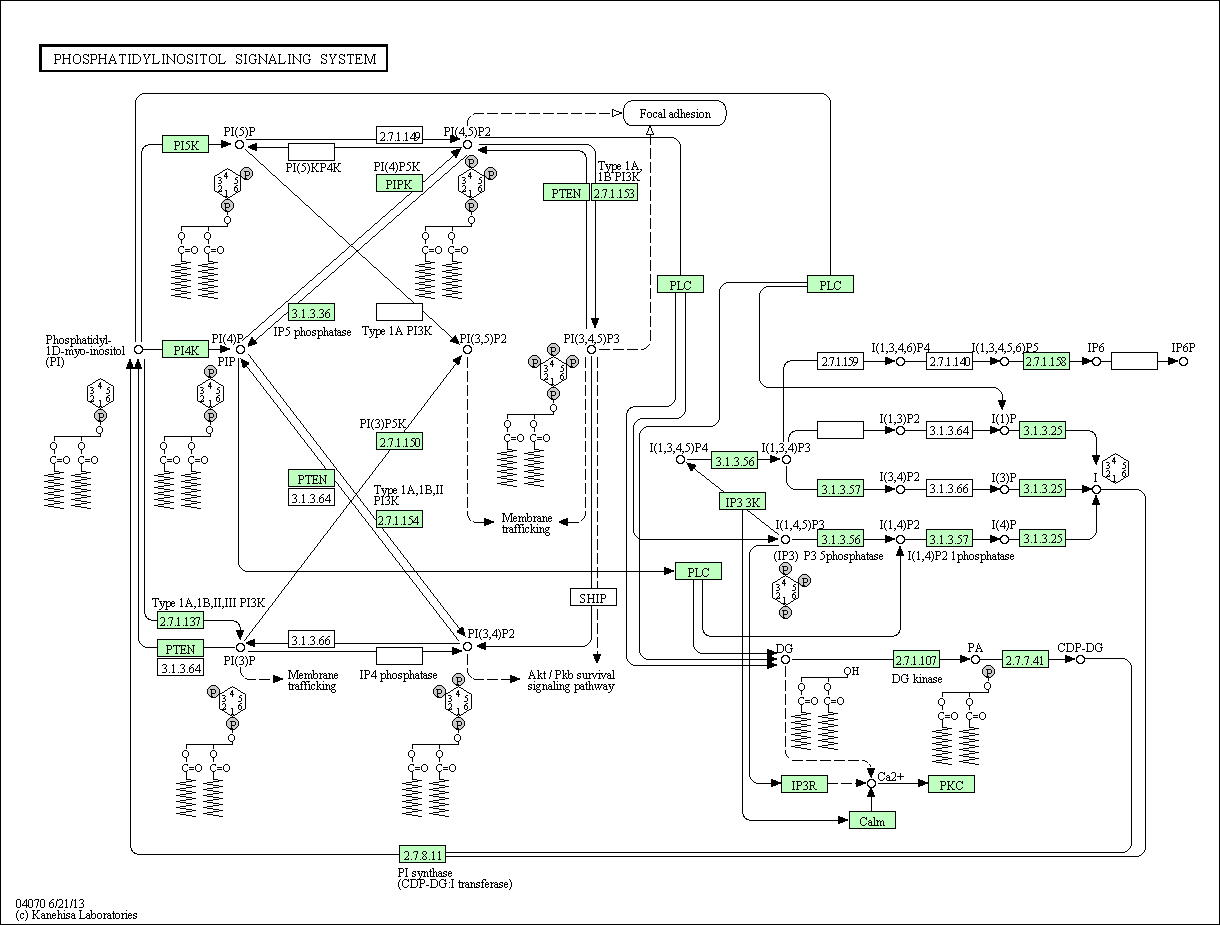

Supplement: Supplementary file 3 — Additional file 3: KEGG classification and functional maps of assembled contigs. Contigs annotated using KEGG Automatic Annotation Server identified sequences in a broad range of functional groups including developmental pathways and cell signaling. (ZIP 11 MB) [file 12864_2013_7026_MOESM3_ESM.zip › KEGG classification/map/map04070.png]

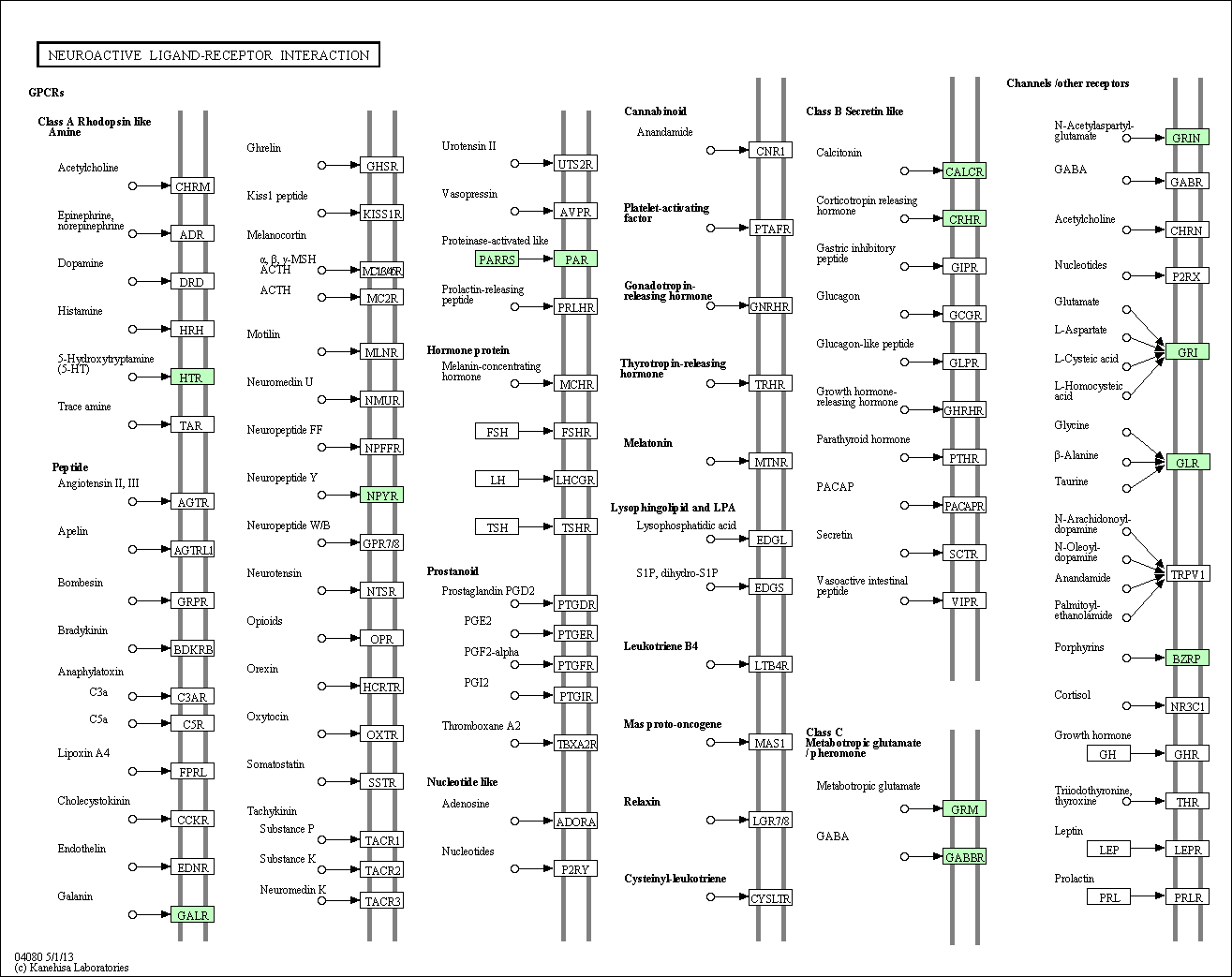

Supplement: Supplementary file 3 — Additional file 3: KEGG classification and functional maps of assembled contigs. Contigs annotated using KEGG Automatic Annotation Server identified sequences in a broad range of functional groups including developmental pathways and cell signaling. (ZIP 11 MB) [file 12864_2013_7026_MOESM3_ESM.zip › KEGG classification/map/map04080.png]

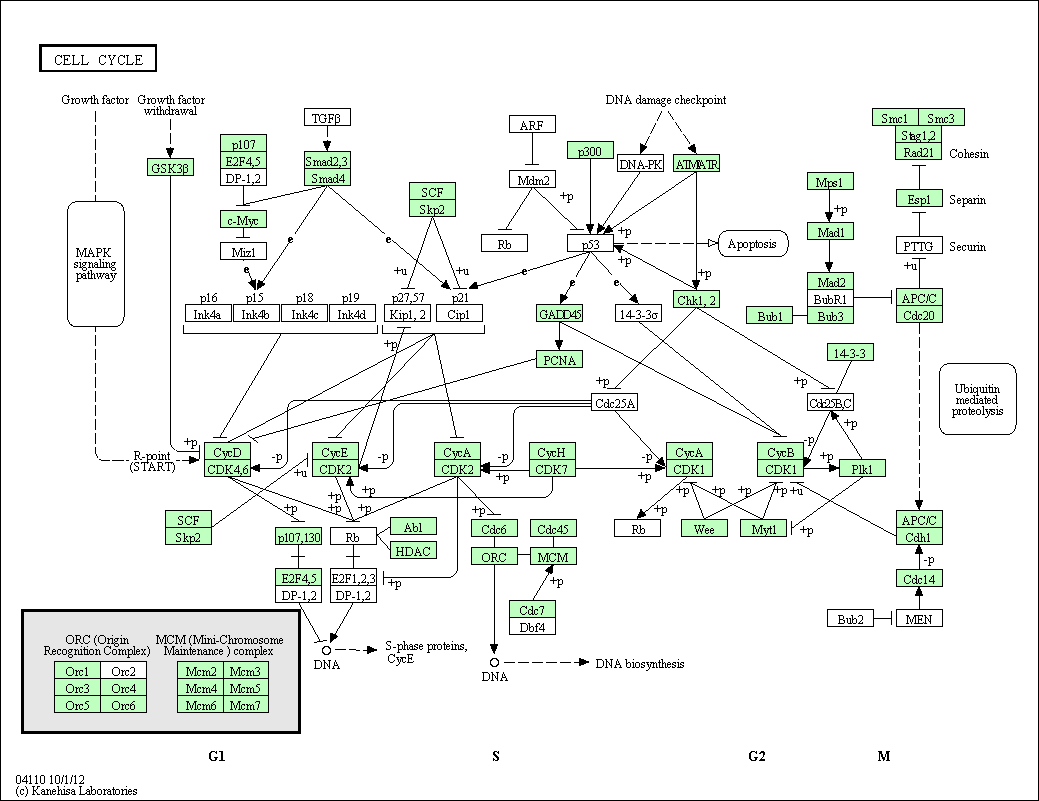

Supplement: Supplementary file 3 — Additional file 3: KEGG classification and functional maps of assembled contigs. Contigs annotated using KEGG Automatic Annotation Server identified sequences in a broad range of functional groups including developmental pathways and cell signaling. (ZIP 11 MB) [file 12864_2013_7026_MOESM3_ESM.zip › KEGG classification/map/map04110.png]

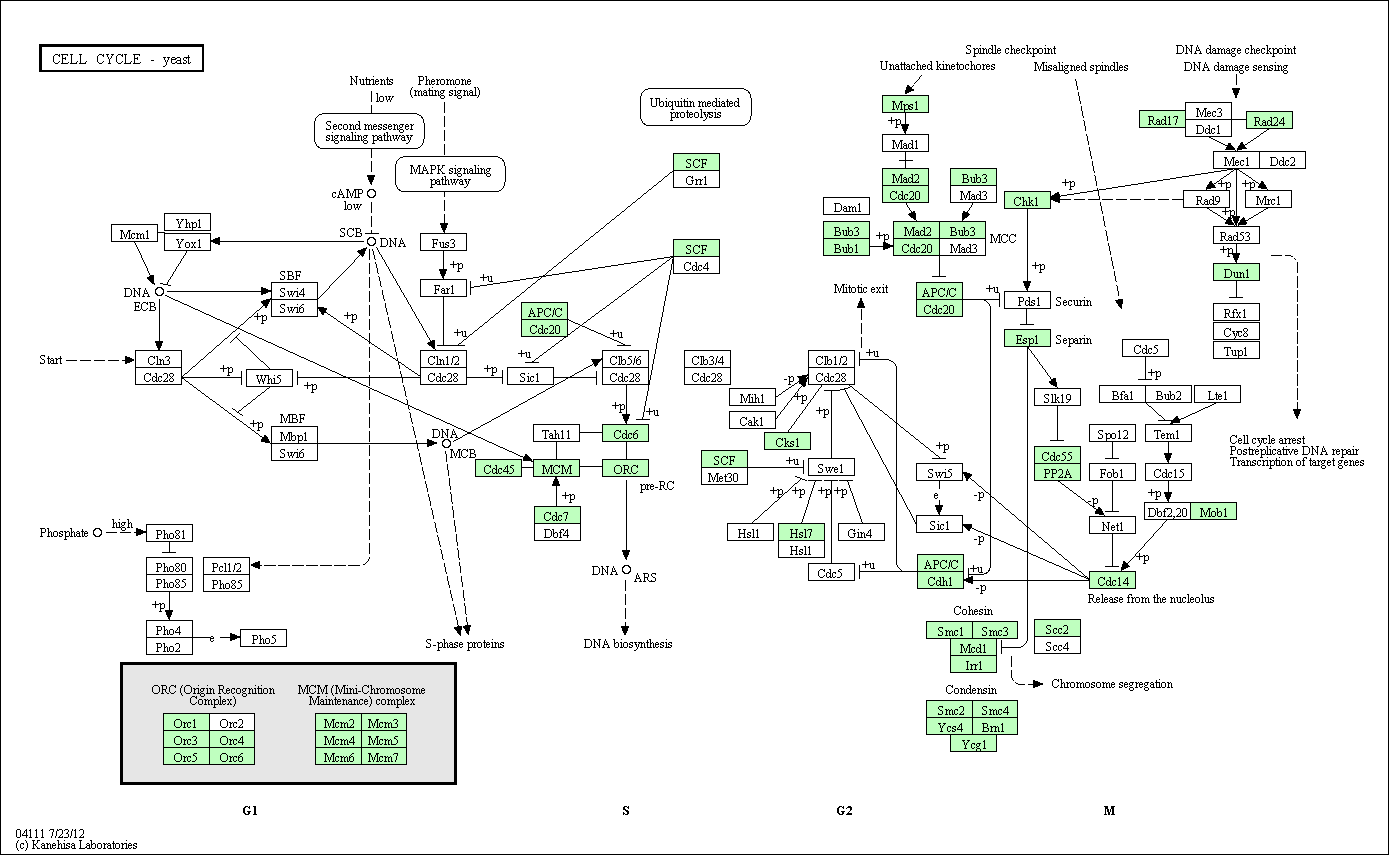

Supplement: Supplementary file 3 — Additional file 3: KEGG classification and functional maps of assembled contigs. Contigs annotated using KEGG Automatic Annotation Server identified sequences in a broad range of functional groups including developmental pathways and cell signaling. (ZIP 11 MB) [file 12864_2013_7026_MOESM3_ESM.zip › KEGG classification/map/map04111.png]

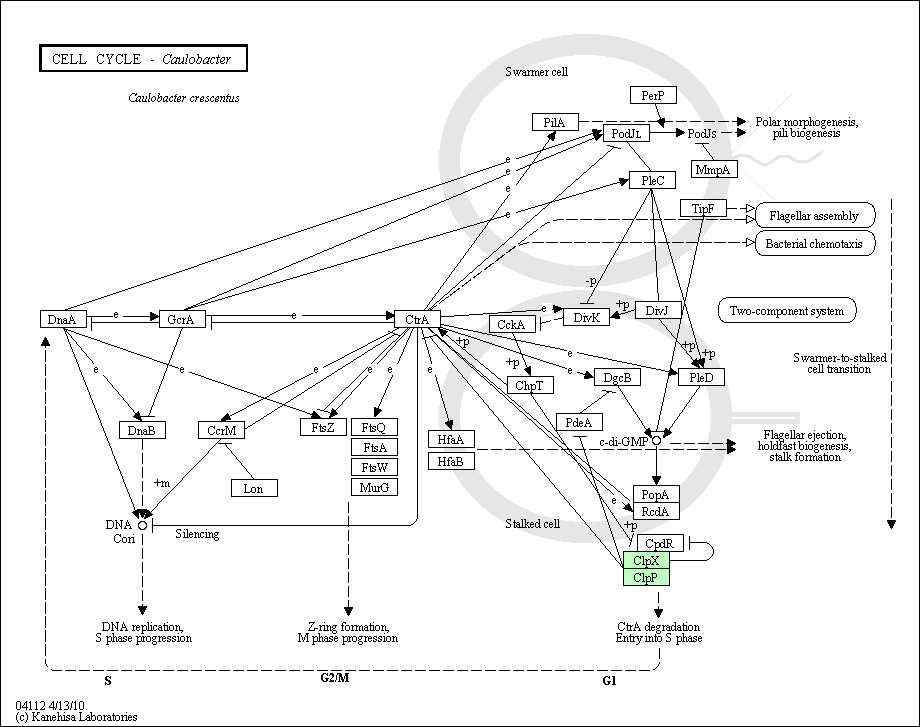

Supplement: Supplementary file 3 — Additional file 3: KEGG classification and functional maps of assembled contigs. Contigs annotated using KEGG Automatic Annotation Server identified sequences in a broad range of functional groups including developmental pathways and cell signaling. (ZIP 11 MB) [file 12864_2013_7026_MOESM3_ESM.zip › KEGG classification/map/map04112.png]

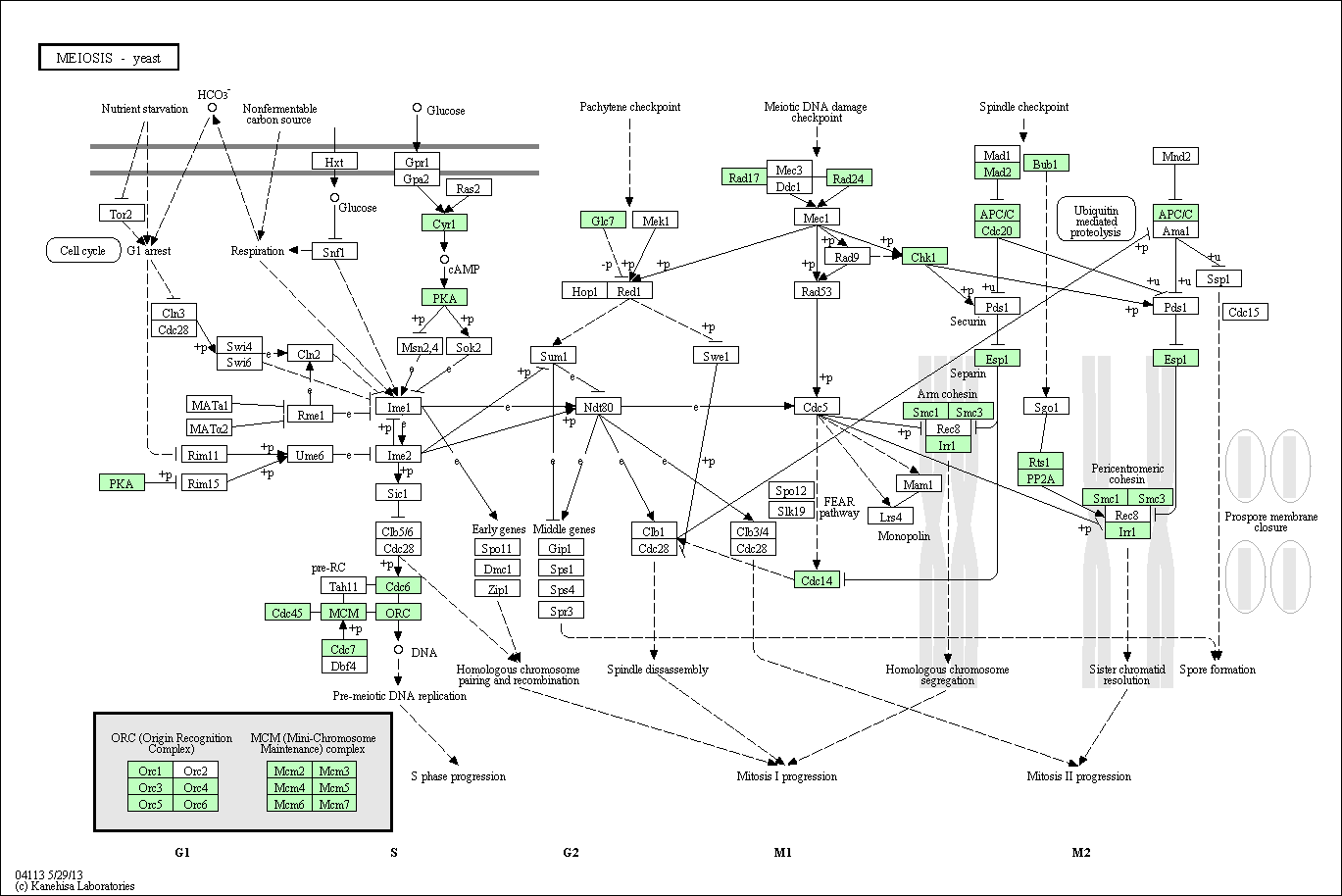

Supplement: Supplementary file 3 — Additional file 3: KEGG classification and functional maps of assembled contigs. Contigs annotated using KEGG Automatic Annotation Server identified sequences in a broad range of functional groups including developmental pathways and cell signaling. (ZIP 11 MB) [file 12864_2013_7026_MOESM3_ESM.zip › KEGG classification/map/map04113.png]

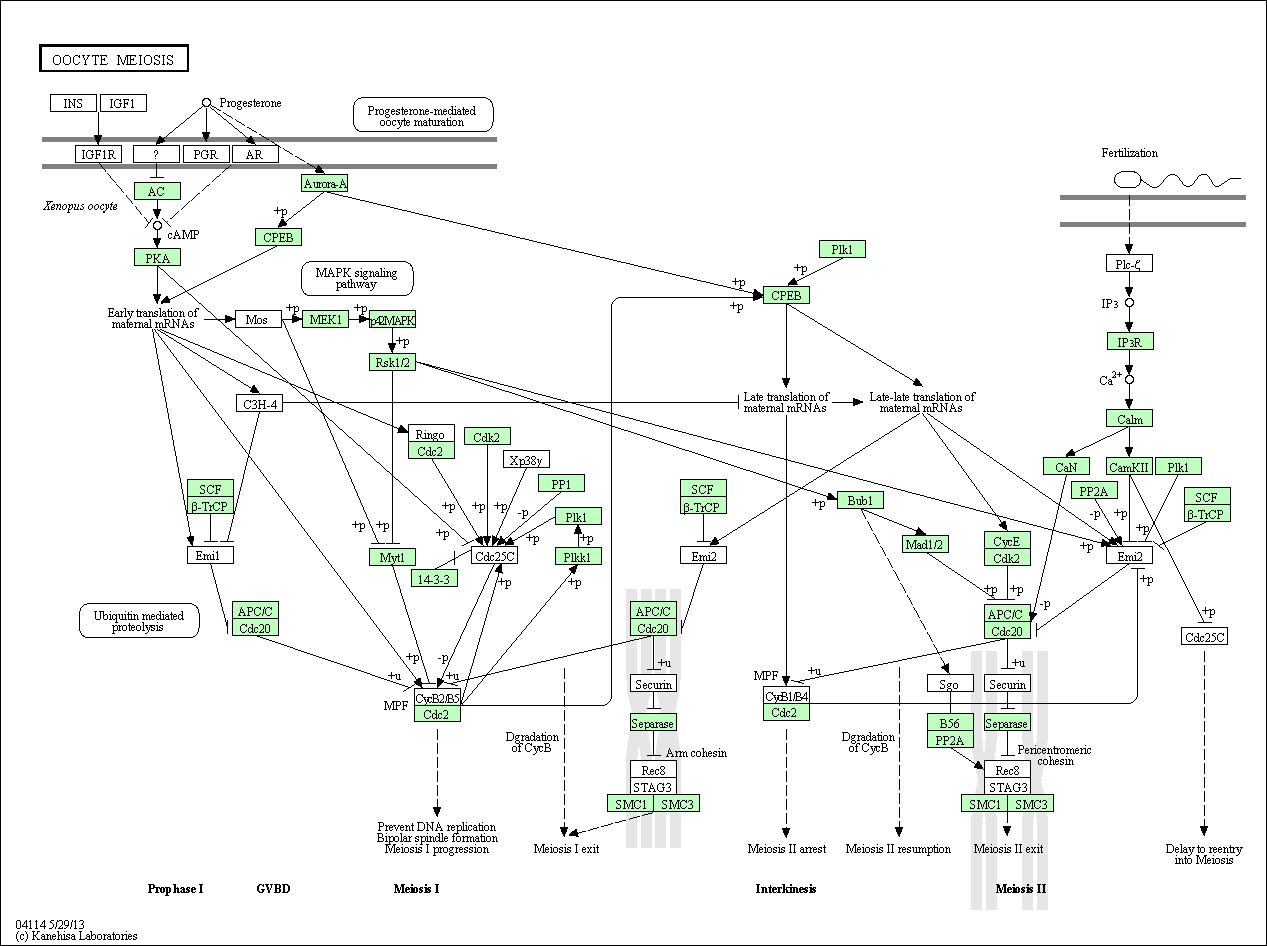

Supplement: Supplementary file 3 — Additional file 3: KEGG classification and functional maps of assembled contigs. Contigs annotated using KEGG Automatic Annotation Server identified sequences in a broad range of functional groups including developmental pathways and cell signaling. (ZIP 11 MB) [file 12864_2013_7026_MOESM3_ESM.zip › KEGG classification/map/map04114.png]

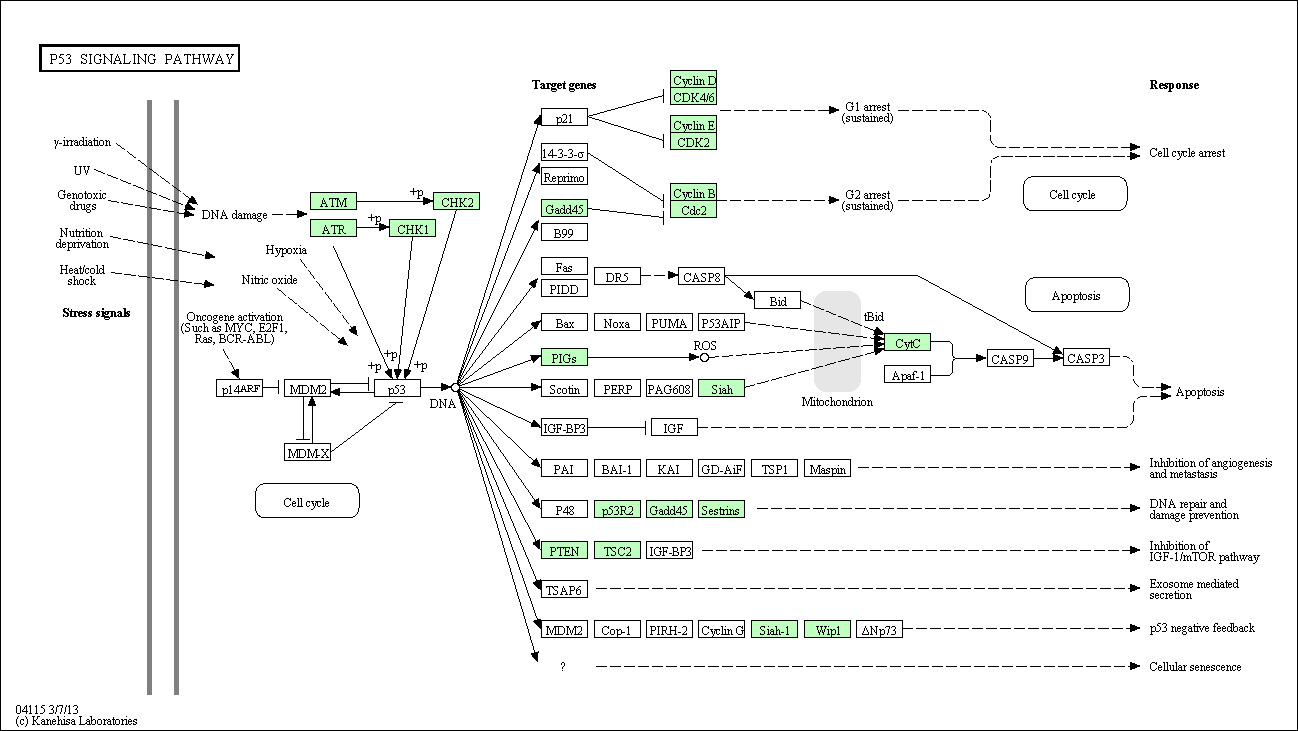

Supplement: Supplementary file 3 — Additional file 3: KEGG classification and functional maps of assembled contigs. Contigs annotated using KEGG Automatic Annotation Server identified sequences in a broad range of functional groups including developmental pathways and cell signaling. (ZIP 11 MB) [file 12864_2013_7026_MOESM3_ESM.zip › KEGG classification/map/map04115.png]

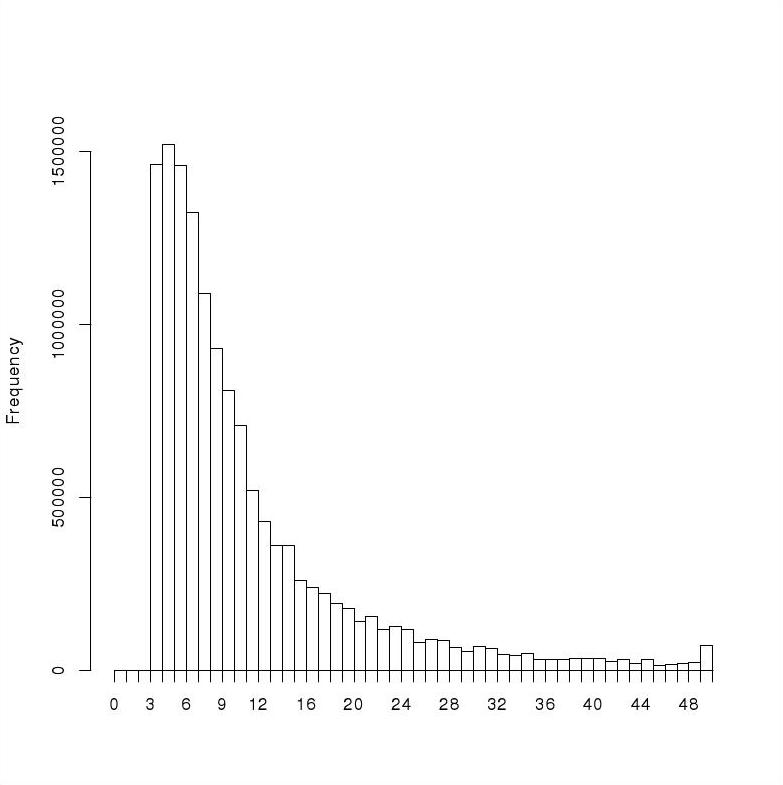

Supplement: Supplementary file 4 — Additional file 4: Average distribution of coverage of T. biloba contigs. Coverage estimates were generated using the Velvet software. (JPEG 33 KB) [file 12864_2013_7026_MOESM4_ESM.jpeg]
